# Molecular evolution of the reactive oxygen-generating NADPH oxidase (Nox/Duox) family of enzymes

**DOI:** 10.1186/1471-2148-7-109

**Published:** 2007-07-06

**Authors:** Tsukasa Kawahara, Mark T Quinn, J David Lambeth

**Affiliations:** 1 Department of Pathology and Laboratory Medicine, Emory University School of Medicine, Atlanta, Georgia, 30322, USA; 2 Department of Veterinary Molecular Biology, Montana State University, Bozeman, Montana, 59717 USA

## Abstract

**Background:**

NADPH-oxidases (Nox) and the related Dual oxidases (Duox) play varied biological and pathological roles via regulated generation of reactive oxygen species (ROS). Members of the Nox/Duox family have been identified in a wide variety of organisms, including mammals, nematodes, fruit fly, green plants, fungi, and slime molds; however, little is known about the molecular evolutionary history of these enzymes.

**Results:**

We assembled and analyzed the deduced amino acid sequences of 101 Nox/Duox orthologs from 25 species, including vertebrates, urochordates, echinoderms, insects, nematodes, fungi, slime mold amoeba, alga and plants. In contrast to ROS defense enzymes, such as superoxide dismutase and catalase that are present in prokaryotes, ROS-generating Nox/Duox orthologs only appeared later in evolution. Molecular taxonomy revealed seven distinct subfamilies of Noxes and Duoxes. The calcium-regulated orthologs representing 4 subfamilies diverged early and are the most widely distributed in biology. Subunit-regulated Noxes represent a second major subdivision, and appeared first in fungi and amoeba. Nox5 was lost in rodents, and Nox3, which functions in the inner ear in gravity perception, emerged the most recently, corresponding to full-time adaptation of vertebrates to land. The sea urchin *Strongylocentrotus purpuratus *possesses the earliest Nox2 co-ortholog of vertebrate Nox1, 2, and 3, while Nox4 first appeared somewhat later in urochordates. Comparison of evolutionary substitution rates demonstrates that Nox2, the regulatory subunits p47*phox *and p67*phox*, and Duox are more stringently conserved in vertebrates than other Noxes and Nox regulatory subunits. Amino acid sequence comparisons identified key catalytic or regulatory regions, as 68 residues were highly conserved among all Nox/Duox orthologs, and 14 of these were identical with those mutated in Nox2 in variants of X-linked chronic granulomatous disease. In addition to canonical motifs, the B-loop, TM6-FAD, VXGPFG-motif, and extreme C-terminal regions were identified as important for Nox activity, as verified by mutational analysis. The presence of these non-canonical, but highly conserved regions suggests that all Nox/Duox may possess a common biological function remained in a long history of Nox/Duox evolution.

**Conclusion:**

This report provides the first comprehensive analysis of the evolution and conserved functions of Nox and Duox family members, including identification of conserved amino acid residues. These results provide a guide for future structure-function studies and for understanding the evolution of biological functions of these enzymes.

## Background

Reactive oxygen species (ROS) [e.g., superoxide anion (O_2_^-^), hydrogen peroxide (H_2_O_2_)] are thought of as cytotoxic and mutagenic; however, recent data point to important biological roles for ROS [1-4]. Phagocytes generate large amounts of O_2_^- ^as part of their microbicidal activity, which results from activation of a membrane-associated NADPH oxidase. The key redox component of the oxidase is flavocytochrome *b*_558_, which is comprised of an O_2_^-^-generating catalytic subunit, gp91*phox *(a.k.a., Nox2), and a non-catalytic subunit, p22*phox *[5-7]. Recent studies indicate that similar NADPH oxidase systems are present in a wide variety of non-phagocytic cells. While the nature of these non-phagocyte NADPH oxidases is still being defined, it is clear that they are functionally and structurally distinct from the phagocyte oxidases.

**Figure 1:**
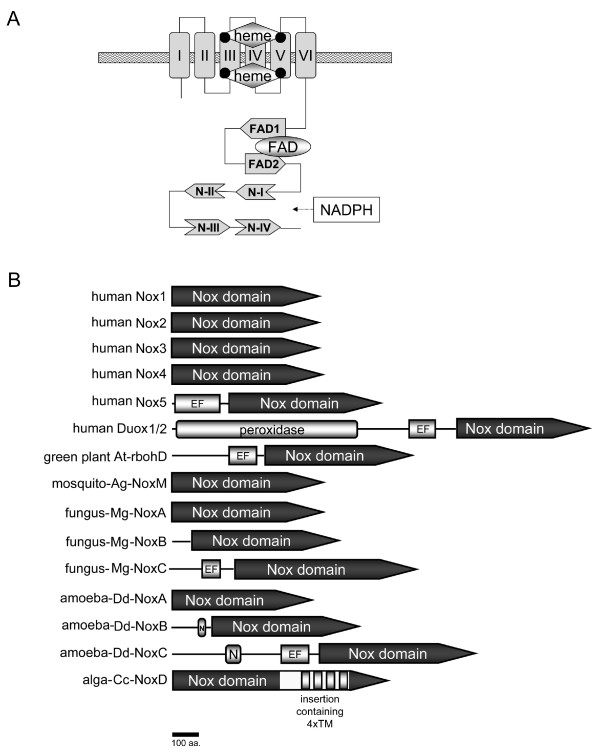
**Schematic domain structures of Nox family**. (*A*) The Nox domain possesses 6 transmembrane α-helices (I through VI in *boxes*), two hemes ("heme" in *diamonds*), and predicted sub-regions that provide binding cavities for a co-enzyme FAD (FAD1 and FAD2) and for a substrate NADPH (N-I, N-II, N-III, and N-IV). (*B*) All members of Nox/Duox family contain the Nox domains. Abbreviations are: "EF" refers to any domain containing one or more EF-hand motifs; "peroxidase" refers to a region that is homologous to a heme-containing peroxidase; "N" refers to an asparagine-rich region; "4 × TM", four predicted transmembrane α-helical segments; "At-rbohD" is *A. thaliana *respiratory burst oxidase homolog-D; "Ag" is *A. gambiae*; "Dd" is *D. discoideum*; "Mg" is *M. grisea*; "Cc" is *C. crispus*.

Nox2 has four conserved histidines that ligate two hemes between the 3^rd ^and 5^th ^of 6 transmembrane (TM) α-helices and subregions that fold to provide binding cavities for FAD and NADPH [2,5,7-10] (referred to here as the "Nox domain" as shown in Figure 1A). These canonical regions occupy about 35% of the Nox2 sequence; however, little is known about essential roles for the other regions. Recently, mammalian homologues of Nox2 were identified and now constitute the Nox/Duox family [2]. In humans, there are five Noxes (Nox1-5) plus Duox1 and Duox2 (Figure 1B). The activation of Nox2 has been extensively studied and requires the association of essential cofactors. Nox2 alone is inactive and must associate with p22*phox *to form a non-covalent heterodimer known as flavocytochrome *b*_558_. Additional regulatory subunits consist of p67*phox*, p47*phox*, p40*phox *and the small GTPase Rac [2,5,6]. Upon cell activation, these subunits translocate from the cytosol, assembling with and activating flavocytochrome *b*_558 _at the cell or phagosomal membrane. Respective homologues of p47*phox *and p67*phox*, "Nox Organizer 1" (NOXO1) and "Nox Activator 1" (NOXA1), regulate Nox1 [11-15], and NOXO1 also activates Nox3 [16-18]. In comparison, Nox4 requires p22*phox *but no other subunits [19-21]. All members of the human Nox/Duox family contain a flavocytochrome moiety, which we refer to as the "Nox domain". Nox5 consists of a Nox domain plus an N-terminal EF-hand-containing calcium-binding domain [22,23]. Duox1/2 build on the Nox5 structure by adding an N-terminal peroxidase domain [24,25]. Nox/Duox family members are reported in mouse, rat, cow, guinea pig, *Takifugu rubripes *(*T. rubripes*), *Caenorhabditis elegans *(*C. elegans*), *Ciona intestinalis *(*C. intestinalis*), sea urchin, fungi, cellular slime mold amoeba *Dictyostelium discoideum *(*D. discoideum*), red alga *Chondus crispus *(*C. crispus*) and *Porphyra yezoensis *(*P. yezoensis*), and green plants, including *Arabidopsis thaliana *(*A. thaliana*) [22,24,26-41]. Plant Noxes are similar in domain structure to Nox5 [26,42]. The EF-hands of human Nox5, human Duox2, and an *A. thaliana *Nox are essential for calcium-stimulated activity [23,25,26]. Red alga *C. crispus *and *P. yezoensis *possess an unusual Nox enzyme [31]. The algal Nox lacks an N-terminal EF-hand region and no regulatory subunit homologs are present; however, the algal Nox has 4 additional predicted transmembrane α-helices that are situated between the 1^st ^and 2^nd ^NADPH-binding sub-regions [31].

Genetic approaches have implicated Nox/Duox-derived ROS in biological roles and pathological conditions, including hypertension (Nox1), innate immunity (Nox2, Duox), suppression of pathogen-induced cell death (plant Nox), stomatal closure (plant Nox), otoconia formation in the inner ear (Nox3), biosynthesis of extracellular matrix (Duox), and thyroid hormone biosynthesis (Duox1/2) [18,24,29,43-48]. Although widely expressed, little is known about evolutionary relationships among Nox proteins.

Herein, we analyzed Nox/Duox protein sequences from 14 vertebrates, one urochordate, one echinodermate, three insects, one nematode, four fungi, two red algae, one amoeba, and one green plant. Using this large data set, we report (i) a novel molecular taxonomy and phylogeny of Nox/Duox proteins, (ii) synteny of vertebrate Nox/Duox genes by genome annotation, (iii) evolutionary substitution rates of vertebrate Nox proteins and regulatory subunits, and (iv) identification of key amino acid residues and regions conserved among all Nox proteins.

## Results

### Molecular taxonomy of Nox domains

We assembled deduced amino acid sequences from 101 Nox/Duox genes (see Additional file 4). Each Nox candidate was preliminarily aligned with human Noxes to check whether the sequences conserved canonical regions required for O_2_^- ^generation, such as the four heme-ligating histidines corresponding to His-101, His-115, His-209, His-222 of human Nox2 (GenBank™ No. NM_000388). Nox genes are present in most eukaryotes including vertebrates, urochordates, echinodermates, nematodes, insects, fungi, plants amoeba, and red alga, but not in prokaryotes. Schematic domain structures of Nox/Duox family proteins of human, the green plant *A. thaliana*, fungus *Magnaporthe grisea *(*M. grisea*), the cellular slime mold amoeba (*D. discoideum*), and the red alga (*C. crispus*) are shown in Figure 1B. All members of Nox/Duox family expressed a Nox domain containing the six transmembrane segments and flavocytochrome moiety (Figure 1A). In addition, Nox5, Duoxes, At-rboh-D, fungal NoxC, and amoeba NoxC all contained an EF hand-containing calcium-binding domain (for details, see the section for EF-hand motif). Duoxes also contained a peroxidase homology domain; whereas, amoeba Noxes B and C also contained an asparagine-rich region (labled "N in Figure 1B). The malaria mosquito *Anopheles gambiae *(*A. gambiae*) genome encoded one unique Nox gene, Nox-mosquito (referred to here as "NoxM"), which encodes only the Nox domain and no calcium-binding domain (Figure 1B).

**Figure 2:**
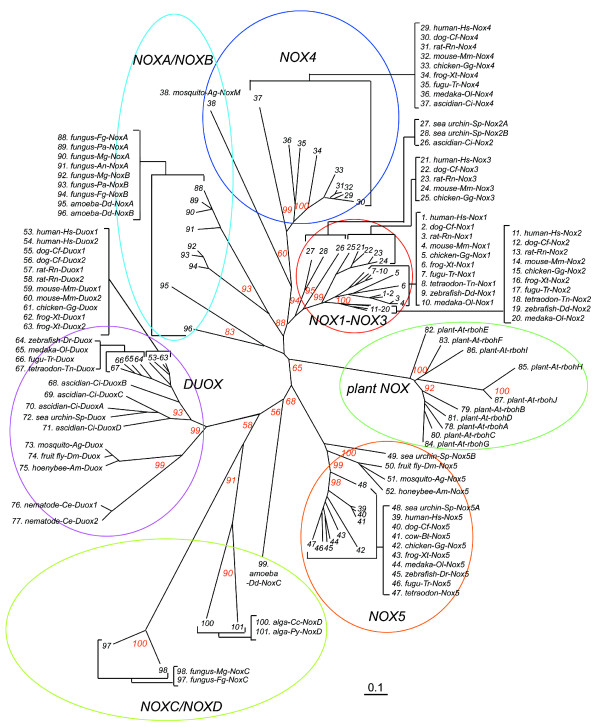
**Molecular taxonomy of the Nox domains of Nox/Duox proteins**. Amino acid sequences of the following species were trimmed to the length corresponding to human Nox2 and were aligned: human-Hs, *H. sapiens*; Cow-Bt, *B. Taurus*; dog-Cf, *C. familliaris*; rat-Rn, *R. norvegicus*; mouse-Mm, *M. musculus*; opossum-Md, *M. domestica*; chicken-Gg, *G. gallus*; frog-Xt, *X. tropicalis*; zebrafish-Dr, *D. rerio*; fugu-Tr, *T. rubripes*; tetraodon-Tn, *T. nigroviridi*s; medaka-Ol, *O. latipes*; ascidian-Ci, *C. intestinalis*; sea urchin-Sp, *S. purpuratus*; fruit fly-Dm, *D. melanogaster*; mosquito-Ag, *A. gambiae*; honeybee-Am, *A. mellifera*; nematode-Ce, *C. elegans*; plant-At,*A. thaliana*; amoeba-Dd, *D. discoideum*; fungus-Pa, *P. anserina*, fungus-An, *A. nidulans*; fungus-Mg, *M. grisea*; fungus-Fg, *F. graminearum*; alga-Cc, *C. crispus*; and alga-*Py, P. yezoensis*. Each subfamily is indicated by a colored circle, and bootstrap values of 1,000 replications are shown at the major branches as percentages. Evolutionary distances (inferior bar) are equivalent to 0.1 amino acid substitution per site.

A molecular taxonomy, constructed by aligning the deduced amino acid sequences of the Nox domains (omitting other domains and unique features, such as the extra transmembrane domains of algae NoxD) revealed seven subfamilies (Figure 2). The subgroup consisting of Nox1-3 includes Noxes that are regulated by regulatory subunits, including Nox2, the classical phagocyte oxidase catalytic subunit (numbers 11–20 in Figure 2). Nox2 of the urochordate *C. intestinalis *(# 26 in Figure 2) branched from a root common to vertebrate Nox1-3 and conserves features common to all Nox1-3 proteins. Sea urchin *S. purpuratus *belongs to Echinodermata, which diverged early from an ancestor common to urochordates and vertebrates [49], and the *S. purpuratus *genome had two Nox2-like proteins, Nox2A and Nox2B (#27 and #28 in Figure 2), which branched from a root common to all chordate Noxes 1–3. The taxonomy indicates that the *S. purpuratus *Nox2 isologs represent a branch closest to the primordial ancestor of all isologs of vertebrate Nox1, Nox2 and Nox3. The Nox4 subfamily includes a urochordate isolog and branched from a root close to the Nox1-3 subfamily. Both the Nox4 and the Nox1-3 subfamilies originated from a common branch, and together form the two subgroups that are known to require a p22*phox *subunit for activity and/or stability [19,20,50]. Neither of these subgroups occurs in green plants, fungi, or nematodes. For example, the *S. purpuratus *genome did not contain a distinct Nox4 ortholog. Although the fruit fly *Drosophila melanogaster *(*D. melanogaster*) and honeybee *Apis mellifera *(*A. mellifera*) did not possess an ortholog of Nox1-3 or Nox4, the malaria mosquito *A. gambiae *possessed one unique Nox gene, NoxM (mosquito-Ag NoxM, #38 in Figure 2). We also searched for the gene in another mosquito genome *Aedes aegypti *(*A. aegypti*), the principal vector of yellow and dengue fevers, using the NCBI BLAST server [51]. The *A. aegypti *genome contained one NoxM ortholog (GenBank™ No. EAT37894) similar to *A. gambiae *NoxM, and also had Nox5 and Duox orthologs (GenBank™ No. EAT46816 and EAT40728, respectively). The *A. gambiae *NoxM protein branched from a root common to all chordate Nox 4 (Figure 2); however, the bootstrap value of the branch was only 60%, making it unclear whether NoxM belongs to the p22*phox*-regulated subgroups (Nox4 and Nox1-3) or to the NoxA/NoxB subgroup. The *A. gambiae *genome did not encode a distinct p22*phox *ortholog, suggesting that the NoxA/NoxB subgroup (which is p22*phox*-independent) may be a more appropriate assignment for this NoxM.

The Nox5 subgroup was composed of the orthologs (#'s 39–52 in Figure 2) present in vertebrates [except for *Mus muscles *(*M. muscles*) and *Rattus norvegicus *(*R. norvegicus*)], echinoderm, and insects; however, Nox5 was not found in the urochordate *C. intestinalis*, or the nematode *C. elegans*. A Nox5 ortholog of a green-spotted pufferfish *Tetraodon nigroviridis *(*T. nigroviridis*) was classified in the Nox5 subgroup, as shown in Figure 2 (# 47), but the predicted protein does not contain the N-terminal extension with the calcium-binding domain that is present in other Nox5 orthologs (amino acid sequence is shown in Additional file 4). However, the nucleotide sequence around the presumed start codon (gaggcaugc, methinone codon underlined) also did not match to a consensus Kozak sequence [cc(a/g)ccaugg] [52], suggesting that the reported sequence is likely to be incomplete. A Nox5 ortholog (# 45) of zebrafish *Danio rerio *(*D. rerio*) also did not contain the presumed start codon and the calcium-binding domain (sequence is shown in Additional file 4), suggesting that the sequence of this Nox5 ortholog is also incomplete. The genome of sea urchin *S. purpuratus *encodes two Nox5 isologs, Nox5A and Nox5B (Figure 2). The Duox subgroup showed broad expression in Bilateria, such as vertebrates, urochordates, echinoderms, nematodes and insects, but was not found in amoeba, fungi, or plants. Plant Nox homologs, previously termed "respiratory burst oxidase homologues (rboh)" [26], formed a distinct subgroup (Figure 2), and *A. thaliana *had 10 rboh homologues, suggesting specialized functions or tissue expression.

Noxes representing the NoxA/NoxB subgroup and the NoxC subgroup were present in fungi, and all fungi examined contained both NoxA and NoxB, except for *Aspergillus nidulans *(*A. nidulans*), which possessed only a NoxA gene. Yeast [*Schizosaccharomyces pombe *(*S. pombe*) and *Saccharomyces cerevisiae *(*S. cerevisiae*)] did not possess any Noxes. The domain structure of the NoxA ortholog is similar to that of Nox1-Nox4; whereas, NoxB proteins also have a short N-terminal extension that does not contain any recognizable domains or motifs (Figure 1B). Fungal NoxC, present in *M. grisea *and *Fusarium graminearum *(*F. graminearum*), has an N-terminal EF-hand domain (Figure 1B). The slime mold amoeba *D. discoideum*, a protozoan that straddles the boundary between animals and plants [53], contained three Nox isologs, NoxA, NoxB, and NoxC (Figure 1B). Amoeba NoxC has an EF-hand domain as well as an N-terminal extension containing an asparagine-rich region ("N", Figure 1B); however, NoxA and NoxB both lack the EF-hand domain (Figure 1B). The EF-hand-containing subfamilies (Nox5, Duox, NoxC, and plant Nox) were the most abundant of the Noxes, comprising well over half of the taxonomic tree (Figure 2). Unlike other members of the NoxC/NoxD family, algal Noxes (#s 100 and 101 in Figure 2) do not contain an EF-hand domain (Figure 1B), but branched from a root shared by amoeba and fungal NoxC (Figure 2). Therefore, we refer to these algal Nox proteins that lack an EF hand domain as NoxD, which together with NoxC form a distinct subgroup. Because they share structural features common to EF hand-containing Noxes, we speculate that that these Noxes may be regulated by an as-yet unknown calcium-binding protein.

### Synteny of Nox/Duox genes in vertebrates

**Figure 3:**
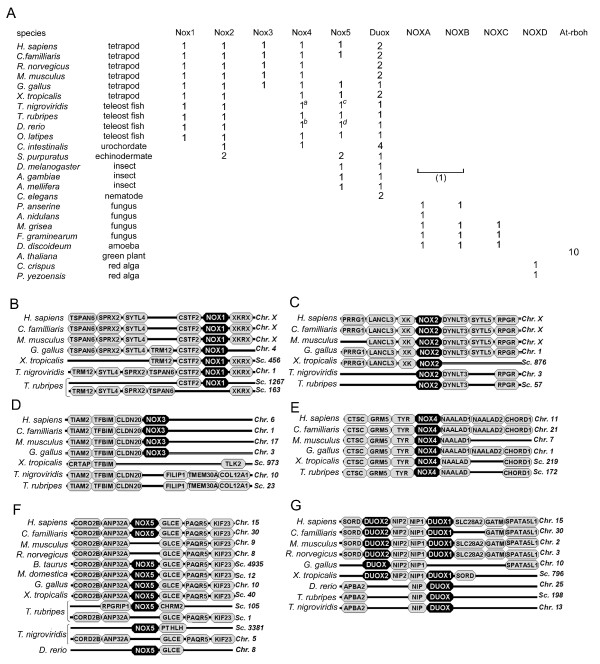
**Syntenies of Nox/Duox genes**. (*A*) Summary of the occurrence of Nox/Duox genes within eukaryotes. The *number *indicates the orthologs in each organism. Superscripted letters "a" and "b" represent incomplete amino acid sequences predicted from nucleotide fragments: a, 221 amino acid length of *T. nigroviridis *Nox4 and b, 201 amino acids of *D. rerio *Nox4 (sequences are shown in SD1). Superscripted letters "c" and "d" indicate these Nox5 orthologs do not contain N-terminal EF-hand-containing domain (#45 and #47 in Figure 2; sequences are shown in Additional file 4). *A parenthesis *represents an ambiguous classification of the "Ag-NoxM" (#38 in Figure 2) in the NoxA/NoxB subgroup. (*B*-*G*) Syntenies of the indicated vertebrate Noxes are shown. Genes are aligned in columns to illustrate orthology. Chromosome (*Chr*) or scaffold (*Sc*) numbers are indicated on the right.

A summary of the occurrence and number of Nox/Duox genes in vertebrates (tetrapods, teleost fish), urochordate, echinodermate, nematode, insects, fungi, green plants amoeba, and red alga is shown in Figure 3A. Although we performed extensive BLAST searches [54,55], some Noxes did not appear to be present in all vertebrate genomes. For example, a rat or mouse ortholog of Nox5 and a frog *Xenopus tropicalis *(*X. tropicalis*) or teleost fish ortholog of Nox3 was not found (Figure 3A). Teleost fish listed in Figure 3A had one complete Duox ortholog, but no paralog corresponding to mammalian Duox2. To substantiate that these genes were indeed absent, we compared chromosomal synteny (preserved orders of genes between related organisms) of Nox/Duox orthologs in vertebrates. Syntenies of *C. intestinalis *Noxes were so divergent from those of vertebrates that it was not possible to perform syntenic analyses for this species. Syntenies of Nox1, Nox2 and Nox4 (Figs. 3B, 3C, and 3E, respectively) were the most highly preserved among Nox/Duox genes.

The human Nox3 gene is positioned following TIAM2, TFBIM and CLDN20 on chromosome 6 (Figure 3D), a synteny that was conserved in mammals and chickens *Gallus gallus *(*G. gallus*). Puffer fish, fugu *T. rubripes *and tetraodon *T. nigroviridis *lacked a Nox3 ortholog, and TIAM2, TFBIM and CLDN20 were followed instead by FILIP1, TMEM30A and COL12A1. In the genome of *X. tropicalis*, there was greater variation in synteny: TFBIM was present, but neither Nox3 nor other linked markers were seen. A Nox3 gene was not found in the genome of zebrafish *D. rerio*, but nucleotide fragments encoding these marker genes of *D. rerio *were too short to demonstrate the absence of Nox3 by syntenic analysis. Thus, Nox3 emerged during evolution sometime after the emergence of fish and amphibians from a common ancestor of birds and mammals.

The synteny of genetic markers surrounding Nox5 was highly conserved in human, dog *Canis familliaris *(*C. familliaris*), mouse, rat, chicken, cow *Bos taurus *(*B. taurus*), opossum *Monodelphis domestica*, (*M. domestica*, a Nox5 ortholog sequence DDBJ™ accession No. BR000304) and frog (Figure 3F). We also found Nox5 gene fragments in rabbit *Oryctolagus cuniculus *(*O. cuniculus*) and armadillo *Dasypus novemcinctus *(*D. novemcinctus*) draft-sequenced genomes (DDBJ accession No. BR000301 and BR000302, respectively). However, rodents (mouse and rat in this study) lacked Nox5, clearly demonstrating that this gene had been lost. Interestingly, pufferfish *T. rubripes *and *T. nigroviridis *had Nox5-like genes, but the gene markers were present on a different scaffold fragment (Figure 3F). Mammals and frog *X. tropicalis *each had two paralogs of Duox (Figure 3G), while chicken had only one. Fish genomes possessed a single Duox gene that followed a single NIP gene (Figure 3G). Like Duox, the NIP gene has also undergone gene duplication to form NIP1 and NIP2. Interestingly, NIP1 and NIP2 in Figure 3G are identical to DuoxA1 and DuoxA2, respectively, proteins that were recently described to participate in the activation and maturation of Duoxes [56]. Due to the complexity of the tetraploid genomes of zebrafish and incomplete genomic sequence, however, we cannot rule out a second Duox in another chromosomal location.

### Structural variations among Nox domains

**Figure 4:**
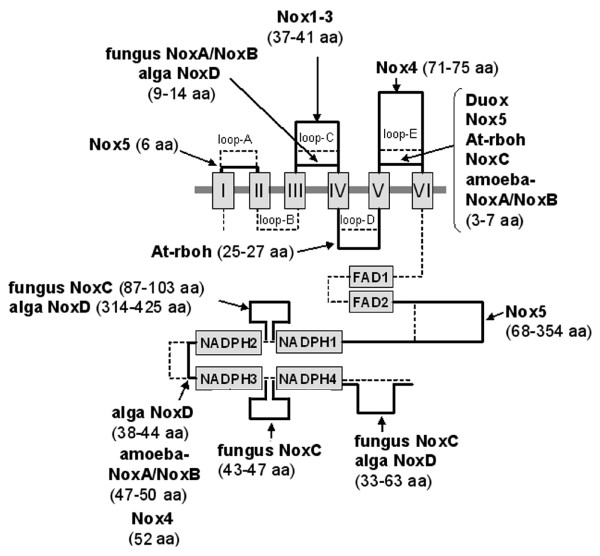
**Loop and segment sizes joining transmembrane regions and canonical NADPH-oxidase domains**. After alignment of canonical and TM domains, the numbers of amino acids linking these regions were counted. I to VI in *boxes *indicate the six predicted TM α-helices, and FAD-binding subregions (FAD1 and FAD2) and the four NADPH-binding subregions (NADPH1 to NADPH4) are shown. Solid lines show the relative length of loops and linkers that are characteristic of specific Nox subfamilies. The numbers of amino acids are indicated. Broken lines show the average lengths. More detailed information on the number of residues linking each of the canonical regions for each Nox subtype is provided in Additional file 1.

The molecular taxonomy of Noxes in Figure 2 suggested that unique structural features characterize each Nox subfamily. Noxes possess five loops (loops A-E) that join TM regions ("I-VI" in Figure 4), and also segments connecting canonical subdomains, such as FAD-binding regions ("FAD1" and "FAD2" in Figure 4) and predicted NADPH-binding regions ("NADPH1-4" in Figure 4) [2,8-10,34]. Alignment of Nox domains demonstrates that each Nox subgroup has characteristic sizes of these loops and segments (Figure 4 and detailed sizes are shown in Additional file 1). For example, Nox1-3 have a longer C-loop, Nox4 has a longer E-loop, and plant Noxes have an extended D-loop (Figure 4). Nox5 proteins have shortened A- and E-loops and a long and variable insertion between FAD- and NADPH-binding domains; whereas, the fungal NoxC and algal NoxD have an extended C-terminus and long insertions between NADPH-binding subregions. Thus, each Nox/Duox ortholog has characteristic structural features conserved throughout evolution. The differences in loop size indicate that the basic catalytic function of the Nox domain is able to tolerate considerable structural variation in this region. Certain loops, especially "loop B" and "TM6-FAD" [the region between 6^th ^transmembrane segment ("VI" in Figure 4) and FAD1-binding subregion ("FAD1" in Figure 4)] were conserved in size among all Noxes, perhaps pointing to their structural or functional importance.

### Substitution rates of Nox domains and Nox regulatory subunits

**Figure 5:**
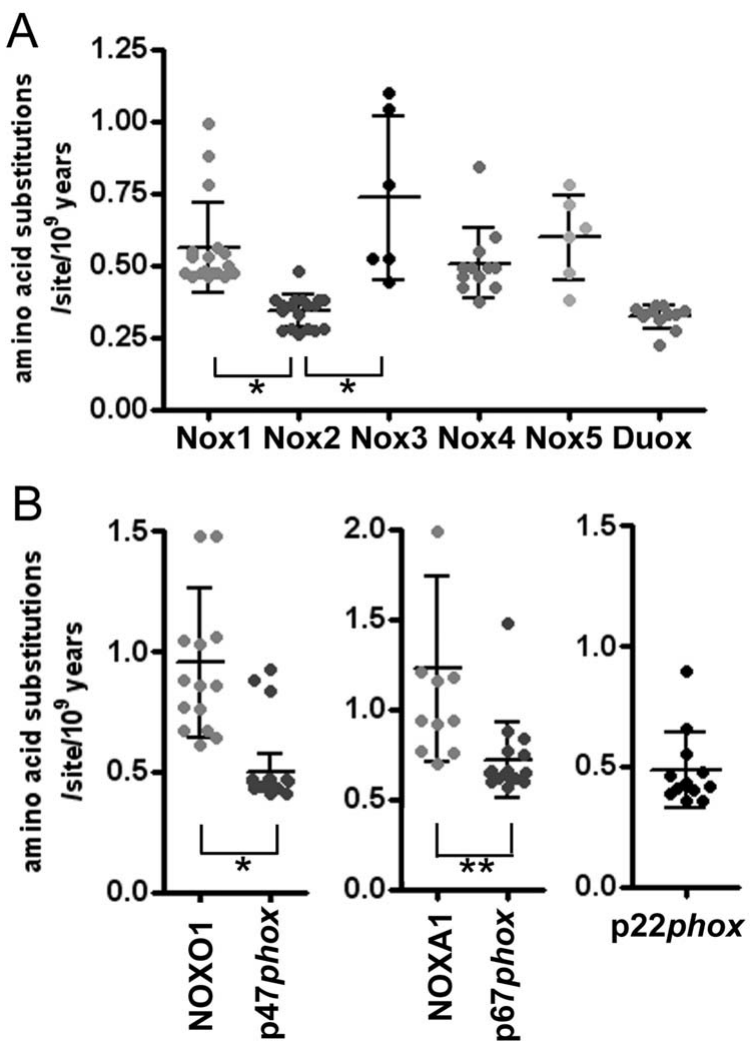
**Comparison of molecular clocks of Nox domains**.(*A*) Amino acid sequences were trimmed to the length of human Nox2 as Nox domains, and the numbers of amino acid substitutions per site relative to the human isolog were counted. Each data point represents amino acid substitution rates per site per 10^9 ^years for a given isolog (see Additional file 3). (*B*) Each data point represents amino acid substitution rates of regulatory subunits per site per 10^9 ^years. *Asterisks *indicate significance: *p *< 0.001 (*) or *p *< 0.002 (**).

To compare the rates of evolution among Nox subfamilies, we calculated substitution rates among the vertebrate orthologs. For Nox2 and Duox1/2, the rates, expressed as amino acid substitutions per site per 10^9 ^years, were notably lower (0.34 ± 0.013 and 0.32 ± 0.011, respectively), compared with other Noxes (Figure 5A). The regulator subunits p47*phox*, NOXO1, p67*phox*, NOXA1, and p22*phox *that are seen in human are also observed in other vertebrates (except for the absence of NOXO1 and NOXA1 orthologs in fugu *T. rubripes *and the absence of a NOXA1 ortholog in tetraodon *T. nigroviridis*, see Additional file 6). Evolutionary substitution rates of the Nox regulatory subunits are shown in Figure 5B. Like Nox2, the substitution rates of p22*phox *and the Nox2 regulators, p47*phox *and p67*phox*, are significantly lower than those of the Nox1 regulatory subunits, NOXO1 and NOXA1 (Figure 5B).

### Identification of critical amino acids common to all Nox/Duox proteins

**Figure 6:**
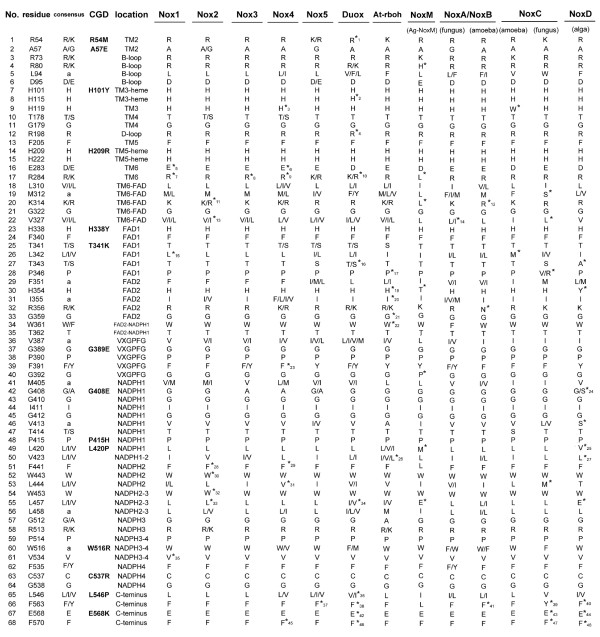
**Identification of amino acid residues conserved in all Nox/Duox proteins**. The single letter amino acid code is used, and consensus amino acid residues and locations are identified based on the alignment in Additional file 5. In the column labeled "consensus", "*a*" refers to hydrophobic side-chain amino acids. In the column labeled "location", "TM" refers to TM α-helix; and "TM3-heme" and "TM5-heme" refer to predicted heme-ligating histidine residues. NADPH1-2, NADPH2-3, and NADPH3-4 refer to amino acids that connect each canonical NADPH sub-region. Ag refers to *A. gambiae*. Point mutations in Nox2, which occur in variants of X-linked CGD, that correspond to the identified conserved amino acids are indicated. Exceptions to consensus residues are indicated by *asterisks *and are listed in Additional file 11.

**Figure 7:**
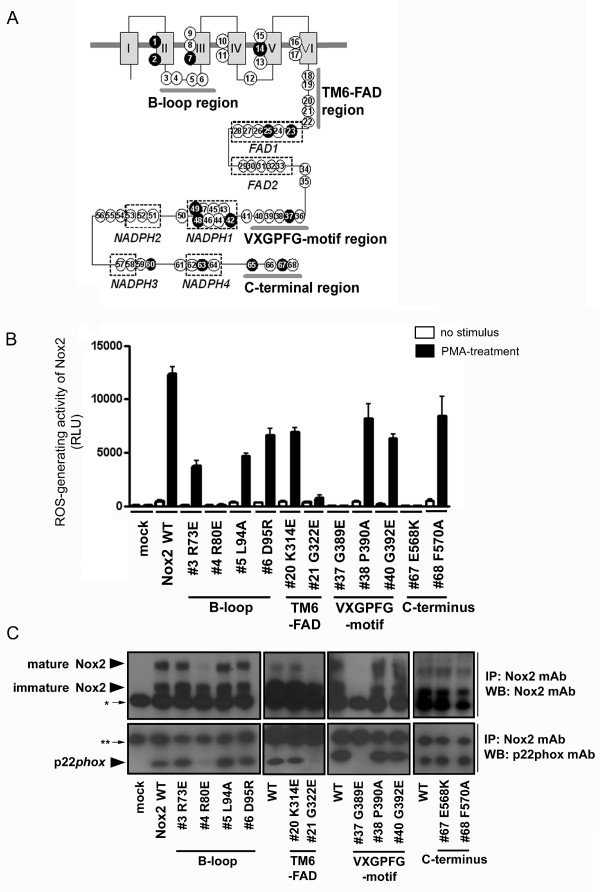
**Effects of mutations of conserved amino acids on Nox enzymatic activity and formation of the Nox2-p22*phox *complex**. (*A*) Conserved amino acids (circles) are indicated on a schematic of the Nox domain, and residue numbers corresponding to the human Nox2 protein sequence are keyed in Figure 6. Filled circles indicate known point mutations in individual variants of X-linked CGD. (*B*) HEK293 cells that constitutively express p22*phox *were co-transfected with wild type (WT) or the indicated mutations of Nox2 along with p47*phox*, p67*phox*, and Rac1(V12G) or with empty vector (mock). Each point mutation of human Nox2 is indicated by the single letter amino acid code. ROS production was measured as described, and the values are presented as mean ± SD (*n *= 4). These experiments have been repeated three times with similar results. (*C*) Nox2 and p22*phox *protein expression was probed by Western blotting (WB) with monoclonal antibodies 54.1 and 44.1, respectively. Proteins were immunoprecipitated (IP) with antibody 54.1 prior to SDS-PAGE. The *asterisks *indicate IgG heavy chain (* in upper panels) and light chain (** in lower panels). Nox2 protein is expressed as both unglycosylated (65 kDa, immature) and glycosylated (90–100 kDa, mature) forms. p22*phox *co-immunoprecipitated with Nox2 was seen at 22 kDa. These experiments have been repeated more than three times with similar results.

Key structural features of Noxes have been retained over hundreds of millions of years, implying key conserved residues from a common ancestor. Conserved amino acids were identified by aligning the 101 sequences of the Nox domain shown in SD2, but omitting hydrophobic amino acids in transmembrane regions. Sixty-eight residues were highly conserved in all Nox proteins (Figs. 6 and 7A). His-101, His-115, His-209, and His-222 [human (h)-Nox2 numbering] were present in all Noxes except for two Duox isologs (*X. tropicalis *Duox2 and *C. familliaris *Duox1), consistent with their proposed roles [57] as axial and distal ligands for the two heme irons. Sequences of the two divergent Duox isologs encode leucines at the residues corresponding to His-115 of h-Nox2 (Figure 6), suggesting that these Duox proteins are probably not able to produce ROS, or that they might have an unknown function conferred by the other canonical structures, such as FAD and NADPH binding sites. These findings also imply that a single Duox protein is sufficient to fulfill the biological role of the enzyme in these species. Specific amino acids were conserved in the two canonical FAD-binding subregions (FAD1-2) and in or near the four NADPH-binding subregions (NADPH1-4). Four additional conserved regions were noted: B-loop, TM6-FAD, VXGPFG-motif, and the extreme C-terminus (Figure 6 and filled circles of Figure 7A). We compared these positions to point mutations previously identified in Nox2 from variants of X-linked chronic granulomatous disease (CGD). CGD is a genetic immune disorder characterized by a functionally defective phagocyte NADPH oxidase, and the X-linked form is caused by mutations in the Nox2 gene. Of 23 known CGD point mutations [58-60], 14 are in amino acids conserved among all Noxes (Figs. 6 and 7A). These naturally occurring mutations in CGD confirm the importance of these residues to Nox function and stability. Among the CGD point mutants that are conserved among all Noxes, His-209-Arg, Gly-389-Glu, Leu-420-Pro and Trp-516-Arg are Nox2 protein-null CGD mutants (X91^0^), and result in the absence of Nox2 protein in the patient's neutrophils. The loss of Nox2 is thought to be due to destabilization of the protein structure and rapid protein degradation. His-101-Tyr and His-338-Tyr cause decreased Nox2-expression in CGD (X91^-^), suggesting that in addition to being functionally important in enzymatic activity or activation of Noxes, many of the 68 amino acids conserved among all Noxes are likely to be involved in maintaining or stabilizing the structure of the Nox domain. There were several exceptions among these otherwise highly conserved residues (indicated by *asterisks *in Figure 6), and these are listed in Additional file 11. These rare exceptions could be due to mis-sequencing of the genome or to tolerance of evolutionary changes in the species. To test functional importance of the B-loop, VXGPFG-motif, TM6-FAD region, and C-terminus, we generated 11 point mutations of human Nox2. Individual mutation of these conserved amino acids markedly inhibited and in some cases, completely abolished ROS production in a cell model system in which Nox2 and its regulatory subunits were also expressed (Figure 7B). Therefore, along with the canonical domains for cofactor binding, sequence comparisons identified additional regions that are important for Nox2-dependent ROS generation. Nox2 is expressed as a protein of 65 kDa ("immature Nox2" in Figure 7C), which is then glycosylated to generate a form with an apparent molecular size of 91 kDa ("mature Nox2" in Figure 7C). None of the mutants of Nox2, except for Gly-389, affected the expression of Nox2 protein (Figure 7C); however, Nox2 mutated at Arg-80 (B-loop) or Gly-322 (TM6-FAD region) failed to form a complex with p22*phox*, as determined by co-immunoprecipitation, and these mutations also failed to become glycosylated (Figure 7C). Protein of the Nox2 mutations in Gly-389 was not detected by Western blotting, which is likely due to de-stabilization of the protein or possibly to impaired recognition by the monoclonal antibody because the epitope of antibody overlaps Gly-389 [61].

### Variations in calcium-binding domain structures

**Figure 8:**
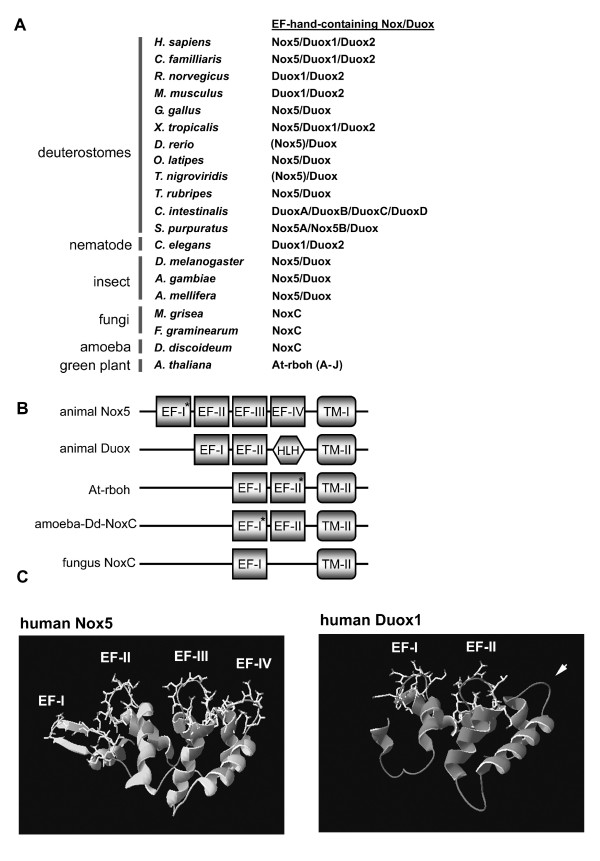
**Sequences of EF-hand motifs in Nox5, Duox, plant Nox, and NoxC**. (*A*) EF-hand containing Nox/Duox family members are listed and demonstrate the widespread occurrence of these Noxes. *Parentheses *represent the presence of Nox5 ortholog in the *T. nigroviridis *and *D. rerio *genomes, which clearly belongs in the Nox5 subgroup (#45 and #47 in Figure 2), but appears to lack the N-terminal EF-hand-containing domain as discussed in the text. (*B*) Based on alignments shown in Additional files 2 and 8, the arrangement of EF-hand motifs within the calcium-binding domain of calcium-regulated Noxes are shown schematically. EF-I to EF-VI in squares indicate each canonical EF-hand motif. TM-I and TM-II indicate the 1^st ^or 2^nd ^TM segments. *Asterisks *represent atypical EF-hand motifs that differ from consensus sequences at positions 1, 3, or 12, normally the most conserved positions [63]. *HLH*, refers to a non-canonical helix-loop-helix predicted structure. (*C*) A structural homology model of the EF-hand-containing domains of human Nox5 (upper panel) and human Duox1 (lower panel) using a comparative protein modeling method (SWISS-MODEL) and visualized with Deep View Swiss-PDB. The N-terminal region of Nox5 and human Duox1 was calculated using the structure of calcineurin B subunit isoform 1 as a fit template. The fit of the N-terminal region of Duox1 corresponding to the first EF-hand-like motif of Nox5 was not accurate enough to determine the molecular model. The *arrow *indicates the position of Duox1 corresponding to the 4^th ^EF-hand motif of Nox5 and models as a HLH structure that lacks canonical calcium binding amino acid residues. Side chains of canonical EF-hand motifs are indicated. Conserved sequences among EF-hand regions in Noxes and Duoxes are aligned and compared in Additional file 2.

The majority of Noxes in Figure 2 fall into calcium-regulated subgroups. Several calcium-binding motifs have evolved, including the EF-hand motif, which is ubiquitous in eukaryote and prokaryote genomes. The EF-hand motif was first described in the crystal structure of parvalbumin [62]. This motif has a characteristic helix-loop-helix (HLH) structure, consisting of approximately 30 residues, with the 12 residues situated in the loop contributing to the calcium binding [63]. By comparing the Nox/Duox sequences with the PROSITE database [64], 51 Nox/Duox sequences contained single or multiple EF-hands (see Additional file 2). They include animal Nox5 and Duox, plant Nox At-rboh, and NoxC of amoeba and fungi (Figure 8A). Nox5 had four EF-hand motifs (Figure 8A). The N-terminal motif was non-canonical [23] (see Additional file 2); however, this motif is also crucial for ROS-generating activity of Nox5, as a truncated mutation of the non-canonical EF-hand motif in human Nox5 lacked calcium-dependent ROS generation in *nox5*-transfected HEK293 cells (T. Kawahara and J. D. Lambeth, unpublished observation). Although there are rare exceptions to the consensus sequences in the C-terminal EF-hand motif sequences of Duox (indicated by asterisks in Additional file 2), Duox has two canonical EF-hands corresponding to the 2^nd ^and 3^rd ^EF-hands of Nox5 (Figure 8B). To add confidence to the alignment between EF-hand motif regions of Nox5 and Duox, a structural homology model of regions containing EF-hands of human Nox5 and human Duox1 was developed using the comparative protein modeling method (SWISS-MODEL [65]). Although the region between the 2^nd ^EF-hand and TM II domains of Duox lacks a classical EF-hand motif, this region of human Duox1 is predicted to form a HLH structure ("HLH" in Figure 8B and arrow head in the right panel of Figure 8C), similar to the predicted HLH structure comprising the 4^th ^EF-hand motif of human Nox5 ("EF-IV" in the left panel of Figure 8C). According to alignment of Duox sequences shown in Additional file 8, this additional "HLH" region of Duox seems to be widely conserved among animal Duox proteins, with the exception of chicken *G. gallus *and fugu *T. rubripes *orthologs. Thus, Duox and Nox5 possess structural homology in these regions, even though the total number of canonical EF-hand motifs is different. As shown in Figure 8B, the alignment also indicates that *A. thaliana *At-rboh and amoeba *D. discoideum *NoxC each possess two EF-hands that corresponded to the 3^rd ^and 4^th ^motifs of Nox5; whereas, the 2^nd ^EF-hand regions of all At-rboh proteins did not correspond to the EF-hand consensus sequence (see Additional file 2). Like At-rboh proteins, one of EF-hand motifs of amoeba NoxC did not follow the consensus sequence (indicated by asterisks in Figure 8). Nevertheless, these inconsistencies in the consensus sequence of EF-hands do not eliminate the possibility that such EF-hand-like motifs could bind to calcium ion, as a number of exceptions to this rule have been found [63,66]. A single canonical EF-hand is detected in two fungal NoxC (*M. grisea *and *F. graminearum*), which corresponds to the position of the 3^rd ^motif of Nox5 (see Additional file 2). Thus, binding of calcium to a single EF-hand may be sufficient in some Noxes to impart calcium regulatability. In contrast, calcium binding to Nox5 is likely to be cooperative, and might be expected to permit regulation over a narrower range of calcium concentrations.

### Phylogeny of Noxes

**Figure 9:**
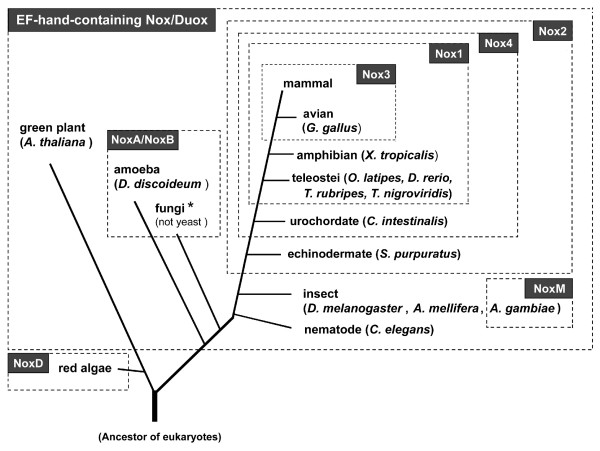
**Occurrence of Noxes in the phylogenetic tree**. A schematic phylogeny of organisms was created from genomic information [49, 53, 67, 91]. Branch lengths are not proportional to divergence. "Mammals" in this figure include *H. sapiens*, *C. familliaris*, *R. norvegicus*, and *M. musculus*. Among fungi, yeast (*S. pombe *and *S. cerevisiae*) lacked Noxes, although they did possess a protein with similar structural domains, Fre (see Additional file 9). The *asterisk *indicates an apparent lack of EF-hand-containing Nox/Duox in two fungi; *M. grisea, F. graminearum *possess an EF-hand-containing Nox, whereas *P. anserina *and *A. nidulans *do not.

The relationship between the phylogenic tree of organisms and the occurrence of Nox/Duox family genes (Figure 9) was determined by synteny and sequence information. Calcium-regulated, EF-hand-containing Noxes are not only abundant (Figure 8A), but also appeared very early during the evolution of eukaryotes. Nox3 appeared late in evolution, corresponding to the permanent transition of vertebrates from water to land. A primordial Nox2 appeared first in echinoderms, while Nox4 first appeared somewhat later in urochordates. Nox1 emerged relatively later in teleost fish. The mosquitoes *A. gambiae *and *A. aegypti *(but not fruit fly or ant) have NoxM gene (Figure 9) that is difficult to classify, but may belong in the NoxA/NoxB family, as discussed above (see Figure 2). Red macro-algae are eukaryotes and are thought to have branched earlier than plants from a common root [67]. The red algae *C. crispus *and *P. yezoensis *possess NoxD, which lacks an N-terminal EF-hand region (Figure 1B). Although these species do not appear to possess any regulatory subunit homologs [31], NoxD contains an additional 4 predicted transmembrane regions. The function of such additional transmembrane regions is unknown, and it is not yet clear whether this atypical Nox represents an ancestral prototype or a later adaptation.

## Discussion

This report provides the first extensive analysis of Nox sequences and synteny throughout evolution and provides a conceptual framework for future structure/enzymatic function studies and for understanding the diversity of biological functions of these enzymes. Molecular taxonomy (Figure 2) revealed seven Nox/Duox subfamilies rather than the three that were previously identified based on the presence or absence of calcium-binding and peroxidase domains [2]. Significantly, Noxes are not present in prokaryotes. One can speculate that while defense enzymes, such as superoxide dismutase, evolved very early to protect aerobic organisms to protect against accidentally-generated ROS, later organisms subsequently developed the capacity to generate ROS in a regulated, "deliberate" manner, with specific regulatory subunits co-evolving with specific Noxes. The earliest Nox2 ortholog seems to have appeared in the sea urchin *S. purpuratus*. A number of investigators have suggested that sea urchin has phagocytic cells that express an ortholog of the complement component C3 and can phagocytose against invading microbes [68-70]. Although it is unclear whether these cells produce ROS to kill microbes, the taxonomy shown in Figure 2 implies that the sea urchin expresses a Nox2 ortholog that might play a role in the innate immune response.

The synteny of each Nox/Duox member raises new questions about Nox/Duox evolution. For example, Nox3 in mouse inner ear is essential for formation of otoconia, mineralized structures that participate in the vestibular system in perception of gravity [44]. Fish and amphibians also have otoconia (called otoliths in fish) but do not express Nox3 (Figure 3). This may implicate another Nox, for example Nox1 ortholog, in otoconia formation prior to Nox3 appearance in land vertebrates, or it may point to a unique function of Nox3 in land vertebrates. Kiss *et al*. have suggested that lactoperoxidase (LPO) functions in peroxidation of the lipid envelope of globular substance in the inner ear together with Nox3 [18]. Interestingly, molecular taxonomy of animal heme peroxidases demonstrates that LPO orthologs emerged in birds and mammals, but not in fish (T. Kawahara and J. D. Lambeth, unpublished observation). It implies that Noxes and their physiological partners evolved simultaneously, resulting in gaining a new function. Mosquito has a unique Nox gene, NoxM. Although a physiological function of NoxM is completely unknown, a unique appearance of NoxM gene in the species imply a possible relationship between NoxM and sucking of blood or a playing role as a principal vector of the pathogen. While the function of Nox5 is not yet understood, its loss in rodents suggests that another Nox may compensate in these species, implying a certain degree of plasticity of Nox isoform function. Alternatively, Nox5 may not perform an essential function, at least in short-lived species.

It is of interest to compare residue substitution rates among Nox isologs, since all members possess fundamentally similar structures in their flavocytochrome domains, including a high degree of conservation of binding sites for prosthetic groups. Substitution rates vary among different proteins, such as EGF (~ 2.5), NGF (~ 1.0), lactate dehydrogenase (~ 0.5), cytochrome *c *(~ 0.3), and histone H3 (~ 0.014) [71]. The substitution rates of Nox/Duox subfamilies ranged from 0.3 ~ 0.7 (Figure 5); whereas, those of p22*phox*, organizer proteins (p47*phox *and NOXO1), and activator proteins (p67*phox *and NOXA1) were 0.5 ~ 1.2. The substitution rates of Nox2 and its regulatory subunits, p47*phox *and p67*phox*, were relatively low, implying evolutionary changes in these proteins are more poorly tolerated. Such a result may be explained by the importance of the biological function of Nox2 in host defense and by the stringent regulation of this enzyme system to prevent inappropriate activation leading tissue damage [5,58,59]. In addition, the Nox2 system requires multiple protein interactions among catalytic and regulatory subunits with upstream regulatory subunits and lipids, and these undoubtedly impose strict limitations on the number of tolerated mutations. Although incompletely understood, substitution data point to the critical nature of Duox functions, since changes in the Duox sequence are also poorly tolerated over evolutionary time (Figure 5). Duoxes are implicated in thyroid hormone biosynthesis [43] and innate immunity in lung [72], and are distributed in a variety of other tissues where they perform unknown functions. Duox is also implicated in fertilization in sea urchin, where H_2_O_2_-supported cross-linking of fertilization envelope proteins prevents polyspermy [73].

In contrast to other eukaryotes, yeast (*S. pombe *and *S. cerevisiae*) did not possess Noxes. Yeast ferric reductase (Fre) has a domain structure similar to Noxes but participates in iron uptake rather than oxygen reduction [74]. Alignment between human Nox2 and Fre proteins demonstrates that the sequence of Fre proteins is very different from the Noxes except for the VXGPFG-motif and NADPH-binding site residues (see Additional file 9). This suggests that this distant homolog has evolved in yeast to carry out an entirely different function, and it is debatable whether it should even be classified with the Nox family.

In addition to binding residues for prosthetic groups, the present study has identified four additional regions (B-loop, TM6-FAD, VXGPFG-motif, and C-terminus) as critical for function in all Noxes. The specific functions of these regions are not yet fully understood; however, mutational analysis demonstrates their importance (Figure 7B). The B-loop (Arg-80) and TM6-FAD (Gly-322) regions of Nox2 appear to participate directly or indirectly in binding to p22*phox *(Figure 7C), since their mutation prevented co-immunoprecipitation of Nox2 and p22*phox*. In addition, these mutations prevented glycosylation of Nox2 to form the mature 91 kDa form of the protein, supporting the concept that association with p22*phox *is a necessary pre-requisite for glycosylation [75]. Nevertheless, these two amino acid residues are also conserved in Noxes that do not require p22*phox *(e.g., human Nox5). Thus, these residues might mediate another important interaction in Nox5 that is analogous to that with p22*phox*, and ongoing studies are investigating the roles of these residues in Nox5 function. The presence of non-canonical, but highly conserved residues and regions that are shown in Figure 7A suggests that Noxes might have an unknown common feature relevant to the mechanism of activation or a common biological function. Moreover, these conserved regions may also provide a key to identifying a novel common molecule that interacts and co-operates with all Nox/Duox proteins.

## Conclusion

In summary, we report herein an exhaustive analysis of Nox/Duox protein family. The present studies provide a new molecular classification system in which Nox and Duox proteins are organized into seven distinct subfamilies. These studies also identify Nox3 as the most recently emerged Nox. Calcium-regulated, EF-hand-containing Noxes appeared very early during the evolution of eukaryotes. Two mosquitoes possess a unique Nox gene, NoxM, but not fruit fly or ant. Consistent with the physiological importance of Nox2 in innate immunity and Duox for hormone synthesis and host defense, these two Nox proteins are more stringently conserved of all Noxes. By comparison of amino acid sequences, 68 residues were identified as highly conserved among all Nox/Duox orthologs, and the B-loop, TM6-FAD, VXGPFG-motif, and extreme C-terminal regions were identified as important for Nox activity. Thus, this report provides a conceptual basis for understanding the evolutionary history of Noxes and Duoxes and provides key structural information relevant to the activation mechanisms of modern Nox/Duox proteins.

## Methods

### Gene identification

Nox/Duox family gene sequences were assembled from the following species: *Homo sapiens *(*H. sapiens*, human), *C. familliaris *(dog), *R. norvegicus *(rat), *M. musculus *(mouse), *B. taurus *(cow to search for Nox5 ortholog), *M. domestica*, (opossum to search for Nox5 ortholog), *D. novemcinctus *(armadillo to search for Nox5 ortholog)*, O. cuniculus *(rabbit to search for Nox5 ortholog)*, G. gallus *(chicken), *X. tropicalis *(frog), *D. rerio *(zebrafish), *T. rubripes *(fugu), *T. nigroviridis *(tetraodon), *Oryzias latipes *(*O. latipes*, medaka), *C. intestinalis*, *S. purpuratus *(sea urchin), *C. elegans*, *D. melanogaster *(fruit fly), *A. mellifera*, (honeybee), *A. gambiae *(malaria mosquito), *A. aegypti *(yellow fever mosquito), *A. thaliana *as a green plant, *D. discoideum *(amoeba), *Podospora anserina *(*P. anserina*, fungus-*Pa*), *A. nidulans *(fungus-*An*), *M. grisea *(fungus-*Mg*), *F. graminearum *(fungus-*Fg*), *C. crispus *(alga-*Cc*), and *P. yezoensis *(alga-*Py*).

In addition to gene accession numbers that have been published in papers cited above [26,28,30,31,33,39,41,42], existing homologues/orthologs of Nox/Duox were searched using NCBI HomoloGene [76]. Human Nox1-Nox5, Duox1, and Duox2; dog Nox1, Nox4, Duox1, and Duox2; mouse Nox1-Nox4, Duox1, and Duox2; rat Nox1-Nox4, Duox1, and Duox2; chicken Nox3; and fruit fly Nox5 and Duox amino acid sequences were obtained from GenBank, and accession numbers are shown in Additional file 4. BLASTP searches were performed for amino acid sequences predicted computationally from genomes of *C. familliaris*, *R. norvegicus*, *M. musculus*, *D. novemcinctus, O. cuniculus, G. gallus*, *X. tropicalis*, *D. rerio*, *T. rubripes*, *T. nigroviridis*, *O. latipes*, *C. intestinalis *and *C. elegans *[55]. Sequences that had >50% identity to the sequence of the closest template were selected.

To identify the Nox/Duox orthologs of sea urchin *S. purpuratus *and insects (*D. melanogaster*, *A. mellifera, A. gambiae*), BLASTP searches were performed using the NCBI sea urchin protein database [77] and the NCBI Eukaryotic Genome Database [78], respectively. Fungi Genome BLAST and NCBI BLASTP [51] and DictyBase BLASTP [79] were used to search for Nox homologs in fungi (*P. anserina*, *A. nidulans*, *M. grisea*, and *F. graminearum*) and amoeba (*D. discoideum*), respectively. The presence of 10 genes encoding *A. thaliana *Nox homologs has been predicted [42], which was confirmed by searching the TIGR *A. thaliana *Protein Database [80]. Sequences and accession numbers of assembled Nox orthologs are listed in Additional file 4, and information on additional database searching is described in Additional file 12. Assembled sequences, including newly defined or previously mis-annotated sequences, were annotated based on molecular taxonomy analysis of the Nox domain (Figure 2), and these sequences were deposited as third party annotation (TPA) sequences in GenBank/EMBL/DDBJ database (accession No. BR000261–BR000304). All amino acid sequences analyzed in this study are shown in Additional file 4 (Nox/Duox proteins) and Additional file 9 (Fre proteins).

To estimate evolutionary substitution rates, we screened orthologs of the regulatory subunits, p22*phox*, p47*phox*, NOXO1, p67*phox*, and NOXA1 in vertebrates (*H. sapiens*, *C. familliaris*, *R. norvegicus*, *M. musculus*, *G. gallus, X. tropicalis*, *D. rerio*, *T. rubripes*, *T. nigroviridis*) using servers described above. In this study, partial Nox/Duox or regulatory subunit genes, which lacked either a presumed start codon or a stop codon, were assumed to be intact if they showed >50% identity to the closest homolog. All amino acid sequences of the regulatory subunits analyzed in this study are shown in Additional file 6.

### Phylogenic analysis and synteny

Multiple sequence alignment and phylogenetic analyses were carried out with ClustalW [81,82]. Phylogenetic trees were reconstructed by the neighbor-joining method [83,84] implemented with Kimura 2-parameter distances [85]. Each node of the phylogenetic tree was evaluated by 1,000 bootstrap replications [86]. Amino acid sequences of Nox and Duox were trimmed and aligned to the length of human Nox2 (see Additional file 5). The additional 4 predicted transmembrane regions of the algal Nox orthologs [residues 372–676 of *C. crispus *NoxD GenBank™ No. AAZ73480.1 and residues 363–778 of *P. yezoensis *NoxD GenBank™ No. ABA18724.1] were also trimmed prior to phylogenetic analyses. To elucidate synteny, we used the AlignSliceView program [87], which provides genome annotation according to DNA-DNA similarity and selected conserved genes among the genomes of vertebrates [88] as markers. These marker genes are defined in Additional file 10.

### Alignment of EF-hand regions

According to the motif search server PROSITE [64], single and multiple EF-hand motifs were found in the extreme N-terminal region of Nox5, plant Nox, NoxC, and a loop region between 1^st ^and 2^nd ^TM domains of Duox protein. After trimming the N-terminal extension from TM1 of Nox5, plant Nox, NoxC (e.g., residues 1–235 of human Nox5α) and the loop between TM1 and TM2 domain of Duox (e.g., residues 767–1074 of human Duox1), alignment was performed. The alignment is shown in Additional file 8, and amino acid residues corresponding to the EF-hand motifs are shown in Additional file 2.

### Homology modeling

SWISS-MODEL [65,89] was utilized to predict protein structure of the EF-hand domain of human Nox5 (residues 1–235 of human Nox5α, GenBank™ No. AF353088) and human Duox1 (residues 767–1074 of human Duox1, GenBank™ No. NM_017434). Modeling of the N-terminal region of Nox5 was carried out based on the structures of calcineurin B subunit isoform 1 (PDB accession No. 1m63F), calcineurin B-like protein 2 (PDB No. 1uhnA), and calcineurin B-like protein 4 (PDB No. 1v1fA). Homology modeling of human Duox1 was performed using calcineurin B subunit isoform 1 (PDB No. 1m63B, 1m63F, and 1auiB). Structures were visualized using the program DeepView [90].

### Estimates of substitution rates of vertebrate Nox/Duox and regulators

Using the estimated divergence time of species and number of identical amino acid residues [91], we calculate a substitution rate for each amino acid site per 10^9 ^years. Detailed methods to calculate rates and amino acid residues are described in Additional files 12 and 3, respectively.

### Generation of point mutations of human Nox2

Point mutations of Nox2 were introduced by site-directed mutagenesis, as described previously [20], and detailed procedures are described in Additional file 12.

### Measurement of ROS production by Nox2-transfected cells

Human embryonic kidney (HEK) 293 cells were grown for 24 hrs in 6-well plates and allowed to reach 50% confluency in 2 ml of culture medium. HEK 293 cells, which endogenously express p22*phox*, were co-transfected with vectors encoding Nox2, p47*phox*, p67*phox*, and an active form of the small GTPase Rac1 [Rac1(V12G)] using FuGENE 6 (Roche Applied Science, Indianapolis, IN). ROS was measured using luminol chemiluminescence, as previously described [20], and detailed procedures are described in Additional file 12.

### Immunoprecipitation of Nox2 and p22*phox *proteins

Transfected cells were lysed for 20 min on ice with lysis buffer containing 50 mM Tris/HCl (pH 7.4), 150 mM NaCl, 1% Triton X-100, 0.25% deoxycholate, 1 mM NaVO_4_, 10 mM NaF, protease inhibitor mixture (Complete™; Roche Applied Science, Indianapolis, IN), 1 mM phenylmethylsulfonyl fluoride, and 100 μM diisopropyl fluorophosphate. The lysates were centrifuged for 5 min at 10,000 × *g*, and protein G-sepharose (Sigma, St. Louis, MO) was added to the supernatants to remove non-specific binding proteins. After centrifugation, the supernatants were mixed with anti-Nox2 monoclonal antibody 54.1 [92]. Protein G-sepharose was added to each mixture, and the precipitated protein from 5 × 10^6 ^cell equivalents was subjected to SDS-PAGE, followed by Western blot analysis.

### Western blot analysis

To assess Nox2 expression and ability to bind p22*phox*, Western blot analysis was performed using monoclonal antibodies, 54.1 and 44.1 against human Nox2 and p22*phox*, respectively [92,93]. Detailed procedures are described in Additional file 12.

### Statistical analysis

GraphPad Prism (GraphPad software Inc.) was used for *t*-test statistical analysis to show significant differences of substitution rates.

## List of abbreviations used

Nox, NADPH oxidase: Duox, dual oxidase; ROS, reactive oxygen species; rboh, respiratory burst oxidase homologue; TM, transmembrane; CGD, Chronic Granulomatous Disease; HLH, helix-loop-helix; Myr, 10^6 ^years; PMA, phorbol 12-myristate 13-acetate; WB, Western blotting; IP, immunoprecipitation; mAb, monoclonal antibody.

## Authors' contributions

The majority of this work here described was planned and carried out by TK in collaboration with MTQ. JDL critically revised the manuscript for important intellectual content and data analysis. All authors read and approved the final manuscript.

## Supplementary Material

Additional File 4 **Amino acid sequences of Nox and Duox proteins**. Amino acid sequences Nox and Duox proteins of *H. sapiens*, *B. taurus*, *C. familliaris*, *R. norvegicus*, *M. musculus*, *G. gallus*, *M. domestica*, *D. novemcinctus*, *O. cuniculus*, *X. tropicalis*, *D. rerio*, *T. rubripes*, *T. nigroviridis*, *O. latipes, C. intestinalis*, *S. purpuratus*, *D. melanogaster*, *A. gambiae*, *A. aegypti*, *A. mellifera*, *C. elegans*, *A. thaliana*, *D. discoideum*, *P. anserine*, *A. nidulans*, *M. grisea*, *F. graminearum*, *C. crispus *and *P. yezoensis *are provided. Click here for file

Additional File 1 **Supplemental Figure S1**. The figure provided shows loop and segment sizes joining transmembrane regions and canonical NADPH-oxidase domains Click here for file

Additional File 6 **Amino acid sequences of vertebrate Nox regulatory subunits; p47 *phox*, NOXO1, p67 *phox*, NOXA1, p22 *phox***. Lists of amino acid sequences Nox regulatory subunits of *H. sapiens*, *C. familliaris*, *R. norvegicus*, *M. musculus*, *G. gallus*, *X. tropicalis*, *D. rerio*, *T. rubripes*, *T. nigroviridis *are provided. Click here for file

Additional File 11 **Exceptions of amino acid residues conserved in all Nox/Duox proteins**. The data provided show exceptions of the 68 residues conserved in all Nox/Duox proteins that are indicated by *asterisks *in Figure 6. Click here for file

Additional File 2 **Supplemental Figure S2**. The figure provided shows sequences of EF-hand motifs in Nox5, Duox, plant Nox, and NoxC Click here for file

Additional File 8 **Alignment of EF-hand regions of plant Nox, NoxC, Nox5, and Duox**. Alignment of EF-hand region of plant Nox, NoxC, Nox5, and Duox are provided. EF-hand motif regions and non-EF-hand "helix-loop-helix (HLH)" structure regions are shown. Click here for file

Additional File 9 **Amino acid sequences of ferric reductase (FRE) proteins of fungi and alignment of FRE protein and human Nox2 protein sequences**. Amino acid sequences of FRE of *A. nidulans*, *Saccharomyces cerevisiae*, *Schizosaccharomyces pombe *and alignment of FRE and human Nox2 proteins are provided. Click here for file

Additional File 12 **Additional methods information**. Methods information about gene identification, estimates of substitution rates, generation of point mutations, and Western blot analysis are provided. Click here for file

Additional File 5 **Alignment of Nox-domains of Nox and Duox proteins**. Amino acid sequences of Nox and Duox orthologs were trimmed to the length corresponding to human Nox2 and aligned. Sixty-eight amino acid residues conserved among all Nox and Duox proteins (shown in Figure 6) are indicated by *asterisks *below the alignments. Click here for file

Additional File 10 **Abbreviation of gene names in Figure **3. Names of the marker genes used in Figures 3 are provided. Click here for file

Additional File 3 **Supplemental Figure S3**. The figure provided shows substitution rates of vertebrate Nox/Duox and Nox regulatory proteins Click here for file

Additional File 7 **Alignment of vertebrate p47 *phox*, NOXO1, p67*phox*, NOXA1, and p22*phox *proteins to estimate substitution rates**. Amino acid sequences of p47*phox*, NOXO1, p67*phox*, NOXA1 and p22*phox *were aligned. These alignments were used to estimate evolutionary substitution rates in vertebrate orthologs of Nox regulatory subunits. Click here for file
