# Supplementary material for: Molecular evolution of the reactive oxygen-generating NADPH oxidase (Nox/Duox) family of enzymes
Source: BMC Evol Biol. 2007 Jul 6;7:109. doi: 10.1186/1471-2148-7-109 (PMC1940245; doi:10.1186/1471-2148-7-109)
Supplement: Additional File 4 — Amino acid sequences of Nox and Duox proteins. Amino acid sequences Nox and Duox proteins of H. sapiens, B. taurus, C. familliaris, R. norvegicus, M. musculus, G. gallus, M. domestica, D. novemcinctus, O. cuniculus, X. tropicalis, D. rerio, T. rubripes, T. nigroviridis, O. latipes, C. intestinalis, S. purpuratus, D. melanogaster, A. gambiae, A. aegypti, A. mellifera, C. elegans, A. thaliana, D. discoideum, P. anserine, A. nidulans, M. grisea, F. graminearum, C. crispus and P. yezoensis are provided. [file 1471-2148-7-109-S4.pdf]

Additional file 4

Amino acid sequences of Nox and Duox proteins

To describe sequences from different species, we used the following naming: human-Hs (as *H. sapiens*), cow-Bt (*B. taurus*), dog-Cf (*C. familliaris*), rat-Rn (*R. norvegicus*), mouse-Mm (*M. musculus*), chicken-Gg (*G. gallus*), opossum-Md (*M. domestica*), armadillo-Dn (*D. novemcinctus*), rabbit-Oc (*O. cuniculus*), frog-Xt (*X. tropicalis*), zebrafish-Dr (*D. rerio*), fugu-Tr (*T. rubripes*), tetraodon-Tn (*T. nigroviridis*), medaka-Ol (*O. latipes*), ascidian-Ci (*C. intestinalis*), sea urchin-Sp (*S. purpuratus*), fruit fly-Dm (*D. melanogaster*), mosquito-Ag (*A. gambiae*), mosquito-Ae (*A. aegypti*), honeybee-Am (*A. mellifera*), nematode-Ce (*C. elegans*), At (*A. thaliana*), amoeba-Dd (*D. discoideum*), fungus-Pa (*P. anserina*), fungus-An (*A. nidulans*), fungus-Mg (*M. grisea*), fungus-Fg (*F. graminearum*), alga-*Cc* (*C. crispus*) and alga-*Py* (*P. yezoensis*). Sequences are available from the indicated servers: http://www.ncbi.nlm.nih.gov/ (GenBank^{TM} numbering genes), http://www.ddbj.nig.ac.jp/searches-e.html. (DDBJ number gene) or http://www.ensembl.org. (ensembl numbering genes), and http://flybase.net/ (FlyBase accession number sequences). Genome locations of vertebrate Nox genes are shown in Figure 3, and those of *C. intestinalis* genes are described herein, following accession numbers. The insertion containing 4 additional predicted transmembrane regions

(TM7-TM10) of the algal Nox orthologs that were trimmed prior to phylogenetic analyses is underlined.

> human-Hs-Nox1: GenBank™ accession No. NM_007052
MGNWVVNHWFSVLFLVVWLGLNVFLFVDAFLKYEKADKYYYTRKILGSTLACARASALCLNFNST
LILLPVCRNLLSFLRGTCSFCSRTLRKQLDHNLTFHKLVAYMICLHTAIHIIAHLFNFDCYSRSRQAT
DGSLASILSSLSHDEKKGGSWLNPIQSRNTTVEYVTFTSIAGLTGVIMTIALILMVTSATEFIRRSYFE
VFWYTHHLFIFYILGLGIHGIGGIVRGQTEESMNESHPRKCAESFEMWDDRDSHCRRPKFEGHPP
ESWKWILAPVILYICERILRFYRSQQKVVITKVVMHPSKVLELQMNKRGFSMEVGQYIFVNCPSISL
LEWHPFTLTSAPEEDFFSIHIRAAGDWTENLIRAFEQQYSPIPRIEVDGPFGTASEDVFQYEVAVLVG
AGIGVTPFASILKSIWYKFQCADHNLKTKKIYFYWICRETGAFSWFNNLLTSLEQEMEELGKVGFL
NYRLFLTGWDSNIVGHAALNFDKATDIVTGLKQKTSFGRPMWDNEFSTIATSHPKSVVGVFLCGPR
TLAKSLRKCCHRYSSLDPRKVQFYFNKENF
> mouse-Mm-Nox1: GenBank™ No. NM_172203
MGNWLVNHWLSVLFLVSWLGLNIFLFVYAFLNYEKSDKYYYTREILGTALALARASALCLNFNSM
MILIPVCRNLLSFLRGTCSFCNRTLRKPLDHNLTFHKLVAYMICIFTVIHIIAHLFNFERYRRSQQAM
DGSLASVLSSLSHPEKEDSWLNPIQSPNMTVMYAAFTSIAGLTGVIATVALVLMVTSAMEFIRRNYF
ELFWYTHHLFIVYIICLGIHGLGGIVRGQTEESLGESHPHNCSHSFHEWDDHKGSCRHPHFAGHPP
ESWKWILAPIAFYIFERILRFYRSQQKVVITKVVMHPSNVLELQMRKRGFSMEVGQYIFVNCPSISF
LEWHPFTLTSAPEEEFFSVHIRAAGDWTRNLIRTFEQQHSPMPRIEVDGPFGTVSEDVFQYEVAVL
VGAGIGVTPFASILKSIWYKFQRADNKLKTQKIYFYWICRETGAFAWFNNLLNSLEQEMEELGKMD
FLNYRLFLTGWDSNIAGHAALNFDRATDILTGLKQKTSFGRPMWDNEFSRIATAHPKSAVGVFLCG
PRTLAKSLRKRCQRYSSLDPRKVQFYFNKETF
> rat-Rn-Nox1: GenBank™ No. NM_053683
MGNWLVNHWLSVLFLVSWLGLNIFLFVYVFLNYEKSDKYYYTREILGTALALARASALCLNFNSM
VILIPVCRNLLSFLRGTCSFCNHTLRKPLDHNLTFHKLVAYMICIFTAIHIIAHLFNFERYSRSQQAM
DGSLASVLSSLFHPEKEDSWLNPIQSPNVTVMYAAFTSIAGLTGVVATVALVLMVTSAMEFIRRNYF
ELFWYTHHLFIIYIICLGIHGLGGIVRGQTEESMSESHPRNCSYSFHEWDKYERSCRSPHFVGQPPE
SWKWILAPIAFYIFERILRFYRSRQKVVITKVVMHPCKVLELQMRKRGFTMGIGQYIFVNCPSISFL
EWHPFTLTSAPEEEFFSIHIRAAGDWTENLIRTFEQQHSPMPRIEVDGPFGTVSEDVFQYEVAVLVG
AGIGVTPFASFLKSIWYKFQRAHNKLKTQKIYFYWICRETGAFAWFNNLLNSLEQEMDELGKPDFL
NYRLFLTGWDSNIAGHAALNFDRATDVLTGLKQKTSFGRPMWDNEFSRIATAHPKSVVGVFLCGP
PTLAKSLRKCCRRYSSLDPRKVQFYFNKETF
> dog-Cf-Nox1: GenBank™ No. XM_549136

MGNWVVNHWFSVLFLATWLGLNVFLFVHAFLSYEKADKYYYTREILGSTLAWARASARCLNFNS
MLILLPVCRNLLSFLRGTCSFCRRTLRKQLDHNLTFHKLVAYMICLHTAIHIIAHLFNFERYSRSRQA
TDGSLASILSTLSHQGKEEDSWLNPMQSPNMTVEYVTFTSIAGLTGVIITIALVLMVTSAMEFIRRSY
FEVFWYTHHIFIIYFIGLGIHGIGGIVRGQTEESLNESHPHRCAESFKQWDDHDSHCKHPRFEGLPA
ESWKWILAPGVLYILERILRFYRSQQKVVITKVVMHPSKVLELQMIKRGFSMEVGQYIFVNCPSVSY
LEWHPFTLTSAPEEDFFSVHIRAVGDWTENLIRAFEQQCSPIPRIEVDGPFGTVSEDVFQYEVVVLV
GAGIGVTPFASILKSIWYKFRHEDHNLKTQTIYFYWICRETGAFAWFNDLLASLECEMEELGKVDF
LNYRLFLTGWDSNIASHATLNFDKATDILTGLKQKTSFGRPMWDNEFSTIANAHPRSVVGVFLCGP
QTLAKSLSKCCCQYSSLDPRKVQFYFNKENF

> chicken-Gg-Nox1: DDBJ™ No. BR000265

MGNWLVNHWFSAAVLAAWLGINIFLFTYYFLFFDRDERYFYTRAILGSALAWARASAKCLNFNSML
ILLPVCRNLLSFLRGSFSCCRRTLRKQLDHNLTFHKLVAYALALLTAVHTIAHLFNLERYNHSQQAN
DGSLHAVLSKMHLQDSNKWLNPIHSNQTTVEYVAFTTIPGLTGVIITLALILMVTSSTEFIRRNYFEV
FWYTHHLFIIYFIGLVIHGVAGLVRGQTEESMKEVHPQSCAEFLVNKSKECRHQCCKEPEFGSIPAE
VGMGVGLQESRLLHTPGCCXSSHTGTELPASQVVMHPAKVLELQMQKKGFRMEVGQYIFVNCPAV
SLLEWHPFTLTSAPEEDFFSIHIRAAGDWTEHIIDTFQQQKLEMPRIKVDGPFGTASEDVFLYEVAM
LVGAGIGVTPFASILKSIWYRFQQNDQTLKTKKIYFYWLCRDTGAFTWFNDLLASLEQKMAESGKA
DFLTYRLFLTGWDTSIANNAALHFDTVTDTVTGLRQKTIFGRPRWDTEFSAVATAHPRSVVGVFLC
GPEALAKVLRRSCHQHSSLDPRKVKFYFNKENF

> frog-Xt-Nox1: Ensembl No. ENSXETP00000021243

MGNWIANNWFSVVVLATWLGLNIFIFINFFMIFEKGDSYSYTRELLGSALAWARGSAACLNFNCLL
ILLPVCRNLLSFLRGTCTCVQRSMRKQLDNNLAFHKLVGYTIALMTAIHTIAHLFNVERYCDAAQK
KTDTLPGELSSIGEDGTWLNPVRSPTVTPPYFAFTTIAGLTGVVITLALILMITSSTEFIRRCYFEVFW
YTHHLFVIFFIGLVFHGAGRIVRGQTSDSMETNNYEKCHNSFTQWQNSKSSNRDDDDHHNDKCT
VPAFQGNEPGVRYKLKMYFETRQKRKTHAIKPNHTTMGHPSKVLEIQMQKRGFKMEVGQYIFINC
PSVSALEWHPFTLTSAPEEDCFSVHIRSAGDWTDNLIKVFQEQAENPPRLEVDGPFGTASEDVFQY
EVSMLVGAGIGVTPFASILKSIWYKFQRDDQRLKTKKIYFYWICRETGSFAWFADLLRSLEQEMICS
GKDGFLNYRLFLTSWDSKIAGHVVIDFDHATDTVTGLRQKTSYGRPIWENEFSKVAEWHPKSTVG
VFLCGPQALGKTLKQCCHQYSSLDPRKVQFYFNKENF

> tetraodon-Tn-Nox1: DDBJ™ No. BR000267

MGNWIVNHGLTSFILVVWMGINIFLFVWFYLFYDLGDQFFYTRHLLGSALAWARAPAAVLNFNCM
LILLPVCRNLLSLLRGSFVCCGRSMRKQLDKNLSFHKLVAYMIALMTAVHTVAHLLNVEWYNNSRQ
GVYDELSTALSKLDDANGTTYLNPIRITDLIPTYFAFTTIAGLTGVIITLSLILIITSSMEVIRRSYFEVF
WYTHHLFIIFFAGLVFHGAGRIVRSQQTTNPPHNTSYCKDHPDDWGHIPECPIPQFAGGFPQTWM
WVIAPMFLYVCERLIRFVRYMQTVRYRRIVMRPSKVLELQLVKSGFKMEVGQYVFLNCPAISQLEW
HPFTMTSAPEEDFFSVHIRSAGDWTDKLIDIMQKLPEGAQGPKMGVDGPFGTASEDVFDYEVSML

VGAGIGVTPFASILKSIWYKFKESNPKLRTRKIYFYWLCRETHAFEWFADLLQVLEKEMDERGMG
DFLTYKLYLTKWDQSHADHIMVHSDQDIDVVTGLRQKTYYGRPAWDKEFEQVRKENPTSVVGTFL
CGPEALAEVLEKKCVKYSDVDPRKTKFYFNKENF

> zebrafish-Dr-Nox1: DDBJ™ No. BR000266

MGNWIINHGLSAFIVVVWMAINIALFVHFYLFYDQGERFEYTRELLGSALAWARAPAAVLNFNCML
ILLPVCRNLLSLLRGSFVCCGRTVRKQLDKNLTFHKLVAYMIALMTAVHTIAHLFNAERYSNSLEGE
DGDLAFELSLLQDSSELNTTYLNPFPSNSTTPMIFVFTSIAGLTGVVITLALILMITSSMEVIRRSYFE
VFWYTHHLFIVFFAGLVFHGAGRVVRGQVTTDPPHNNSFCEDQPDNWGKIPECPIPQFAGGSPQT
WMYVIGPMIIYICERLLRFIRYMQPVTYRKIVIRPSKVLELQLVKPGFSMDVGQYVFLNCPAISQLEW
HPFTLTSAPEEDFFSVHIRSVGDWTEKLLKMVENLPEGGQGPKYVLLWIFTITSRMGVDGPFGTAS
EDVFHYEVSMLVGAGIGVTPFASILKSIWYKFKDSDPKLRTKRIYFYWLCRETHAFEWFADLLQVL
EREMEERGMRDFLTYKLYLTGWDQSHADHAMVHFDKDTDIITGLKQKTHYGRPNWDKEFEQVR
QENPSSVVGTFLCGPQALAKDLEKKCVKYSDVDPRRTKFYFNKENF

>medaka-Ol-Nox1: Ensembl No. ENSORLP00000001741

MANWIINNGFPAVMVLVWMTINTFLFVWYYLQYDRDDDFYYTRHLLGSALAWARAPAAVLNFNCM
LILLPVCRNLLSLIRGSFVCCSRTMRKQLDKNLSFHKLVAYMIALMTAVHTVAHLLNLEWLNNSKL
GVYGKLSTALSNLEDEGNETFLNPLQHIEADPQQKPIKFAFTSIAGLTGVVITLSLILIITSSMEVIRR
SYFEVFWYIHHLFIVFFVGLVFHGYGRIVRSQFNTDDHNATFCKDRPDDWGKIPECPIPQFKGGEP
MTWKWVIGPMIIYVCERVLRFIRYMQAVQYRKIVIHPSKVLELQLRKKGFKMEVGQYVFLNCPSIS
QLEWHPFTMTSAPEEDFFSVHIRSAGDWTDQLIDTMQKLPEGAQGPKMGVDGPFGTASEDVFDY
EVSMLVGAGIGVTPFASIMKSIWYKFKECDPKLRTRKIYFYWLCRETNAFEWFADLLQVLEKEMEE
RNLGDFLTYKLFLTGWDQGHTDQVIVHFDEDTDVVTGLKQKTHYGRPNWDKEFDQVRKENPASV
VGTFLCGPAALAKVLQKKCAKYSDVDPRKTKFYFNKENF

> fugu-Tr-Nox1: DDBJ™ No. BR000268

MGNWIINHGLTAFILVVWMGINIFLFVWFYLFYDLGERFFYTRHLLGSALAWARAPAAVLNFNCML
ILLPVCRNLLSLFRGSFVCCGRSMRKQLDKNLSFHKLVAYMIALMTAVHMIAHLLNVEWYNNSRQ
GVYDKLSTALSNLEDTKNTTYLNPIRSPTLSTYFVFTTIAGLTGVIITLALILIITSSMEVIRRSYFEVF
WYTHHLFIIFFAGLVFHGAGRIVRSQNSEPAHDATFCKDRTEDWGKIPECPIPQFSGGFPQTWMYVI
GPMVLYLCERLIRFIRYMQTVRYRKIVMRPSKVLELQLMKRGFKMEVGQYVFLNCPAISQLEWHPF
TMTSAPEEDFFSVHIRSAGDWTDKLIEIMQQLPEGAQGPKMGVDGPFGTASEDVFDYEVSMLVGA
GIGVTPFASILKSIWYKFKESNPKLRTRKIYFYWLCRETHAFEWFADLLQVLEKEMDERGMVDFLT
YKLYLTKWDDGHVNHIKVYPDTDVDMVTGLRQQTNYGRPNWDKEFEQVRKENPTSVVGTFLCGP
EALGEVLAKKCGKYSDVDPRKTKFYFNKENF

> human-Hs-Nox2: GenBank™ No. NM_000397

MGNWAVNEGLSIFVILVWLGLNVFLFVWYYRVYDIPPKFFYTRKLLGSALALARAPAACLNFNCML
ILLPVCRNLLSFLRGSSACCSTRVRRQLDRNLTFHKMVAWMIALHSAIHTIAHLFNVEWCVNARVN

NSDPYSVALSELGDRQNESYLNFARKRIKNPEGGLYLAVTLLAGITGVVITLCLILIITSSTKTIRRSYF
EVFWYTHHLFVIFFIGLAIHGAERIVRGQTAESLAVHNITVCEQKISEWGKIKECPIPQFAGNPPMT
WKWIVGPMFLYLCERLVRFWRSQQKVVITKVVTHPFKTIELQMKKKGFKMEVGQYIFVKCPKVSK
LEWHPFTLTSAPEEDFFSIHIRIVGDWTEGLFNACGCDKQEFQDAWKLPKIAVDGPFGTASEDVFS
YEVVMLVGAGIGVTPFASILKSVWYKYCNNATNLKLKKIYFYWLCRDTHAFEWFADLLQLLESQM
QERNNAGFLSYNIYLTGWDESQANHFAVHHDEEKDVITGLKQKTLYGRPNWDNEFKTIASQHPNT
RIGVFLCGPEALAETLSKQSISNSESGPRGVHFIFNKENF

> mouse-Mm-Nox2: GenBank™ No. NM_007807

MGNWAVNEGLSIFVILVWLGLNVFLFINYYKVYDDGPKYNYTRKLLGSALALARAPAACLNFNCM
LILLPVCRNLLSFLRGSSACCSTRIRRQLDRNLTFHKMVAWMIALHTAIHTIAHLFNVEWCVNARVG
ISDRYSIALSDIGDNENEEYLNFAREKIKNPEGGLYVAVTRLAGITGIVITLCLILIITSSTKTIRRSYFE
VFWYTHHLFVIFFIGLAIHGAERIVRGQTAESLEEHNLDICADKIEEWGKIKECPVPKFAGNPPMT
WKWIVGPMFLYLCERLVRFWRSQQKVVITKVVTHPFKTIELQMKKKGFKMEVGQYIFVKCPKVSK
LEWHPFTLTSAPEEDFFSIHIRIVGDWTEGLFNACGCDKQEFQDAWKLPKIAVDGPFGTASEDVFS
YEVVMLVGAGIGVTPFASILKSVWYKYCDNATSLKLKKIYFYWLCRDTHAFEWFADLLQLLETQM
QERNNANFLSYNIYLTGWDESQANHFAVHHDEEKDVITGLKQKTLYGRPNWDNEFKTIASEHPNT
TIGVFLCGPEALAETLSKQSISNSESGPRGVHFIFNKENF

> rat-Rn-Nox2: GenBank™ No. NM_023965

MGNWAVNEGLSIFVILVWLGLNVFLFVKYYKVYDDEPKYNYTRKLLGSALALARAPAACLNFNCM
LILLPVCRNLLSFLRGSSACCSTRIRRQLDRNLTFHKMVAWMIALHTAIHTIAHLFNVEWCVNARVG
TSDPYSVALSNIGDKENEEYLNFAREKIKNPEGGLYVAVTRLAGITGIVITLCLILIITSSTKTIRRSYF
EVFWYTHHLFVIFFIGLAIHGAERIVRGQTSDSLKEHNLDVCADKIKEWGKIKECPVPKFAGNPPM
TWKWIVGPMFLYLCERLVRFWRSQQKVVITKVVTHPFKTIELQMKKKGFKMEVGQYIFVKCPQVS
KLEWHPFTLTSAPEEDFFSIHIRIVGDWTEGLFNACGCDKQEFQDAWKLPKIAVDGPFGTASEDVF
SYEVVMLVGAGIGVTPFASILKSVWYKYCDNATSLRLKKIYFYWLCRDTHAFEFADLLQLLETQMQ
ERNNANFLSYNIYLTGWDESQANHFAVHHDEEKDVITGLKQKTLYGRPNWDNEFKTIASQHPNTR
IGVFLCGPEALAKTLSKQSISNSESGPRGVHFIFNKENF

> dog-Cf-Nox2: DDBJ™ No. BR000269

MGNWIENEGLSIFVILVWLGLNVFLFIWFYGVYNNGEEFFYTRKLLGFALPLARAPAACLNFNCML
ILLPVCRNLLSFLRGSSACCSTRIRRQLDRNLTFHKLVAWMIALHTAIHTIAHLFNVEWCVNARVNN
SDVYSIALSNLGDNPGESYLNFARRRIKNPEGGLYVAVTLLAGITGIVITLCLILIITSSTKTIRRSYFEV
FWYTHHLFVIFFIGLAIHGAERIVRGQTAASRLEHNYKVCADNISQWGKIPDCPIPQFSGNPPMTW
KWIVGPMFLYLCERLVRFWRSQQKVVITKVVTHPFKTIELQMKKKGFKMEVGQYIFVKCPKVSSL
EWHPFTLTSAPEEDFFSIHIRIVGDWTEGLFNACGCDKQEFQDAWKLPKIAVDGPFGTASEDVFSY
EVVMLVGAGIGVTPFASILKSVWYKYCNNATNLRLKKIYFYWLCRDTHAFEWFADLLQLLETQMQ
ERNNAGFLSYNIYLTGWDESQANHFAVHHDEEKDVITGLKQKTLYGRPNWDNEFKTIASQHPNTR

IGVFLCGPEALAETLSKQCISNSESGPRGVHFIFNKENF

> chicken-Gg-Nox2: DDBJ™ No. BR000270

MGNWVENEGLSIFVVLVWLGLNVFLFWWFYLAYDLPQNFFYTRVLLGRALALARAPAACLNFNC
MLILLPVCRNLLSFLRGSSACCSTRVRRQLDRNLTFHKMVAWMIALHTAIHTIAHLFNVEWSVHAR
VEEEGTLAAVLSRLGDSPNESYINFYRQTIPNPVGGLYVAFTYLAGLTGVIITLALILIITSSTKIIRRSY
FEVFWYTHHLFVIFFIGLVIHGAGRIVRGQTAVSLAEHIPEVCSKNFTDWGKKGACPVPQFAGNPP
MTWKWVVGPMFLYFCERLVRFWRSQQKVVITKVVIHPFKTIELQMMKKGFKMEVGQYIFVKCPAV
SKLEWHPFTLTSAPEEDYFSIHVRIVGDWTEGLFNACGCDKQEFQEAWKLPKIAVDGPFGTASEDV
FSYETVMLVGAGIGVTPFASVLKSVWYKYCHDATNLKLKKIYFYWLCRDTHAFEWFADLLQSLET
QMQERNNAEFLSYNIYLTGWDETQATHFVMHHEEEKDVITGLKQKTLYGRPNWENEFKTIARQH
PGSRIGVFLCGPEGLADTLNKQSISNSEADPRGVHFIFNKENF

> frog-Xt-Nox2: GenBank™ No. NM_001030518

MGNWIVNEGLSIAVIIVWLGLNGYLFWNFYLVYDEGEKYFYSRKLYGSALAWARAPAACLNFNCLL
ILLPVCRNLLSFLRGSSACCGRSLRRQLDRNLTFHKMVAWMIALHTAIHTGAHLFNVERLVDARVE
ANGTIQAALTDLGDREGESYLNFVRSRVPNPIGGINVAFTFLAGLTGVVITLALILIITSSTKTIRRSYF
EVFWYTHHLFVIFFIGLVIHGAGKIVRGQTDKSLEKHNSTECEDKFTEWGNITSCPIPQFAGNEPM
TWKWVVAPMVLYVFERLVRFWRSQQKVVITKVVTHPFKTIELQMKMKGFKMEVGQYIFVQCPAVS
KLEWHPFTLTSAPEEDFFSIHIRIVGDWTEGLFKACGCDKTEFQDAWKMPKIAVDGPFGTASEDVF
SYEVAMLVGAGIGVTPFASVLKSVWYRYVNDASTLRLKKIYFYWLCRDTQAFEWFADLLQSLETQM
QERDNANFLVYNIYLTGWDESQATAFSLHHDQEKDVITGLKQKTLYGRPNWENEFKTIANAHTSS
RVGVFLCGPESLAETLNKQSIANSTVDPRGVHFIFNKENF

>tetraodon-Tn-Nox2: DDBJ™ No. BR000271

MGNFAANEGLSVFVILVWLGINAFLFVHFYMAFLVERWFYTRVLLGHALSWARAPAACLNFNCML
ILLPVCRNLLSFLRGSIQCCSRTAARQLDRNLTFHKLVAYMIAFHTAVHIVAHLFNFEFFMDAQLNR
NSSYLPFILSEIGTGDNASFLNPIRTNETNPTIVMFTTIAGLTGVAITLALILIITSSMEVIRRSYFEVF
WYTHHLFVIFFIGLVLHGFGRIVRGQTAASLKTNKPEVCADRFEEWGRNGSDCAVPEFAGNPPMT
WKWVVGPMILYVCERIVRFYRSHQKVVITKVVMHPSKTLELRMKRKGFHMEVGQYVFIQCPSVSR
LEWHPFTLTSAPEEDYFSAHIRIVGDWTQALYEACGGDRSEPQEAWKLPKVAIDGPFGTASEDVFR
YEVVMLVGAGIGVTPFASILKSVWYKHIQKNQEVFTKKIYFYWLCPETEAFEWFADLLQSLEGQMA
DKGMTDFLSYNIYLTRWKEKEAAHFRVHHEAENDPITGLKQKTLYGKPNWDHEFASIASQHPRSK
VGVFLCGPPKLGQSLQKQCLSYSGADVKFIFNKENF

> fugu-Tr-Nox2: GenBank™ No. AB099894

MGNFAANEGLSTFVILVWLGINAFLFVHFYMAFLVDRWFYTRVLLGHALSWARAPAACLNFNCML
ILLPVCRNLLSFLRGSIQCCSRTAARQLDRNITFHKLVAYMIAFHTAVHIVAHLFNFEFFMDAQLNR
NSSYLPFILSEIGTGDNASFLNPIRTNETNPTIVMFTTIAGLTGVAITLALILIITSSMEVIRRSYFEVF
WYTHHLFIIFFIGLVLHGFGRIVRGQTPASLKSNDPTVCADQFEDWGRNGSNCAVPEFAGNPPMTW

KWVVGPMILYVCERLVRFYRSHQKVVITKVVMHPSKTLELQMKRKGFRMEVGQYVFIQCPSVSRL
EWHPFTLTSAPEEDYFSAHIRIVGDWTQALYEACGGDKSEPQEAWKLPKVAIDGPFGTASEDVFRY
EVVMLVGAGIGVTPFASILKSVWYKHIQNNQEVFTKKIYFYWLCPETEAFEWFADLLQSLEGQMTE
KGMTDFLSYNIYLTRWKEKEAAHFRVHHEAENDPITGLKQKTLYGKPNWDNEFTNIASKHPGSKV
GVFLCGPPQLGKSLQKQCLSHSEADVKFIFNKENF

> zebrafish-Dr-Nox2: GenBank™ No. NM_200414.1

MGNFAANEGLSVFVILVWLGINVFLFVYFYLAFLIDKYYYTRVILGHALSWARAPAACLNFNCMLIL
LPVCRNLLSFLRGSIQCCSRTAARQLDRNITFHKLVAYMIAFHTAVHIIAHLFNFERFMDSQLMINSS
HLPYVLSQIGNNDNRSYLNPIRSNDTNPTIVMFTTVAGLTGVVITLALILIITSSMEVIRRSYFEVFWF
THHLFIVFFIGLVLHGIGRIVRGQTDADLQVHDPTICHSKFEKWGQNVTDCPVPIFAGNPPKTWKW
VVGPMFLYVCERLVRFYRSQQKVVITKVVTHPSKTLELQMKKKGFKMEVGQYIFMMCPSISQLEW
HPFTLTSAPEEDHFSVHIRIVGDWTQALYSACGGDKTAVLDAWTLPKMAVDGPFGTASEDVFRYEA
VMLVGAGIGVTPFASVLKSVWYKHVQENQNVFTKKIYFYWLCPETQAFEWFADLLQSLEKQMSDK
NMSDFLSYNIYLTRWKDAEAAHLRVQYEAEDDPITGLKQKTRYGKPNWDNEFSLIASQHPGTKVG
VFLCGPTALGKALSKQCLSHTEGGTEFIFNKENF

>medaka-Ol-Nox2: Ensembl No.ENSORLP00000002049

MGNFVANEGLSIFVILVWLGINAYLFVQFYMNFLVERWFYTRVLLGHALSWARAPAACLNFNCMLI
LLPVCRNLLSFLRGSIQCCSRTAARQLDRNLTFHKLVAYMIAFHTAVHIIAHLFNFEYFMDAQLNRN
HSHLPFILSEIGNDENVSFLNPIRSNETKSPTIVMFTTIAGLTGVVITLALILIITSSMEVIRRSYFEVF
WYTHHLFVIFFIGLVFHGFGRIVRGQTSRSLDSNDPDVCADRFEDWGKNESGCAVPAFAGNPPMT
WKWVVGPMFLYVCERLVRIYRSHQKVVITKVVMHPSKTLELQMKKKGFHMEVGQYVFIQCPSISR
LEWHPFTLTSAPEEDYFSVHVRIVGDWTQALYEACGGNKSELQEAWKLPKVAIDGPFGTASEDVFR
YEVVMLVGAGIGVTPFASILKSVWYKHIQNNEGVFTKKVRYIRLLRMSSSYLLFTLMVQKKIIRAWD
RHRGNWLATPVARFGQLKNQAAHLRVHHEDENDPITGLKQKTLYGKPNWDNEFTNIASTHPGSK
VGVFLCGPPMLGKSLEKESISHTEAGVKFIFNKENF

>ascidian-Ci-Nox2: DDBJ™ No. BR000272 (Chromosome 6q: 1.91 M)

MNARLNGFLVNEFPKYIVFLLWLGLNGFLFGYYYNFYNTKKTFFYTRVLLGPALALARAPAACLNL
NCLLVLLPVCRNLLSLFRKACMCCPRRIRRVLDKNIKFHRMCAYMIVLMTLIHYFAHCFNVDFFTS
AYQSKILATDTPAIIQQKKLIAKLIQIGNNGNETYLNPIRKSVFSVGAVFLLTGGWTGVIITLSLFFMV
TSSLEFIRRSYFEVFWFTHHLFIVFYGFLVVHGISMQVRGQTPQSLTVHDPIRCSTIDPATWSQNNC
PTPVFAGSPPMTWKWVIAPMVLYVIERIIRLVRFNQQVEVLKVIKHPSRVLEIQMRKNGFFAEVGQY
VFIMCPQLSQLEWHPFTLTSAPEEDYFSIHVRIVGDWTTGLSKVLGADEAGNEVQPSWKMPRLAID
GPFGTASEDVFNYPVAICVGSGIGVTPFASLLKSVWYKNLNPEHEMVLKKVYFFWICPETHAFEWF
GDLLKYLERQLTEIGRQDLIEYHIYLTRGWDHKQAKAIYAHEEDTHDVITGLEQKTNYGRPNWDEI
FSKTARDYPNTHIGVFFCGVAALSAKLHKMSNKHSGGGVYFHYNKENF

>sea urchin-Sp-Nox2A: GenBank™ No. AY704915 (initially termed Nox-U1)

MGDKFLNEGLKYFFLLLWLAANVAYWVVTFLVYEQGPQYFYIRRITGVGLSIAKAAGAALNLNSMII
LLPICRNLISFFRGSCATNTLCRRSVRRQLDKNLTFHKTVAYMIVVWTIVHVVAHAFNFRNLYNHYR
CVTTDNDELCEGISAIGRKFKAKPEDNWLNPIQGAKTLPAGLGLIEQALIPIAGWSGAVLTLALILM
FSSATEFIRRSYFETFWITHHLFIVYFAMLLAHGVGGIIRSQTNLDRHDVVFCSENLDVWGPTSAQC
EDPVFKDGSAASYKWVSGPLFIYLLERTIRFWRSCQTVTLTKVVKHQSKVIELQMKKKGFKMEAG
QYIFLKCPSISHVQWHPFTLTSAPEEDHFSVHIRVVGDWTRDLFKAMGADKPEQQSQDELARVAVD
GPFGTASIDIFKYQVAICVGAGIGVTPFASILKSIWLKSVNNSASLKLKKVYFFWICPDTNAFEWFST
LLDSIDTHFTEQGKPDFLKYYIYLSRGWNNTQAKNIYLQEEQEIDAITGLRQKTHYGRPKWDSNFK
MIAEENPGRVSSVFFCGPKALSSVLHENANKFTSLTPDGAKFFYNKENF

>sea urchin-Sp-Nox2B: GenBank™ No. XM_001180659

AAWAIVNLIIWLVTFFKYMDNANYIYTKYLMKNGLPVARASAACLNFNSMLILFPVCRNMISYLRGS
CESTKFSRRNLRRQLDKNITFHKLIAYAIGFFVILHVGAHCFNLQNLYNGRKATSEDDWLANRLSQ
PSFDLNPFKTIRSSDVSGLGVIGPGLSLLAGWTGAVLALTYILMFTSATEFIRYYFETFWLTHHLFVI
YYAMLMTHGMGGVVKYQTNVDEHDPVECMVDEETFDQCVIDNPPLFAGTPGASWKWCVTPLCV
YFLERILRMIRTWPDVTIVQVVQHQSKVIELRMKKQGFKMLPGQYIFLKCSPISKVQWHPFTLTSAP
EEDYFSLHIRRVGDWTDELAVKMGADQAEPLSITQLPRVQVDGPFGTSCTDIFDYDVVMCVSAGIG
VTPYASTLKSIWISSRQNFCTLHLKRMYFYWICRDTHAFEWFVELLSSLELILRQIDKEHLLSYSIYL
TRGWDYTQAKNIFMQEDREIDAVTGLRQKTHYGRPKWDSNFSYIAEKNPRVSRIWNATIGVFFCGP
KSLSTILHQSCNKHTSDESDGTRFVYYKENF

> human-Hs-Nox3: GenBank™ No. NM_015718

MMGCWILNEGLSTILVLSWLGINFYLFIDTFYWYEEEESFHYTRVILGSTLAWARASALCLNFNCM
LILIPVSRNLISFIRGTSICCRGPWRRQLDKNLRFHKLVAYGIAVNATIHIVAHFFNLERYHWSQSEEA
QGLLAALSKLGNTPNESYLNPVRTFPTNTTTELLRTIAGVTGLVISLALVLIMTSSTEFIRQASYELF
WYTHHVFIVFFLSLAIHGTGRIVRGQTQDSLSLHNITFCRDRYAEWQTVAQCPVPQFSGKEPSAWK
WILGPVVLYACERIIRFWRFQQEVVITKVVSHPSGVLELHMKKRGFKMAPGQYILVQCPAISSLEWH
PFTLTSAPQEDFFSVHIRAAGDWTAALLEAFGAEGQALQEPWSLPRLAVDGPFGTALTDVFHYPVC
VCVAAGIGVTPFAALLKSIWYKCSEAQTPLKLSKVYFYWICRDARAFEWFADLLLSLETRMSEQGK
THFLSYHIFLTGWDENQALHIALHWDENTDVITGLKQKTFYGRPNWNNEFKQIAYNHPSSSIGVFF
CGPKALSRTLQKMCHLYSSADPRGVHFYYNKESF

> mouse-Mm-Nox3: GenBank™ No. AY573240

MPVCWILNESGSFVVALLWLAVNAYLFIDTFFWYTEEEAFFYTRVILGSALAWARASAVCLNFNCML
ILLPVSRNFISLVRGTSVCCRGPWRRQLDKNLNFHKLVAYGIAVNSVIHIVAHLFNLERYHLGQAKD
AEGLLAALSKLGDAPNESYLNPVRTFDMGTTTELLMTVSGITGLGISLALVFIMTSSTEFIRRSSYEL
FWYTHHIFVFFFISLAIHGGGRIIRGQTPESLRLHNVTYCRDHYAEWQAAALCPVPQFSGKEPSAW
KWALGPVVLYACERIIRFWRSHQEVVITKVVSHPSAVLELHMKKRDFKMAPGQYIFIQCPSVSPLE
WHPFTLTSAPQEDFFSVHIRASGDWTEALLKAFRVEGQAPSELCSMPRLAVDGPFGGSLADVFHYP

VSVCIATGIGVTPFASLLKSVWYKCCESQSLPELSKVYFYWICRDAGAFEWFADLLLSLETRMSEQG
KAHLLSYHIYLTGWDENQAIHIALHWDESLDVITGLKQKAFYGRPNWNDEFKQIAYNHPSSSIGVF
FCGSKAMSKTLQKMCRLYSSVDPRGVHFYYNKENF

>dog-Cf-Nox3: DDBJ™ No. BR000273

MMGCWILNESLSVILVLSWLGVNLYLFIDTFCWYEEEESFLYTRVILGSTLAWARASAVCLNFNCML
ILLPISRNLISFMRGTSTCCRGLWRRQLDKNLKFHKLVAYGIAVNATIHIVAHLCNLQRYHWSQSAE
VQGLPATLSKLGNAPNESYLNPIRTFHTNTITELLTTIAGVTGLIISLALVLIMTSSTESIRQVSYELF
WYTHHVFIIFFIGLAIHGAGRIVRGQTPESQLLHNVTFCRDHHAQWQKMAQCPMPQFSGKEPSAW
KWVLGPVVLYACERIIRFWRFQQEVVITKVVSHPSGVLELHMKKRNFKMAPGQYILVQCPSISWLE
WHPFTLTSAPQEDFFSLHIRVAGDWTEALWKAFGAEGQALKEPWSLPRLAVDGPFGTTLTDVFHY
PVSVCIAAGIGVTPFASLLKSIWYKCESQTQLKLSKVYFYWICRDPKAFEWFADLLLSLETLMSERG
KAHFLSYHIFLTSWDENQAVHIALHWDENTDVVTGLKQKTFYGRPNWSNEFRQLAYAHPSSSIGV
FFCGPKALSKTLQRMCHLYSSADPRGVHFYYNKESF

>chicken-Gg-Nox3: GenBank™ No. XM_426166.1

MACWILNEKLSVLLLLVWLGLNLYLFIDTFHWYEDEDAYVYTRIMLGSTLAWARASATCLNFNCML
ILLPVSRNLISFLRGASACCGGAPRRQLDKNIAFHKVVAYGIAVNATIHIVAHLINIERYHNSQSKEAG
GLQNKLSGLGKRPNESYLNPIRTYETNTTGEVLTTIAGVTGVMITVAFVLIVTSSTELIRRSCYEVFW
YTHHLFVVFFIGLIIHGTGQLVRGQTPHSLLLHNITYCKEHYLEWEKATQCPLPQFSGNKPVAWKW
VVSPVVLYICERIVRFWRFQQEVVITKVVTHSSGVLELHMKKHGFKMEAGQYIFLQCTSISPLEWH
PFTLTSAPEEDFFSVHIRVAGDWTAALFKAFGAEEKTFKELWMLPRLVVDGPYGSATTDVFHYGVS
VCIAAGIGVTPFASILKSIWYKSCNPNTVLVLQKVYFYWICRDPSTFEWFADLLFLLETKMVEKGKN
DFLSYHIFLTGWDENQATHIALHYDEKMDVITGLRQKTFYGRPNWDSEFKQLAENHPSNSIGVFF
CGPKNLSKILQKMCSSYSTVDPRGVQFHYNEESF

>rat-Rn-Nox3: GenBank™ No. NM_001004216

MPTCWILNESVSFVVALLWLAINIYLFIDTFCWYAEEESFFYTRVILGSALAWARASAVCLNFNCMLI
LLPVSRNFVSLVRGTSVCCRGPWRRQLDKNLKFHKLVAYGIAVNSVIHIVAHLFNLERYHLGQAKD
AEGLLAALSKLGNAPNESYLNPVRTLYTGTTTQLLMTVSGITGLVISLALILIMTSSTEFIRQSSYELF
WYTHHIFIFLFISLAIHGGGRIIRGQTPESLRLHNVTFCRDHFDEWQEAASCPVPQFSGKEPSAWK
WTLGPVVLYACEIIIRFWRSHQEVVITKVVSHPSAVLELHMKKRDFKMAPGQYIFIQCPSISPLEWH
PFTLTSAPQEDFFSVHIRASGDWTEALLKAFGAEGQAPSELCSMPRLAVDGPFGGSLADVFHYPVS
VCIATGIGVTPFASLLKSVWYKCCESQSLPGLSKVYFYWICRDAAAFEWFADLLLSLETQMSEQGK
AHLLSYHIYLTGWDEYQAIHIALHWDESLDVITGLKQKTFYGRPNWNEEFKQIAYNHPSSSIGVFF
CGPKAMSKTLQKMCRLYSSSDPRGVHFYYNKENF

>human-Hs-Nox4: GenBank™ No. NM_016931.2

MAVSWRSWLANEGVKHLCLFIWLSMNVLLFWKTFLLYNQGPEYHYLHQMLGLGLCLSRASASVL
NLNCSLILLPMCRTLLAYLRGSQKVPSRRTRRLLDKSRTFHITCGVTICIFSGVHVAAHLVNALNFSV

NYSEDFVELNAARYRDEDPRKLLFTTVPGLTGVCMVVVLFLMITASTYAIRVSNYDIFWYTHNLFFV
FYMLLTLHVSGGLLKYQTNLDTHPPGCISLNRTSSQNISLPEYFSEHFHEPFPEGFSKPAEFTQHKF
VKICMEEPRFQANFPQTWLWISGPLCLYCAERLYRYIRSNKPVTIISVMSHPSDVMEIRMVKENFKA
RPGQYITLHCPSVSALENHPFTLTMCPTETKATFGVHLKIVGDWTERFRDLLLPPSSQDSEILPFIQ
SRNYPKLYIDGPFGSPFEESLNYEVSLCVAGGIGVTPFASILNTLLDDWKPYKLRRLYFIWVCRDIQS
FRWFADLLCMLHNKFWQENRPDYVNIQLYLSQTDGIQKIIGEKYHALNSRLFIGRPRWKLLFDEIA
KYNRGKTVGVFCCGPNSLSKTLHKLSNQNNSYGTRFEYNKESFS

>mouse-Mm-Nox4: GenBank™ No. NM_015760

MAVSWRSWLANEGVKHLCLLIWLSLNVLLFWKTFLLYNQGPEYYYIHQMLGLGLCLSRASASVLN
LNCSLILLPMCRTVLAYLRGSQKVPSRRTRRLLDKSKTLHITCGVTICIFSGVHVAAHLVNALNFSV
NYSEDFLELNAARYQNEDPRKLLFTTIPGLTGVCMVVVLFLMVTASTYAIRVSNYDIFWYTHNLFF
VFYMLLLLHVSGGLLKYQTNVDTHPPGCISLNQTSSQNMSIPDYVSEHFHGSLPRGFSKLEDRYQK
TLVKICLEEPKFQAHFPQTWIWISGPLCLYCAERLYRCIRSNKPVTIISVINHPSDVMELRMIKENFK
ARPGQYIILHCPSVSALENHPFTLTMCPTETKATFGVHFKVVGDWTERFRDLLLPPSSQDSEILPFI
HSRNYPKLYIDGPFGSPFEESLNYEVSLCVAGGIGVTPFASILNTLLDDWKPYKLRRLYFIWVCRDIQ
SFQWFADLLCVLHNKFWQENRPDFVNIQLYLSQTDGIQKIIGEKYHTLNSRLFIGRPRWKLLFDEIA
KCNRGKTVGVFCCGPSSISKTLHSLSNRNNSYGTKFEYNKESFS

>rat-Rn-Nox4: GenBank™ No. NM_015760

MALSWRSWLANEGVKHLCLLVWLSLNVLLFWKTFLLYNQGPEYYYIHQMLGLGLCLSRASASVLN
LNCSLILLPMCRTVLAYLRGSQKVPSRRTRRLLDKSKTLHITCGITICIFSGVHVAAHLVNALNFSVN
YSEHFLALNAARYQNEDPRKLLFTTVPGLTGVCMVVVLFLMVTASTYAIRVSNYDIFWYTHNLFFV
FYMLLLLHVSGGLLKYQTNLDTHPPGCISLNRTPSQNMSIADYVSEHFHGSLPGGFSKLEDHYQK
TLVKICLEEPKFQAHFPQTWIWISGPLCLYCAERLYRCIRSNKPVTIISVINHPSDVMELRMIKENFK
ARPGQYIILHCPSVSALENHPFTLTMCPTETKATFGVHFKVVGDWTERFRDLLLPPSSQDSEILPFI
QSRNYPKLYIDGPFGSPFEESLNYEVSLCVAGGIGVTPFASILNTLLDDWKPYKLRRLYFIWVCRDIQ
SFQWFADLLYVLHNKFWQENRPDFVNIQLYLSQTDGIQKIIGEKYHTLNSRLFIGRPRWKLLFDEIA
KCNRGKTVGVFCCGPSSISKTLHNLSNRNNSYGTKFEYNKESFS

>dog-Cf-Nox4: GenBank™ No. XM_542262.2

MENAVMILCTEYYEHDEEYIDYRFIWLSLNVLLFWKAFLLYNQGPEYHYLHQMLGLGLCLSRASASVLNLN
CSLILLPMCRTLLAYLRGSQKVPSRRTRRLLDKSRTFHITCGVTICIFSGVHVAAHLVNALNFSVNYNEDFTE
LNAARYRDEDPRKLLFTTVPGLTGVGMVLVLFLMITASTYAIRVSNYDIFWYTHNLFFVFYMLLMLHVSGG
LLKYQANLDTHPPGCININGTRYQNIHLPNYRSEHFHESFPGGLSKPDELTQNRSVNICMEEPRFQANFPQLP
MWELTPCTLCLYCDTCFTSVKLNAPKVIVSNMINHPSDVMEIRMIKENFKARPGQYIILHCPSVSALENHPFT
LTMCPTETKATFGVHLKIVGDWTERFRDLLLPPSNQDSEILPVIQSRKYPKLYIDGPFGSPFEESLNYEVSLCV
AGGIGVTPFASILNTLLDDWKPYKLRRLYFIWVCRDIQSFRWFADLLCVLHNKFWQENRPDYVNIQLYLSQT

DGIQKIIGEKYQALNSRLFIGRPRWKLLFDEIAKCNRGKTVGVFCCGPNSISKTLHKLSNRNNSYGTRFEYNK
ESFS

>frog-Xt-Nox4: Ensembl No. ENSXETP00000025676

MALPCASWLSNEALKHLFLLSWLALNIGLFYKTFTVYYSGPQYFYLHQMLGLGLCVSRASASVLN
LNCSLVLLPMCRTVIGLLRGPKMVNIHWKTRRMLDKHKTFHAACGLAICLFSAHVLNAVNFSVNY
NHEFPSINVARYKNEVVPGVTGVLMVLILFLMCTASTSSIRTANYGIFLHTHNLFFIFYLLLLLHACA
GVLKYQSNLEEHPPGCLYLNRSAQGEVPGAAADGGEFPGRAARALMGSFFSHEDMSVHNNSEKIC
TKGPTFRPHFPETWLWISGPLCLYCAERLYRYIRSSKPVTIVAVITHPCDVVEIRMVKEKFSARPGQY
ITLLCPSVSALETHPFTLTMCPTESKATFAIHIKVVGDWTERFYELLESHLTAGTEILPKCQQRKNP
KIYVDGPFGSPSEEVFNYQISLCIAGGIGVTPFASVLNRLLDSWDGYKLQRLYFVWVCRDIHSFLWF
ADLLCLLHRKLWQENRPDYLNIQLYLSQTNGIQNIIGEKYQALNSRLSIGRPQWKLLFEEVAKSSRG
KTVGVFCCGPKGISKELHKLCNSANQYGTTFEYNKESFT

>chicken-Gg-Nox4: DDBJ™ No. BR000274

PSYLTLPVFVQLGLCVSRASASVLNLNCCLVLLPMCRILLAFLRGSQKVASRKTRRLIDKSKTFHVT
CGVTVCIFSVLHVAAHLVNALNFSENYNEDFLAINAANYRGEDPRKLLFATVPGLTGVIMVLVLFL
MCTASTYAIRVSNYDIFWYTHNLFFVFYILLMLHVSGGVLKYQTNLEEHPPGCFNPNKTLLGNMTV
PKSFEELFPDYTTEPFPEDLTFPQPLVQSNFMRICSKEPKFQSHFPETWFWISGPLCLYCVERLYRYI
RSNKPVTITSVISHPSNVLEVRMIKDDFRARPGQYVILHCPRVSGLESHPFTLTMVRNKNTKATFGV
HLKVVGDWTERFRDLLLLHSNQDAEILPIFQQRHYPKLYVDGPFGSPFEESLNYEVSLCVAGGIGV
TPFASVLNALLDGWKCYKLRRLYFIWVCRDVESFRWFADLLCMLHNKLWQENRPDYINIQLYLSQT
DGIQKIIGEKYQALNSRLLIGRPRWKLLFDEIAKYNRRKTIGVFCCGPSKMSKILHKLSNSSNPYGT
RFEYNKESFS

>medaka-Ol-Nox4: Ensembl No. ENSORLP00000013799

MAVSVRSWLANEAGKHFVLMLWLAANTWLFLDTYLLYSTGQQYHYLYQMLGLGLCISRASASVLN
LNCSLVLLPMCRSLLTFIRGSHTMSTRRMRRLLDKSKSFHVACGIAICIFSAVHVSAHLINVVNFSAG
FSEDFPALNLARYKGEDPKLIIFTTIPGVSGVLLVLILLLMFISSSHCIRVCNYEIFWYTHNLFIVFYII
LMVHMAGGALKFQTNIEAHPPGCLRASQSLRQQQQAEDLDQKQRCKEDAHFQPHYPQTWLWVS
GPLCLYCVERFYRYIRSSHPVTIVTVIRHPCDVVELRMLKKNFRARPGQYILLNCPGVSPFENHPFT
LTACPTENKQTFGIHLRIVGDWTEHFAHLLLPQPRAALEILPVVHQRRYPTLYVDGPFGSPSEEVFN
YDVSLCIAGGIGVTPFACVLNALIPSERWQSFRLQRLYFVWVCRELQSFYWFAELLCALHEKLWQD
NRPDYLNVKLYVTQKDSLQSMSELRYRPLAARLQVGRPKWKLLFDEIGKSNKDKRVGVFCCGPKG
ISRTLHRLCNSAKSSGATFEFNKESFS

>fugu-Tr-Nox4: DDBJ™ No. BR000275

MSVRSWIANEGGKFLVLMLWLGVNTWMFLNTFLLFSSGEQYYYLYKMLGLGLCISRASASVLNLN
CSLVLLPMCRSLLKFIRGTHTVSSRKTRRMLDKYKTFHVACGLAICIFSAIHVSAHLANAANFSTSY
SEEFPSLNVARYRGEDPKWIILTTIPGVTGIVLVLILFLMLMSSSNCVRSFNYEIFWYTHNLFIVFYIV

LMVHMVGGVLKYQTNIEAHPPGCLTANQSNMDPQAKEMEKADNEERRCTEEAHFQGHYPQTWI
WLSGPLCLYCAERFFRYIRSCDPVTIVTVIRHPCNVIELQMLKNKFAARPGQYILLNCPAVSSFENHP
FTLTTCPTENKKTFSIHLRIVGDWTERFTQLLLTGSRTDMKTLPMVQHRKYPKIYVDGPFGSPSED
VFNYDVSLCVAGGIGVTPFACMLHTLLDRGWTHFRLQRLYFVWVCSELQSFYWFAELLCSVHHKL
WQENRPDYFNMKLYVSQTDSLENMSAKYRPLTSRLLVGRPRWKLLLNELGKTNKHKRIGVFCCG
PKAISRTLHRFCNSFQSSETVFEFNKES

>zebrafish-Dr-Nox4: Ensembl No. ENSDARP00000079892

FSARFSQLLLQPSNSSEILPMMHQRRYPTVHVDGPFGSPSEEVFNYEVSLCVAGGIGVTPFACVLQA
LYDDWCHYKLKRLYFVWICRDIQCFYWFADLLCGLYEKLWRDNRPDYLNVQLYLSSSQGLQSIGEE
RYRFLSSRLRIGRPNWKLLFQEIGRANQLKRVGVFCCGPKGISKALHTLCNSNPHSHTAFEYNKES
FS

>tetraodon-Tn-Nox4: Ensembl No. GSTENP00010317001

QCPTEKRKTFSIHLRVVGDWTERFTQLLLPGSRTDLKILPVVQQRQYHKIYVDGPFGSPSEEVFNYDVSLCV
AGGIGVTPFACVLHTLLDGWTHFRLQRLYFVWVCRELRSFYWFAELLCSVHHKLWQENRPDYFNMKLYVS
QTDTLQVMSEEKYRPITSRLLVGRPRWKLLLNEVGKTNKHKRVGVFCCGPKAISSTLHRLCNSFQSSETVFE
FNKESFN

>ascidian-Ci-Nox4: GenBank™ No. NM_001033829 (Chromosome 6q: 909K)

MQLKNYLVNDGLRLFIWVAWISINASLFYFTFMYYYNGIQFYYLHQMLGYGLCISRASAACINLNSS
FILFPMCRGLVTFMRGLPRGVGRQVRRLLDRGRSFHILCGYILCLLAGVHCAAHAYNAVYFSKYYN
SRYKDLNVAKYSNQNPLLMLVTSLSGITGILLVISLVVISAFASRPIRRNNHNKFWKTHHIFIVFYALI
FIHAMDGVIKYQTNVDQHKPGCFILIDQTNNTSNISMVQPEPEPFPNHPSMVKPKMVAVPEPPHRE
PFPNMKLASKMPSKTDMKFMNNTAPQPHHVIMPEPEPMPHKGAHGAPNTTRIFVHGSWVEVME
CVQPPPKFSSCRQEAWLWLCAPLIIYVIERIGRHFRSSHDVTTIVKFIEHPCDVIELRLYRNGFSAKP
GQCIWVRCPQLSKVESHPFSLTSVPSKDDPTFGIHVKLRGDWTEELRDLMVRELNPVVEILKDKII
AGGNFLEKDDELSNRGETEYKCNPDNSQYLATMASPNQSINKPCMVPNFRTQETDFQMQCTLSN
STISVYDNHEMPTHQENIDDQTCKKTSSTCLSQYSLESESQRNLPKSTTLHSKDQQNLQNETTLYS
EDQRNVPKSTALVSEACSTKNDIHLPCNQTVSKQLPILCVEGPTGGAMEDIFKYKISMCVAGGIGVT
PYASVLNALLKDEDLFSRMKLKRLYLIWSCKDPRSFSWFASLIRDVQIVLWKRNCPDLLSVRLHITG
SNTLQSEESGDQLLSDIQGCHVAYGRPDVTQVFEEIRTAAQYQRSTVGVFCCGHRLLVSSVKHHCL
KTKSSKVKFLFNKEAF

>mosquito-Ag-NoxM: GenBank™ No. XP_310312

LLWTGFNFVVFCKAFSNYHHDVEYYYLSRILGNGLCVSRGTAPVLNVTMALITLPTCKTFNLLLHK
LFGRCSTRLLVHYLEKTKVLHLILGCSLLIVAIVHSVAHFVNIVNFIDNYDERYREINWANGPDDNV
LRLLFATPTGFSGCIMLLTLAAMAYLARRSMRDRFYNSFFTSHHLFLVFYGMMFYHPLRLVHGGST
LNDAIDRFNIIKHQTNVDKHKIMCDLVDNVTLHSNEDLLVLCEEPPQFSAGTKRAWIWPLVGLAIY
LADISFRYLTSHSERYRVTTVQTYAMAGHAIHLRLQFCRKAMVKILPGQYVLLQCPAISTLEWHPFT

ITELPIEGRNDITLTIKVRGDWTEELYDRIVQREQCKRNLGGVDPYRRIEFLLDGPYPSVMSNMLDC
KRILFVGAGVGITPFVTIMRLLLSSNVDQPARVHLVWIARNLETFLWFSDEIARLQEKFWSQNKPDR
FWVKLYWTQNYDEHLLAECFGDMPSIKSRMHRGRPNWNDVFIDLVTLYPKKSVSVFSCGPKELTK
EIRLKCKEYSKHGCKLSYFHEGFG

>mosquito-Ae-NoxM: GenBank™ No. EAT37894

MAIITLPVSRSFNVLLNALFGRWSIRALVFYLEKIKVLHLFLGTGLIIVGVIHSIAHFINIINFVDNYDA
KFDAINWATGKDDSKLRLLVATPTGFSGCVMLVTLFAIAYFSSRQMRDRFYNSFLASHHLFLVFYGM
MFYHPLSNIIKHQTNLKAHPNGCDIIDDHVFRNDSVLQAICSEEPKFSAGDKSAWIWPLMGLSIYIL
DIIIRYLIAHSDRRKVSTLQSYVLPANGVYLRLRFTSSKRIVISAGQYVLLQCPAISTLEWHPFTVVDV
SIHNTVSLTVAVRGDWTQRLYDLVSEKERLKQSGAGHDALGRLQFLLDGPYPSAMTGMLKCKRIVY
IGAGVGITPFAGFVRHLLNFNTDRPSRIHLIWIVRKAEMFTWFADELKRLQERFWKQNKPDRFTLK
LFLTRNYNVNVIDEYFGDYPTLKARINKGRPNWDEVFLDLTALYAGKSVTVFSCGPKGMTKELKG
MCREYRKHACKFTYLHEGFG

>human-Hs-Nox5: GenBank™ No. AF353088

MNTSGDPAQTGPEGCRGTMSAEEDARWLRWVTQQFKTIAGEDGEISLQEFKAALHVKESFFAERF
FALFDSDRSGTITLQELQEALTLLIHGSPMDKLKFLFQVYDIDGSGSIDPDELRTVLQSCLRESAISL
PDEKLDQLTLALFESADADGNGAITFEELRDELQRFPGVMENLTISAAHWLTAPAPRPRPRRPRQL
TRAYWHNHRSQLFCLATYAGLHVLLFGLAASAHRDLGASVMVAKGCGQCLNFDCSFIAVLMLRRC
LTWLRATWLAQVLPLDQNIQFHQLMGYVVVGLSLVHTVAHTVNFVLQAQAEASPFQFWELLLTTR
PGIGWVHGSASPTGVALLLLLLLMFICSSSCIRRSGHFEVFYWTHLSYLLVWLLLIFHGPNFWKWLL
VPGILFFLEKAIGLAVSRMAAVCIMEVNLLPSKVTHLLIKRPPFFHYRPGDYLYLNIPTIARYEWHPF
TISSAPEQKDTIWLHIRSQGQWTNRLYESFKASDPLGRGSKRLSRSVTMRKSQRSSKGSEILLEKHK
FCNIKCYIDGPYGTPTRRIFASEHAVLIGAGIGITPFASILQSIMYRHQKRKHTCPSCQHSWIEGVQD
NMKLHKVDFIWINRDQRSFEWFVSLLTKLEMDQAEEAQYGRFLELHMYMTSALGKNDMKAIGLQ
MALDLLANKEKKDSITGLQTRTQPGRPDWSKVFQKVAAEKKGKVQVFFCGSPALAKVLKGHCEK
FGFRFFQENF

>dog-Cf-Nox5: DDBJ™ No. BR000277

DPAQPDLESCRGTMSTEEDAKWLQWVTHQFETIAEKDREINLQQFKTALNVKEAILFAERFFTLFD
SDGSGTITLQELLEALTLLIHGNPMDKLKFLFQVYDVDGWARQGGVCRYRVGPSTRPHSAASPLGT
GSGSIDADELRTVLRSCMRESAISLPDEKLDQLTLALFESADKDCNGAITFDELRDELQRFPGVME
NLTIRCGSASRVGTARVQVPLQLRRLALTWAYWHNHRSHLLCLAAFAGLHLLLFALAASEHRARGA
SVMVAKGCGQCLNLDGSFIAVLMLRRCLTWLRATWLAQVLPLDQNIQFHQFVGYVVIVLSLVHTVA
HIVNFALQAQAEASPFQFWELLLTTRPGIGWIHGLASPTGVALLLLLLLMFACSSSCIRRSGHFEVF
YWTHLSYLPMWILLILHGPNFWKWLLVPGTLFFLEKIIGLAVSRMAALCIVEVNLLPSKVTHLLIKR
PPLFHYRPGDYLYLNIPTIARYEWHPFTISSAPEQKDTIWLHIRSEGQWTNRLYESFKTSCPMDCDP
KPLSRSLKMRRSQRRPEVSEKSSENHLFCNIKCYIDGPYGTPTRRIFASEHAVLIGAGIGITPFASILQ

SIMYRHQKRKNICPSCQHSWMDSAQDEDMKLHKVDFMWINRDQRSFEWFVSLLTKLEMDQAEIS
QEGPFLELHMYMTSALGKNDMKAIGLQMALDLLAKKEKKDSITGLQTRTQPGRPDWNKVFQKVA
AEKKGKVQVFFCGSPALAKVLKGHCEQFGFKFFQENF

>cow-Bt-Nox5: DDBJ™ No. BR000276

MSAPGDTAWTDSEGFRGTMNAEEDAKWLQWVTHQFKTIAGEDGEINLQDFKKALKVKESFFAER
FFVLFDSDGSGTITLQELQKALTLLIHGSPMDKLKFLFQVYDVDGKHPLWDGRRTQREGQRERPVS
VTAQHWASSSPETGSGSIDADELRTVLQSCLYESAISLPKEKLDQLTLALFESADKDCSGTITFEELR
DELQRFPGVLENLTISAAHWLTPPAPQRHRRQPRLLTSAYWHNHRSHVLCLAVFVGLHMLLFALAA
SAYRAFGSSVMVAKGCGQCLNFDCSFIAVLMLRRCLTWLRATWLAQVLPLDHNIQFHQLMGYVVV
GLSLVHTVAHVVNFALQAQSETSPFRFWELLLTTRPGIGWVHGSASPTGVALLLLLLLMFACSSSCV
RRSGHFEVFYWTHLSYLPMWLLLILHGPNFWKWLLVPGTLFFLEKTISLAASRMAALHIVEVNLLP
SKVTHLLIKRPPLFHYRPGDYLYLNIPSIARYEWHPFTISSAPEQKDTIWLHIRSQGQWTNRLFESF
KKPEPVFCGSKRLSRRLEMKRSQRKPQVSEMSSENHQFCNIKCYIDGPYGTPTRRIFASEHAVLIGA
GIGITPFASILQSILYRHQKRKHICPNCQHSWMESGQDEDMKLHKVDFIWINRDQQSFEWFVSLLT
KLEMDQAEETQVGRFLELHMYMTSALSKNDIKAIGLQMALDLLAKKEKKDSITGLQTRTQPGRPD
WNKVFQKVAAEKKGKVQVFFCGSPALAKILKGHCEQFSFKFFQENF

>opossum-Md-Nox5: DDBJ™ No. BR000304

MNADDDAKRLEWVSFQFEIAGEDREIDLQEFKKALKVKESFFAERFFALFDSDGSGTITLQELLGA
LNLLIHGNTMDKLKFLFQVYDVDGLCYRSYSPSTGSQWSLGKCSSPLGHGNGSIDPDELRVVLQSC
LKESAISLPEEKLDDLTLALFESADKDHSGSITFEELQEELDKFPEVMENLTISAANWLKPPSSKKR
PQIPRHLTSAYWHNNCGKLAVLAVYIGLNILLFTLAALKYQSSGTRIMIARGGGQCLNFNCSFIVVL
MLRRCLTWLRATWLARVLPLDQNVEFHQLIGYVVVGFSFLHTTAHVVNFAQLAQSENSTFQFWEY
LLTTRPGIGWVYGTASLTGIVLQLLILIMLVCSCSFVRRSGHFEVFYWTHLLYISIWALLIVHGPNFW
KWLLVPGLLFFLEKVVGLVLSRMAALSIVEVNLLSSKVTHLVISRPPFFRYKPGDYIYLNIPAIAKYE
WHPFTISSAPEQEDTIWLHIRSQGQWTNRLYEYFKAADPICLGAKRLTQSLKLRRSQRKSQKEGPS
PVNENHRFCKIKCYLDGPYGTPTRRIFASEHAVLIGAGIGITPFASILQSIMYRHQKRKQHCPNCHYS
WCEDIRDDFLIFKVDFIWINRDQKSFEWFVSLLTKLEMDQAEEEHGGHFLELHMYMTSALSKND
MKAIGLQMALDLLAKKENKDSITGLKTRTQPGRPDWSKVFQKVAAEKKGKVQVFFCGSPALAKVL
KSHCEQLNFKFFKENF

>chicken-Gg-Nox5: DDBJ™ No. BR000278

LLALQAPGTMGTAEDAAWLRWVTERFQSIAGHDEEIGLEEFKAALQVKESFFAERFFALFDVDGSG
TISLAELHGALALLLRGTAADKLRFLFQVYDVDGSGSIDAAELLLVLRACLRESAISLPPQRLHDMA
RVLLEAADQDGNGSITFQELQQQLEAVPGLMESLTISAASWLKPPAPTRHSRRPRCPTSRSWHNHR
GQLAFLGGYVSLNLLLFTLAALRHFGGSGWVAAARGCGQCLNFNCPFIAVMPMLRACLTRLRATPA
GRALPLEHCVALHQPVGSAVLALAVLHAGAHVANYGRLAQDGHGALSEFLLVARPGGGGFGGTAP
QTGLALQLLLFAMLAFSSPCVRRGGHFELFYWSHLSYVPVWALLLFHAPNFWKWFLVPGGLFVLE

KAVGTAVSRAVGLRIVEVHLLPSQVTHLVIQRPRSFRFEPGDYIYLNIPAIAAYEWHPFSISSAPEQQD
TIWLHIRSLGQWTTRLYEFFRQPEPLQPHGNLERKGGGSGCRWVSAGLCFLQAQRLNRAVCRLQG
GLRAVPVGRSAPAARSTQHRLGLSKKGNKNHTGDAAIELTSYRRSGAPTTTRGTEDGDRDWDTSL
QAVGASTAPKPPRRPQGRGGPSLSCDARCPNDFGIEVGVHRSISHHSHWAQGSWESGCGECWKLW
EQGGGGFPLLLMDAPHLQVSVGPGESQQLCSIKVSSGERNGVGAVLSRVLALPRAEHPASVLGLSP
CFWVLSLYLSQCYIDGPYGTPTRRIFTSEHAVLIGAGIGITPFASILQSIMXQVGPYRQRKQSCPSCET
VWDEDMALTKVDFIWINRDQQHFEWFLDLLAALELQQEEQDPGGRFLELHLYMTSALGRSDVKA
VGLQLALDLLAAKEQRDSITGLRTRTQPGRPDWSQVLGRVAEERKGKVHVFFCGSPALAKVVRMH
CERFGFRFFKENF

>frog-Xt-Nox5: Ensembl No. ENSXETP00000017078

TMSTEDDSKWLEWVTKQFENIAGDDKEIDLEEFKTALKVKESFFAERFFALFDSDGSGSISLDELL
KALNLLIHGNETDKLRFLFQVYDVDIWCLCPPTGSRTDSEWNSRHTVHWQCLEPFSFPGSGSIDPS
ELRTVLKSCLRESAISLPEEKLDDLTLVLFESADKDHSGSITFQELKEELERFPEVMENLTISAANW
LKPPAVQNKSHTPRYLTRTYWHNNRSKLLFMCCYWCLNVLLFGLAAVNHASLGGWIMVAKGCGQ
CLNFNCTFIVVMMLRRCLTWLRTTCVVRFLPLDQNVVLHELIGYVIFVLTVIHTAAHVTNFTLINLT
EKTGAYTFWEYLLTIRPGIGWISGTASITGILLQLLICLMLLFSNTFVRKGGYFEVFYWTHLSYIWIW
ILLFLHTPKFWKWFLVPGLLFLLEKLFGAAVSRTGDVYITEVNLLASKVTHLVIKRPPSFQFKPGDY
IYLNIPVIAKYEWHPFTISSAPEQADTIWLHIRSLGQWTNSLYEYFHYPQTVNRHETKRQILTQKNR
QHHSQVSANDMKCSYDMFNRHQFIDRYCYIDGPYGTPTRRIFTSDHAVLIGAGIGITPFASILQSIMY
RYRMRKQNCPSCQYSWCETLKENEMDLRKVDFIWINRDQKFFEWFVSLLTKLELDQADEEPDET
GRFLEMHMYMTSALSKNDMKAIGLQMALDLLAQKEKKDSITGLRTRTQPGRPDWNKVFQKIEQE
NKGKVQVFFCGSPALAKIIKAHCEKFNFKFFKENF

>fugu-Tr-Nox5: DDBJ™ No. BR000279

FFAERFFALFDSDGSSSISLDELLKALDLLIHGSETDKLRFLFQVYDVDGSGSIDPDELRIVLKSCLR
ESAISLPEEKLDDLTLVLFESADKDKSGAITFEELKAELESFPEVMENLTISAANWLKPPDLEQKKQ
NHTPRYLTRAYWHNNCRKLLFLCMYAFFSLMLFVNAMLQHSYGGGWYMVAKGCGQCLNFNCTFI
MVLMLRRCLTWLRATWVVRVLPLDQNILLHQIVGYAILFYTLLHTSAHIFNFVQLSESSGFTLWEYL
LTTRPGIGWVKGTASVTGVVLQFMICLMVLCSSTFVRRSGHFEIFYWSHLSYVWVWILLMVHCANF
WKWFVAPGFVFLLEKIIGIAVSRMGGLYIVEVNLLPSKVTHLVIKRPQFFHFKPGDYVYINIPEIAKY
EWHPFTISSAPEQSDCLWLHIRSMGQWTNRLYEYFRQLDRQTVSTKRLSVTLRKHRQLPKAKVHT
DTQRHLRPVAQLFTLTCVSSPQDEVFSSAKSNKAVAFNEDDAVTLMMYQQRSSGADVVSPAAPESQ
VPPEPLLDELSPAERGEAPPLREVRDHIKGLLGLLFYSNKGFNLSSLYCRFLQSLEIIIGSATSRSKA
QLRNAANKMHLNNFLFKMSFLADEGKQENHSLVSDLSVTYDIEQILLKTSAHTDTEESVKTSDAH
SMTLYRFQCYVDGPYGTPTRQIFASEHAILIGAGIGITPFASILQSIMYKYRRRKQNCPNCNYSWCEN
LKDSDMKLRKVDFIWINRDQKSFEWFVSLLTKLEMDQADEEPEGRFLEMHMYMTSALSKNDMK
AIGLQMALDLLAKKEKRDSITGMRTRTQPGRPDWGKLFQKVSEEKKGKVHVFYCGSPALAKVIKA

QCEHYKFNFYKENF

>tetraodon-Tn-Nox5:Ensembl No. GSTENP00000868001

MLQHRSGAACYMLAKGCGQCLNFNCTFVMVAARAPAGRGLEPRVKRASPTCVQVLMLRRCLTWL
RATWVVRVLPLDQNILLHQIVGYAILFYTLLHTCAHVFNFGTPHTHARTVFPDAEGTCACFPCALQ
RTSESSGFTLWEYLVTTRPGIGWVKGTASLTGVVLQLLIGLMVLCSSTFVRAHGHFEVFYWSHLSY
VWVWALLMVHCANFWKWFVGPGFVFLLEKIVGIAVSRMGGLHIVEVNLLPSKVRWTHGWPVASH
VVPSAMAGRCVCVRPQVTHLVVRRPQFFHFKPERYVYINIPEIAKYEWHPFTISSAPEQSGTCRRRA
RKPPGLTGTNAPVDPLRFLPRTPQLGGVLGGEGDCTLSSPRRLPVAPHPLHGPVDQPPVRVLPGTG
EPDLQQHQEADGEPEKAAAAAEGPGFGQVWRQSSVLQHQVLRRWDYGTPTRQIFASEHAILIGAGI
GITPFASILQSIMYKYRRRKQNCPNCNYSWCENLKDSDMKLRKVDFIWINRDQKSFEWFVSLLTKL
EMDQADEEPEGRFLEMHMYMTSALSKNDMKAIGLQMALDLLAKKEKRDSITGLRTRTQPGRPD
WGKV

>zebrafish-Dr-Nox5:Ensembl No.ENSDARG00000011667

IINVLGSGSIDPDELRTVLKSCLRESAISLPEEKLDDLTLALFESADKDNSGSITFEELKAELETFPE
VMENLTISAANWLKPPDLEQNKRKTPRYLTRAYWHNNSRKLFFLCLYGLLNTFLFIMAMLKHAD
GGLWIMLARGCGQCLNLNCTFVMVLMLRRCLTWLRATWVVRVLPLDQNILLHQIVGYAILIFSVG
HTGAHIMNFARLSQNDGAYQLWEYLFTIRPGIGWVNGTASITGVVIQILIGLMVVCSSTFVRRSGHF
EVFYWSHLSYIWVSALLVVHCANFWKWFVVPGVAFLIEKLVGIAVSRMGGLYIVEVNLLPSKVTHL
VIKRPPFFQFKPGDYVYINIPTIAKYEWHPFTISSAPEQQETLWLHIRSMGQWTNRLYEYFRQPDTQ
TNKRLTASLRSRRHQSRAQLTAKLSENHRYCNIKCYVDGPFGTPTRQIFASEHAILIGAGIGITPFAS
ILQSIMCRYRMRKQNCPNCSYSWCETIKDNEMKLRKVDFIWINRDQKSFEWFVSLLTKLEMDQAD
EEPEGRFLEMHMYMTSALSKNDMKAIGLQMALDLLAKKEKRDSITGLRTRTQPGRPDWAKVFQK
VSEEKKGKVHVFYCGSPALAKVIKAQCERFGFHFYKENF

>rabbit-Oc-Nox5: DDBJ™ No. BR000301

FLELHMYMTSALGKNDMKAIGLQMALDLLAEKEKKDFITGLQTRTQPGRPDWNKVFQKVAAEKK
GKVQVFFCGSPALAKVLRAHCADFRFRFFQENF

>armadillo-Nox5: DDBJ™ No. BR000302

MWLLLVVHGPNFWKWLLVPGLLFVLEKAIGLTASRMAARYIMEVNLLPSKVTHLVIKRPSLFHYRP
GDYVYLNIPTIARYEWHPFTISSAPEQKDTIWLHIRSEGQWTNKLYESFKASDLVGSGSKRLSRSLR
MRRCQSRP

>medaka-Ol-Nox5: Ensembl No. ENSORLP00000017662

KHSQGSNVVLVSSGCSGVSCIMSVDEDARWLEWVTKQFESIAGDDKEINLCEFKTALKVKESFFAE
RFFALFDSDGSGSISLDELLKALDLLIHGTETDKLRFLFQVYDVDACFMMKVTGSGSIDPDELRTVL
KSCLSESAISLPEEKLDDLTLVLFESADTDNSGSITFEELKEELENFPEVMENLTISAANWLKPPDL
DQNKHQTPRYLTRAYWQNNSRKLLFLFGYGVLNLLLFVVAMLRHSDGGVWLMVARGCGQCLNFN
CTFIVVLMLRRCLTWLRATWVVRVLPLDQNILLHQIVGYAIFCFSLGHAIAHVVNFDLTSTNLSQHS

EFLLWEYLLTTRPMIGWVKGTASLTGVVLLLLICLMVLCSSTFVRRSGHFEVFYWSHLSYIWVLILL
IVHCANFWKWFVVPGLLFLLEKIVGIAVSRMGGLYIVEVNLLPSKVTHLVIKRPQFFHFKPGDYIYI
NIPVIAKYEWHPFTISSAPEQSDTLWLHVRSMGQWTNRLYEYFRQTDSMELCSGRLATSLKKRRQQ
AKAEVSAKFAENHRVCNIKCYVDGPYGTPTRQIFTSEHAVLIGAGIGITPFASILQSIMYRYRLRKQN
CPSCNFSWCENLKDSDMTLRKVDFIWINRDQKSFEWFVSLLTKLEMDQADEEPEGRFLEMHMYM
TSALSKNDMKAIGLQMALDLLAKKEKKDSITGLRTRTQPGRPEWGKVFQKLSKENKGKVHVFYC
GAPSLAKAIKAQCERFGFNFYKENF

>sea urchin-Sp-Nox5A: GenBank^{TM} No. XM_001177608

MATARRKSSLAFSSVCGNAQPADEDSQWLSWAEKQFCQIAGEDRQIDEDEFKMALNIKKSFFAERFFHLFD
QDGSGYISLDELMEGLYLLTKGDPVDKLRFLFSVYDVDGNGAIDHEELKVVLRACLCESSMTISEATIDALT
SALFEAADTDGSGAISFEELKEELEKNPDVMENLTISAASWLKPPSLKPSRRVLPRYLTWRYVHNNYRKILFL
VVFILINVALFTEAAYRYAKKKSNWCLITARGCGQCLNFNSAFVLVLMLRKTITTLRTTKAAEILPTDQNIVF
HKLVGIFIALLSGIHTLGHIGNAWFVEKTTDGNVTMSALLFTNPHLTSLGLAPVSGSAFLTGWVLDIILAIMVI
CSMPFVRRSGHFQVFYFTHMLYVVFWGLLLIHGPRFWYWFVVPGIIFIVEKLSQTKCVKQARYGKTYVQEV
NLLPSGVTHLALTRPNRFHYKAGDYIFINIPQIAQYEWHPFTISSAPEQQGTISMHIRSAGNWTNRLYAFFEDR
QKRNRDETELLLGSASDIRVAMETEEVEETNHAGEFIRLREMECDAAEADVVKPTLNHRGANGNLPHGLPR
GYSVEREESEDNQHQHRTIACQTTFEMKGAKSWRSSLREEKIQVFIDGPYGTATRGIFQAEHAILVGAGIGVT
PFASILQSIMHRYRVGRQTCPICQHTWLGNIPTDMMRLKKVDFIWINRNQNAFEWFVSLLTQLEMEQAQEPF
DRFLELHMYMTSAMAKNDMKGIGLQMALDIMHKKGHRDLITGLKTRTQPGRPDWNKIFTQIAREKKGKV
QVFFCGSPTLAKIIKKSCEKFNFSFHKENF

>sea urchin-Sp-Nox5B: GenBank^{TM} No. XM_780967

MAGMQEKDEKWLVKLEKHFQEVAGDDNLIDLDEFINALNVKKSFFAERFFELIDTDQSGSISLKELIGALRL
LVNGTEQEKLHFLFQVYDVDGSGFIDFDELKTVLRSCTAESAMTLCDETLTELTEILFDDADVDGDGEVSFE
ELSEQLQRYPGITSNLTISYNTLRNHFSVIIFWIVFVMINAGLAAWGAYEGYQSVSDERHPAAISIARSAGRCL
SFECCFVLVLMLRKLLTILRNTFLMSVLPLDQHVVIHKIVAVFIIILSVIHTAGHIANIGLIYQVENVNGTTAA
WILDVLVRPFPGLGLVEGSCIITGILIIIVLIIMTICSLPFIRRNGYFKVFYWTHQLCIVFWCLIIIHSKYFWIWFIA
PGIIYLAERLVRLQFFRRARFGKVYIQKGYVLPANVVQLVIQRPAKFKFHAGEYIHVNIPSIASHEWHPFTISS
APEQQEYLTLHIRCVGHWTKRLYDVVRERELTLLEHENAGFGEIDKDHDEPLEVIVDVSSTKSTTAVEPNGQ
QSIISDTHSNRNGRRSSQTYSKRRRTASGAINSGFEPELPNGDKDGTKTVTEGKPYNTGQNDNESVTQKVNA
AQLSLPRKADHPSASQGDCQPMSYKELGEVSMTLEVNGDSFKMKSLNSRQSIDVQEAPRGSTTKRQSLRVR
QSLVGREKRSVCGKPNGDVNRKMSLVTLRRNGARHSLDLSKDLGGRPHTGLEVILDGPYGAPAQHIMEAE
HAVLIGAGIGITPFASILQSINERYKAARKHCPNCNHTWVTDSSSILKTKKVDFVWINRDQHSFEWFISLISAIE
LEQAEIPAADRFLDIHLYMTSALSPSDMKAIGLHVALDLIHKKKKRDTITGLKTRTQAGRPDWDEVFQNLKQ
QHKGKITVFFCGSPALGKVLSTKCLQYQMEFRKENF

>fruit fly-Dm-Nox5: FlyBase No. CG3896

MDFAEQIESVAYVICGENKRVSFKNFRDIWHTRGILDKLYRLIELDGSNLVSTNQVMEFISHLTNSR

PRTGFDKSSLARLEQLFRTTVGNEQEIRREEFQKIVTSKNPFFTERVFQIFDKDNSGSISLQEFIDAI
HQFSGQSADDKIRFLFKVYDIDGDGLIQHKELHDVIRHCIKENGMEFSEDQIEDLTSAMFEDADPH
NSGEITYEALKNQLHKHGGLLENLSITIDRWLVPIAEDRQAGGAAKSGFWNSLPHQFSLAYMKNN
QVFVTYLFFYITVNLCLFISRAIQYRASNGFVIIARACGQCLNFNCAWVLVLMLRHSLTYLRGRGLSS
YLPLDHHVYLHKLTGITISVLSLIHTIMHLFNFSIIVINDPNINAGHYTIGEWLLTDRPGLFGLIPGCA
NPTGVALLAILVVMFVCSQPFVRRKGSFEVFYWTHLLYVPFWILCLFHGPNFWKWFLLPGLVYIVE
RALRFIWMRGEHGKTYISSGLLLPSKVVHLVIKRPHHFNFRPGDYVFVNIPAIANYEWHPFTISSAP
EQEDYMWLHIRTVGEWTNRLYRYFEREQQKLQQSGSSQEIPQHMHAIPTPSFMLLNEARNPAIAG
ERSATPQTDFLAKNLGVQAVPPVRPPRQNRKPAPGAPIDPPATGVNRIRSIKKTLQRTFSRKEAVDP
KKGIPNGAFIADGEREDSNLKQRPLEKSISLPDISVKSKKRSRLKALRALGRSESESAFDEKRVRRA
RNNSVGLAYLSPQNKSLAQSFRYMRTKPTIIAFKTPSMEEREHQVAAGEANGASPASRAEQGQLSS
RMDSADKLQLARLSLSAEGASKPLEDQTQTGSPSRKSILRRPTFLRSLSASINNRTGGGGGGSTGSS
TTNSGGKVTLDAGVMEIFIDGPYGAPSSHIFGAQHAVLIGTGIGVTPFASILQSIMHRYWKARHSCP
RCQFEWASEIPKSVMNLRKVDFFWINRDQRSFEWFVNLLSQLEIEQAELGGAMERFLDMHMYITS
ALQRTDMKAVGLQLALDLLHEKGKRDLITGLKTRTNAGRPNWDKVFKQLQAQQKGKVTVFYCGP
PQLAKTLRYKCDQYGFAFRKECF

>mosquito-Ag-Nox5: DDBJ™ No. BR000280

IDFAEQLESVAYVLCGDGTVTYDKFCQIWHAKGILDKLYRLIDVDCTNLISTNQIMEFISNLTNSRPR
TGFDKSSLERLEQLFIKTVGNEKEIRREEFKKIVTSKNPFFTERVFQIFDKDNSGSISLQEFIDAIHQ
FAGQSPEDKIKFLFKVYDLDGDGLIQHRELQHVMRACMEENGMRFSEDQIEDLTMAMFEDADKY
NRGAITYEALKNQLEKHGGLLENLSISIDRWLVPLPQEDTKKKRKKKPLPHQLTAPYIKNNYVYLS
FLTVFTLINVGLFVSRAIQYRNSNGFVIMARACGQCLNFNCAFILVLMLRQCITFLRTRGFTAFLPLD
QHIYLHKLTGVLVAIFSLVHTIMHLFNFTTIVVYDPVLNANNYTTAEWLFTARPGLFGLIGGCANPT
GVVLVLILLIMFICSQPFVRRGGSFEVFYWTHLLYVPFWILVLFHGPNFWKWFIVPGLIYLVERTIRL
VWMRTEHGKTYISSGLLLPSKVTHLVIKRPLHFCFRPGDYVFVNIPAIAQYEWHPFTLSSAPEQEDY
IWLHIRGVGEWTNRLHNFFEREQERLHNGEIPALVAGRVGGAGGPVGPRAGDATTPAGIMKQRHP
PGTSKLAMEGYSAPSPVATSTAQPAKFERQMSDNRAFKKIQATLQRTFSRRDQLIPRSGGAGGVGGI
ANEGFSGDGQKVPLEKSLSMPDMQNKFKKRERMMVLREYMRSESERSFDEVQIRKARLQSLGLA
YLSPQNKSLAQSFRYMRNKPTIIAFKTPSLENCEPRDSTNSIVVSPGVFTQKDAEEGRTAGALPTSG
AASSVVAVGSGAASAASSNPASRPVNYPVGKPLEIYIDGPYGAPSSHIFQAQHAILIATGIGVTPFASIL
QSIMHRYWKARHCCPRCSYEWSSEIPPTIMNLRKVDFFWINRDQRSFEWFVNLLSQLEIEQAELGS
AMERFLEMHMYITSALQKTDMKAVGLQLALDLLHEKEKRDLITGLKTRTNAGRPNWDKVFKQIQ
DQKKGKVTVFYCGPPQLAKTLRYKCDQFGFQFRKEVF

>honeybee-Am-Nox5: DDBJ™ No. BR000281

DLIIQFENVAYSICCDNPVTFEKFQQIFSTKEIIDKLFRRIDEENLGYITSFQIMEFLSNISDTRPLAGF
DKRSLEWLEKIFKQTVGNEKEIRREEFNKIVTSKNPFFTDRVFQIFDKDNSGTISLQEFVDAMHQF

AGKSPDDKIKFLFKVYDIDGDGLIQLRELEHVMRACLEENGIRFSEEQIEELTMALFDDADQSNRG
AITFEALKKQLEKHEGLLENLSISIDRWLVPPKPESKRKSRLQLLASLRPYQLTKPYMKNNYVYIFF
ISIFILINVSLFVSRLYEYRKSNGYVMLARACGQCLNFNCSFILVLMLRQCITFLRTHGFNSVLPLDQ
HIYLHKVTGGLICVFSIAHTLMHLLNFGTIVIYDEILNHNNYTLSEWLLTSRPALFGLVRGYANPTGF
ILVILFLIIMICSMPFVRRGGCFEIFYWSHLLYIPYWILVILHAPNFWKWFIGPGLIYLLERIRRIAWSR
SQLGKTYISSGLLLPSKVTHLVIKRPPHFVFHPGDYVFVNIPVIARYEWHPFTISSAPEQEDYIWLHI
RAVGEWTNSLYSYFEKEQMKLQRDNIFPIENRNNPNVSESVSMNGKFLSPIPRGNRRSFDNSVFVS
DDEKITQSPQSVIGNLFKFIQNYLSITDVKKMSNNRKLHSLLVSKMPLEKSVSVPDMLPGKKKNDQ
LIATQGYRRKQSNNLSTIQSAAEEGRLQMKFEENSKKNHNDIEFDSISHSLNYTVGKPLEIFLDGPY
GAPSSHIFQAQHAVLIATGIGVTPFASILQSIMHRYWKARHTCPKCKFSWASEIPPTVMHLRKVDFF
WINRDQQSFEWFVNLLSQLEMEQAELGDAMERFLEMHMYITSALQKSDMKAVTLQLAMDLVHQ
MEKRDLITGLKTRTNAGRPNWDKVFKHLQDQKKGKVTIFYCGPPQLARILRYKCDQFGFNFRKES
F

>mouse-Mm-Duox1: GenBank™ No. XM_130483

MGFHLALAWILLVGTLASLGAQNSISWEVQRFDGWYNNLMEHRWGSKGSRLQRLVPASYADGVY
QPLKEPYLPNPRHLSNRVMRGSAGQPSLRNRTVLGVFFGYHVLSDLVSVETPGCPAEFLNIYIPHG
DPVFDPDKRGNVVLPFQRSRWDRNTGQSPSNPRDQSNQVTGWLDGSAIYGSSHSWSDTLRSFSGG
QLASGPDPAFPSDSQSSLLMWMAPDPSTGQGGPRGVYAFGAQRGNREPFLQALGLLWFRYHNLCA
RKLAQEHPHWGDEELFQHARKRVIATYQNIAMYEWLPSFLKQTPPEYPGYRPFLDPSISPEFVVAS
EQFLSTMVPSGVYMRNASCHFQGIPSHNSSVSGALRVCNSYWSREHPKLQRAEDVDALLLGMASQ
IAEREDHVVVEDMQDFWPGPLKFSRTDYLASCLQRGRDLGLPSYTKAREALGLSPISHWQDINPAL
SRSNGTVLEATAALYNQDLSRLELLPGGLLESHGDPGPLFSTIVLDQFVRLRDGDRYWFENTRNGL
FSKEEIAEIRNTSLRDILVAVTNVDPSALQPNVFFWLAGDPCPQPSQLSAKGLPACAPLFIRDYFEGS
GFGFGLTIGTLCCFPLVSLLSAWIVARLRKRNFKRLQRQDRQSIMSEKLVGGVEALEWQGRNEPCR
PVLVHLQPGQIRVVDGRLTVLRTIQLRPPQQVNLILSSNRGRRTLLLKIPKEYDLVLLFNMEEERQA
LVENVRGALKENGLSFQEWELREQELMRAAVTRQQRGHLLETFFRHLFSQVLDINQADAGTLPLD
SSTKVREALTCELSRAEFADSLGLKPQDMFVESMFSLADKDGNGYLSFREFLDILVVFMKGSPEEK
SRLMFRMYDFDGNGLISKDEFIRMLRSFIEISNNCLSKAQLAEVVESMFRESGFQDKEELTWEDFH
FMLRDHDSDLRFTQLCVKGVEVPEVIKNLCRRASYISQEKICPSPRMSAHCARNNMKTASSPQRLQ
CPMDTDPPQEIRRRFGKKVTSFQPLLFTEAHREKFQRSRRHQTVQQFKRFIENYRRHIGCVAVFYTI
TGALFLERAYYYAFAAHHSGITDTTRVGIILSRGTAASISFMFSYILLTMCRNLITFLRETFLNRYIPFD
AAVDFHRLIASTAIILTVLHSAGHVVNVYLFSISPLSVLSCLFPGLFHDDGSEFPQKYYWWFFQTVP
GLTGVLLLLALAIMYVFASHHFRRRSFRGFWLTHHLYIFLYILLIIHGSFALIQMPRFHIFFLVPAIIYV
GDKLVSLSRKKVEISVVKAELLPSGVTHLRFQRPQGFEYKSGQWVRIACLALGTTEYHPFTLTSAP
HEDTLSLHIRAAGPWTTRLREIYSPPTGDTCARYPKLYLDGPFGEGHQEWHKFEVSVLVGGGIGVT
PFASILKDLVFKSSVSCQVFCKKIYFIWVTRTQRQFEWLADIIREVEENDRQDLVSVHIYITQLAEKF

DLRTTMLYICERHFQKVLNRSLFTGLRSITHFGRPPFEPFFNSLQEVHPQVRKIGVFSCGPPGMTK
NVEKACQLINRQDRTHFSHHYENF

>mouse-Mm-Duox2: GenBank™ No. XM_917937

MLPTSPKTLVLLGALLTGPLGPAGGQDAPSLPWEVQRYDGWFNNLKYHQRGAAGSRLRRLIPANY
ADGVYQALEEPLLPNPRRLSDAVAKGKAGLPSVHNRTVLGVFFGYHVLSDLVSVETPGCPAEFLNI
YIPRGDPVFDPDKRGNVVLPFQRSRWDRNTGQSPSNPRDQSNQVTGWLDGSAIYGSSHSWSDTLR
SFSGGQLASGPDPAFPRNSQSSLLMWMAPDPSTGQGGPQGVYAFGAQRGNREPFLQALGLLWFRY
HNLCARKLAQEHPHWGDEELFQHARKRVIATYQNIALYQWLPSFLQKTPPEYSGYRPFMDPSISPE
FVVASEQFLSTMVPPGVYMRNSSCHFRKFPKEGSDSSPALRVCNSYWIRESPNLKTAQDVDQLLLG
MASQISELEDRIVIEDLRDYWPGPERFSRTDYVASSIQRGRDMGLPSYSQALLALGLEPPKNWSAL
NPQVEPQVLEATAALYNQDLSQLELLLGGLLESHGDPGPLFSNIILDQFVRLRDGDRYWFENTRNG
LFSKEEIAEIRNTTLRDVLVAVSNVDPSALQPNVFFWQEGAPCPQPRQLTTDGLPQCAPVTVIDYFE
GSGAGYGVTLVAVCCFPLVSLIVAGVVAHFRNRERKMLLKKGKESLKKQPASDGVPAMEWPGPKE
KSYPVTLQLLPDRSLQVLDKRFTVLRTIQLQSPQQVNLILSSNSGRRTLLLKIPKEYDLVLMFNSEE
DRGAFVRLLQDLCICCTPGLHIAEVDEKELLRKAVTKQQRAGILEIFFRQLFAQVLDINQADAGTLP
LDSSQQVREALTCELSRAEFADSLGLKPQDMFVESMFSLADKDGNGYISFREFLDILVVFMKGSSE
DKSRLMFTMYDLDGNGFLSKDEFFTMMRSFIEISNNCLSKAQLAEVVESMFRESGFQDKEELTWE
DFHFMLRDHDSDLRFTQLCVKGGAGGTKDIFKQSSACRVSFINRTPGNRVMGPSPRLYTEALQEK
KQSGFLAQKFKQYKRFVENYRRHIVCVTIFSAICIGLFADRAYYYGFASPPTDIEETTYVGIILSRGTA
ASISFMFSYILLTMCRNLITFLRETFLNRYIPFDAAVDFHRWIAMAAVVLAVLHSAGHAVNVYIFSVS
PLSLMACVFPNVFVNDGSKFPPKYYWWFFETVPGMTGVLLLLVLAIMYVFASHHFRRHSFRGFWL
THHLYVVLYVLIIIHGSYALIQLPSFHIYFLVPAIIYGGDKLVSLSRKKVEISVVKAELLPSGVTYLQFQ
RPKTFEYKSGQWVRIACLDLGTNEYHPFTLTSAPHEDTLSLHIRAVGPWTTRLREIYSPPVGGTCAR
YPKLYLDGPFGEGHQEWHKFEVSVLVGGGIGVTPFASILKDLVFKSSMGSQMLCKKIYFIWVTRTQ
RQFEWLADIIREVEENDRQDLVSVHIYITQLAEKFDLRTTMLYICERHFQKALNRSLFTGLRSITHF
GRPPFELFFNSLQEVHPQVRKIGVFSCGPPGMTKNVEKACQLINRQDRAHFVHHYENF

>human-Hs-Duox1: GenBank™ No. NM_017434

MGFCLALAWTLLVGAWTPLGAQNPISWEVQRFDGWYNNLMEHRWGSKGSRLQRLVPASYADGVY
QPLGEPHLPNPRDLSNTISRGPAGLASLRNRTVLGVFFGYHVLSDLVSVETPGCPAEFLNIRIPPGD
PMFDPDQRGDVVLPFQRSRWDPETGRSPSNPRDPANQVTGWLDGSAIYGSSHSWSDALRSFSRGQ
LASGPDPAFPRDSQNPLLMWAAPDPATGQNGPRGLYAFGAERGNREPFLQALGLLWFRYHNLWAQ
RLARQHPDWEDEELFQHARKRVIATYQNIAVYEWLPSFLQKTLPEYTGYRPFLDPSISSEFVAASEQ
FLSTMVPPGVYMRNASCHFQGVINRNSSVSRALRVCNSYWSREHPSLQSAEDVDALLLGMASQIA
EREDHVLVEDVRDFWPGPLKFSRTDHLASCLQRGRDLGLPSYTKARAALGLSPITRWQDINPALSR
SNDTVLEATAALYNQDLSWLELLPGGLLESHRDPGPLFSTIVLEQFVRLRDGDRYWFENTRNGLFS
KKEIEEIRNTTLQDVLVAVINIDPSALQPNVFVWHKGDPCPQPRQLSTEGLPACAPSVVRDYFEGSG

FGFGVTIGTLCCFPLVSLLSAWIVARLRMRNFKRLQGQDRQSIVSEKLVGGMEALEWQGHKEPCRP
VLVYLQPGQIRVVDGRLTVLRTIQLQPPQKVNFVLSSNRGRRTLLLKIPKEYDLVLLFNLEEERQAL
VENLRGALKESGLSIQEWELREQELMRAAVTREQRRHLLETFFRHLFSQVLDINQADAGTLPLDSS
QKVREALTCELSRAEFAESLGLKPQDMFVESMFSLADKDGNGYLSFREFLDILVVFMKGSPEEKSR
LMFRMYDFDGNGLISKDEFIRMLRSFIEISNNCLSKAQLAEVVESMFRESGFQDKEELTWEDFHF
MLRDHNSELRFTQLCVKGVEVPEVIKDLCRRASYISQDMICPSPRVSARCSRSDIETELTPQRLQCP
MDTDPPQEIRRRFGKKVTSFQPLLFTEAHREKFQRSCLHQTVQQFKRFIENYRRHIGCVAVFYAIAG
GLFLERAYYYAFAAHHTGITDTTRVGIILSRGTAASISFMFSYILLTMCRNLITFLRETFLNRYVPFDA
AVDFHRLIASTAIPVAVLHSVGHVVNVYLFSISPLSVLSCLFPGLFHDDGSELPQKYYWWFFQTVPG
LTGVVLLLILAIMYVFASHHFRRRSFRGFWLTHHLYILLYVLLIIHGSFALIQLPRFHIFFLVPAIIYGG
DKLVSLSRKKVEISVVKAELLPSGVTHLRFQRPQGFEYKSGQWVRIACLALGTTEYHPFTLTSAPHE
DTLSLHIRAAGPWTTRLREIYSAPTGDRCARYPKLYLDGPFGEGHQEWHKFEVSVLVGGGIGVTPF
ASILKDLVFKSSVSCQVFCKKIYFIWVTRTQRQFEWLADIIREVEENDHQDLVSVHIYITQLAEKFDL
RTTMLYICERHFQKVLNRSLFTGLRSITHFGRPPFEPFFNSLQEVHPQVRKIGVFSCGPPGMTKNV
EKACQLINRQDRTHFSHHYENF

>human-Hs-Duox2: GenBank™ No. NM_014080

MLRARPEALMLLGALLTGSLGPSGNQDALSLPWEVQRYDGWFNNLRHHERGAVGCRLQRRVPAN
YADGVYQALEEPQLPNPRRLSNAATRGIAGLPSLHNRTVLGVFFGYHVLSDVVSVETPGCPAEFLNI
RIPPGDPVFDPDQRGDVVLPFQRSRWDPETGRSPSNPRDLANQVTGWLDGSAIYGSSHSWSDALR
SFSGGQLASGPDPAFPRDSQNPLLMWAAPDPATGQNGPRGLYAFGAERGNREPFLQALGLLWFRY
HNLWAQRLARQHPDWEDEELFQHARKRVIATYQNIAVYEWLPSFLQKTLPEYTGYRPFLDPSISPE
FVVASEQFFSTMVPPGVYMRNASCHFRKVLNKGFQSSQALRVCNNYWIRENPNLNSTQEVNELLL
GMASQISELEDNIVVEDLRDYWPGPGKFSRTDYVASSIQRGRDMGLPSYSQALLAFGLDIPRNWSD
LNPNVDPQVLEATAALYNQDLSQLELLLGGLLESHGDPGPLFSAIVLDQFVRLRDGDRYWFENTR
NGLFSKKEIEDIRNTTLRDVLVAVINIDPSALQPNVFVWHKGAPCPQPKQLTTDGLPQCAPLTVLDF
FEGSSPGFAITIIALCCLPLVSLLLSGVVAYFRGREHKKLQKKLKESVKKEAAKDGVPAMEWPGPKE
RSSPIIIQLLSDRCLQVLNRHLTVLRVVQLQPLQQVNLILSNNRGCRTLLLKIPKEYDLVLLFSSEEE
RGAFVQQLWDFCVRWALGLHVAEMSEKELFRKAVTKQQRERILEIFFRHLFAQVLDINQADAGTLP
LDSSQKVREALTCELSRAEFAESLGLKPQDMFVESMFSLADKDGNGYLSFREFLDILVVFMKGSPE
DKSRLMFTMYDLDENGFLSKDEFFTMMRSFIEISNNCLSKAQLAEVVESMFRESGFQDKEELTWE
DFHFMLRDHDSELRFTQLCVKGGGGGGNGIRDIFKQNISCRVSFITRTPGERSHPQGLGPPAPEAP
ELGGPGLKKRFGKKAAVPTPRLYTEALQEKMQRGFLAQKLQQYKRFVENYRRHIVCVAIFSAICVG
VFADRAYYYGFASPPSDIAQTTLVGIILSRGTAASVSFMFSYILLTMCRNLITFLRETFLNRYVPFDAA
VDFHRWIAMAAVVLAILHSAGHAVNVYIFSVSPLSLLACIFPNVFVNDGSKLPQKFYWWFFQTVPG
MTGVLLLLVLAIMYVFASHHFRRRSFRGFWLTHHLYILLYALLIIHGSYALIQLPTFHIYFLVPAIIYGG
DKLVSLSRKKVEISVVKAELLPSGVTYLQFQRPQGFEYKSGQWVRIACLALGTTEYHPFTLTSAPHE

DTLSLHIRAVGPWTTRLREIYSSPKGNGCAGYPKLYLDGPFGEGHQEWHKFEVSVLVGGGIGVTPF
ASILKDLVFKSSLGSQMLCKKIYFIWVTRTQRQFEWLADIIQEVEENDHQDLVSVHIYVTQLAEKFD
LRTTMLYICERHFQKVLNRSLFTGLRSITHFGRPPFEPFFNSLQEVHPQVRKIGVFSCGPPGMTKN
VEKACQLVNRQDRAHFMHHYENF

>chicken-Gg-Duox : GenBank™ No. XM_425053

EVQRYDGWYNNLQHRSRGSVGSRLLRLLPANYADGVYQALQEPHVPNARQLSNAVARGPSGLPSK
RNTTVLAVFFGFHVLSDILGTEKPGCPAEFLNIHIPEGDPVFDPAGTGDIVLPFQRIRWALETGQSPN
SPREQTNEVTGWLDGSSIYGPSHSWSDALRNFSNGQLASGPGGHVPRETDGKVPMWKALDPSTG
QGGLRGIYDLGNAWGNENRFLQAMSIAWFRYHNYLAAELAKDHPSWSDEDIFQHARKRVIATFQS
IVLYEWLPALLGTPVQKYTGYQQHMDPSISPEFVAAARIFLATTVPPGVYKRDPRCHVRNVSSSGGP
FPAMRLCNSYWSRESIEMQQEDVDDLLLGMSSQIAEREDSIVVEDLQDYWYGPLKYSRADYVASW
LQRGRDLGLPTYNQARERFGLEPLQDWTNLAPHSQQKVLEVAALYANNTARLELLPGGMLEGDSP
LFSAIILDQFVRLRDGDRFWFENTKNGLFTAKEAEEIRNTTFRDILAAVTYSQPAELQSNVFVWSKG
DPCPQPQQLTAQLLANCTPMTVLDYFEGSGAGFGIIIIVLCCLPLGLPLQFVIHLLFYQRKHRKGAL
WYLMPLSPMCSQYMFPMLCYFPSAMEWHGPKTDSSLVYIQFQADKVLKVLDGRGLMLRSVSLNA
QQSVEVILSSNKGNKALLLKSPKEYDLVLFFSEEAERSSFFRKLQDYLKTSCLELHVSEMKEQSLL
KRAVTQEQRKQILETFFRHLFARMLDIDKTDAGELNFESSQKARESLTCELSRAEFAEALGLKVNS
MFVDSMFSLADKDGNGYISFREFLDILVVFMKGSSEEKSKLMFRMYDIDENGFLSKEEFLRMLRY
FSSAQQTFSQRLQLQVSAQAAHCFSSSVPKEATWKPMGSTLTTRSPFLLANQYQLHLYTEAQRKKY
QQNKVKQKIQEFKRFIENYRRHIVCVVLFSAITAGLFVERAYYYAFASPSTGIAQTTFVGIIISRGSAA
CISFMYSYILLTMCRNLITVLRETFLNRYIPFDAAVDFHRWIAMAALIFSVLHTAGHLVNVYIFSVTP
LSVLSCLFSSVFMNDGPDPFPILPFTGMTGVLLLIILAVMYVFATHHFRRVSFQAFWITHHLYVLLYV
LVIIHGSYALIQQPRFHIYFIIPALIYGADKLLSLSRKKVEISVVKAELLPSGVTHLRFQRPQDFDYKS
GQWVRIACMALGTTEYHPFTLTSAPHEDTLSLHIRAVGPWTTRLRELYSPESLALIGKLPKLYLDGP
FGEGHQEWHKFEVSVLVGGGIGVTPFASILKDLVFKSSINSKLMCKKIYFIWVTRTQRQFEWLADII
REVEETDRNELVSVHIYITQLAEKFDLRTTMLYICERHFQKVLNKSLFTGLRSITHFGRPPFIPFFDS
LQEVHPEVHKIGVFSCGPPGMTKSVEKACQQLNKKDQAYFAHQ

>dog-Cf-Duox1: GenBank™ No. NM_001003122

MGFCLALTWTFLVGSWTSMGAQKPISWEVQRFDGWYNNLMEHKWGSKGSRLQRLVPASYADGVY
QPLGEPHLPNPRDLSNAAMRGPAGQASLRNRTVLGVFFGYHVLSDLVSVETPGCPAEFLNIRIPPG
DPVFDPNGRGDVVLPFQRSRWDPESGQSPSNPRDLTNAVTGWLDGSAIYGSSHSWSDALRSFSGG
QLASGPDPAFPRNAQPPLLMWSAPDPASGQRGPGGLYAFGAERGNRDPFLQALGLLWFRYHNLCA
QRLARQHPHWGDEELFQHARKRVIATYQNIALYEWLPSFLQQAPVKYAGYNPFLDPSISPEFLVASE
QFFSTMVPPGIYMRNASCHFQEVINRNSSISRALRVCNSYWSRKHPNLRRAEDVDALLLGMASQIA
EREDHVVVEDVLDFWPGSLKFSRTDHVAGCLQRGRDLGLPSYTKARAALGLPPITRWQDINPALSQ
NNHTVLEATAALYNQDLSQLELLPGGLLESHGDPGPLFSAIVLNQFVRLRDGDRYWFENTRNGLF

SEEEIAEIRNTSLRDVLVAVTNMNPSTLQPNVFFWHMGDPCPQPRQLSTQGLPACAPSTMQDYFEG
SGFGFGVTIGTLCCFPLVSLLSAWIVARLRKKNFKKLQGQDRKSVMSEKLVGGMEALEWQGHKEP
CRPVLVHLQPGQICVVDGRLSVLRTIQLRPPQQVNLILSGNRGRRALLLKIPKEYDLVLLFNLEEER
QVLVENLRGALKESGLKFQEWELREQELMRTAVTRQQRSHLLETFFRHLFSQVLDIDQADAGTLP
LDSSQKVQEALTCELSRAEFAESLGLKPQDMFVESMFSLADKDGNGYLSFREFLDILVVFMKGSPE
EKSRLMFRMYDFDGNGLISKDEFIRMLRSFIEISNNCLSKAQLTEVVESMFRESGFQDKEELTWED
FHFMLRDHDSELRFTQLCVRGVEVPEVIKDLCRRASYISQEKICPSPRVSARCPHSNTEVEWTPQR
LQCPVDTDPPQEIRRRFGKKVTSFQPLLFTEAQREKFQRSRRHQTLQQFKRFIENYRRHIGCVAVFY
AITGGLFLERAYYYAFGAHHMGITDTTRVGIILSRGTAASISFMFSYILLTMCRNLITFLRETFLNRYV
PFDAAVDFHRLIASTAIVLTGRALDAGHVVNVYLFSISPLSVLSCLFPGLFHNDGSEFPQKYYWWFF
QTVPGLTGVMLLLVLAIMYVFASHHFRRHSFRGFWLTHHLYILLYVLLIIHGSFGLIQLPRFHIFFLV
PALIYVGDKLVSLSRKKVEISVVKAELLPSGVTHLQFQRPQGFEYKSGQWVQIACLALGTTEYHPFT
LTSAPHEDTLSLHIRAAGPWTTRLREIYSPPTGDGCAKYPKLYLDGPFGEGHQEWHKFEVSVLVGG
GIGVTPFASILKDLVFKSSVSCQVFCKKIYFIWVTRTQRQFEWLADIIREVEENDCQDLVSVHIYITQ
LAEKFDLRTTMLYICERHFQKVLNRSLFTGLRSITHFGRPPFEPFFKSLQEVHPQVRKIGVFSCGPP
GMTKNVEKACQLINRQDRTHFSHHYENF

>dog-Cf-Duox2: GenBank™ No. XM_535461

AFGAERGNRDPFQALGLLWFRYHNLCAQRLRQHPHWGDEELFQHARRVIATYQNIALYEWLPSFL
QKTLPKYTGYHPFLDPSISPEFLVASEQFFSTMVPPGVYMRNASCHFQMVLTKDLGSSPALRVCNS
YWLRENANLNSAQAVDQLLLGMASQISELEDRIVVEDLRDYWPGSGKFSRTDYVASSIQRGRDMG
LPSYTQALMALGLEIPRNWSHINPNVDAQVLEATAALYNQDLTRLELLPGGLLESHGDPGSLFSAII
LDQFVRLRDGDRYWFENTRNGLFSKEEIAEIKNTTFWDVLVAVTNVNHSALQPNVFIWHDGAPCP
QPRQLTTQGLPQCVPLTVFDYFEGSGPGFGITIVALCCLPLVSLLISGVVAHFRGRERKRLQKKGKE
SVKKEAAKDGVPAMEWPGPREKSYPITIQLLPDRRLQVLDRKLSVLRTVQLQPPQQVNLILSSNRG
CHTLLLKIPKEYDLVLLFNSEEERGTFVQHLQDFCVQWSLGLDVAEMRENELFRRAVTKQQRGRIL
EIFFRYLFAQVLDIDQADAGTLPLNSSQKVREALTCELSRAEFAESLGLKPQDMFVESMFSLADKD
GNGYLSFREFLDILVVFMKGSPEDKSRLMFTMYDLDANGFLSKDEFFTMMRSFIEISNNCLSKAQL
TEVVESMFRESGFQDKEELTWEDFHFMLRDHDSELRRTQLCVRGVGDIFKPNISCRVSFITRTPGK
RSCSQDLEFSASEAPELGGPGLKKRFGKKVVGPTPRLYTEALKEKMQRGLLAQKLRQYKRFVENY
RRHIVCVVVFSAICAGLFAERAYYYAFASPPSGIAETTFVGIILSRGTAASISFMFSYILLTMCRNLITF
LRETFLNRYVPFDAAVDFHRWIAMAAVVLAILHSAGHAVNVFIFSVSPLSLLACIFPNIFMNDGSQL
PQKFYWWFFQTVPGMTGVLLLLVLAIMYVFASHHFRRRSFRGFWLTHHLYIVLYVLLIIHGSFGLIQ
LPRFYIYFLVPALIYVGDKLVSLSRKKVEISVVKAELLPSGVTHLQFQRPQGFEYKSGQWVRIACLAL
GTNEYHPFTLTSAPHEDTLSLHIRAVGPWTTRLRETYSLPKGDGCARYPKLYLDGPFGEGHQEWH
KFEVSVLVGGGIGVTPFASILKDLVFKSSLGSQMLCKKIYFIWVTRTQRQFEWLADIIREVEENDHQ
DLVSVHIYITQLAEKFDLRTTMLYICERHFQKVLNRSLFTGLRSITHFGRPPFEPFFKSLQEVHPQV

PKIGVFSCGPRNDQECREGLSAHQQAGPGPLR

>rat-Rn-Duox1: GenBank™ No. NM_153739

MAVYSAVAWILLFGMLASLGAQNSVSWEVQRFDGWYNNLMEHRWGSKGSRLQRLVPASYADGVY
QPLREPYLPNPRHLSNRVMRGPAGQPSLRNRTVLGVFFGYHVLSDLVSVETPGCPAEFLNIYIPRGD
PVFDPDKRGNVVLPFQRSRWDRSTGQSPSNPRDLVTGWLDGSAIYGSSHSWSDXLRSXXGGQLAS
GPDPAFPRNSQNSLLMWMAPDPATGQGPQGLYAFGAQRGNRXXFLQALGLLWFRYHNLCAKRLA
QEHPHWGDEELFQHARKRVIATYQNIAMYEWLPSFLKQTPPEYPGYHPFLDPSISPEFVVASEQFL
STMVPPGVYMRNASCHFQGIANRNSSVSGALRVCNSYWSRENPKLQRAEDVDALLLGMASQIAER
EDHLVVEDVQDFWPGPLKFSRTDYLASCLQRGRDLGLPSYTKAREALGLPPVSHWQDINPALSRS
NGTVLEATAALYNQDLSRLELLAGGLLESHGDPGPLFSAIVLDQFVRLRDGDRYWFENNRNGLFS
KEEIAEIRNTSLRDILVAVTNVDPSALQPSVFFWLAGDPCPQPSQLSTQGLPACAPLFVRDYFKGSGF
GFGLTIGTLCCFPLVSLLSAWIVARLRMRNFKRLQRQDRQSIMCEKLVGGVEALEWQGRKEPCRPV
LVHLQPGQIRVVDGRLTVLRTIQLRPPQQVNLILSSNRGRRTLLLKIPKEYDLVLLFNMEEERQALV
ENIRAALKENGLSFQEWELREQELMRAAVTRQQRGHLLETFFRHLFSQVLDINQADAGTLPLDSST
KVREALTCELSRAEFADSLGLKPQDMFVESMFSLADKDGNGYLSFREFLDILVVFMKGSPEEKSRL
MFRMYDFDGNGLISKDEFIRMLRSFIEISNNCLSKDQLAEVVESMFWESGFQDKEELTWEDFHFM
LRDHDSDLRFTQLCVKGVEVPEVIKNLCRRASYISQEKICPSPRMSAHCARNNTKTASSPQRLQCP
VDTDPPQEIRRRFGKKVTSFQPLLFTEAHREKFQRSRRHQTVQQFKRFIENYRRHIGCVAVFYTITG
ALFLERAYYYAFAAHHSGITDTTRVGIILSRGTAASISFMFSYILLTMCRNLITFLRETFLNRYIPFDAA
VDFHRFIASTAIILTVLHSAGHVVNVYLFSISPLSVLSCLFPDLFHDDGSEFPQKYYWWFFQTVPGL
TGVLLLLALAIMYVFASHHFRRRSFRGFWLTHHLYIFLYILLIIHGSFALIQMPRFHIFFLVPAIIYVGD
KLVSLSRKKVEISVVKAELLPSGVTHLRFQRPQGFEYKSGQWVRIACLALGTTEYHPFTLTSAPHED
TLSLHIRAAGPWTTRLREIYSPPTGDTCARYPKLYLDGPFGEGHQEWHKFEVSVLVGAGIGVTPFAS
ILKDLVFKSSVSCQVFCKKIYFIWVTRTQRQFEWLADIIREVEENDSRDLVSVHIYITQLAEKFDLRT
TMLYICERHFQKVLNRSLFTGLRSVTHFGRPPFEPFFNSLQEVHPQVRKIGVFSCGPPGMTKNVEK
ACQLINKQDRTHFSHHYENF

>rat-Rn-Duox2: GenBank™ No. NM_024141

MLPTSLKTLVLLGALLTGPLGPAGGQDAPSLPWEVQRYDGWFNNLKYHQRGAAGSQLRRLVPANY
ADGVYQALQEPLLPNARLLSDAVSKGKAGLPSAHNRTVLGLFFGYHVLSDLVSVETPGCPAEFLNI
YIPRGDPVFDPDKRGNVVLPFQRSRWDRSTGQSPSNPRDLTNQVTGWLDGSAIYGSSHSWSDTLRS
FSGGQLASGPDPAFPRNSQNSLLMWMAPDPATGQGGPQGLYAFGAQRGNREPFLQALGLLWFRYH
NLCAKRLAQEHPHWGDEELFQHARKRVIATYQNIALYQWLPSFLQKTPPEYSGYRPFMDPSISPEF
VAASEQFLSTMVPPGVYMRNSSCHFREFPKEGSSSSPALRVCNNYWIRENPSLKTAQDVDQLLLG
MASQISELEDRIVIEDLRDYWPGPDRYSRTDYVASSIQSGRDMGLPSYSQALQALGLEPPKNWSAL
NPKVDPQVLEATAALYNQDLSRLELFLGGLLESHGDPGPLFSNIILDQFVRLRDGDRYWFENTRNG
LFSKEEIAEIRNTTLRDVLVAVSNVDPSALQPNVFFWQEGAPCPQPQQLTTEGLPQCVPVTVIDYFE

GSGAGYGVTLLAVCCFPVVSLIIAWVVARFRNRERKMLLKKGKESLKKQTASDGVPAMEWPGPKE
SSYPVTVQLLPDRSLKVLDKRLTVLRTIQLQPTQQVNLILSSSHGRRTLLLKIPKEYDLVLMFNSEE
DRDAFVQLLQDLCVCSTPGLRIAEMDEKELLRKAVTKQQRAGILEIFFRQLFAQVLDINQADAGTLP
LDSSQQVREALTCELSRAEFADSLGLKPQDMFVESMFSLADKDGNGYISFREFLDILVVFMKGSPQ
DKSRLMFTMYDLDGNGFLSKEEFFTMMRSFIEISNNCLSKDQLAEVVESMFRESGFQDKEELTWE
DFHFMLRDHDSDLRFTQLCVKGGAGGTGDIFKQSNACRVSFLTRTPGNRVMAPSPRLYTEALQEK
MQRGFLAQKLKQFKRFVENYRRHIVCVTIFSAICAGLFADRAYYYGFASPPTDIEETTYVGIILSRGT
AASISFMFSYILLTMCRNLITFLRETFLNRYIPFDAAVDFHRWIAMAAVVLAVVHSLGHAVNVYIFSV
SPLSLMTCVFPSVFVNDGSKLPPKYYWWFFETVPGMTGVLLLLVLAIMYVFASHHFRRHSFRGFW
LTHHLYVVLYALIIIHGSYALIQLPSFHIYFLVPAIIYVGDKLVSLSRKKVEISVVKAELLPSGVTYLQF
QRPKTFEYKSGQWVRIACLSLGTNEYHPFTLTSAPHEDTLSLHIRAVGPWTTRLREIYSPPVGGTSA
RYPKLYLDGPFGEGHQEWHKFEVSVLVGGGIGVTPFASILKDLVFKSSMGAQMLCKKIYFIWVTRT
QRQFEWLADIIREVEENDSRDLVSVHIYITQLAEKFDLRTTMLYICERHFQKVLNRSLFTGLRSVTH
FGRPPFELFLDSLQEVHPQVHKIGVFSCGPPGMTKNVEKACQLINRQDRAHFVHHYENF

>frog-Xt-Duox1: Ensembl No. ENSXETP00000051515

NDINWEVQRYDGWYNNLAYHSTGSPGSKLLRHIPAFYTDGVYQVPKEPELPNPRSISNVVTKGKSG
LPSSRNLTVMAISFGYHVLSEVVYTGKPACPAEFLNIEIPKGDLVFDRTNTSKVVIPFQRSKWYPDT
GRSPNNPRDHVNSVSAWIDGSSIYGSSHSWCDALRNFSGGKLASGPDEGVPRFAKSDLLMWKVPN
PSTGETGNNGLYAFGNARANESPFLQSLGILWFRYHNYKAQEFAKENPEWSDEVLFQHARKWVIA
VYQNIVFYEWLPAFLSRNVTAYKGYQQHVDPSISPVFTVLLSQMIYTMMPSGIYMRNKDCNFQNVS
GSNGRLWPALRLCNNYWNRENSNLKNSSSTDEILMGMASQIAEKEDNILVNDLTDYCYGGQIKYSR
TDLFAASILHARDLGLPNYNKVCEYYQCPLLENWEKMTDKELGKKLASLYGNDTEKLEFIPGLLA
EMAGNSINFFSDIVLEQFYRLRDGDRFWFENVKNGLFSKKDIDNIRNIKLQNVLVAVTDAGDTIQQ
NVFFLKEDDPCPQPKQLQSEELEECVPFTVTDYFEGCAAGFGITIVALCCLPLVSFLIAWLVAKSQK
RTFRAFQKKITTANKNSMCGSEASEWCGYKEPSQDVIIQLHPNQILKVLDSSKKKPRVVNLHKHP
NVKVVLSNSDGSRTVLIKIPKEYDLVLQFNNQRDRDVFIEQLKESLAGSTISPTFSHLKETVLLKES
FTKKQRQQMLETFIRHSLSHVIDINKEHAGTTQGQNFRDVLQCELSREEFADSLGLNPNAQFVES
MFSMADKDHNGYLSFEEFCFILCSLIKGSAEDKLKFIFSMHDVNGNGILPKEEFSRMLRSFRNVSS
FLSNEKTENVIESMFNEAGISNKKELAWEDFYGLFKDHKNILNQTNLYFDGIHVINQSYVLCIICSI
DSNIYTNISSALCFILFICFIALLLIKKHLCHPNRNTSQIQLPNIYTVARREKYETSTFRQKIQQFKRLV
ENYRRHIVCLIIFYGISAGLFAERAYYYGFASPSSGIADATFIGLIISRGSAASISFMFSYMLLTMCRNL
ITLLRETFLNQYIPFDSAVDFHRLIAVTALVLSILHSLGHLVNVYIFTIIPLSVLSCLFPTVFVDDGSDH
PNKYYWWFFETVPGMTGVLLLAVMALMYVFSCYHFRRVSFRCFWLTHHLYVVFYILTIIHGSFALI
QQPRFHIFFIVPALIYSADKLISLSRKKIQINVLDIQRLPSDVIHLEFQRPNDFDYKSGQWVRIACLDL
GTDEYHPFTLTSAPHEDTLSLHIRAVGPWTTKLRELYSSKKVENIPYPKLYLDGPFGEGHQEWNKF
EVSVLVGGGIGVTPFASILKDLVFKSSVNSRIHCKKVYFIWVTRTQHQFEWLTDIIREVEKNDKQEL

LSVHIYITQLAEKFDFRTTMLYICEQHFQKVKNQSLMTGLRSVTHFGRPPFAGFFSSLQDVHPKVK
KIGVFSCGPPGMTKNVENACRKLNKRDESYFVHHYENF

>frog-Xt-Duox2: Ensembl No. ENSXETP00000056553

KNYCLEMLYRYAVIPFRNPFNSQKNIHWEIQRYDGWYNNLHQHNRGSAGFRLLRLVPAAYSDGVY
QAIQEPELPNPRQISNVAMKGDSGLSSNRNRTVLGVFFGYHVLSEIISTENSGCPAEFLNIAVPKGD
KIFDPDATGQVMLPFQRSQWWKCSGESPNNPRHQINSVTAWIDGSSIYGSSHSWSDALRSFSGGKL
ASDSDPMFPKEAGDALLMWRAPDPSTGHRGKEGIYGFGNARANENPFLQAVSILWFRYHNYLAQQ
FAKKYPQWSDEDLFHNTRKWIIATYQNVVMYEWLPVFLQKEVSAYEGYNQHVDASLSHEFQAAV
QFMATLLPAGIYTRNKTCHFHNVQSSDGGFWPAYRLCDSYWKRKNPNIKTAQDIDNLLLGMISQIA
EKEDNVVVEDLRDYWYGTLKYTRMDYISWSIQQGRDFGLCSYNHARQHFGLSPIANWSEINRSLY
QRNKELFEDLANLYGNDTEKLELFPGVMLESDGDPGELLTAIIMDQFQRLRDGDRFWFENSKNGL
FTLEEIERIKKTTFHDVILSVTSADLGDFQPDVFKWVNGDPCPQPEQISAQMLANCTPPTVTDYFAD
SVTGYSILIAVICSFPLAQKVSFFIAWMVAQSRRRNFKRFQKRRAIQSIRQSLPAEGISAQEWQGPKE
PTCNVLLQIDPQHVIKVLDSRCAIRRTISLQSQKNVIVIQSNSHGSKTLLIRIPKEYDLVLLFHSEEEC
REFINQLQENGIELHQYNKKEKHLLKEAVTKKQRSHILETFFRQSFAQVLHITQSDNMGSSDKVKE
ALKCELSRTEFAESLGLKPQSMFVESMFSLADEDGNGYLSFREFLNILVIFMTGTPKEKSELMFKM
YDVDGNGFLSKEEFFTMLKSFIEISNNCISKDQTEQVIEIMFRDSGFQNKEELTLEDFHYLLRDHD
KELRFSQICIAGMDAPEVFNQMCRRVSFVDTTNMCQSPNPSSLTGRIRKKVRNTTFLMHINFFRTN
FPHLFDLEKQTRKLINKPKTSSEARLYTEAKKEKYPKGIIHQKLQEFKRFAENYRRHIVCVVIFYGIS
VGLFLERAYHYAFESQHRGISEVTMPGIIISRGTAASISFMFSYILLTMCRNLITFLRETFLNQYIPFD
AAVDFHRLVAKTAIILTEFKLLFSHQTDIKLMIVFTLRLCMSCYFLQATYSNDNVLSLNLHPECLPLL
TGLTGVLLLAVLALMYVFSSHHFRRISFRGFWVTHHFYILLYILTILHGSFGLLQQPKFHVFLMAPA
LIFIRDSLISLRRKKTEINVMKADLLPSDVTCLRFQRPADFDYKSGQWVRIACLALGTNEYHPFTLTT
APHEDILSLHIRAAGPWTTRLRELYSPQSVAELGGYPKIYLDGPFGEGHQEWNKYEVSVLVGGGIG
VTPFASILKDLVFKSSVNSKIACKKIYFIWVTRTQRHFEWFADIIREVEENDKCDLVSVHIYITQLAE
KFDLRTTMLYICERHFQKVLNRSLFTGLRSITHFGRPQFEQFFISLQEVHPEVRKIGVFSCGPPGMT
KNVEKACQMLNKENQAHFSHHFENF

>zebrafish-Dr-Duox: GenBank™ No. AB255050

AITWEVQRYDGWYNNLADHDRGAADASLVRLYPAQYMDGVYLARQEPHLPNPRRISTTAMSGQSGLLSH
KNRSVLSVAFGYHVWSEISESRRAGCPPEFMHIKVQKDDPVFVSNSSQPVLLQFQRADWDTSTGKSPNNPRT
QVNHVTAWIDGSSIYGSSSSWSDALREFSGGRLSSSSSRDMPRRSSNGYLMWSSPDPSSGPDSGSQELYEFG
NAWANENIFSVTEGIIWFRYHNYLASKLHKEHPSWSDEELFQHARKRVIATFQNIAFYEWLPAFLGTHVTSY
PGYQKYVDPGISVEFEAAAVRFGLTLAPPGVYKRNRTCHYRSVVNDDASKSPGLRLCNTFWNRRNNPHLQSS
LDVDELIMGMASQIAEREDNIIVEDLRDYMYGPLRFSRSDAVALTIQRGRDFGLPSYNQIREALSMAPVNSFE
DINPKLKDTKLLKELADLYENDISRLELFVGGLLETQEGPGPVFSTIILDQFERIRNADRFWFENKQNGLFTEE
EIKAIRNTTFHDVLLDVTSAEKGDIQRSVFFWKNGDPCPQPQPIRASDLQPCTKAFSMSYFDDSSKVGFGVTV

VVLFLFPVVSYIVASAVAQVRTARFKRFQQKRSGSTKHKEPAHGTTASEWLGHNTAPRQVTLAFSEKKVFQ
TFDENGSPLSSHSLGNQDHLXVIVSNDHQHRALLLKIPKEYDLVLFFEDGVQRSEFLSLLRSELEGRIQSLTV
MEKSEKEMLCDAVTREQRGKIVETFFKHAFSKVLDIDKSDAGDLSSRTREALQCELTRAEFASVLGLKSDSL
FVESMFTLADKDGNGYLSFQEFLDVIVIFMTGTSEEKSKLLFSTHDIKGDGFLSKEEFTSLLRSFIDISGALSKS
QADDGIAAMLQTAGLYNKDRFSWEDFHFLLRDHSAQLNIKGMEVLGKKKLGRQHKVSFIRKNSSSSSVEEL
THTPEEEHGQELRQRQTKKAGQSQAKLYVRPQRERFNRNPVQQCVQQFKRFIENYRRHIICTVVIYAISAGL
ALERCIYYGLQAHSSGIPETSMVDVLVSRGSAAAISFLFPYMLLTVCRNLITMCRETFLNRYIPFDAAIDLHRQ
MAATALILSVVHSLGHLVNVYIFCISDLSILACLFPKVFSNNGSELPMKWTFWFFKTVPGITGVILLLIFAFMY
VFASHYFRRISFRGFWITHHLYVLIYVLTVVHGSYGLLQQPRFHIYLIPPGLLFLLDKLISLSRKKVEIPVLKAE
LLLSDVTMLEFKRPQGFVYRSGQWVRIACLTLGTDEYHPFTLTSAPHEETLSLHIRAAGPWTSKLREAYSPE
KHQSSEDSQKLYLDGPFGEGHQEWTDFEVSVLVGAGIGVTPFASILKDLVFKSSVKFKFHCKKVYFLWVTR
TQRQFEWLSDIIREVEDMDMQDLVSVHIYITQLPEKFDLRTTMLYVCERHFQKVWNRSLFTGLRSVTHFGRP
PFLAFLSSLQEVHPEVEKVGVFSCGPPGLTKNVEKACQQMNKRDQTHFVHHYENF

>medaka-Ol-Duox: Ensembl No. ENSORLP00000006648

KGYCMHVTWHEIKQNIFTSATDSQSEITWEVPRFDGWYNSLGNPRRGSVGSRLVRLVPAQYWDGVYQPVL
EPLQPNPRRLSRVLAEGPSGLPSTRNQTVLFLFFGYHVAFEISDLRSSGCPPEFMNIPVPQGDPVFDPNATGEV
QLPFRRGEWDQASGQSPSNPRTQINSVTAWIDGSSIYGPSSSWSDLLRSFSGGRLTSGSEWNMPKQGGETNF
MWSAPDPSTGEHRPQGLYELGNAWANENMFTAAEGIIWFRYHNYVASKLHEEHPEWSDEELFQNARKTTV
ATFQNIALYEWLPGYLGNKTLPPYPGYQQFVDPGISPEFEVAAMRISATMTPPGVYMRNRTCHFRNITNADG
STSPAIRLCNSFWKRQVINVKTGQDVDDLLMGMASQIAEREDNIVVEDLRDYMYGPLRFTRTDLVAMTIQR
GRDFGLQSYTEIRKALDLPPVETFEEINPGLNRTNPQMLQAVAELYDGDISKLELFPGGLLESLDGPGPVFSAI
ILDQFERIRNGDRFWFENKLNGLFTDEEIQEIRNVTYRDVLLAVTSADATDIQENVFFWKDGDPCPQPAQLE
ASMLYPCTNATKLNYFDGSKAGFGIFIIVLFLFPVVSFLVACMVACLRKYRYKRFQRRRRIAGNGTEKPPVGI
PAFEWQGHSKQMCPISVEVEKRRLQVFDRSGSTHRSLNLGNQDCLDVLLANDRQNKALLLKVPKEYDLVL
FFEEESKRADFIKYLCLELKEIRQEIRVKELREKELLKEALTKEQRAQIVETFIRHAFSKVLEIQKCDAGDMSG
VSPRRAREVLQCELTASEFADALCLKPDSLFVDSMFTLADKDGNGYLSFQEFLDVMVIFMKGSPEEKSKLM
FSMNDVGGNGYLSKEEFARMLRSFIEISNGALSKTQAEDGIKAMMQAAGFNNKEQISWEDFHFLLADHDKE
LQFAKLNVKGQTCIVKEVLPLWMEKEGRKRLSQCQRVSFICPKKSVICFWLCLTVCSNKDKAEGLELRRRK
NTSIKTPNVYVKAKRQEYIRNPVQQKIQQFKRFIENYRRHIVCFIITYSITVGVTLERCYYYGLQAEATGVPGT
TVVGIIVARGSAAGVSFLFPYMLLTVCRNLITLCRETFLNRFIPFDAAIDFHRSLAMTAVVLSVAHSLAHVVNI
YIFSISDLSILSCLFPKVLNNNGSELPPKWYWWFLQTVPGITGVLLLLVLAFMYVFASRYFRHISFRAFWITHY
LYIVVYILTVIHGSFALLQEPRFYIYLIPPSLLFLLDKLISLSRKKVEIPVVRAELLPSGVTHLEFKRPSGFVYRS
GQWVRIACLMLGADEYHPFTLTSAPHEETLSLHIRAVGPWTSQLRELYTDESLLELGSYPKLYLDGPFGEGH
QEWVDFEVSVLVGGGIGVTPFASILKDLVFKSSIKSKFMCKKVYFIWVTRTQRQFEWVSDIIREVEEMDTQE
LVSVHTYITQVAEKFDLRTTMLYVCERHFQKVWNRSLFTGLRSVTHFGRPPFVSFFNSLQEVHPEVGKMGV
FSCGPPGLTKNVEKACQQMNKRDQAHFIHHYENF

>fugu-Tr-Duox: DDBJ™ No. BR000283

EVPRFDGWYNSLASARRGAAGAHLVRLTPARYWDGVYLPVQEPLMPNPRRLSSLLAGGPSGLAST
RNQTVLSLFFGYHITFEMFDSRTPGCPPEFMNIPVPKGDPVFDPTATGKVLLPFQRGPSDRDSGQN
PSNPRSQVNSVTAWLDGSSIYGPSASWSDSLRSFSGGLLASGSEWNMPRGGTHLHVERCRPLYRRT
WPSGPDELGNAWANENTFTAAEGIIWFRYHNYVASELHREHPGWSDEELFQKARRTVVATFQNIA
VYEWLPAHLGDKELPPYPGYQKFVDPGISPEFVAAAIRFGITMVPPGVYMRNKTCHFREVANADGS
SSPALRLCNSFWSRQSRNMKTSHDLDDLIFGMASQIAEREDNIVVEDLRDFMYGPLRFTRTDLVAV
TVQRGRDFGLRSYADVRNALDLPPVETFEDLNPELSSSNPKLLRDVADLYSGDISKLELFPGGLLES
LSGPGPVFSAIILDQFERLRNGDRFWFENRQNGLFTEEEIQKIRRTTFHRVLVKVTSAGAADLQEDV
FFWKDGDPCPQPTQLKESMLHPCTNATKLSYFDGSKAGFTVFILVLFLFPAGSSRGEEKLEAEQRN
QLWGPLVCNGCEMFNTKSALTNQMGVGFGFAACEWQGHQKPLLPVTVEVSEEKRVQVCDRSGAV
LRCFDLGVQDHLEVVLSNNRHRKAALLKGSKERHLVLFFDNGPDVRFVQLLHQCSSDVGVKEMR
EEELLQEALTREQRAQIVETFIRHAFSKVLEIDKCDAGDMSGVSRKKAKEVLQCELTAAEFADALG
LKADSLFVDSMFTLADKDGNGSLSFQEFLDVIVIFMKGSSEEKSSLMFSMNDIGGTGSLSKGEFAR
MLRFDPHFLFILAPVPITRPILPFCRSFIEISNCTLSKKQAEDAIGAMMTAAGFDHKEKITWEDFHF
LLRDHEKELHVRRPNVYVDPRRERYIRSPVRQKIQHFKRFVENYRRHIVCSALVYGMAAGLCLERC
YYYGFQAESTGLPETSVVGVALARGSAAAVSFLFPYMLLTVCRNLITLCRETFLNRYIPFDAAIDFHR
FMAMAAIVLSVVHTLAHVVNIYIFSLSDLSILSCLFPKVFRNNGSERPMKWSWWFFQTVPGLTGIL
LLFTLAFMYVFASRYFRRISFRGFWFTHCLYVVVYALTVIHGSYALIQEPRFHIYLIPPALLFLLDKLI
SLSRKKLEIPVVRAELLPSGVTHLEFKRPQGFVYRSGQWVRIACLTLGADEYHPFTLTSAPHEDTLS
LHIRAVGPWTSQLRELYAEESVLRGLSEGRTEASPLMSMSPSAAVSITHALFPPKLYLDGPFGEGHQ
EWDDYEVSVLVGGGIGVTPFASILKDLVFKSSAKSKIRCKKVYFIWVTRTQHQFEWVSDIVREVEE
MDTQQLVSVHTYITQVAEKFDLRTTMLYVCERRFQKVWNRSLFTGLRSVTHFGRPPFLSFFNSLQD
VHPEVGKIGVFSCGPPGLTKNVEKACQRMNKRDQAHFIHHYENF

> tetraodon-Tn-Duox: Ensembl No. GSTENP00025605001

EVPRFDGWYNSLASPRRGAAGSWLVRLMPAHYWDGVYQPVQEPVRPNPRRLSRLLVGGPSGLPST
RNQTVLSLFFGYHVTFEMFDSRTPGCPPEFMNIPVPKGDPVFDPTATGKILLPFQRGPSDKDSGQN
PSNPRTQVPYPDFNQKKVKEGVVIWTNTCFCFLKHVKVNSATAWIDGSSIYGPSASWSDSLRSFSG
GLLASGSEWNMPRQAEGRTFMWSAADPCTGEHGPQGLYELGNSWANENIFTAAEGIIWFRYHNY
LASRLQQEHPAWSDEELFQNARKIVVATFQNIALYEWLPAHLGDRELPPYPGYQKFVDPGISPELVA
AAMRFGITMVPPGVYMRNKTCHFQEVVNLDGSSSPALRLCNSFWSRRSPNMKTSQDLDHLLFGM
ASQIAEREDNIVVEDLRDFMYGPLRFTRTDLVAVTIQRGRDFGLRSYTEVRNSLDLPPVGSFDDLNP
ELSSSDPKVKPTNQSSWEGQRLTNVLGVSLSRPRQLLRDIAELYNGDISKLELFPGGLLESSGSPGP
VFSAIILDQFERIRNGDRFWFENRENRYVPLSQTFRANWGSRQSRTLSHCRLFTEEEIQKIRRTTLH
HVLVAVTSAEATDLQKDVFFWKDGDPCPQPTQLKESMLHPCANATKLSYFDESKAGFTVFILLLFL
FPAVSFLVAYMVAYHRKYRYRKFQRTRKAGDRTAEPAVGVAACEWQGHKKPLLPVRVEVSKGKVQV

SECSGSLLRCFNLGSQDHLDVVLSDNRQRRAALLKGSKEYDLVLFFDDEARRAAVVEHLHRGSAD
VRVKEMAEEELLKEALTREQRAQVVETFIRHAFSKVLEIDKCDAGDMSDVSRKKAKEVLQCELTA
GEFADALGLKPDSLFVDSMFTLADKDGNGYLSFQEFLDVIVIFMKGRSFLEISNCTLSKAQAEDGIR
AMMQATGFDRKEKITWKDFHFLLQDHEKELQFAQLNVKGMEKRGQRRLSRDQRVSFICPASRWG
HWFSIIFNCNDHFNTSCSMSAASARRKYTRFADRKSQFLPNVFVDPKREQYIRSPIRQKIQQFKRFV
ENYRRHIVCFIVVYGITAGLCLERCYYYGFQAESTGLPETSVVGVAFARGSAAAISFLFPYMLLTVCR
NIITLCRETFLNRYVPFDAAIDFHRLMAMTAIVLAGWRSRYEGTHLRARKLTAASPPPVVHTLAHVV
NIYIFSMSDLSILACLFPKVFLNNGSELPMKWSWWFFETVPGTAEVKMVNLSSHRKMMPVHPGCR
KATRTPILVLLLLGLTGILLLFTLAFMYVFASRYFRRISFRGFWLTHCLYVLVYALTVVHGSYAFIQEP
RFHIYLIPPALLFLLDKLISLSRKKLEIPVVRAELLPSGVTHLEIKRPQGFVYRSGQWVRIACLALGA
DEYHPFTLTSAPHEGTLSLHIRAVGPWTSRLRELYTQDSLQQLGAFPKADGPFGEGHQEWDDYEV
SILVGGGIGVTPFTSILKDLVFKSSMKSRIRCPTVFFPNILKVYFIWVTRTQRQFEWVSDVIREVEEM
DTQELVSVHTYITQVAEKFDLRTTMLYVCERHFQKVWNRSLFTGLRSVTHFGRPPFLSFFNSLQDV
HPKVGKMGVFSCGPPGLTKNVEKACQRMNKRDQTHFIHHYENF

>sea urchin-Sp-Duox: GenBank^{TM} No. XM_001176709

MTPRDLRGPICVGLLLCMIALCSGQTTENATTVMMMTEVTNGTTAAMMTEVTTVPTQTAQGPSTAQTTSST
DPPGTTTAPPVTTPADEDEPIVNDNELLRSIGAFKDDSHSEPEGYDGWYNNLAHPDWGGAELPLTRRLPVSY
ADGVYAMAGKDRPNPMSISKATMQGETGQRSHLRRTALMTFFGQQVVEEILDAQRGGCPREYENIKIPDGH
EYLKEKEGISIMPFVRSRYSFNTGYSPNVPREQLNEITPWFDGGLVYGTTKAWADALRSFKDGRLADNGEIG
GEPQFPEQNTLGLPMANPPNPIAEGAARLQRSERFFKLGNPRGNENPFLLTFGVLWFRWHNYWADKFKAET
DWEDERIFNEARKWVIATYQSVVFYEWLPGYLNLNENETAEVEYSGYKGYIHPGITHEFQSAAMRFGHTLV
PPGVMRRNEQCVFRTSTMKSSGFTEDQDEFSESQGNHGVRTCNSFWNPQHSVREHDIEEFLLGMASQVTER
EDNIITEDLQRRVFGPLEFSRRDLMALNIQRGRDHGLPDYNTARVSLGMDRRETFESINNASAHADGVETFID
SEVLDNLARVYENDIDKVDIWAGGLLETTSNGPGELFRFIILDQFVRSRDADRFWFENNVSGQFTNDQIDFIK
KVKLWDIIVACTNINGSSLQKDPFHYTPDDPCYDQHPFEGENRTISENDMENCTKLQTFDYFEGSEVSYALSF
LALGIWVVGVVAVLLILAHIRQHSIQEARKLQHRKTRSRDPSSLKTYAAAEWCGKKEGSRQVQVKLGPGKK
IRITNERGNKILRTVDLTHYTKVGLLVSPDGKRRHLVVKFDREYDIVLKFSDPDSRLEFISDFESFLGSEEVGV
GRERQEIGEKELLKVAVTKEHRTKLLEKFFLMAFSQAFKLDFDPENLESLNNKETKDILECELTKTEFADVLT
MKPDSLFVEQMFELVDQDNSGSISFREFLDVIVVFAKGQPEDKLKLMFNMYDIDRSGHLSREEFRQMLKSM
MEMVSASVEETDLDKLIHDMFQNAGLGDKEALSLDDFIAVMAEHKDELNNAKLDIAGNIPQIAGQKDAGPP
GRGAATVIRRGNLNSRARQTIIRAYKDRDQASTKANGAAKGAGGSGGGVRKRAQSKSVRVETVQQEEAKT
QSSKAYNTVVRFFENNRLQIFYVVLYLLVLAGVFIERAYYYSVEREFAGLRRIAGFGVSVTRGAASAMMFT
YSSLLVTMCRNTITKLRETFLHRYVPFDSALNMHKLIAMLALFFSIMHTIGHSINFYHISTQTADDLTCYFRDF
FHRSHELPKFHYWAWGTITGFTGILLVMVCTVIYTFAFQYARRRVFNLFWFTHNMWIIYFILMFLHGSGRLV
QPPFTHYFALGPIVLFTLDKLVSISRKKAEIAVTRAELLPSDVTMLEFKRPQGFEYKSGQWVRIACKTLSSSEY
HPFTLTSAPHEENLSLHIRAIGPWTMNLRATYDPNVVREHPLPKLFLDGPYGEGHQDWYQYEVAVLVGGGI

GVTPFASILKDIVNKSTIGARVTCKKVYFIWVTRTQKHYEWLTDIIRDVEDNDTNDLVSVHIFVTQFFQKFDL
RTTMLYICERHFQKISNRSLFTGLKSITHFGRPQFTSFLQSLEDEHPGVGKIGVFSCGPPGMTGGVEQACVDL
NKFDGAAFIHHYENF

>ascidian-Ci-Duox-A: DDBJ™ No. BR000261 (Chromosome 1p: 60.95k)

LMFCCVIQFVVLIYNYDSLVLGQEVEKEYPPYDGWYNNRAHPEWGTVDGPLTRRLPSHYADGTYA
PSGGERPNPRTISENTMAGLTGQGSSTKRTALLVFFGQQVVEEILDAQRPGCPPEYFNIKIPKGDPL
YDKTGQGNIELPFLRSRYDMSITGYSPNNPRQQLNEITPFIDGGLTYGINKAWADALRFQSKPTDCR
GELASYKNEGKFPIENSLGLPLANPPPPRDHNTYCIGLYWLGNPRGNENPFLLTFGVLWFRHHNW
LARYIRDKHSTWSDEKVYNEARILNIAIHQKIIFYDWLPLFLGECLPTYTGYKSAIHPGITHVFQSAA
MRIGHTLVPPGVYRRTNYNTTTKQCTFLNTTLKTAGDRHAFQALHVNKPGLRTCNTYWNSLEAVV
ESDIDQLLMGMSSQVAEKEDNIITPDLRGSVFGPLDFSRRDLMALNIQRGRDHGLPDYNTARASFG
LKKRTTFEEINPDLFAATPGLLGNLTATHDDDISKLDVWTGGLLETLSTGPGELFRHIIRDQFIRIRD
GDRFWYENSQNGLLTAEDLAFVQATTIRDVILRTNPTMNATYDIQNNPFQAAENDSSLYCPQPRQL
SELHMEECTEPESFDYFTGSEISYSLTFASFGLFIIATILFMYCLGYRSEKRKLQMKKAINKHRSTQQ
REGDISDIATEVLGTTQSREVQIKLGPGKLIHVKSSSGRQNYRSIDLSKADDVRFVKAYNHGRNTMF
LRVDKDTHDLILMFNDTEERDSFIARCKHFVHSHQISLSEQEVSHNELMDRAQTSEMRDQLLQRF
FKAALIAVSSIKVEDGDMSDVINSREARKFIHVELSKKEFADYLKLKPNSLFVDQMFSSADSDGSGA
ISFREFLDIMVLFTKGSPEEKAKLMFNMYDLDKSGELSKKEFKVMLKSMMDMVNASVDSEQVEE
LVNTMLATHGYEHKESLTLEDFQTVMQKYSTELSDASLTVPGHYIIALAYSFCKKIAFTVDSRKCPS
ARITSNKSKKNFFRNVVNCRLDYRKKFRICDTFKKNRTPETSRIQIKVYEKVFKPGRLNNWLRAME
NYVENHRLQIFWLTLYLLVLAGIFIERAYFYSVEREFAGLRRIAGYGVSITRGAASAMMFTYTSLLVT
MCRNTITALRETFLHLYIPFDSAITMHRIIAWLALFFTGMHIIGHSLNIYAISTQTPGDLTCLFRDFWR
TSDVLPKFHYWCWQTITGITGVLLTLIVIVMYTFASDYSRRRVFQWFWWTHNFGYIFLFFFMILHG
SGFLVQDPFFYYFFLGPAILYTLDKLYSVSRSKCEISVVNAELLPSEVTHLEFKRPVNFNYKAGQWV
RIACLAQSQNEYHPFTLTSAPHEDTLKLHIRAVGPWTINLRSIYNPDVLRDSPYPKLYLDGPFGEGH
QDWYKYDVSVLVGGGIGVTPFASILKDLVSVAQSGVKIQCKKVYFMWITRDQKQYEWLTDIIQEVES
KDASDLLDTHIFITQFPQKFDLRTTMLYICERHFQKVAGKSLFTGLRAVTHFGRPEFKSFFVSLTEE
HAEVEKFGVFSCGPPPMTSCVEQTCAKLNKYEGASF

>ascidian-Ci-Duox-B: DDBJ™ No. BR000262 (Chromosome 1p: 2.80M)

RRMDGWYNNLDHPQWGSPGERMIRRSTNGYSNGINKPMEDGLPNPHTLSRLLMRGESGIPSVTG
KSVFLAFFAGQHVGDDMIDSRRNACPSEYINIEIEENKESNNHILPMYRSKFITGSGSSVNSPRMLV
NEATSWLDGSVIYGNSHSWSEHLRSFERGRLKEEDGHPGYPSFNRNEIPLYNPTIDLKRPPKTRNP
EELLSFGNPRGNENPFLMTIEIIWFRWHNHLAEKIAVQNPDWSDQQIFDKARKWTIATYQNIAFYE
WLPEIIGTSPPPYREYNKFMLPSVSVEFSIAAMRLGHSLVPSGVVLRQPDCSPIDPLPEDYLASAGEP
ALRLCNTYWQLQDALLDLGIEGIVLGMTSQNAEEEDAVVVDDLAGFLYGPLKRTRMDLEAMTIQR
GRDAGLQRYNEVRKTYDLPPITDFSQLNPSLPNEVIARLRNAYNNDTNKLELYVAGMMEDRSNAS

NVLFYKIILEQFQRTRDGDRLWFENAQNGLFNASELQEIRQTTMRDIIAATTTFNRSLFQPNVFNTA
GSRCASITVRKDMFSKSCTPNTGIDFFYGYWGKYAGIVVLLLLFPFVSYFVMCMIAWKRQYDLAKA
KKIATQKVTRRKSSSQNEAEIESEDGAVFSGGQDKSRRVVVMLEGTCIKLFSSAGRELRKICFSHFK
KSFLFMSRNKRKDLIVIRVPKEYDVVLRFNDSLERVEAIKNVQEFMQHNGVNVDVFEMDESALKE
EAVTKEARQALLETFLRQTFAEALNIDDAVEQNSKAKYRHKTMEVADCELTQMELAETMGMKPDS
LFVTQMFQLADTDHSGYLSFREFADLIILLMNGSPEQKAKMLFDMYDVDHSGEINREEGRNMIKS
FLEMAGANLGPDEVSAAVSTIFKEAGIGENKDSLTLEDFTYVLLKDHREAFESSELSMPGRSNLYK
DQGELSEQNKMSDQRRVDKAKIIFYQKMVQSNGKVFSDATVSHEEKTWFARTWKYLKRNVENYR
RHIFCMVIFYGITIALVVERATFYAFGAEHIGIRRVTEWGIIISRASAAAISFHFSFILLTMSRNLITTCR
ETFLKNFIPFDSAVAFHKQIAYVALVETIFHVLGHASNFYHFCVHPLPVLACLFPKIFVDDGSDLPKS
LTWWFFETITGLTGVLLLLTISIIYVFAMQYSRRFCFRAFWITHHLYTVLYILTILHGSLGIVQAPVFH
FYLVVPVVIFLIDKMITISRSKVQITVIKAEALPSGVLNLVFKRPVAFDYQSGQWVRIASLSLGTNEYH
PFTLTSAPHERYLSLHIRSVGPWTSNLRNLYQTAVEHQGKLPNLYLDGPFGEGHQDWYKYEVSVLV
GAGIGVTPFASILKDIVNRTSTKKGSHIPCKKIYFIWVTRTQRHFEWLTDIIRELEETAGGDLVSTHIY
ITQFANKYDLRTTMLYICERYFQKVANKSMFTGLKAITHFGRPQFEAFLDSLQTKHKEVRTLGVFS
CGPPGLTNGVEDACRNLNKLNKARFNHFYENF

>ascidian-Ci-Duox-C: DDBJ™ No. BR000263 (Chromosome 2q: 2.08M)

QQEIGKEFPPFNGWYNNRGNPSLGIPDSSLTRSLPPHYKDGVYEPSGWERPNVRSISNLIFSGPQG
LPSTNNKTALFLFFGEHVMQDILDTSRPGCPPEYFNIMLNASDSHFSTTEMPYERSSYQASNTGYS
PNHPREQINAVTSYLDGSQIYGHTKAWSNNLRLLRSTCNGRTSTSSLFSIGSRRGNENPFVLTIGIT
WFRHHNWLARNIRDSNPNWSDDDVFNEARIQNIAMYQKVLMYEWLPGLLGTCSSLNQTYSSGTS
ASVSDIFEGAARHFLSTITPPGVFVRSKFNESCEFRTTSATSPALSLCNSYWEPAVKIFASIDGLTMG
MASQIAELEDNVITPSLRHNYYGSRRFSRRDLMATILQKGRDHGLPDYNKVLMYEWLPELLGTCS
SLNQSQCLNVTPYTGTLYQEFTFFFVINWWFVRDHRGSPGELFRYVIADQFLRLRNGDRFWFENV
KTSLLSSTKLNEILNTTFRDVILRTNPTINGSFDIQDNPFVWATGDSKLFCPQPYQLTENVLEECTPL
QRYDYFTSSEVSFPLSFAFFGVFALITLLVMYLVGRQRMKSALKTKKDITTKKRATITTSIASGNVTIC
TEILEFFVKTVYLEYGCSVHAIMYHEVFDIYVLSFNDSYELDEFFNELHNFVKPLGLKCNTFAIPEK
HIMEEARTKEMRDKQLQNFFRTALSQAMNIESEDLDRPKRKTLKDLVKVNITKSEFAEYLKLKED
SLFVEQMFLVADSDEDGTISFREFLDIIVLFTKGTPKEKAQLMFNMYDLDKSGGLSKEEFTTMLKS
MMEMVNSSADVDNIDSVVDDMMRANGFSSKDSLDLNDFLMLLGQYSDISNQQSTSNGHKTYQAK
RQSKYHRYSKRFKEGYVCLLHCIKSMFTKLYITHRPQTPQRRLTRKVKTVREDYKTKSYEKFFVAL
VKLTEHYANHIFCLSLYSLITAGVFLNAFFVVYSKYATGLYGIGGPLMALARASAAALMFNFSTLLLT
MCRNIITFLRETFLHRFIPFDSAVTMHRIVAWMALAFTALHILAHGINFYSIVTQSPDDMACLFRDM
WYPSDYIPTFVFWLFQTITGITGVILTLALIVMYVFASNYARRMIFNWFRWTHKLGYLSLYFFSFVH
GSGMLISSPQFYYYFLVPGILFTLDKVYTYSRKKAYISVVRAELFPSDVTHLEFKRPKNFDYKAGQW
VRIACLAQSSSEYHPFTLSSAPHEDTLKLHIRAVGPWTRNLRNIYDPNVLRDSPYPKLFLDGPFGEG

HQDWYKYEVSVLVGGGIGVTPFASILKDLVNRSQSGVAITCKAVYFIWVTRDQNQYEWLTDIIQEVE
GKDKKQILNTHIFITQFPQKFDLRTKMLYICEENFQKIAGKSLFTGLRAITHFGRPDFPDFFVTLGE
EHSSVETFGVFSCGPPPMTEGVEKACAKLNKYEGPTFSHHFENF

>ascidian-Ci-Duox-D: DDBJ™ No. BR000264 (Chromosome 1q: 5.10M)

RAADSKEYPSFNGWYNNLANPSSGATETALTRRLKSHYEDGVYAPSGSGRPNPRTLSVEIASGPSG
LHSYDNKTTLFVYFGQHIIKEIVENSRQGCPPEYFWIPVPEGDEIFDPRGDGNIRLPYHRSPYIASNT
GYTPNHPRDQLNEVTSLLDGDSIYGSTKAWSGELRLRKQDCELRSADDDGKLPMRNTIGLPLQNP
VVPVYHEMRNAKRLFATGSRTGNENPFLLTIGVTWFRHHNWLARQLRDNNPDWSDEHVFMESRT
RNIATYQKVFFYEWLPILLGSCPSFPIIPFFKWTGYHASVPVGVSNVFQSAAMNYLDTMVPPGGFIP
KTFDRFVLEASINSDEMDKWRTCNTYWNSDELFENATPFNIVYGMMGQMAESEDHIISEDLRNSF
HGSMEFSRTDKRGRDHGLPDYNTAREDLGLKKKETIRDINPDLFKKDEKLLERLHSLHGGSPSNM
DIYVGGMLESKDGRPGELFRSIILEQMVRLRDGDRFWFENLNNGLFTVDQVNGIFNVTFLDVLLRT
YPELNLAGNTAFNPFIWKSNKHICRQPYQMTGDNMTSCTTMQRQDDFNHNQISFLAIFCGVVFFAI
ACVIFMIYVGSWKEDKRNKEKTKTTSTYSEQDAFTAKEILGRKRHERKDVQVQFAFTYYIQVITQE
NNTTHITHITESEEVVIMYTDSGKRKKMLLRCAKRTYDLILAFTGSSERDIFLTKLRLFFKQNDILW
KERAIHEKHFFEEANTKDERDTELSNFYQAVIAQSMRMEFGIQISSFPENLVDIKLSQEEFASLLQM
KSSSLFVQHIFTTADEDQDGFISFHDFRKIIVLFVKGSPNEKLRLLFDMFDLNQNGSLTKQQFKEM
FVCLADTYQSTVDQNVLENVLSKIVPPKNDEEIQYLNFDDFKKLMDLPEMDEAMLNLTAPGRVILL
LGQNYRFSNQQKFFTISKILKNCHYNNKMKFPTFLQIQFEAIKQLDLEITGSQMFIFIGKNKKNVYF
QNSSKFRLAYRAIRRLFECYALHIFWTSLYIWITIGVFLWAFSMTIFNKAAGLGVIAGNALPLARASA
AALMFNISTLLLTMCKNILTSLRETQLHLYIPFDAAVAFHKLVAWMALFFTALHIIAHGINFYSIQTQT
PSDLLCLFRDLWFPSDYRPTFVFWCLQTLTGNTGVLLTIIFIVMYVFSLDYPRQVMFNWFQWIHFF
GYISVYFFTVLHGSGMLIQIPSFYYYFLVPAILYTFDKLYSVYRKKFQLPVIKAEILPSDVVYLEFVRP
SDFYYKAGQWVRIACVGLSKWEYHPFTLSSSPDEETLQLHIRAVGPWTRNIRNIYKEGEPYPKLYV
DGPFGEGHQDWYKYEVAVLVGGGIGVTPFASILKDLVNKSTVGVGIPCKSVYFLWVARDQRQFEWL
LDIIEETEKNDALGILSTHIFITEIPNKFDLRTTMLYVCEQHFKKVSEKSMFTGLNAVTHFGRPNFP
DFLKTLSWKHSEVKKIGVFSCGPPSMTESVESACLKANKHKGPMYAHHFENF

>nematode-Ce-Duox1: GenBank™ No. NM_058285

MRSKHVLYIAILFSSIFGGKGIQQNEEFQRYDGWYNNLANSEWGSAGSRLHRDARSYYSDGVYSVN
NSLPSARELSDILFKGESGIPNTRGCTTLLAFFSQVVAYEIMQSNGVSCPLETLKIQVPLCDNVFDKE
CEGKTEIPFTRAKYDKATGNGLNSPREQINERTSWIDGSFIYGTTQPWVSSLRSFKQGRLAEGVPG
YPPLNNPHIPLNNPAPPQVHRLMSPDRLFMLGDSRVNENPGLLSFGLILFRWHNYNANQIHREHP
DWTDEQIFQAARRLVIASMQKIIAYDFVPGLLGEDVRLSNYTKYMPHVPPGISHAFGAAAFRFPHSI
VPPAMLLRKRGNKCEFRTEVGGYPALRLCQNWWNAQDIVKEYSVDEIILGMASQIAERDDNIVVE
DLRDYIFGPMHFSRLDVVASSIMRGRDNGVPPYNELRRTFGLAPKTWETMNEDFYKKHTAKVEKL
KELYGGNILYLDAYVGGMLEGGENGPGELFKEIIKDQFTRIRDGDRFWFENKLNGLFTDEEVQMI

HSITLRDIIKATTDIDETMLQKDVFFFKEGDPCPQPFQVNTTGLEPCVPFMQSTYWTDNDTTYVFT

LIGLACVPLICYGIGRYLVNRRIAIGHNSACDSLTTDFANDDCGAKGDIYGVNALEWLQEEYIRQVRI

EIENTTLAVKKPRGGILRKIRFETGQKIELFHSMPNPSAMHGPFVLLSQKNNHHLVIRLSSDRDLSK

FLDQIRQAASGINAEVIIKDEENSILLSQAITKERRQDRLDLFFREAYAKAFNDSELQDSETSFDSSN

DDILNETISREELASAMGMKANNEFVKRMFAMTAKHNEDSLSFNEFLTVLREFVNAPQKQKLQTL

FKMCDLEGKNKVLRKDLAELVKSLNQTAGVHITESVQLRLFNEVLHYAGVSNDAKYLTYDDFNAL

FSDIPDKQPVGLPFNRKNYQPSIGETSSLNSFAVVDRSINSSAPLTLIHKVSAFLETYRQHVFIVFCFV

AINLVLFFERFWHYRYMAENRDLRRVMGAGIAITRGAAGALSFCMALILLTVCRNIITLLRETVIAQY

IPFDSAIAFHKIVALFAAFWATLHTVGHCVNFYHVGTQSQEGLACLFQEAFFGSNFLPSISYWFFSTI

TGLTGIALVAVMCIIYVFALPCFIKRAYHAFRLTHLLNIAFYALTLLHGLPKLLDSPKFGYYVVGPIVL

FVIDRIIGLMQYYKKLEIVNAEILPSDIIYIEYRRPREFKYKSGQWVTVSSPSISCTFNESHAFSIASSP

QDENMKLYIKAVGPWTWKLRSELIRSLNTGSPFPLIHMKGPYGDGNQEWMDYEVAIMVGAGIGVT

PYASTLVDLVQRTSSDSFHRVRCRKVYFLWVCSTHKNYEWFVDVLKNVEDQARSGILETHIFVTQT

FHKFDLRTTMLYICEKHFRATNSGISMFTGLHAKNHFGRPNFKAFFQFIQSEHKEQSKIGVFSCGP

VNLNESIAEGCADANRQRDAPSFAHRFETF

>nematode-Ce-Duox2: GenBank™ NM_058283

MAAENFYNVNNFQSLPLEIKVQFSKETLFSALQQEAETQRYDGWYNNLANSEWGSAGSRLHRDA

RSYYSDGVYSVNNSLPSARELSDILFKGESGIPNTRGCTTLLAFFSQVVAYEIMQSNGVSCPLETLKI

QVPLCDNVFDNECEGKTTIPFYRAKYDKATGNGLNSPREQINERTSWIDGSFIYGTTQPWVSALRS

FKQGRLAEGVPGYPPLNNPHIPLNNPAPPQVHRLMSPDRLFMLGDSRVNENPGLLSFGLILFRWH

NYNANQIYREHPDWTDEQIFQAARRLVIASMQKIIAYDFVPGLLGEDVRLSNYTKYMPHVPPGISH

AFGAAAFRFPHSIVPPAMLLRKRGNKCEFRTEVGGYPALRLCQNWWNAQDIVKEYSVDEIILGMAS

QIAERDDNIVVEDLRDYIFGPMHFSRLDVVASSIMRGRDNGVPPYNELRRTFGLAPKTWETMNED

FYKKHTAKVEKLKELYGGNILYLDAYVGGMLEGGENGPGEMFKEIIKDQFTRIRDGDRFWFENKL

NRLFTDEEVQMIHSITLRDIIKATTDIDETMLQKDVFFFKEGDPCPQPFQVNTIGLEPCAPLIQSTY

WDDNDTTYIYTLIGLACIPLICYSIGHYMVERRIRIGHNSACDSLTTDFSTESPKVNVYKVNALEWL

QEEYIRQVRIEIENTTLTVKKPRGGILRKIRFETGQKIEVFHSIPNPSAMHGPFVLLSQKNNHHLVIR

LSSDRDLSKFLDQIRQAASGINAEVIIKDEENSILLSQAITKERRQDRLDLFFREAYAKAFNDSELQD

SETSFDSSNDDILNETISREELASAMGMKANNEFVKRMFAMTAKHNEDSLSFNEFLTVLREFVNA

PQKQKLQTLFKMCDLEGKNKVLRKDLAELVKSLNQTAGVHITESVQLRLFNDVLHKSGVSDDAEY

LTCNNFDALFSEISDVQPIGLPFNRKNYNSHIKEPSCHTSFPIVDHSTPAPLSLIQRICAFLETYRQH

VFIIFCFVAINIVLFFELFWHSRYLNEDRDLRRVMGAGIAITLSSAGALSFCMALILLTVCRNIITLLR

ETVIAQYIPFDSAIAFHKIVALFTLFWSTLHTIGHCVNFYHVGTQSDRGLACLFQETFFGSDVVPTLS

YWFYGTITGLTGIGLVIVMSIIYVFALPKFTRRAYHAFRLTHLLNIGFYALTILHGLPSLFGSPKFGYY

VVGPIVLFVIDRIIGLMQYYKSLDIAHAEILPSDIIYIEYRRPREFEYKSGQWITVSSPSISCTFNESHA

FSIASSPQDENMKLYIKAVGPWTWKLRSELIRSLNTGSPFPLIHMKGPYGDGNQEWMNYEVAIMV

GAGIGVTPYASTLVDLVQKTSSDSFHRVRCRKVYFLWVCSSHKNFEWFVDMLKNVENQAKPGILE
THIFVTQMFHKFDLRTTMLYICEKHFRATNSGISMFTGLHAKNHFGRPNFKAFFQFIQSEHKEQSE
IGVFSCGPVNLNESIAEGCADANRQRDAPSFAHRFETF

>fruit fly-Dm-Duox: FlyBase No. CG3131

MYSQTEKQRYDGWYNNLAHPDWGSVDSHLVRKAPPSYSDGVYAMAGANRPSTRRLSRLFMRGKD
GLGSKFNRTALLAFFGQLVANEIVMASESGCPIEMHRIEIEKCDEMYDRECRGDKYIPFHRAAYDR
DTGQSPNAPREQINQMTAWIDGSFIYSTSEAWLNAMRSFHNGTLLTEKDGKLPVRNTMRVPLFNN
PVPSVMKMLSPERLFLLGDPRTNQNPAILSFAILFLRWHNTLAQRIKRVHPDWSDEDIYQRARHTVI
ASLQNVIVYEYLPAFLGTSLPPYEGYKQDIHPGIGHIFQAAAFRFGHTMIPPGIYRRDGQCNFKETP
MGYPAVRLCSTWWDSSGFFADTSVEEVLMGLASQISEREDPVLCSDVRDKLFGPMEFTRRDLGAL
NIMRGRDNGLPDYNTARESYGLKRHKTWTDINPPLFETQPELLDMLKEAYDNKLDDVDVYVGGM
LESYGQPGEFFTAVIKEQFQRLRDADRFWFENERNGIFTPEEIAELRKITLWDIIVNSTDVKEEEIQK
DVFMWRTGDPCPQPMQLNATELEPCTYLEGYDYFSGSELMFIYVCVFLGFVPILCAGAGYCVVKL
QNSKRRRLKIRQEALRAPQHKGSVDKMLAREWLHANHKRLVTVKFGPEAAIYTVDRKGEKLRTFS
LKHIDVVSVEESATNHIKKKPYILLRVPSDHDLVLELESYGARRKFVKKLEDFLLLHKKEMTLMEV
NRDIMLARAETRERRQKRLEYFFREAYALTFGLRPGERRRRSDASSDGEVMTVMRTSLSKAEFAAA
LGMKPNDMFVRKMFNIVDKDQDGRISFQEFLETVVLFSRGKTDDKLRIIFDMCDNDRNGVIDKGE
LSEMMRSLVEIARTTSLGDDQVTELIDGMFQDVGLEHKNHLTYQDFKLMMKEYKGDFVAIGLDCK
GAKQNFLDTSTNVARMTSFNIEPMQDKPRHWLLAKWDAYITFLEENRQNIFYLFLFYVVTIVLFVE
RFIHYSFMAEHTDLRHIMGVGIAITRGSAASLSFCYSLLLLTMSRNLITKLKEFPIQQYIPLDSHIQF
HKIAACTALFFSVLHTVGHIVNFYHVSTQSHENLRCLTREVHFASDYKPDITFWLFQTVTGTTGVM
LFIIMCIIFVFAHPTIRKKAYNFFWNMHTLYIGLYLLSLIHGLARLTGPPRFWMFFLGPGIVYTLDKIV
SLRTKYMALDVIDTDLLPSDVIKIKFYRPPNLKYLSGQWVRLSCTAFRPHEMHSFTLTSAPHENFLS
CHIKAQGPWTWKLRNYFDPCNYNPEDQPKIRIEGPFGGGNQDWYKFEVAVMVGGGIGVTPYASIL
NDLVFGTSTNRYSGVACKKVYFLWICPSHKHFEWFIDVLRDVEKKDVTNVLEIHIFITQFFHKFDLR
TTMLYICENHFQRLSKTSIFTGLKAVNHFGRPDMSSFLKFVQKKHSYVSKIGVFSCGPRPLTKSVMS
ACDEVNKTRKLPYFIHHFENFG

>honeybee-Am-Duox: DDBJ™ No. BR000285

VHSYADKQRYDGWYNNLAHPDWGSIAVDSRLIRKMPAAYSDGVYMLAGQDRPSPRKLSQLFMQG
DDGLPSVKNRTALFAFFGQLVTSEIIMASESGCPIEYHRIDVDKCDPVFDKECQGNKYIPFRRADYD
RQTGRSPNSPREQINKVTSWIDGSFVYSSSEAWANTMRSFKNGSLLMEPTRKFPVRNTMRAPLFN
HAVPHVMRMLSPERLYLLGDPRTNQHPPLLALGILFYRWHNVIAARIQLENPTMSDEDIFQKARRV
VIGTLQQNIILYEYIPILLNEDLPPYTGYKSDLHPGISHIFQSAAFRFGHTLIPPGLYRRDENCEYRRT
NTDQPAIRLCSTWWDSNEVLTNSTIEELLMGMTSQIAEKEDNLLGTDIRNNLFGPMEFSRRDLGAL
NIMRGRDNGLPDYNTARAHFKLPRKKTWNEINPELFNKNPSLLRTLVEIHSNNLNNMDVYVGGM
LESSAGPGELFSTVIKEQFLRLRDSDRFWFENEENGIFTRSEIADIRRITLWDVIVNATGIPADSIQRK

VFTWEEGDPCPQPYQLNSTMLEPCVPLQRYDYFEGSELVYIYACVFLGFVPILCAGAGYGLVKLQN
RRRRRLKILQEAIQKRNDGKICVDKMIVREWLHANHRRLVKVKFGPEAALHIVDRKGEKLRTFDF
NDVNTVTMEESQENENGHRKPLVLLRIPRDYDLVLELDSLASRRKFIAKLEAFLASHKKHFTLSQV
SRDIMLAKAETKERRQKKLEQFFREAYALTFGLRPGERRRRSEDSDSGEVVTVMRTSLSKSEFASA
LGMRADAVFVKKMFNIVDKDRDGRISFQEFLDTVLLFSRGKTEDKLRIIFDMCDKDCNGVIDKEEL
SEMLRSLVEIARTTSLSDDHVTELIDGMFQDAGLERKDYLTYNDFKLMMKEYKGDFVAIGLDCKG
AKQNFLDTSTNVARMTSFHIDQLPPEDSKTWAQKQWDAISTFLEENRQNIFYLFVFYVTTIALFVE
RFIYYSFMAEHTDLRHIMGVGIAITRGSAAALSFCYSLLLLTMSRNLLTKLKEFSIQQYIPLDSHIQF
HKIAACTALFFSVLHTVGHMVNFYHVSTQPLAHLRCLTSELSFPSDARLTISFWLFRTVTGLTGILL
FIVMTIIFVFAHPTIRQKAYKFFWSTHSLYVVLYALCLIHGLARLTGSPRFWIFFVGPAIIYALDKVVSL
RTKYMALDIIETELLPSDVIKIKFYRPPNLKYLSGQWVRLSCTAFRSNEFHSFTLTSAPHENFLSCHI
KAQGPWTWKLRNYFDPCNYNPEDEHPKIRIEGPFGGGNQDWYKFEVAVMVGGGIGVTPYASMLN
DLVFGTSTNRYSGVACKKVYFLWICPSHKHFEWFIDVLRDVERKDVTDVLEIHIFITQFFHKFDLRT
TMLYICENHFQRLSKKSIFTGLKAINHFGRPDMTSFLKFVQKKHSYVSKIGVFSCGPRPLTKSVMSS
CDEVNKGRRLPYFIHHFENFG

>mosquito-Ag-Duox: DDBJ™ No. BR000284

LMSHVEKQRYDGWYNNLAHPDWGAVDNHLTRKAPSAYSDGVYYVMAGSNRPSPRKLSRLFMRGTDGLPSM
ENRTALLAFFGQVVTNEIVMASESGCPIEMHRIEIEKCDEMYDRECRGDRYIPFHRAAYDRNTGQSPNAPREQ
INQMTAWIDGSFIYSTSEAWLNAMRSFQDGALLTDKQGTMPVKNTMRVPLFNNPVPHVMRMLSPERLYLLG
DPRTNQNPALLSFAILFLRWHNVVAKRVRRQHRDWSDEEIFQRARRVVIASLQNIVAYEYLPAFLDKEIPPYDG
YKADTHPGVSHMFQAAAFRFGHSLIPPGLFRRDGQCNFRRTNMDFPALRLCSTWWNSNDVLDNTPVEEFIM
GMASQIAEKEDPLLCSDVRDKLFGPMEFTRRDLGALNIMRGRDNGLPDYNTARAAYRLPKKKSWRDINPAV
FERQPELLDLLIKTYDNQLDNVDVYVGGMLESDGRPGELFSAVIIDQFTRIRDADRFWFENEDNGIFTKEEIA
EIRKFTLWDIIVNSTDIEADEIQRDVFHWKQGDPCPQPEQLNATLLEPCNYLEGYDYFSGSELAYIYSCVFLGF
VPILCAGAGYCVIKLQNSRRRKLKIKQEAMKNTANTKVSVEKMVAREWLHANHKRLVTVKFGPEASIYTVD
RKGEKLRTFNLKHVDVVTVEQSQENYTAKKPYILLRVPNDHDLVLELESNSARRKFVKKLEDFLVLHKKTM
TFVESNRDLMLAKAETRERRQKRLEHFFREAYALTFGLRPGERRRRSDASLDGEVMTVMRTSLSKSEFAAAL
GMKQDDMFVRKMFNIVDKDKDGRISFQEFLETVVLFSRGKTDDKLRIIFDMCDNDRNGVIDKGELSEMMRS
LVEIARTTSVTDEQVNELIDGMFQDVGLEHKNHLTYEDFKLMMKEYKGDFVAIGLDCKGAKQNFLDTSTNV
ARMTSFHIEPISDSRRHWMQEKWDCYTTFLEENRQNIFYLFLFYVITIVLFVERFIHYSFMAEHTDLRHIMGV
GIAITRGSAASLSFCYSLLLLTMSRNLLTKLKEFPIQQYIPLDSHIQFHKIAACTALFFSLLHTVGHIVNFYHVST
QSIENLKCLTKEVHFTSDYRPDITYWLFQTITGVTGVMLFVTMCIIFAFAHPTIRKKAYKFFWNAHSLYVVLY
ALCLVHGLARLTGAPRFWLFFIGPGIVYTLDKIVSLRTKYMALDVIETDLLPSDVIKIKFYRPPNLKYLSGQW
VRLSCTEIKPEEMHSFTLTSAPHENFLSCHIKAQGPWTWKLRNYFDPCNYNPDDQPKIRIEGPFGGGNQDWY
KFEVAVMVGGGIGVTPYASILNDLVFGTSTNRYSGVACKKVYFLWICPSHKHFEWFIDVLRDVEKKDVTNVL

EIHIFITQFFHKFDLRTTMLYICENHFQRLSKTSMFTGLKAVNHFGRPDMSSFLKFVQKKHSYVSKIGVFSCGP
RPLTKSVMSACDEVNKSRKWPYFIHHFENFG

>fungus-Pa-NoxA: GenBank™ No. AF364817 (initially termed Nox1)

MGGLVPLLKKQLTGSKILFHILFWTFHWGIFAYGWWKQAADARLAGLNTLQYSVWLSRGAGLVLSVDGM
LILLPVCRTIMRFIRPKIKFIPLDENIWMHRQLAYSMLLFTIIHTAAHYVNFYNVEKTQIRPVTAVQIHYVQPG
GATGHVMLLCMLLMYTTAHHRIRQQSFETFWYTHHLFIPFFLGLYTHTVGCFVRDTADAISPFAGDEYWEH
CIGYLGWRWELWTGGFYLIERLYREIRAIRETKITRVVKHPYDVVEIQFNKPSFKYKAGQWLFLQVPSVSKY
QWHPFTITSCPYDPYVSVHIRQVGDFTRELGNAVGAGGIHAKLYEGVDPLGMYDVALANGQKMPALRIDG
PYGAPAEDVFENEIAVLIGTGIGVTPWASILKNIWHLRNGPNPPTRLRRVEFIWVCKDTSSFEWFQTLLLSLEE
QSAEAARVPGSSGVEFLKIHTYLTQKLDMDTTQNIVLNSVGSSVDPLTELKARTNFGRPNFGRIFQSMSEGIQ
NRTYLNGLEGNMRTTVGVYFCGPSAAARDIKKAAKAASSSEVRFRFWKEHF

> fungus-Pa-NoxB: GenBank™ No. AY372210（initially termed Nox2）

MSGYGGYDGGLRSSGSMKQSERSRWTPLTRMLLSGEMTQERQKELTPREKFDKWMVNEGYRRIFVFVFMF
LHAILFAFSFVNFAVKENLQIARDTFGPTFMIARSAALVLHVDVALILFPVCRTLISMARQTPLNGIIQFDKNIT
FHITTAWSIVFWSWVHTIAHWNNFAQVAAKNNLGIYGWLLANFVSGPGWTGYVMLIALMGMVITSVEKTR
RANYERFWYTHHMFIVFFFFWSIHGAFCMIQPDFAPFCISIGTQAIGVFWQYWMYGGFAYLAERVAREIRGR
HKTYISKVIQHPSNVCEIQIKKEHTKTRAGQYIFFCCPAVSLWQYHPFTLTSAPEEDYISIHMRVVGDFTRAV
AETLGCEFDKKKGDASKVVGVDQSNDEVDPALRRVLPRVYIDGPFGSASEDVFKYEISVLCGAGIGVTPFAS
ILKSIWYRMNYPQKRTRLSKVYFFWICRDFGSFEWFRSLLLAIEAQDVDNRIEIHTYLTAKIKVDDATNIMIN
DANADKDTITGLRSPTNFGRPNWDMIFRGIRKLHTPAEAGVFFCGPKGLGSQLHVFCNKYSEPGFNFVWGK
ENF

> fungus-An-NoxA: GenBank™ No. AY174088

MGRYPLKSYFAPSKLFFYTWFWGAHIAIFAYGWYHQAKSEPLSPLNVLSYSVWISRGAGLVLTVDGTLILLP
MCRNLVRFLRPKLRWLPLDENIWFHRQVAYATLVFTILHVAAHYVNFYNIERKQLRPETALQIHYAQPAGV
TGHVMLFCMMLMYTTAHHRIRQQSFETFWYTHHLFIPFLLGLYTHATGCFVRDSAEPYSPFAGERFWKHCI
GYQGWRWELVAGFFYLCERLWREIRALRETEIVKVVRHPYDAMEIQFRKPGFKYKPGQWLFIQVPEVSNTQ
WHPFTITSCPFDDYVSIHVRQVGDFTRALGDALGCGPAQARDLEGLDPMGMYEVALQNGQQMPKLRVDGP
YGAPAEDVFENEIAVLIGTGIGVTPWASILKNIWHLRASPDPPRRLRRVEFIWVCKDTTSFEWFQALLSSLEA
QSASDAAYQGVSEFLRIHIYLTQRLDQDTTTNIYLNSVGQELDPLTELKSRTNFGRPDFKRLFTAMRNGLQD
QSYMRGLHTHSRTEIGVYFCGPNVAARQIKAAASSASTNEVKFKFWKEHF

> fungus-Mg-NoxA: GenBank™ No. EAA49092

MSVGEFLAKQLTAQKLFFNISFWGFHIGIFAYGWYKQFSDPRLAGLNTLTFSVWISRGAGLVLSVDGMLILL
PVCRTIMRWIRPKIRFIPLDENIWFHRQIAYAMLIFSITHTAAHYVNFFNVERLQIRAQTAVQIHYAQPGGATG
HMMLLCMLLMYTTAHHRIRQQSFETFWYTHHLFIPFFLGLYTHTVGCFVRDTVEPHSPFAGDEYWNHCIGY
LGWRWELWTGGFYLLERLYREIRARRETKITRVVRHPYGEFFFFLFFLLFGPSMVGFSVLIRDSADVVEIQFN
KPSFKYKAGQWLFLQVPSVSKYQWHPFTITSCPYDPYVSVHVRQVGDFTKALGDATGAGAAQAKLYEGVD

PMGMYEVALQNGQQMPMLRIDGPYGAPAEDVFENEIAVLIGTGIGVTPWASILKNIWHLRNGPNPPTRLRR
VEFLWVCKDTSSFEWFQTLLSSLEQQSTDAAGLPGGNGVEFLKIHSYLTQKLDMDTTQNIVLNSVGAALDP
LTELKSRTNFGRPNFAKLFASMRDGIMDRTYLSGLEGSMKTTVGVYFCGPSAAARDIKAACKTASVNEVEF
RFWKEHF

> fungus-Mg-NoxB: GenBank™ No. EAA56588

MSGYGYGGGGGGFRDSYGSEKGQSERSRWTPLTRMLLSGEMTQERQKELTPREKFDRWMVNEGYRRFFV
FVFMILHAMVFAFGFVNYAVKDNLQRARDTFGPTFMIARAAALVLHFDVALILFPVCRTFISLARQTPLNGII
QFDKNITFHITTAWSIVFFSWVHTVAHWNNFAQIAAQQKLGIYGWLLANFVSGPGWTGYVMLIALMGMVF
TSVEKPRRANYERFWYTHHFFIVFFFFWSIHGAFCMIQPDFAPFCMSFGTSAIGVFWQFWMYGGFVYMAERI
AREIRGKHKTYISKVIQHPSNVCEIQIKKEHTKTRAGQYIFFCCPEVSVWQYHPFTLTSAPEEDYISIHMRVVG
DFTRGVSKALGCDWDRKGDASKVVGVNGENPDVDPALKRVLPRVYVDGPFGSASEDVFKYEIAVLCGAGI
GVTPFASILKSIWYRMNYPQKKTRLAKVYFFWICRDFGSFEWFRSLLLAIEAQDVDNRIEIHTYLTAKIKADD
ATNIMINDANADKDAITGLRAPTNFGRPNWDMIFRGIRKLHTPAEAGVFFCGPKGLGSTLHIFCNKYSEPDFA
FVWGKENF

> fungus-Mg-NoxC: GenBank™ No. EAA57330

MAKSTTTDGENVKANSENGSTAAASPNDEHVKIEPSTKDKGEDKPKAADARDDGSEAEARADQHPEKPAQ
ANGPETADGDHHPFANSYQVDSARAADDPNVVYLRDDEIETFLDELDHNGDGCIDYSEVERKLDEVHDEL
APTAQPHHLHHDSKQDRERHVFLRSVIGSDQDRIPRADFARVVKSWRVPSMKQEGDKKDDQDKYLRDMST
FRRVRAYWAVHGPEIAFLGIVVGLQLGLGIWQCHKYASGEQYQAAFGWGVALAKLCAGALYPTFFFLILS
MSRYFSTFLRRSYHLSRFINWDLSQEFHIKISIVALVLASLHALGHLSGTFNWGSRPERQDAVGVLLGEDQVP
RPYSAYVSSLPGITGLTALGLFYTLALLSMPQVRRWNYEVFQLAHLLMFPIIGLLAAHGTAQLLQYAMFGY
WLAVPTILVLTERLVRVGTGFHRIPASLKVLDDETVELRATIPSERIWKYQAGQWAYLQVPTISMWQWHPF
TISVCVGKEMRMHIKTDGNWTGRLRDLAKDAPQGQEVDIEIGINGPFGAPAQRFYDFNHTILVGAGIGLTPF
SGILADLQAKEDRLHGGPTQKLQEQAEKGGDVRGSTSADAPDGRRIGTSREAEAMPQRTDVDMQAPTSGD
QSNTTLQETADADSDSASSISRPSSSFSSFASDYRRVDFHWMVRDRNHLLWISELLNTVSRSQAWHHRHDAP
GEYHLDIRMQTHVTQKRKNVSTHVYRWLLEQHRTPEHPASPITGLINPTQFGRPDFVSILDRHYDDMRKYK
AGLVARASRGGGGEDDAASVADDEVKVGVFFCGTPIVGEILADRCKALTARGRHDGSKIEYHFMIEVFN

> fungus-Fg-NoxA: GenBank™ No. XM_380915

MGSQLGFVELIKKQFVPGKLLYHFLFWTFHWGIFAYGWWKQAVDPRLAGLNTLKFSVWISRGAGLVLSVD
CMLILLPVCRTVMRWVRPKIRFLPLDENLWMHRQLAYSMLLFTCLHTGAHYVNFYNVEITQIRPVTALQIH
YAQPGGITGHIMLLCMLLMFTSAHARIRQQSFETFWYTHHLFIPFFLGLYTHTVGCFVRDTPEAFSPFAGDEF
WEHCIGYLGWRWELWTGGAYLLERLWREVRARRSTKITRVVRHPYDVVEIQFNKPSFKYKAGQWLFLQVP
SLSKYQWHPFTITSCPFDPYVSVHVRQVGDFTRELGDALGAGAAQAKLYDDVDPMGMYEVALQNGDQMP
ALRIDGPYGAPAEDVFENEIAVLIGTGIGVTPWAAILKNIWHLRNSPNPPRRLRRVEFIWVCKDTGSFEWFQT
LLSSLEEQSNEAARMPGSTGVEFLKIHTYLTQKLDIDTAQNIVLNSVGSQMDPLTELQSRTNFGRPDFPRLFT
TMRNGILDRTYLNGLESHIRTTVGVYFCGPSAAARDIKLACKAATVPDVDFRFWKEHF

> fungus-Fg-NoxB: GenBank™ No. XM_390983

MQDWSEKPSERSRWTPLTRMLLSGEMTQEKQQELSSREKFDRWMINEGYRRFFVFVFMILHALIFSFACVH
YAQKESLETSRQTFGFTFIIARSAALVLHVDVAIILFPVCRTLISLLRQTPLNGILQFDKNITFHIVTAWSIVFWS
WVHTIAHWNNFAQVAIKYNLGIYGWLLANFVSGPGWTGYVMLIALMGMVLTSMEKPRRANFERFWYTHH
MFIVFFFFWSIHGAFCMIQPDVAPFCTSIGSSAIGVFWQFWMYSGFCYLAERIAREVRGRHRTFISKVIQHPSN
VCEIQMKKEHTKTRAGQYIFLCCPAVSLWQYHPFTLTSAPEEDYISIHMRCVGDFTKELAKSLGCDWSKKK
DAGDASKVVGLTGREAEIDPAIRRVLPRVYVDGPFGSASEDVFKYEVSVLVGAGIGVTPFASILKSIWYRMN
YPQKKTRLSKVYFFWICRDFDSFEWFRSLLLAVEAQDLDHRIEIHTYLTARIKADDATNIMINDANADKDTIT
GLRSPTNFGRPNWDMIFRGIRKIHSPAEAGVFYGGPKGLGSSLHTYCNKYTEPGFSFVWGKENF

>fungus-Fg-NoxC: GenBank™ No. XM_391371

MAAAHRHRKSVAADHPVRSSQNVEFLTDKEISDFLDDLDHDNDGHINYEEVERKLDQEHANLVP
KPSAHHVISTDHSDDDRTRHAFLRRMMGDSGVDQIPRDEFAKMVKEWKIPSLKQAKKEEEEDKS
YIKRLPGWRRIRSYWAVHGPEIVFLGVVISMQLAFGIWQLVKYQTTPGYRAAFGWGVVMAKTCAGA
LYPTFFFLILSMSRYFSTWLRRSYHISRFFNWDLSQEFHIRISCVAILLATLHAIGHLTGSFVHGSDPA
NEDAVAEALGPDKVPRPYIDYVRSLPGFTGITALGLFWILCLLSIPQVRRWNYEVFQLGHLLMFPIIG
LMMAHGTAALLQWPMFGYFLAFPTLLVLVERTVRVGLGFHRIKATMKVLDKETVEVTAIIPSERLW
KYKAGQYIFLQVPKISFFQWHPFTVSFCRGNKMMLHIKTDGNWTAKLRELGGDSGESEIEVGING
PFGAPAQRFYDFNHSIIIGAGIGVTPFSGILADLQYNDDLDHGGPNHEVDHHRHDSEATAIPQAARR
SDSSSSDEATTSDNVPETPTRQGSVGPDLINKEKQPQADKAGSFAEDYRRVDFHWMVRERNYLLW
LSDLLNDVSMSQDWHREHEDKPHLDIRINTHVTAKQKKISTHVYRWLLEMHRTDEHPASPLTGLL
NPTHFGRPDFDLILDEHYEEMLKFRASKRTSTRNKEDENYEEDEELKVGVFYCGAPVVGEILADK
CRELTLRGWQDGIHELGRIEWLVNYDGYDDHIN

>alga-Cc-NoxD: GenBank™ No. AAZ73480 (insertion of TM7-TM10 in Nox domain, underlined)

MIPRSKPDVARPSARIEAYLSTHAFKVLFFAFYGAAVTLMFAWGFKAEFTFEDNFDMPHFNTVRWFIGIARG
MGYTLNLNTAFVILLASRLLFTKLRDSPLQLVLPFDAAFPALHIVVGYTIFFAVLVHGSFHFVWLITWDAWT
WGLWSFNMSVITGFLLAIVFGTMLVLARPSVRKNNFRLFYAVHIIGATLFFGLLIIHGMFRQVPYTYKWVIPP
LILYAIDRFLRRRKVSAVELFLSAENAVLKDGDILELRVPKAFSYQAGQYAEVQVPFINREWHPFTIASAPQD
KTMCFYIKALGDWTKELRGAFQARVDGAVTDSLQVNIRGPYGAPAQHVGLYERVVLISGGIGSTPFTSICKD
LHHRKVKENATSATGFEPSTSTLLKRIESRVSTAISTLYGVDISNAKDINQEEEEKRVYLANMLNLTAPGSGS
SSGETTELEVEMVDASKQADESSSDSDRSTSMESYNVKNMLRKEQDEEYILDDIKNSANARRGNRERLSHL
YEGRSKVLEFLHTSRVNLLLLFVLIARIFFICISSIIKADYIMINAEPHAIESGLWIVIVDTVLSIIFAIVLPLTIFLE
LSYMGSRFFRTVGRTLDFFVFLPLTITSASLGIKALVTERTDEQIVLFLHYIVFLPTLFVLLAVRMYRALGKRT
LLTDAPCHCSHRDIVPNVDFVWTVPHENDDEWLRSELEPLADGTELKLHRYVTRAKEVDMEAGSEFITSSN
TGRPEWDEIFGKIAAEAPSNSVVGVFFCGPHKMGDSVQSAMRRAEINSNLRGAYLRSTKEKTLMKDLGLPQ
RGLIKMLMGTGCSVRFVFREENFG

>alga-Py-NoxD: GenBank™ No. ABA18724 (insertion of TM7-TM10 in Nox domain, underlined)

MGDDKPPPPKSRVSRVESYLSTNGFVLTFLGLYILANVILFFFAATPERRLWPVGHYRRNLTPVARGAGNLIN
FNSAVILLVSARKFMSWLRNTPLNMVVPFDKAMPAFHMLVGRVFLAASVVHVGFHLPVYVVSKPWGPGY
NGFTQLFITGSMLVALFAILFVTSVRVNRSKRYELFWYSHAICASLGFVLLMIHGLHYGVYWTYRWAAGPM
AVYIIDRLMRRVEQKEVRMEVSRDVGAIKGNSMLCLRLPRSFTYEPGQYAEVKVPAISSVQWHPFTIASAPH
EPELVFYIKKSGDWTTNLHAMFASTDPTQVEIKVRGPYGSPAQHVGQFENVVLIGGGVGSTPFASVVKSAH
NWMAASSTRGPEMSPSSSFNAAAGQVSVPAATARDATTVPASASLSARLTMQHSTSAATVTAQARMPTVD
SLADMDEDLSSNGSRIPAARLSDDAALRPPQAPSSFTSDVLERRVAELDRLYSIADKEDNDRVFPPPSSVQSV
VEQDMTGHAVDMDGEDGALSDDDDSSRLDEEEEDQLQSQQSIGRVLRHSAFINSTAGQQLIGLALDADAD
VVKQRAAAKEGANRRTSVLGAFFGGLKGGDDRRGTLPSEVVRARTKRVAVLQILHSVSVSLALLWAMVA
RFAIVALASIMRGFSPSTAGLAMFNTRGLVVADLVLASACAVPLAVSLGCEASILGVSVYFRQRGSLVDTLF
LLPLLLAGVITDALALAGHGRSAAWFASVNLLVLWPLLLFALLFRLTRVVGSRLVLAQNLQSSHSLRSLDFV
WTSPSPEHDAWLVEELLPISRSGTVRLHRHITRSAAEVEPWMLDYDEVPLKTTYKRPDWAAIFAGITERSRS
GSVVGVFFCGPHPMSKSIQDGIARATALSLARGYRRGAIGLDGSREMRT

>At-rbohA: GenBank™ No. NM_120821

MMNRSEMQKLGFEHVRYYTESPYNRGESSANVATTSNYYGEDEPYVEITLDIHDDSVSVYGLKSP
NHRGAGSNYEDQSLLRQGRSGRSNSVLKRLASSVSTGITRVASSVSSSSARKPPRPQLAKLRRSKSR
AELALKGLKFITKTDGVTGWPEVEKRFYVMTMTTNGLLHRSRFGECIGMKSTEFALALFDALARR
ENVSGDSININELKEFWKQITDQDFDSRLRTFFAMVDKDSDGRLNEAEVREIITLSASANELDNIRR
QADEYAALIMEELDPYHYGYIMIENLEILLLQAPMQDVRDGEGKKLSKMLSQNLMVPQSRNLGAR
FCRGMKYFLFDNWKRVWVMALWIGAMAGLFTWKFMEYRKRSAYEVMGVCVCIAKGAAETLKLN
MAMILLPVCRNTITWLRTKTKLSAIVPFDDSLNFHKVIAIGISVGVGIHATSHLACDFPRLIAADEDQ
YEPMEKYFGPQTKRYLDFVQSVEGVTGIGMVVLMTIAFTLATTWFRRNKLNLPGPLKKITGFNAF
WYSHHLFVIVYSLLVVHGFYVYLIIEPWYKKTTWMYLMVPVVLYLCERLIRAFRSSVEAVSVLKVAV
LPGNVLSLHLSRPSNFRYKSGQYMYLNCSAVSTLEWHPFSITSAPGDDYLSVHIRVLGDWTKQLRS
LFSEVCKPRPPDEHRLNRADSKHWDYIPDFPRILIDGPYGAPAQDYKKFEVVLLVGLGIGATPMISI
VSDIINNLKGVEEGSNRRQSPIHNMVTPPVSPSRKSETFRTKRAYFYWVTREQGSFDWFKNVMDE
VTETDRKNVIELHNYCTSVYEEGDARSALITMLQSLNHAKHGVDVVSGTRVMSHFARPNWRSVFK
RIAVNHPKTRVGVFYCGAAGLVKELRHLSLDFSHKTSTKFIFHKENF

>At-rbohB: GenBank™ No. NM_202070

MREEEMESSSEGETNKISRCKATGSDNPDEDYVEITLEVRDETINTMKAKATLRSVLSGRLKTMVK
SLSFASRRLDRSKSFGAMFALRGLRFIAKNDAVGRGWDEVAMRFDKLAVEGKLPKSKFGHCIGMV
ESSEFVNELFEALVRRRGTTSSSITKTELFEFWEQITGNSFDDRLQIFFDMVDKNLDGRITGDEVKE
IIALSASANKLSKIKENVDEYAALIMEELDRDNLGYIELHNLETLLLQVPSQSNNSPSSANKRALNK
MLSQKLIPTKDRNPVKRFAMNISYFFLENWKRIWVLTLWISICITLFTWKFLQYKRKTVFEVMGYC
VTVAKGSAETLKFNMALILLPVCRNTITWLRTKSKLIGSVVPFDDNINFHKVVAFGIAVGIGLHAISH
LACDFPRLLHAKNVEFEPMKKFFGDERPENYGWFMKGTDGWTGVTMVVLMLVAYVLAQSWFRR

NRANLPKSLKRLTGFNAFWYSHHLFVIVYVLLIVHGYFVYLSKEWYHKTTWMYLAVPVLLYAFERL
IRAFRPGAKAVKVLKVAVYPGNVLSLYMSKPKGFKYTSGQYIYINCSDVSPLQWHPFSITSASGDDY
LSVHIRTLGDWTSQLKSLYSKVCQLPSTSQSGLFIADIGQANNITRFPRLLIDGPYGAPAQDYRNYD
VLLLVGLGIGATPLISIIRDVLNNIKNQNSIERGTNQHIKNYVATKRAYFYWVTREQGSLEWFSEVM
NEVAEYDSEGMIELHNYCTSVYEEGDARSALITMLQSLHHAKSGIDIVSGTRVRTHFARPNWRSVF
KHVAVNHVNQRVGVFYCGNTCIIGELKRLAQDFSRKTTTKFEFHKENF

>At-rbohC: GenBank™ No. NM_124485

MSRVSFEVSGGYHSDAEAGNSGPMSGGQLPPIYKKPGNSRFTAENSQRTRTAPYVDLTVDVQDDT
VSVHSLKMEGGSSVEESPELTLLKRNRLEKKTTVVKRLASVSHELKRLTSVSGGIGGRKPPRPAKL
DRTKSAASQALKGLKFISKTDGGAGWSAVEKRFNQITATTGGLLLRTKFGECIGMTSKDFALELFD
ALARRRNITGEVIDGDQLKEFWEQINDQSFDSRLKTFFDMVDKDADGRLTEDEVREIISLSASANN
LSTIQKRADEYAALIMEELDPDNIGYIMLESLETLLLQAATQSVITSTGERKNLSHMMSQRLKPTFN
RNPLKRWYRGLRFFLLDNWQRCWVIVLWFIVMAILFTYKYIQYRRSPVYPVMGDCVCMAKGAAET
VKLNMALILLPVCRNTITWLRNKTRLGRVVPFDDNLNFHKVIAVGIIVGVTMHAGAHLACDFPRLL
HATPEAYRPLRQFFGDEQPKSYWHFVNSVEGITGLVMVLLMAIAFTLATPWFRRGKLNYLPGPLK
KLASFNAFWYTHHLFVIVYILLVAHGYYLYLTRDWHNKTTWMYLVVPVVLYACERLIRAFRSSIKAV
TIRKVAVYPGNVLAIHLSRPQNFKYKSGQYMFVNCAAVSPFEWHPFSITSAPQDDYLSVHIRVLGD
WTRALKGVFSEVCKPPPAGVSGLLRADMLHGANNPDFPKVLIDGPYGAPAQDYKKYEVVLLVGLG
IGATPMISIVKDIVNNIKAKEQAQLNRMENGTSEPQRSKKESFRTRRAYFYWVTREQGSFDWFKNI
MNEVAERDANRVIEMHNYCTSVYEEGDARSALIHMLQSLNHAKNGVDIVSGTRVMSHFAKPNWR
NVYKRIAMDHPNTKVGVFYCGAPALTKELRHLALDFTHKTSTRFSFHKENF

>At-rbohD: GenBank™ No. AF055357

MKMRRGNSSNDHELGILRGANSDTNSDTESIASDRGAFSGPLGRPKRASKKNARFADDLPKRSNS
VAGGRGDDDEYVEITLDIRDDSVAVHSVQQAAGGGGHLEDPELALLTKKTLESSLNNTTSLSFFRST
SSRIKNASRELRRVFSRRPSPAVRRFDRTSSAAIHALKGLKFIATKTAAWPAVDQRFDKLSADSNGLL
LSAKFWECLGMNKESKDFADQLFRALARRNNVSGDAITKEQLRIFWEQISDESFDAKLQVFFDMV
DKDEDGRVTEEEVAEIISLSASANKLSNIQKQAKEYAALIMEELDPDNAGFIMIENLEMLLLQAPNQ
SVRMGDSRILSQMLSQKLRPAKESNPLVRWSEKIKYFILDNWQRLWIMMLWLGICGGLFTYKFIQY
KNKAAYGVMGYCVCVAKGGAETLKFNMALILLPVCRNTITWLRNKTKLGTVVPFDDSLNFHKVIA
SGIVVGVLLHAGAHLTCDFPRLIAADEDTYEPMEKYFGDQPTSYWWFVKGVEGWTGIVMVVLMAI
AFTLATPWFRRNKLNLPNFLKKLTGFNAFWYTHHLFIIVYALLIVHGIKLYLTKIWYQKTTWMYLA
VPILLYASERLLRAFRSSIKPVKMIKVAVYPGNVLSLHMTKPQGFKYKSGQFMLVNCRAVSPFEWHP
FSITSAPGDDYLSVHIRTLGDWTRKLRTVFSEVCKPPTAGKSGLLRADGGDGNLPFPKVLIDGPYG
APAQDYKKYDVVLLVGLGIGATPMISILKDIINNMKGPDRDSDIENNNSNNNSKGFKTRKAYFYWV
TREQGSFEWFKGIMDEISELDEEGIIELHNYCTSVYEEGDARVALIAMLQSLQHAKNGVDVVSGTR
VKSHFAKPNWRQVYKKIAVQHPGKRIGVFYCGMPGMIKELKNLALDFSRKTTTKFDFHKENF

>At-rbohE: GenBank™ No. NM_101781

MKLSPLSFSTSSSFSHADGIDDGVELISSPFAGGAMLPVFLNDLSRNSGESGSGSSWERELVEVTLE
LDVGDDSILVCGMSEAASVDSRARSVDLVTARLSRNLSNASTRIRQKLGKLLRSESWKTTTSSTAGE
RDRDLERQTAVTLGILTARDKRKEDAKLQRSTSSAQRALKGLQFINKTTRGNSCVCDWDCDCDQM
WKKVEKRFESLSKNGLLARDDFGECVGMVDSKDFAVSVFDALARRRRQKLEKITKDELHDFWLQI
SDQSFDARLQIFFDMADSNEDGKITREEIKELLMLSASANKLAKLKEQAEEYASLIMEELDPENFG
YIELWQLETLLLQRDAYMNYSRPLSTTSGGVNNWQRSWVLLVWVMLMAILFVWKFLEYREKAAF
KVMGYCLTTAKGAAETLKLNMALVLLPVCRNTLTWLRSTRARACVPFDDNINFHKIIACAIAIGILV
HAGTHLACDFPRIINSSPEQFVLIASAFNGTKPTFKDLMTGAEGITGISMVILTTIAFTLASTHFRRN
RVRLPAPLDRLTGFNAFWYTHHLLVVVYIMLIVHGTFLFFADKWYQKTTWMYISVPLVLYVAERSL
RACRSKHYSVKILKVSMLPGEVLSLIMSKPPGFKYKSGQYIFLQCPTISRFEWHPFSITSAPGDDQL
SVHIRTLGDWTEELRRVLTVGKDLSTCVIGRSKFSAYCNIDMINRPKLLVDGPYGAPAQDYRSYDVL
LLIGLGIGATPFISILKDLLNNSRDEQTDNEFSRSDFSWNSCTSSYTTATPTSTHGGKKKAVKAHFY
WVTREPGSVEWFRGVMEEISDMDCRGQIELHNYLTSVYDEGDARSTLIKMVQALNHAKHGVDILS
GTRVRTHFARPNWKEVFSSIARKHPNSTVGVFYCGIQTVAKELKKQAQDMSQKTTTRFEFHKEHF

>At-rbohF: GenBank™ No. NM_105079

MKPFSKNDRRRWSFDSVSAGKTAVGSASTSPGTEYSINGDQEFVEVTIDLQDDDTIVLRSVEPATAI
NVIGDISDDNTGIMTPVSISRSPTMKRTSSNRFRQFSQELKAEAVAKAKQLSQELKRFSWSRSFSGN
LTTTSTAANQSGGAGGGLVNSALEARALRKQRAQLDRTRSSAQRALRGLRFISNKQKNVDGWNDV
QSNFEKFEKNGYIYRSDFAQCIGMKDSKEFALELFDALSRRRRLKVEKINHDELYEYWSQINDESF
DSRLQIFFDIVDKNEDGRITEEEVKEIIMLSASANKLSRLKEQAEEYAALIMEELDPERLGYIELWQ
LETLLLQKDTYLNYSQALSYTSQALSQNLQGLRGKSRIHRMSSDFVYIMQENWKRIWVLSLWIMIM
IGLFLWKFFQYKQKDAFHVMGYCLLTAKGAAETLKFNMALILFPVCRNTITWLRSTRLSYFVPFDD
NINFHKTIAGAIVVAVILHIGDHLACDFPRIVRATEYDYNRYLFHYFQTKQPTYFDLVKGPEGITGIL
MVILMIISFTLATRWFRRNLVKLPKPFDRLTGFNAFWYSHHLFVIVYILLILHGIFLYFAKPWYVRTT
WMYLAVPVLLYGGERTLRYFRSGSYSVRLLKVAIYPGNVLTLQMSKPTQFRYKSGQYMFVQCPAVS
PFEWHPFSITSAPEDDYISIHIRQLGDWTQELKRVFSEVCEPPVGGKSGLLRADETTKKSLPKLLID
GPYGAPAQDYRKYDVLLLVGLGIGATPFISILKDLLNNIVKMEEHADSISDFSRSSEYSTGSNGDTPR
RKRILKTTNAYFYWVTREQGSFDWFKGVMNEVAELDQRGVIEMHNYLTSVYEEGDARSALITMVQ
ALNHAKNGVDIVSGTRVRTHFARPNWKKVLTKLSSKHCNARIGVFYCGVPVLGKELSKLCNTFNQ
KGSTKFEFHKEHF

>At-rbohG: GenBank™ No. NM_118641

MQRVSFEVKDTEAEKSSSEILSGSLPSTYRNPAMENVGNAVDDGSSVKNNPKLDMQKQNGLVKW
FKKCLTMVSGESKAPRLDRSKSTAGQALKGLKIISKTDGNAAWTVVEKRYLKITANTDGLLLRSKF
GECIGMNSKEFALELFDALARKSHLKGDVITETELKKFWEQINDKSFDSRLITFFDLMDKDSDGRL
TEDEVREIIKLSSSANHLSCIQNKADEYAAMIMEELDPDHMGYIMMESLKKLLLQAETKSVSTDIN

SEERKELSDMLTESLKPTRDPNHLRRWYCQLRFFVLDSWQRVWVIALWLTIMAILFAYKYIQYKNR
AVYEVLGPCVCLAKGAAETLKLNMALILLPVCRNTITWLRNKTRLGVFVPFDDNLNFHKVIAVGIAI
GVAIHSVSHLACDFPLLIAATPAEYMPLGKFFGEEQPKRYLHFVKSTEGITGLVMVFLMVIAFTLAM
PWFRRGKLEKKLPGPLKKLASFNAFWYTHHLFVIVYILLVLHGYYIYLNKEWYKKTTWMYLAVPV
ALYAYERLIRAFRSSIRTVKVLKMAAYPGKVLTLQMSKPTNFKYMSGQYMFVNCPAVSPFEWHPFSI
TSTPQDDYLSVHIKALGDWTEAIQGVFSEVSKPPPVGDMLNGANSPRFPKIMIDGPYGAPAQDYKK
YEVVLLIGLGIGATPMISIIKDIINNTETKEQLSQMEKGSPQEQQGNKETFKTRRAYFYWVTKEQGT
FDWFKNIMNEIAERDKSKVIELHNHCTSVYEEGDVRSALIRMLQSLNYAKNGLDIVAGTRVMSHFA
RPNWKNVYKQIAMDHPGANVGVFYCGAPVLTKELRQLALEFTHKTSTRFSFHKENF

>At-rbohH: GenBank™ No. NM_125394

MKSNTPTEDSTKWMLESVEIDSMGESSSKEPEINLNKNEGGLKKNASRNLGVGSIIRTLSVSNWR
KSGNLGSPSTRKSGNLGPPTNAVPKKTGPQRVERTTSSAARGLQSLRFLDRTVTGRERDAWRSIEN
RFNQFSVDGKLPKEKFGVCIGMGDTMEFAAEVYEALGRRRQIETENGIDKEQLKLFWEDMIKKDL
DCRLQIFFDMCDKNGDGKLTEEEVKEVIVLSASANRLGNLKKNAAAYASLIMEELDPDHKGYIEM
WQLEILLTGMVTNADTEKMKKSQTLTRAMIPERYRTPMSKYVSVTAELMHENWKKLWVLALWAII
NVYLFMWKYEEFMRNPLYNITGRCVCAAKGAAETLKLNMALILVPVCRKTLTILRSTFLNRVVPFD
DNINFHKVIAYMIAFQALLHTALHIFCNYPRLSSCSYDVFLTYAGAALGNTQPSYLGLMLTSVSITGV
LMIFFMGFSFTLAMHYFRRNIVKLPKPFNVLAGFNAFWYAHHLLVLAYILLIIHGYYLIIEKPWYQK
TTWMYLAVPMLFYASERLFSRLLQEHSHRVNVIKAIVYSGNVLALYVTKPPGFKYKSGMYMFVKCP
DLSKFEWHPFSITSAPGDDYLSVHIRALGDWTTELRSRFAKTCEPTQAAAKPKPNSLMRMETRAA
GVNPHIEESQVLFPKIFIKGPYGAPAQNYQKFDILLLVGLGIGATPFISILKDMLNHLKPGIPRSGQK
YEGSVGGESIGGDSVSGGGGKKFPQRAYFFWVTREQASFDWFKGVMDDIAEYDKTHVIEMHNYLT
SMYEAGDARSALIAMVQKLQHAKNGVDIVSESRHSLSSYKYLNYISTP

> At-rbohI: GenBank™ No. AL161531

MSMSFSGGTHNDRWGSDLASAGEFTQSFPSLPATYSPSPSSSSSSGEELLEVTIEFPSGVIINIDSVT
GTGTDISGTDLEITSCSDSGSGSRSLSLGWSASSERLTAGTNSKQQIQKISRRGYGYSSRSAPEPVVP
HRGEITDSVNLPRALSQRPTRPNRDGSGTERAIHGLKFISSKENGIVDWNDVQNNFAHLSKDGYLF
KSDFAHCIGLENENSKEFADELFDALCRRRRIMVDKINLQELYEFWYQITDESFDSRLQIFFNMYC
YQLSSNLVKHIDQHIIILSASANNLSRLRERAEEYAALIMEELAPDGLYSQYIELKDLEILLLEKDISH
SYSLPFSQTSRALSQNLKDRRWRMSRNLLYSLQDNWKRIWVLTLWFVIMAWLFMWKCYQYKHKD
AFHVMGYCLVMAKGAAETLKFNMALILLPVCRNTITYLRSTALSHSVPFDDCINFHKTISVAIISAM
LLHATSHLACDFPRILASTDTDYKRYLVKYFGVTRPTYFGLVNTPVGITGIIMVAFMLIAFTLASRRC
RRNLTKLPKPFDKLTGYNAFWYSHHLLLTVYVLLVIHGVSLYLEHKWYRKTVWMYLAVPVLLYVG
ERIFRFFRSRLYTVEICKVVIYPGNVVVLRMSKPTSFDYKSGQYVFVQCPSVSKFEWHPFSITSSPGD
DYLSIHIRQRGDWTEGIKKAFSVVCHAPEAGKSGLLRADVPNQRSFPELLIDGPYGAPAQDHWKYD
VVLLVGLGIGATPFVSILRDLLNNIIKQQEQAECISGSCSNSNISSDHSFSCLNSEAASRIPQTQRKTL

NTKNAYFYWVTREQGSFDWFKEIMNEIADSDRKGVIEMHNYLTSVYEEGDTRSNLLTMIQTLNHA
KNGVDIFSGTKVRTHFGRPKWKKVLSKISTKHRNARIGVFYCGVPSLGKELSTLCHEFNQTGITRF
DFHKEQF

>At-rbohJ: GenBank^{TM} No. NM_114450

MKNNKKVGTEDSTKWMLESVEIDPKGDSSVKQPESTINSNNPESSGAGGGILKNVSKNLAVGSIIRSMSVNK
WRKSGNLGSPSTRKSGNLGPPLPVSQVKRPGPQRVERTTSSAARGLQSLRFLDRTVTGRERDSWRSIENRFN
QFAVDGRLPKDKFGVCIGMGDTLEFAAKVYEALGRRRQIKTENGIDKEQLKLFWEDMIKKDLDCRLQIFFD
MCDKDGDGKLTEEEVKEVIVLSASANRLVNLKKNAASYASLIMEELDPNEQGYIEMWQLEVLLTGIVSNAD
SHKVVRKSQQLTRAMIPKRYRTPTSKYVVVTAELMYEHWKKIWVVTLWLAVNVVLFMWKYEEFTTSPLY
NITGRCLCAAKGTAEILKLNMALILVPVLRRTLTFLRSTFLNHLIPFDDNINFHKLIAVAIAVISLLHTALHMLC
NYPRLSSCPYNFYSDYAGNLLGAKQPTYLGLMLTPVSVTGVLMIIFMGISFTLAMHYFRRNIVKLPIPFNRLA
GFNSFWYAHHLLVIAYALLIIHGYILIIEKPWYQKTTWMYVAIPMVLYASERLFSRVQEHNHRVHIIKAIVYS
GNVLALYMTKPQGFKYKSGMYMFVKCPDISKFEWHPFSITSAPGDEYLSVHIRALGDWTSELRNRFAETCE
PHQKSKPSPNDLIRMETRARGANPHVEESQALFPRIFIKGPYGAPAQSYQKFDILLLIGLGIGATPFISILKDML
NNLKPGIPKTGQKYEGSVGGESLGGSSVYGGSSVNGGGSVNGGGSVSGGGRKFPQRAYFYWVTREQASFE
WFKGVMDDIAVYDKTNVIEMHNYLTSMYEAGDARSALIAMVQKLQHAKNGVDIVSESRIRTHFARPNWR
KVFSELSNKHETSRIGVFYCGSPTLVRPLKSLCQEFSLESSTRFTFHKENF

>amoeba-Dd-NoxA: GenBank^{TM} XM_630972

MRLPTKEEIQRYWVNEGNKLILVILYTLGNIAAFVYTFVHYYNSPAFEVVGYGVCFARGCAQLLKLN
CALILVPVLRNLLSFLRGTFLNNYVPFDKNIVFHKLIAWVICFATFGHVMAHFNNFRLYQDITPQEY
KRILGIDYPNLTPIKYAFATLAGWTGHVVCIVMVLMYTSAVESIRRPMFEGFWYTHHLFVVFFGLLV
VHGLHSILEPTSFWKWVIGPCALYIVERLIRLLRSKKTTMLIQARIHPSRVIEVRMKTERFKYKPGQ
YLFLNCPTIAQNEWHPFTITSAPEEDFVSCHINVVGNWTGKLSTLLNPDKKMGIVQENVLKSPDG
KPILRIDGPFGAASEEVFKYKQVILVGAGIGVTPFASILKHIKYQMARTYNTTPLIDKVHFYWICRDR
NSFEWFSGLIGELEMENHNNFLEIHPYLTGALSAQEIRDVMYGDEEKDLITGFTTPTQFGRPKWD
EIFADHALRYAEKDVGVFFCGPKLLSKSLYKASTHYTKTTTCRFHYNKENF

>amoeba-Dd-NoxB: GenBank^{TM} No. XM_632294

MNEKKELQQELELQEFQTPKNQQLEKLQEPNGEISSTGNETSESGISSPPISQNDNSNNENESLNI
TPNKPFVSMQEELQNLDIENIPIPPTIQTPKIYKNTNNLIHSKNNLSLPISLSQENIVKLDKVDIESN
DQVNSNTDNNNNTNNNNNTNNNKNEKIGLRSKIFKSKIFIKIRGWWWHRGISTYIMLFYIALNIGV
GVHMFYNMYHSDIFKFLGLSFCFSRTAARLINLNSAVILLPVLRNFLSWLRGTIVNNYIPIDKHLNF
HKLCAFMLFCCTIIHCVGHYISFKKINDDVLKIDDGKSVAGDYLNININNFPDEKYLFFKSVPGITG
HIMLLILILIVSSSMWRIRRPMFEIFWYVHHLFIPFYILLCFHGYSKILKKDPQSWMWIIAPFILYSIE
RLIRIARSKKRVILEKAIMHPSKVLELRMKRDNDNFNFKPGQYLYLNCPSIAYHEWHPFTITSAPDD
PFISVHINIVGNWTRKLFKLLNPDNKLGLIQEDLKSTQNRGKRRILKIDGPFGAPAENFFKYRNLVL
IGAGIGVTPFSSILRHLKNQNDKQTNADENHLKINKIYFIWISRQKNSFQWFTDILAELENDERIDSI

LEIHIFLTGALELDDYAKIKNAQKCHITNLHSKTLFGRPNFRSIFNQLTQLHQREKIGVFYCGNKAL
GKNIIKNCNKFNGKNNCHLIFHKENF

>amoeba-Dd-NoxC: GenBank^{TM} XM_630295

MEKQKNISIKKFANPLSFPHEFRTNLHKSTFLDASLLELPNISVDKTKNENNFSITDKILMESLKENEFKIREFI
KNQESGNRVLMIDKNSLKELFNINDNQALDLIFNQYVQSTRPNLIRKSSNSSLNIDLKKEIKKKTKKSRKSFSR
NSKLNNNDSKIDDKNDNYIDKINDINNFNSDIEEIFKKKKEIENTLKIPIGGNSFPIPVEFNNNNNNNNNNNNN
NNNNYNNNIDNNNNNNNNNNNNNINNCNNNNINNDNNNNNNNNDNDNNNINTVDNHDDDIINNSNNFNKN
EYPSSNISPISPKSSISSFPTNLNNSINNTGSMVSDSLSSCRNSISSSSIDSSVASIPITIQSIDFEDKNIKSDQFKIISK
SNIENTIETNPIPPFNQTNNQCEVQLQSHSLPTILKQPHIYKSKSFSSSINSNSKIKKIKKSRSFEIESKINLFDVIN
HIYLNLSKVGSEEQKITSVFKLYDIYDKGFISRDDLKEVLNYRTKQNGLKFQDFTMESLIDHIFQQFDKNMDG
YIDFEEFKSELTINNENKVKEKEENTNYNFKEENIGIYTEKESFHSLKRYLKIEGSKLFFISLFFIINSILVITSFLN
VHANNKRAIELFGPGVYITRIAAQLIEFNAAIILMTMCKQLFTMIRNTKFKFLFPVDKYMTFHKLIGYTLIIASF
LHTIGWIVGMAVATGKPDNIFYDCLAPHFKFRPTVWEMIFNSLPGVTGFIMISFLIIMAILSLKIIRKSNFELFY
YSHHLFIGFYVLLILHGTMGWIRPPTFWKWFIVPGFFYTVDRSFRLFKRTHRVEVLDYCLKNERVINLTFSKP
PSFDYKPGQYLLINVPHISKLQWHPFTMTSSPLEDKIYVHIRVTGNWTKKLFRWLSIKKQLQQQQLYNNIK
QQNVLPDGSNFIINNNNNIDQIDLEIGLKPFRINIDGPFGSSSQYALKQKQVILVGAGIGVSPMASLLKDISLKK
QRLQLLNQGDQIALEQSKNEITTKFGLGNLEKVHFFWLNRDQHSFQWFEDLLIDISTNGNSNLPKISINTFNT
RVFPKNDVRVFMLWNGLDKLFKAQGLDPTTNLPFKTHWGRPNWDTIFQYYSKKYSGESISVFCCGPSQLSK
ELYEKCRYYTCLKTGGTKFYFHKENF
