# Supplementary material for: Molecular evolution of the reactive oxygen-generating NADPH oxidase (Nox/Duox) family of enzymes
Source: BMC Evol Biol. 2007 Jul 6;7:109. doi: 10.1186/1471-2148-7-109 (PMC1940245; doi:10.1186/1471-2148-7-109)
Supplement: Additional File 1 — Supplemental Figure S1. The figure provided shows loop and segment sizes joining transmembrane regions and canonical NADPH-oxidase domains [file 1471-2148-7-109-S1.pdf]

**Additional file 1**

**Supplemental Figure S1. Loop and segment sizes joining transmembrane (TM) regions and canonical NADPH-oxidase domains**

The numbers of amino acid linking predicted TM regions and other canonical domains are indicated. Abbreviations indicate the six TM domains (TM1 to TM6), two FAD-binding subregions (FAD1/2), and the four NADPH-binding subregions (NADPH1 to NADPH4). Amino acid positions of each region in human Nox2 sequence (GenBank$^{TM}$ No. NM_000388) are: TM1, residues 10-34; TM2, residues 50-72; TM3, residues 99-123; TM4 residues 166-190; TM5, residues 204-228; TM6, residues 270-289; FAD1/2, residues 336-361; NADPH1, residues 375-389; NADPH2, residues 440-447; NADPH3, residues 504-513; NADPH4, residues 535-540. The numbers of amino acid residues between these regions were counted using alignments shown in Additional file 3, and are indicated in the diagram according to the subfamily classification in Figure 1B. The numbers of residues between FAD1/2 and NADPH1 of Nox5 proteins show extensive variability: 68-71 amino acid (aa) residues (*H. sapiens*, *B. taurus*, *C. familliaris*, and *X. tropicalis*), 210 aa (*A. mellifera*), 267 aa (*T. rubripes*), 281 aa (*G. gallus*), 307 aa (*A. gambiae*), and 354 aa (*D. melanogaster*). Exceptions to the indicated numbers are shown by *asterisks*: *1 = 48 aa (*C. intestinalis* Nox2), *2 = 21 aa (*X. tropicalis* Nox4), *3 = 57 aa (*T. rubripes* Nox4), 135 aa (*C. intestinalis* Nox4), *4 = 54 aa (*M. grisea* NoxA), *5 = 205 aa (*C. intestinalis* Nox4), *6 =

68 aa (*T. rubripes* Duox), *7 = 11 aa (At-rboh-H), *8 = 22 aa (*C. familliaris* Duox), 24 aa (*G. gallus* Duox1), 21 aa (*C. intestinalis* DuoxA), and *9 = deletion (At-rboh-H).

| | | TM1 | loop-A | TM2 | loop-B | TM3 | loop-C | TM4 | loop-D | TM5 | loop-E | TM6 | | FAD1/2 | | NADPH 1 | | NADPH 2 | | NADPH 3 | | NADPH 4 | |
|---|---|---|---|---|---|---|---|---|---|---|---|---|---|---|---|---|---|---|---|---|---|---|---|
| Nox1 | | | 16 | | 27 | | 37-41 | | 13 | | 42-44 | | 46-49 | | 38-52 | | 19 | | 57 | | 21 | | 30 |
| Nox2 | | | 16 | | 27 | | 38-41[*1] | | 13 | | 42-44 | | 46 | | 44 | | 19 | | 57 | | 21 | | 27-30 |
| Nox3 | | | 16 | | 27 | | 38 | | 13 | | 41 | | 46 | | 44 | | 19 | | 57 | | 21 | | 30 |
| Nox4 | | | 16 | | 27 | | 24[*2] | | 13 | | 71-75[*3] | | 45-46 | | 49[*5] | | 15-16 | | 52 | | 21 | | 26-27 |
| Nox5 | | | 6 | | 23 | | 24-29 | | 15 | | 3 | | 48-49 | | 68-354 | | 33-35 | | 65-66 | | 21 | | 22 |
| Duox | | | 16 | | 23 | | 25-31 | | 14 | | 5 | | 47-49 | | 37-41[*6] | | 18-20 | | 66 | | 22 | | 28-29[*8] |
| At-rboh | | | 12 | | 24 | | 24-26 | | 25-27 | | 11-12 | | 48 | | 53-69 | | 30-56 | | 58 | | 21[*7] | | 28[*9] |
| NoxA/NoxB | (fungus) | | 15 | | 21 | | 13-14 | | 13 | | 20-21 | | 46[*4] | | 60-65 | | 18 | | 53-63 | | 21-33 | | 27 |
| NoxA/NoxB | (amoeba) | | 12 | | 21 | | 28-31 | | 13 | | 6-7 | | 46-48 | | 50-51 | | 20-23 | | 47-50 | | 21 | | 28 |
| NoxC | (fungus) | | 13 | | 24 | | 25 | | 13 | | 7 | | 48 | | 38-41 | | 87-103 | | 66-68 | | 43-47 | | 33-37 |
| NoxC | (amoeba) | | 14 | | 23 | | 25 | | 13 | | 6 | | 47 | | 77 | | 40 | | 61 | | 21 | | 30 |
| NoxD | (alga) | | 16 | | 24 | | 9-11 | | 13 | | 5 | | 47-48 | | 38-41 | | 314-425 | | 38-44 | | 20 | | 36-63 |
