# Supplementary material for: Molecular evolution of the reactive oxygen-generating NADPH oxidase (Nox/Duox) family of enzymes
Source: BMC Evol Biol. 2007 Jul 6;7:109. doi: 10.1186/1471-2148-7-109 (PMC1940245; doi:10.1186/1471-2148-7-109)
Supplement: Additional File 6 — Amino acid sequences of vertebrate Nox regulatory subunits; p47 phox, NOXO1, p67 phox, NOXA1, p22 phox. Lists of amino acid sequences Nox regulatory subunits of H. sapiens, C. familliaris, R. norvegicus, M. musculus, G. gallus, X. tropicalis, D. rerio, T. rubripes, T. nigroviridis are provided. [file 1471-2148-7-109-S6.pdf]

Additional file 6

Amino acid sequences of vertebrate Nox regulatory subunits; p47*phox*, NOXO1, p67*phox*, NOXA1, p22*phox*

To describe species of the genes, we use the same abbreviations as in Additional file 4.

Sequences are available from the indicated databases:

http://www.ncbi.nlm.nih.gov/(GenBank™ numbering genes),

http://www.ddbj.nig.ac.jp/searches-e.html (DDBJ™ numbering gene) or

http://www.ensembl.org (ensembl numbering genes).

>human-Hs-p47*phox*: GenBank™ No. NM_000256
MGDTFIRHIALLGFEKRFVPSQHYVYMFLVKWQDLSEKVVYRRFTEIYEFHKTLKEMFPIEAGAIN
PENRIIPHLPAPKWFDGQRAAENRQGTLTEYCGTLMSLPTKISRCPHLLDFFKVRPDDLKLPTDNQ
TKKPETYLMPKDGKSTATDITGPIILQTYRAIANYEKTSGSEMALSTGDVVEVVEKSESGWWFCQM
KAKRGWIPASFLEPLDSPDETEDPEPNYAGEPYVAIKAYTAVEGDEVSLLEGEAVEVIHKLLDGWW
VIRKDDVTGYFPSMYLQKSGQDVSQAQRQIKRGAPPRRSSIRNVHSIHQRSRKRLSQDAYRRNSVR
FLQQRRRQARPGPQSPGSPLEEERQTQRSKPQPAVPPRPSADLILNRCSESTKRKLASAV
>dog-Cf-p47*phox*: DDBJ™ No. BR000287
MGDTFIRHIALLGFEKRFVPSQHYVYMFLVKWHDLSEKVVYRRFTEIYEFHKMLKEMFPIEAGDIN
PENRIIPHLPAPRWFDGQRAAESRQGTLTEYYNTLMGLPVKISRCPQLLDFFRVRPDDLKLPTDSQV
KKPETYLVPKDGKSSVTDITGPIILQTYRAIADFEKTSSSQMALATGDVVDVVEKSESGWWFCQTKT
KRGWVPASYLEPLDSPDEAEDPEPNYEGEPYVTIKAYTAEMEDEMSLQEGEAIEVIHKLLDGWWV
VRKDDITGYFPSMYLQKSGQDAAQAHRQIKSRGAPPRRSSIRNAHSIHQRSRKRLSQDTYRRNSVP
TQPGKPQPAVPPRPSADLILHRCSESTKRKLASSV
>mouse-Mm-p47*phox*: GenBank™ No. NM_010876
MGDTFIRHIALLGFEKRFIPSQHYVYMFLVKWQDLSEKVVYRKFTEIYEFHKMLKEMFPIEAGEIH
TENRVIPHLPAPRWFDGQRAAESRQGTLTEYFNGLMGLPVKISRCPHLLDFFKVRPDDLKLPTDSQ
AKKPETYLVPKDGKNNVADITGPIILQTYRAIADYEKSSGTEMTVATGDVVDVVEKSESGWWFCQM

KTKRGWVPASYLEPLDSPDEAEDPDPNYAGEPYVTIKAYAAVEEDEMSLSEGEAIEVIHKLLDGWW
VVRKGDITGYFPSMYLQKAGEEITQAQRQIRGRGAPPRRSTIRNAQSIHQRSRKRLSQDTYRRNSVR
FLQQRRRPGRPGPQSTDGTKDNPSTPRVKPQPAVPPRPSSDLILHRCTESTKRKLTSAV
>zebrafish-Dr-p47*phox*: Ensembl No. ENSDARG00000033735
MAETYVRHVELLGFEKRFFPSQHYVYMLLVKWSDQSEKLVYRRYPEVHTLHKTLKEMFPIEAGDI
DEKDRIIPTLPAPKWLDNQKTTETRQATLAEYCRSLLNLPANISRCQLIRDFFKMRPEDETPPAPHP
YKRNETFIMSTNRVRSNTTSEITGPIILETYRVIADYSKSSKYELTLKMGDMVDIVEKSPNGWWFCQ
CESRRGWVPASYLEPLDGADESEEPEPNYAGELYKTTRGYKAVEQDEMTLEAGVIIEVIHKLLDGW
WVVRKGEETGFYPSMFLCRTGEKKEVDAERDVVRRATPPPRRSTIRNAQSIHSTVRRRISQDSYRK
QSRRFLQQRGRLNSHSRIGTRSPLQERRTNKNIEKSSAPQAEDEDKSVPVVPPRPSPQLILERCTEN
TSKRMSMQEA
>chicken-Gg-p47*phox*: GenBank™ No. NM_001030709
VGDTFIRHIELLRYEKRFFPSQHYVYMFLVKWNDLSEKLIYRRFTDIYEFHKALKEMFPIESGDINA
ENRIIPHLPAPKWFDGQRSTQSRQGTLAEYCYTLVNLPHKISRCRHVVSFFEVRPDDMNPVTDSQI
RKPEVFLLPKDAKKNTSDITGPIVLQTYRAIADYEKSSKSEMAVKAGDAVDVVEKSETGWWFCQLK
TKRGWVPAAYLEPMDGPDESEEQEPNYAGELYVVQKSYTAVEEDELTLKEGDTIEVIHKLLDGWW
VIRKDETTGYYPSMYLQKSGEVNSPEKSGLRNHNIPPRRSTIRNAKSIHNKGRKQISQETYRRNSK
KYMQNRRNMRGNLQNKDIISEKNEQEENKSKAQPAVPPRPSKDLIMNRCTESTRRKI
>rat-Rn-p47*phox*: GenBank™ No. NM_053734
MGDTFIRHIALLGFEKRFVPSQHYVYMFLVKWQDLSEKVVYRKFTEIYEFHKMLKEMFPIEAGEIH
TENRVIPHLPAPRWYDGQRAAESRQGTLTEYFNSLMGLPMKISRCPHLLNFFKVRPDDLKLPNDS
QVKKPETYLTAKDGKNNVADITGPIILQTYRAIADYEKGSKTEMTVATGDVVDVVEKSESGWWFCQ
MKTKRGWVPASYLEPLDSPDEAEDPDPNYAGEPYVTIKAYAAVEEDEVSLSEGEAIEVIHKLLDGW
WVVRKGDITGYFPSMYLQKAGEEITQAQRQIRSRGAPPRRSTIRNAQSIHQRSRKRLSQDTYRRNSV
RFLQQRRRPARPGPQSPDSKDNPSTPRAKPQPAVPPRPSSDLILHRCTESTKRKLTSAV
>frog-Xr-p47*phox*: Ensembl No. ENSXETG00000018448
MTEPHIRHIQLLGFEKRFIPSQHYVYMFMVKWQDLTEKLVYRKFTEIYEFHKSLKEMFPIEAGDIS
KEHRTIPHLPAPKWFDGLRSTENRQVTLSDYFSSLLSLPPKISRCPHVLNFFQVRSDDVNPVANNT
NGRKPETFLLKVDTAKKNVSDITGPIILQSYRVIADYEKNSKSELAAKNGDVVEIVEKSENGWWFC
QLRNKRGWMPAAYLEPLDGPDESEEQDPNYEGDLHITTKDYSGELDDELSLQEGENVEVIHKLLD
GWWVVRKGSITGYFPAMYLQKSGETAPANENPSKRKGLPPRRSTISNANSIHKKERKQISQDTYRR
NSKKYLKQRQSIVDTKSPIITEENKEEESKSKPQPAIPPRPSKELILDRCSENTKSKI
>tetraodon-Tn-p47*phox*: DDBJ™ No. BR000288
MADTYVRHVQLLGFEKRFFPSQHYVYMLLVKWSDLTEKLIYRTYPEIYTFHKALKEMFPIEAGKIE
KRDRIIPSLSAPPWLDSQKSTETRQTSLSDYCQALVNLPPHISRCTHLTSLFKVRPEDENPAAPNTL
KRNETFVVSRDLARGNASEISGPIILDMYRAIADYTKTTKYEINLLAGDQVEIVEKNQNGWWFCQM

DSKRGWVPASYLEPLDGPEESEEADPDYEGSELFITIKAYKAEQEDEISLDLGESIEVIHKLLDGWW
VVRKGEGTGYFPSMFLQKASKRAQAEAARNHLQGQKPPPRRSTIRNAKSIHNKSRQRLSQDAYRR
NSRRYLQQKGGQRDLQNKYARTAAKSPLQERKNQGNIPEESGTASEGEPKKEAPVVPPRPSPELIL
QRCSDNTRKKIS

>fugu-Tr-p47*phox*: GenBank™ No. AB099897

MAETYVRHVQLLGFEKRFFPSQHYVYMLLVKWSDLTEKLIYRTYPEIYTFHKSLKEMFPIEAGKIE
KRDRIIPSLSAPPWLDSQKSTETRQTTLSDYCHSLVNLPPHISRCTHLTGFFTVRPEDENPPSPNILK
RNETFVVSKDLARGNVSEISGPIILDMYRAIADYTKTTKYEINLHAGDQVEIVEKNQNGWWFCQCD
SKRGWVPASYLEPLDGPEESEEAEPDYGGSPCELYITIKAYKAEQEDEITLDLGESIEVIHKLLDGW
WVVRKGEQMGYFPSMFLQKANKREQSESSRANVQGHKPPPRRSTIRNAKSIHNKSRQRLSQDTYR
RNSRRYLQQKGGQLVKPDPYPRNVAKSPLRERRNQGNIPEESSTISENEGKREAPVIPPRPSPELIL
QRCSDNTRKKISIHKSSSASTSKPGQA

>human-Hs-NOXO1: GenBank™ No. NM_172167

MAGPRYPVSVQGAALVQIKRLQTFAFSVRWSDGSDTFVRRSWDEFRQLKKTLKETFPVEAGLLRRS
DRVLPKLLDAPLLGRVGRTSRGLARLQLLETYSRRLLATAERVARSPTITGFFAPQPLDLEPALPPGS
RVILPTPEEQPLSRAAGRLSIHSLEAQSLRCLQPFCTQDTRDRPFQAQAQESLDVLLRHPSGWWLV
ENEDRQTAWFPAPYLEEAAPGQGREGGPSLGSSGPQFCASRAYESSRADELSVPAGARVRVLETSD
RGWWLCRYGDRAGLLPAVLLRPEGLGALLSGTGFRGGDDPAGEARGFPEPSQATAPPPTVPTRPSP
GAIQSRCCTVTRRALERRPRRQGRPRGCVDSVPHPTTEQ

>dog-Cf-NOXO1: GenBank™ No. XM_547183

MAGSRHPVSVRAAALVQTGRLQTFAFSVCWSDGSDTFVRRSWAEFKELHKTLKEAFPVEAGLLRR
SDRILPKLPDTSLLVRGGRTGRGLARLRLLDTYTRALLAAAEQLSRSAVLTGFFEPQPVDLEPVLPP
GSLVILPTPEEPHRRPPHSPAICSLEAQSLRCLQPFSTQDTQGWPFHARAQEVIDVLLRHPSGWWLV
ANEEQQMAWFPAPYLEEAAPDREGTTLRSSGSQFCASQAYESSHADELSVPAGARVSVLETSDRG
WWLCRFRGRSGLLPAVLLQPEGLGALLSGPGLHREANSKEDRGGEAQRTPEACQATTLSPSVPARP
PLSAIRSRCCSVTRRALASKYPPRAGQ

>chicken-Gg-NOXO1: Ensembl No. ENSGALP00000008924

YMMFVSWSDQNNILIYRTLEEFKRFHKELKRKFPIESGSLRRSDRTIPRFKDINGKQKKSGKINRSL
ERLKLLETYTQELLKVDAKISQGEDVIQFFKAQTQDLDPCFPEDSVVIMPSEIGGEKKKEVQQQQL
SITYPQVSQSYRCIETFETKDTKNKTFKVAKKEIVEVLLKDMTGWWLVENADKQIAWFPASYLEQIS
AHKDIQNVESSDEEGSLYFVMRAYEAQKADELSLNKGVVVEVVRRSDNGWWLIRYNGRKGYMPS
MCLQAYKNPHHRLQTIMNSGLHISTPNLCSPSPALQPLRDSTARDCTSGDGSDEDLESDSSSLSSGS
APSGVLSWKPDLSRSLPEVEQAVPMRPSAHEILQRCSTVTKRAVQQSA

>mouse-Mm-NOXO1: GenBank™ No. NM_027988

MASPRHPVSAHAVALVQMDRLQTFAFSVCWSDNSDTFVRRSWDEFRQLQKTLKKTFPVEAGLLRR
SEQVLPKLPDAPLLTRRGHTGRGLVRLRLLDTYVQALLATSEHILRSSALHGFFVPKPLDLEPMLPP

GSLVILPTPEEPLSQPRGSLDIHSLEAQSIPCVQPFHTLDIRDRPFHTKAQEILDILLRHPSGWWLVE
NKDQQVAWFPAPYLEEVATCQGQESGLALQGSGRQFCTTQAYEGSRSDELSVPSGARVHVLETSDR
GWWLCRYNGRTGLLPAMSLQPEGLGSLLGRPGFPDSAGADKVAEDRTIPPVVPTRPCMSAIQSRCC
SITRRALGQEQGTRVPR

>rat-Rn-NOXO1: GenBank™ No. XM_220221

MASPRHPVSAHAVALVQMERLQTFAFSVCWSDNSDTFARRSWEEFRQLQKTLKKIFPVEAGLLQRS
ERVLPKLPGQACRNAPLLTRRGHTGRGLLRLRLLETYVRSLLATSQHIVTSSTLNSFFAPKPLDLEP
MLPPGSLVILPTPEEPLSQPIGSLAIHSLEAQSMRCLQPFHTLDTKDRPFHTQAKEILDILLRHPSG
WWLVENKDQQTAWFPAPYLEEIATGQGQESGMAVQGSGRQFCATQAYEGSRPDELSVPSGARVHV
LETSDRGWWLCRYNGQTGLLPAVLLQPEGLGSLLGRPGLPDSGGADKVTEGRTVPPVVPTRPCMS
AIQSRCCSITRRAVGQEQRTQVPP

>frog-Xt-NOXO1: Ensembl No ENSXETT00000015702

WSDHNEILIYRTFEDFKKLNRQLKKKFPLEAGLFRKSDNLLPKLKDVPIFRKNRTTNRFIERLRLL
EKYSQELLRTDGKISQCDLVLKFFTPSNNDLNPKFPENSLVMMTSDSKDQKEQKKPLPEAPAIHPI
VSQQYICMEDYETKDTKNRPFKVKRHELVGVLIKENTGWWLVENEEKHLAWFPAPYLKDVDNSE
DTDSGTSEDEGVLYYAAKAYEAMNSDEVSITVGVLVEVIEKSNNGWWLIR

>zebrafish-Dr-NOXO1: DDBJ™ No. BR000290

LIKLFIRRVEHRNMDINIHSSPPQLYMTTVLWSDGNEITVYRSLEDFKKMHRQLKKKFPPSNPFKR
SARIVPEFKGNKWSGSKSVLRMKALEEYCGQLLKSDAQVCRSSELIQFLLPKAHDLNADFAKNCIV
IMPSDVTLGSSKAESNSGVTQPFVTETYRCIANYETKDTKNRPFKVEVDETVDVLIKDQKGWWLVE
NESKHLAWFPAPYLERAEMADDGPDEMDNESFQSAGVFYVATKAYKATNSDELSVELGSVLEVLQ
KSDNGWWIVRYNRKAGYVPSMYLQPHNNPRILLKSTQKEISRSTLDLAQLQHPQTLQDSRLRELS
RSQGNLLLQPAETDIMDKQKSDPCLNKMPSTPKVPPRPAVQEILTRCTTVTRKNMQ

>tetraodon-Tn-NOXO1: DDBJ™ No. BR000291

METQRYPISARLVGVLHKEKSKVGAHKSQVVLFHARFNQQLCPLQMYMTSVLWSDHNEIVVYRTF
QDFRKMHKLKRSGKKKSPTRSLVRLKFLQKYCNELLSCEPRVSQSADLIQFFHPNAQDLEPEFSK
NRQEEVKAEAGHGSVGNVTQPFVTVTYRCVSQYETKDTKNKPFKVAADEKVDVLIKDKAGWWLV
ENEEKRMAWFPAPYLEKLEEDGDEDDTDGTRTLYLTAKNYKASKGDEISVAVGAVVEVLQKSDSG
WWLIRYQGKVGYVPTLCLQPYNRPQVRLNGAAFPGLQQQSNKLSSSRGNLLQLPSAGRSPSPQQP
HADGRQRSHSLNALLETLPAQPARGAAPDTGTPPSPQQAPPPVIRPQYPRGQNDAQRLRSQHLQGS
RNPQGSTQTPGPGDPHQVHHHHPQECQQRRSVAHPTRDTESVKLPLARFSCSFRLHA

>human-Hs-p67*phox*: GenBank™ No. NM_000433

MSLVEAISLWNEGVLAADKKDWKGALDAFSAVQDPHSRICFNIGCMYTILKNMTEAEKAFTRSINR
DKHLAVAYFQRGMLYYQTEKYDLAIKDLKEALIQLRGNQLIDYKILGLQFKLFACEVLYNIAFMYAK
KEEWKKAEEQLALATSMKSEPRHSKIDKAMECVWKQKLYEPVVIPVGKLFRPNERQVAQLAKKD
YLGKATVVASVVDQDSFSGFAPLQPQAAEPPPRPKTPEIFRALEGEAHRVLFGFVPETKEELQVMP

GNIVFVLKKGNDNWATVMFNGQKGLVPCNYLEPVELRIHPQQQPQEESSPQSDIPAPPSSKAPGRP
QLSPGQKQKEEPKEVKLSVPMPYTLKVHYKYTVVMKTQPGLPYSQVRDMVSKKLELRLEHTKLS
YRPRDSNELVPLSEDSMKDAWGQVKNYCLTLWCENTVGDQGFPDEPKESEKADANNQTTEPQLK
KGSQVEALFSYEATQPEDLEFQEGDIILVLSKVNEEWLEGECKGKVGIFPKVFVEDCATTDLESTR
REV

>mouse-Mm-p67*phox*: GenBank™ No. NM_010877

MSLAEAIRLWNEGVLAADKKDWKGALEAFSEVQDPHSRICFNIGCVNTILENLQAAEQAFTKSINR
DKHSAVAYFQRGMLYYRMEKYDLAIKDLKEALTQLRGNQLIDYKILGLQFKLFACEVLYNIALMHA
KKEEWKKAEEQLALATNMKSEPRHSKIDKAMESIWKQKLFEPVVIPVGRLFRPNERQVAQLAKKD
YLGKATVVASVVHQDNFSGFAPLQPQSAEPPPRPKTPEIFRALEGEAHRVLFGFVPETPEELQVMP
GNIVFVLKKGSDNWATVMFNGQKGLVPCNYLEPVELRIHPQSQPQEDTSPESDIPPPPNSSPPGRL
QLSPGHKQKEPKELKLSVPMPYMLKVHYKYTVVMETRLGLPYSQLRNMVSKKLALSPEHTKLSY
RRRDSHELLLLLSEESMKDAWGQVKNYCLTLWCEHTVGDQGLIDEPIQRENSDASKQTTEPQPKEG
TQVVAIFSYEAAQPEDLEFVEGDVILVLSHVNEEWLEGECKGKVGIFPKAFVEGCAAKNLEGIPRE
V

>dog-Cf-p67*phox*: DDBJ™ No. BR000293

MSLAEAISLWNEGVLAADKKDWKGALEAFAAVQDPHSRICFNVGCMHTILGNMPEAEKAFTRSIN
KDKHLAVAYFQRGMLYYHMEKYDAAIKDLKEALTQLRGNQLIDYKILGLQFKLFACEVLYNIAFMY
AKNEEWKKAEEHLALAMSMKSEPRHTKIDKAMECVWKQKLYEPVVIPMGRLFRPNERQVAQLAK
KDYLGKATVVASVVDQDSFSGFAPLQPQAAEPPPRPKTPEIFRALEGEAHRVLFGFVPETPEELQV
MPGNIVFVLKKGNDNWATVMFNGQKGLVPCNYLEPVELRIHSQQQPQEEASPESDIPAPPSSSAPG
RPQLPPGWSSFCFLSPQDVKLRVPMPYTVKVHYKYTVVMEIQAGLPYSQLRDMVAKKLELLPEHT
KLSYRPPDSHELEPLSEDNMKAAWGQVRNYCLTLWCENTVGDQGFPDEPQESEKSEANNQTTEP
TLKEGGHVVALFTYEATQPEDLEFQQGDIIQIISMVNEDWLEGECKGKIGIFPKAFVEEHATTDLES
SPRGV

>zebrafish-Dr-p67*phox*: DDBJ™ No. BR000296

MSFVSTLRQWDEAVACVEQRDPDAALRIFLSIEEKNSKIAFNIGCLCLNNSDLDEAEKAFDGSIGKD
EHLAVAFFQRGVTFYKKEKFEESLLDFQQAFKQLRGNQLIDYTPLGLRYKLYACEVLHNIGLAQAQ
LGKWEKAQENLLTALSLRADAKFSHIDHALDAILKHKLFPLVEVRAGLLFKPNKKYVAELEKRDYL
GKAKVVASVVPADEFSGFAPLQPQIDNVPSIPKVPEVLRVLEGEPHTVLYEFVPETKEELAVLPGNIV
FVLHRGTDNWASVVFNEKRGLVPYNFLEPLDIVTMTSKPVETEALNENDDIPAPPRRAAPSRPVAP
EGLKTVNAKLYSPQTVNTREFSGCVVKVHFQFTIAIAIAHGQPYGVILQMISSKLKLPASTLTLRYAK
EGSAERVIIEDSEMEAVWNSAKDGRLTLWCSVTEGKSASHAKVVALYSYESSTPEDLEFKQGNVIT
VLSKVNDEWLEGQCNGKIGIFPSSFVEPLNGDPH

>chicken-Gg-p67*phox*: DDBJ™ No. BR000295

MSLVETIRLWQEGVCAADGKEWGAALKAFTAVQNPPAKICFNIGCTHLVLGQLAEAEEAFTQSISC

DKHLAVAYFQRGTVFYKRHNHEMALKDFKEALAQLRGNQLIDYKILGLRYRLFACEILYNIALVYAT
MENWKKAEEHLTLAMSSKSEPQHNKIDRAMEAILKQKLYEPVAIPTGKLFRPNEKQVAQLEKKDY
LGKAMVVASVVDKDSFSGFAPLQPQASGPPPRPKTPEILRALEGQPHRVLYEFIPETAEELQVLPGN
IVFVLKKEKDNWATVMFNGKKGIVPCNFLEPMELQHKLHVQDEAPLEPDIPEPPSSTAPRPRRPAP
GQEKPDTPIHHLQESEPDIPKPYVLKVHYKYTVAMQVKPDLSYKELLGLVCDKLELQPEHTMLRY
KSAASGELVPLSAQNLEEAWSHSKDQCLTVWCDCTEGEGFLPDSKPEEPQQAAAETGPTQVVAQY
SYEATQPEDLEFQAGDVILVLSKVNEDWLEGQCNGKIGIFPSAFVRDGNTKDP

>rat-Rn-p67*phox*: DDBJ™ No. BR000294

MSLAEAIRLWNEGVQAADKKDWKGALEAFSEVQDPHSRICFNIGCMYTILDNLQEAEQAFTKSIN
RDKHLAVAYFQRGMLYYSMEKYLLCVYCLREALVTFRKKQVLSPKPEGLQFLLQRSPVLYNIALMH
AKKEEWKKAEEQLALATNMKSEPRHSKIDKAMESIWVSVVVDGPCCLAQYHVQRASHKQVPVLS
KDLLGEETVVASVVHQDNFSGFAPLQPQSAEPPPRPKTPEIFRALEGEAHRVLFGFVPETPEELQV
MPGNIVFVLKKGSDNWATVMFNGQKGLVPCNYLEPVELRIHPQSQPQEDTSLESDIPPPPNSSPPE
RLQLSPGHKQKEEPKEVKRSVPMPYMLKVHYKYTVVMETQLGLPYSQLRNMVSKKLELLPEHTK
LSYQRRDSPELLLLLSEESMKDAWAQVKNYCLTLWCEHTVGDQGFVDEPKEKENSDADNRTTEPQ
PKEGTQVVAIFSYDATQPEDLEFVEGDVILVLSHVNEEWLEGECKGKIGIFPKAFVEGCAAKNLEG
TPREV

>frog-Xt-p67*phox*: DDBJ™ No. BC099979

MALVEIMRLWSEGVAAAENEDWNGALKSFTSITDPRSKICFNIGCCHLVLGDLEKAEKAFTLTIERD
MHLAVGYFQRGFVFFQRGKYSLALQDWTRAYTEMRGNQLIDYKILGLIFKLYSCEILHNIALTHAK
EGKWAKAEESILLALSQKVELRHNTKLEKAMEDILKEKVFAAVKIPKGRIFQPNERLVEQLEKKDY
LGKALVVASVVDKDSFSGFAPLQPQASNPPPRPKTPEILRTLQGEPHRVLFEFNPETAEEMQVLPG
NIVFVLKKGDDNWATVVFNGKKGIVPCNYLEPVELRFQSAQQTGVQSELDSPTNRPQQSDVPAPP
DATPPQLLKNTKEAVAVASYLVKVYYKYTVAIQISSKLPFADLLTLISSKLQLLPSRMKLSFKEDQDD
VLLNEENTEKAWSLATDNCLKLKCTEVQVRQARSLYCSTMQGYFKAQGKYPIALFEYEATQPEDL
PFCKGDIIKILSHVSEDWWEGECQGRMGIFPKVFTEE

>tetraodon-Tn-p67*phox*: DDBJ™ No. BR000297

MSFLDTLRQWDNACTVADGQDFSAALQVFLSIQEPNSKICFNIGCLHLLNEDLSAAEKAFDSSISK
DEHLAVAFFQRAITFYKMSRQTHRPCHFQQTFKELRGNQLIDYGALGLRYKLTACEVLHNMALAE
AQLGRWEKAQESLVKALDYSSDSKLGAIDKALQATLKQKLFKLTGFPSKVLFKPNKRYVAELEKKD
YLCKAKVVASVVPQDEFSGFAPLMPQVRPHLRAASSPTAESNSARVHRALEGEPHTVLYEFVPETS
DELAVVPGNVVFVLQKGADNWASVVFNERRGLVPYNYLERLEISLAAKLKDVRTCQPGKPRPPSQ
QPPTRPERNLLTLFSGLEFQEAQLVDDSYVVKVRFTFTFAVIVPRGSSYATLAQKVGEKLSVPADAVI
LSLSSEAAEEDVINGSTDMEAVWGRASGRCITLWCRLAEQTSETEPRRETFLLALHTYDSPNPEDL
TFQQGDKILLLSKVNQDWLEGQCHGNTGIFPAAFVEEVSVSE

>fugu-Tr-p67*phox*: GenBank™ No. NM_001032682

MSFLDTLKQWDKASTVADRQEFSEALEIFLSIEEPNSKIYFNIGCLHLLNEDLKDAEKAFDSSICKD
EHLAVAFFQRAITFYKMTRQEYSLADFQQTLKELRGNQLIDYGALGLRYKLNACEVLHNIALTEAQ
MGHWEKAQESLVKALDYRTESKLGIIDNALQATLKQKLFKLIGFPSKVLFKPNKHYVAELEKKDYL
GKAKVVASVVPQDEFSGFAPLMPQVESGQTFSKPEPELLRALEGEPHTVLYKFVPETSDELAVVPG
NVVFVLQKGADNWASVVFNERRGLVPYNYLERLEITMASKQNNVQSRPPSRQPPTRPERKSGLPP
CADDRRNTQMKESELADDSCVVKVRYTFTFAVLVPRGSSYATLAEKISEKLSVPANAIVLSLSSEATE
QNVIDGGTDMEGVWSRVSGRCITLWCRLAQTNERVQKESSLLALHSYDSSNPEDLSFHQGDRITLL
SKVNQDWLEGEFNGNTGIFPAAFVEEVPANG

>human-Hs-NOXA1: GenBank™ No. NM_006647

MASLGDLVRAWHLGAQAVDRGDWARALHLFSGVPAPPARLCFNAGCVHLLAGDPEAALRAFDQAV
TKDTCMAVGFFQRGVANFQLARFQEALSDFWLALEQLRGHAAIDYTQLGLRFKLQAWEVLHNVAS
AQCQLGLWTEAASSLREAMSKWPEGSLNGLDSALDQVQRRGSLPPRQVPRGEVFRPHRWHLKHL
EPVDFLGKAKVVASAIPDDQGWGVRPQQPQGPGANHDARSLIMDSPRAGTHQGPLDAETEVGADR
CTSTAYQEQRPQVEQVGKQAPLSPGLPAMGGPGPGPCEDPAGAGGAGAGGSEPLVTVTVQCAFTVA
LRARRGADLSSLRALLGQALPHQAQLGQLSYLAPGEDGHWVPIPEEESLQRAWQDAAACPRGLQL
QCRGAGGRPVLYQVVAQHSYSAQGPEDLGFRQGDTVDVLCEEPDVPLAVDQAWLEGHCDGRIGIF
PKCFVVPAGPRMSGAPGRLPRSQQGDQP

>dog-Cf-NOXA1: DDBJ™ BR000298

MPSLGDLVHDWHRGVQAVARGDWGCALRLFSGDPDPPAKMCFNLGCVHLLAGDPEAALRAFDQA
VTKDTCMAVGFFQRGVANFQLERFQEALSDFRLALAQLRGNAAIDYTQLGLRFKLKTWEVLFNVG
AAQCALGLWAEAAGSLEEALCKGPEGAGEDLHAALAQVQKQATLQLRQVPRGEVFRPHRRHVEH
LEPVDFLGKAKVVSSAIPDDHLQGSRPQQRQVSGAPSSQPPNGPGDPSCGASLACSPHPSAPRPSQ
LGPQRAALGWTEPGGCLSASSQVVATGGPESLVTVTVQCAFTLALKVPWGAGLPHLRTLLSQALPL
QAQHGQLSYRDPSHEARWVALPGEEALQGAWRDTAASPRGLQLQCRAAGSRPVLYQAVAQHNYCA
QGPEDLDLRQGDMVDVLCAGQGPDVPLHVVDPAWLEGHCDGRIGIFPKCFVVPAG

>mouse-Mm-NOXA1: GenBank™ No. NM_172204

MSSLGDQIRDWHRGVLAVAREDWDSALCFFSDVREPLARMYFNRGCVHLMAGDPEAALRAFDQA
VTKDTCMAVGFLQRGVANFQLQRFQEAVSDFQLALAQLRDNAVIDYTQLGLNFKLQAWEVLYNMA
SAQCQAGLWTKAANTLVEAISKWPEGAQDILDIAMDKVQKQVPLQLQQVPKGEVFQPPRRYLKHL
EPMDFLGKAKVVASVIPDDHNAQPQQRSQAEHAGHQPSSSMCKRVLSTTGGHTSPGLYDSLLASR
RPGPGPSEVSSGSEGAATKDPESLVTVTVQCHFTVPLKVPRGTGLSSFQTLLAQALLHQTQTGQLS
YKAPGEERSWIPISTEESLQSIWRNVPVGPGGLQLQCQGVWGRPVLYQVVAQYNYRAQRPEDLDF
HQGDTVDVLCEVDEAWLEGHRDGCVGIFPKCFVVPAGAYVEAMLVLGPQPGDQN

>rat-Rn-NOXA1: DDBJ™ No. BR000299

MSSLGDQIRDWHRGVLAVAREDWDSALCFFSDVREPLAKMYFNMGCVHLMAGDPEAALRAFDQA
VTKDTCMAVGFLQRGVANFQLQRLQEAVSDFQLALAQLRGNAAIDYTQLGLDFKLQAWEVLYNMA

SVQCQAGLWTKAANTLVEAISKRPEGAQDTLEAAMDKVQKQVPLQLRQVPKGEVFQPPRRYLKHL
EPMDFLGKAKVVASVIPDDHNSDIQPQQSSQVEQAGLQSSSPVCKRVLSTRGGHMSPGLWDSLLAT
GGPVPGPSEDSSSAEGTATKDPESLVTVTVQCHFTVPLKVPRGTDLSSFRTLLSQALLQQTQKGQFS
YKARGEDRAWVPISTEDSLQSVWRNVPVSPRGLQLQCRGAWGRPVLYQVVAQYDYRAQRPEDLDF
RQGDTVDVLCEVDEAWLEGHRDGRVGIFPKCFVVPAATCVEALPVPEPQPGEQH

>chichen-Gg-NOXA1; Ensembl No. ENSGAL00000008952

MAYRELLRRWHQAALAADGGDWDAALETLCGIEEPPARICFNVGCMHLRAGRLRDALRAFDETV
MKDNSLAVGYFQRGFVCLQLEMYEEALSDYHMAFSHLRKNPFIDYKQLGLRHILYAWEVLYSTAAT
QCRLQQWQEARDTLEKAVVWRPEGRSATLALALERVQNHQFLEPMQVPPGEFFRPRKKEVEQLD
SKDFLGKPKVISSIIPNDEYIGFEPLRPQEMGAERGCRICPVFLSRDRESGYYRVLSHYYPEGTEKLA
VKASSLVFVLARGANGWATAIHDGQKLHIPTSLLEPASKMDKWPSDSTEKIGDGIPLPPAQVPPSRL
HMLPCHYGRTESPLTHREASSSTDRPAVLRVRCECTVVVRAGEVPSVPALRALLRERFGQQAERGR
LSYRHLDGKELGAVSGEEDLEKMWQQLTDGRITLCCQDSDSHSGRPILYRMLAQHSYSAQGPGDL
EFSKGDVLDILSEVNEDWLEGHCNGKTGIFPKCFATQTSCAAFP

>frog-Xt-NOXA1: GenBank™ No. BC075351

MHYKEVVRRWHEGVVAAEGKDYDAALRSFTAIEDPPSRIWFNVGGIYLLRGDLPRALEAYDKSLA
QDPCLAVGYYQRGYLQFKLGRYEKALSDCHLALSNLRNNSFIDYKQLGLRHVLFSWEAQYNMAAV
LCSLGRWESAEEKLKETLQGDGRNAKLDWALDQVQRRSLLQPMSVPEGEFFRPRKQEVEQLNSV
DFLGKPTVISSVVPNDQYSGFEPLRPQQPGFYEPCRDAMQCREAGYHRVVVHYYPENSNEVAVKA
NSVLFVLNKDGDWATAIHDGQKILIPTSFLEPTNPPKADIKKMNNGIPLPPMKTPPTRPNVRPGME
PLTGVQAGAPVPPQPAGGAAEPYKIKALPVGMEPIVEVAVPVQRSVPTHKETGNVPLGNDASLVED
AGRTIIKPKGESAPEPGPFPTRQTGPDNGPMATPVPTDDDKLVLSVHAEFTVNMTVSKAITYPELQ
GALREELRKHGEQMANHLSYRDPESRGLTPVTGSKDWQEVLKLSRANQVTLCCKETTLCAGRPVL
YRMRAQYDYLAQGPADLSFQQGDLISILSEVNGEWLEGHCHRGIGIFPKCFAQRAEGI

>zebrafish-Dr-NOXA1: GenBank™ No. XM_679087

MLYIELIRLWDEAVKAIDIRDWQGALSKLNQITDHNCRTMFVVASTHIALGQVDLAIKALDRVIAKD
SCLAVGFFQRSAVHMMANRLEEALSDCIWAQKYMRENPVIDYKQLGLRYKLYSWQVLYNAAAVHS
RLQQWDKARDILLAASQERGAGRSNLIDTALEAISRKDVLEPLLLPEGEVFRPRKLEVDQLKPRDF
LGEAKVITSMIPNDDFRGFDPLRPQKPGYYEPKVEDGQDSRYMIMKSAYVAKGAGELTVPAGAEVF
VYSDDDRDGLAVIIYDGKELANGIPHPPALKPPNRPQLPSQRSLELHPSSAGASHTTPPTTSSSTRIP
PQQIQTSPQESGSVVVKVHYTYTMALRVPAETPFRDLQEKIAQKLGQPAMNIRLRHRRPGTRVLTP
LNGDDGLDCLEGVAESGRAQIWCQNEDPLANRTILYQMVALYDYNAQGPEDLEFSEGDTIDILSEV
NEEWLEGHVAGNIGIFPQSFAHRDTDSISGASTD

>human-Hs-p22*phox*: GenBank™ accession No. NM_000101

MGQIEWAMWANEQALASGLILITGGIVATAGRFTQWYFGAYSIVAGVFVCLLEYPRGKRKKGSTME
RWGQKYMTAVVKLFGPFTRNYYVRAVLHLLLSVPAGFLLATILGTACLAIASGIYLLAAVRGEQWTP

IEPKPRERPQIGGTIKQPPSNPPPRPPAEARKKPSEEEAAVAAGGPPGGPQVNPIPVTDEVV

>dog-Cf-p22*phox*: DDBJ™ No. BR000286

MGQIEWAMWANEQALASGLILIMGGIVATAGQFTKWYFGAYSIGAGVFVCLLEYPRGKRRKGSTM

ERCGQKYMTKVVKVFGPLSRNYYIRAFLHLGLSVPAGFLLATILGTACLAIASSIYLLAAYHGEQWIP

IEPQPKERPQVGGTIKQPPSNPPPRPPAEARKKPSEEEEAAGAVGVSGGPQENPVPVIDEVV

>zebrafish-Dr-p22*phox*: GenBank™ No. BC056702

MAKIEWAMWANEQALAAGLIYLTGGIVGVAGQFRGWQFAAFGIAAGVFVCLLEYPRSKRGKGTSIE

RSGQYCFTVCVKSFGPLTRNYYVRAFLHAALCVPGGFMLATVLGCVCLGMASLIYLSAPIHGEHWE

PILHIETKKRLGESIKEPPQNPPPRPPPELRRKKADNLDAAAYDNPMSVTINE

>mouse-Mm-p22*phox*: GenBank™ No. NM_007806

MGQIEWAMWANEQALASGLILITGGIVATAGRFTQWYFGAYSIAAGVLICLLEYPRGKRKKGSTME

RCGQKYLTSVVKLFGPLTRNYYVRAALHFLLSVPAGFLLATILGTVCLAIASVIYLLAAIRGEQWTPI

EPKPKERPQVGGTIKQPPTNPPPRPPAEVRKKPSEGEEEAASAGGPQVNPMPVTDEVV

>rat-Rn-p22*phox* GenBank™ No. NM_024160.1

MGQIEWAMWANEQALASGLILITGGIVATAGRFTQWYFGAYSIVAGVLICLLEYPRGKRKKGSTMER

CGQKYLTAVVKLFGPLTRNYYVRAVLHLLLSVPAGFLLATILGTVCLAIASVIYLLAAIRGEQWTPIEP

KPKERPQVGGTIKQPPTNPPPRPPAEVRKKPSEAEEEAASAGGPQVNPIPVTDEVV

>frog-Xt-p22*phox*_Ensembl No. ENSXETG00000017431

MGQIEWAMWANEQALASGLILLAGGIVAVAGQFKGWQFGAYGVAAGVFITLLEYPRSKRKKGSTM

ERCGQKYLAAVVKLFGPLTRNYYVRAILHAGLAVPGGFILSTILGTVCLGIASIIYFLAAIRGEEWRPI

EKQAEPKPRAGETIKRPPENPPPRPPAEVRRKQADEVSVGGGHVNPIPVTDNV

>tetraodon-Tn-p22*phox*: DDBJ™ BR000303

MGKIEWAMWANEQALASGFILLAGGIVGVAGRFRGWEFAAYAVAAGVFVCLLEYPRSKRSKGTSVE

RPGQHCFTVCVKAFGPVTKNYYVRAVLHAAICVPGGFMLATVLGCVCLGIASIIYLVAAIRGEHWEP

ILPKKEIQKPVAESIKNPPQNPPPRPPAETRRKRVDDLEAAAYDNP

>fugu-Tr-p22*phox*: GenBank™ No. AB099895

MGKIEWAMWANEQALASGFILLTGGVVGVAGQFRGWQFAAYAVAAGVLVCLLEYPRSKRSKGTSVE

RPGQRCFTVCVKAFGPVTRNYYVRAVLHAAICVPGGFMLATVLGCVCLGIASIIYLVAAIRGEHWEPI

LPSKEIRKPVAESIKNPPQNPPPRPPADTRRKRVEDLEAASYDNPISVTANE
