# Supplementary material for: Molecular evolution of the reactive oxygen-generating NADPH oxidase (Nox/Duox) family of enzymes
Source: BMC Evol Biol. 2007 Jul 6;7:109. doi: 10.1186/1471-2148-7-109 (PMC1940245; doi:10.1186/1471-2148-7-109)
Supplement: Additional File 11 — Exceptions of amino acid residues conserved in all Nox/Duox proteins. The data provided show exceptions of the 68 residues conserved in all Nox/Duox proteins that are indicated by asterisks in Figure 6. [file 1471-2148-7-109-S11.pdf]

Additional file 11

Exceptions of amino acid residues conserved in all Nox/Duox proteins

We identified 68 amino acid residues that were highly conserved in eukaryote Noxes and showed them in Figure 6. Possibly due to mis-sequencing of the part of the genome or to tolerance of evolutionary changes in the species, some of conserved amino acids contained rare exceptions, as indicated by *asterisks* in Figure 6. The residue numbers shown below correspond to the numbers that are shown in Figure 6 with *asterisks*. The single letter amino acid code is used. Sequences of *A. thaliana* rbohH and *T. nigroviridi*s Nox5 are truncated in the C-terminus (alignment was shown in Additional file 5), and they failed to conserve many of the identified 68 amino acids (9 residues of *A. thaliana* rbohH and 16 residues of *T. nigroviridi*s Nox5). Therefore, we did not list exceptions in these two sequences here.

*1 = L (*C. elegans* Duox2), *2 = L (*C. familliaris* Duox1 and *X. tropicalis* Duox2), *3 =A (*X. tropicalis* Nox4), *4 = I/T (*C. elegans* Duox1/2, respectively), *5 = P (*G. gallus* Nox1), K (*X. tropicalis* Nox1), *6 = T (*C. familliaris* Nox4), *7 = G (*G. gallus* Nox1), M (*X. tropicalis* Nox1), *8 = I (*R. norvegicus* Nox3), *9 = deletion (*C. familliaris* Nox4), *14 = S (*X. tropicalis* Duox2), *11 =M (*X. tropicalis* Nox2), *12 = T (*D.

discoideum NoxA), *13 = M (*D. rerio* Nox2), *14 = F (*P. anserine* NoxB, *M. grisea* NoxB), *15 = M (*T. nigroviridis* Nox1, *T. rubripes* Nox1, *O. latipes* Nox1), *16 = A (*C. elegans* Duox1/2), *17 = S (*A. thaliana* rbohB), *18 = Y (*C. elegans* Duox1/2), *19 = R (*T. nigroviridis* Nox5), *20 = Q (*T. nigroviridis* Nox5), *21 = A (*T. nigroviridis* Nox5), * 22 = E (*T. nigroviridis* Nox5), *23 = T (*C. intestinalis* Nox4), *24 = S (*C. crispus* NoxD), *25 = C (*C. crispus* NoxD), *26 = M (*A. thaliana* rboh H and J), *27 = A (*P. yezoensis* NoxD), *28 = Y (*O. latipes* Nox2), *29 = L (*C. intestinalis* Nox4), *30 = R (*O. latipes* Nox2), *31 = S (*C. intestinalis* Nox4), *32 = deletion (*R. norvegicus* Nox2), L (*O. latipes* Nox2), *33 = M (*O. latipes* Nox2), *34 = M (*C. intestinalis* DuoxD), *35 = T (*T. nigroviridis*, *T. rubripes, D. rerio* Nox1), *36 = D (*C. familliaris* Duox2), *37 = M (*S. purpuratus*-Nox5B), *38 = P (*C. familliaris* Duox2), Y (*C. intestinalis* Duox-D), *39 = W (*F. graminearum* NoxC), *40 = deletion (*P. yezoensis* NoxD), *41 = L (*D. discoideum* NoxB), *42 = deletion (*C. familliaris* Duox2, *G. gallus* Duox1, *C. intestinalis* Duox-A), *43 = D (*F. graminearum* NoxC), *44 = deletion (*P. yezoensis* NoxD), *45 = deletion (*T. rubripes* Nox4), *46 = deletion (*C. familliaris* Duox2, *G. gallu* Duox1, *C. intestinalis* Duox-A), *47 = Y (*F. graminearum* NoxC), *48 = deletion (*P. yezoensis* NoxD).
