# Supplementary material for: Molecular evolution of the reactive oxygen-generating NADPH oxidase (Nox/Duox) family of enzymes
Source: BMC Evol Biol. 2007 Jul 6;7:109. doi: 10.1186/1471-2148-7-109 (PMC1940245; doi:10.1186/1471-2148-7-109)
Supplement: Additional File 2 — Supplemental Figure S2. The figure provided shows sequences of EF-hand motifs in Nox5, Duox, plant Nox, and NoxC [file 1471-2148-7-109-S2.pdf]

Additional file 2

Supplemental Figure S2. Sequences of EF-hand motifs in Nox5, Duox, plant Nox, and NoxC

The original alignment data are shown in Additional file 8. EF (I) to EF (IV) represent four EF-hands of Nox5. Each EF-hand domain contains a twelve residue loop that is involved in calcium-binding. The letters X, Y, Z, -Y, -X, and -Z indicate the six residues that directly participate in calcium-binding, and these six residues correspond to positions 1, 3, 5, 7, 9 and 12. The consensus sequences of these six positions are: D at position 1, D/N/S at position 3, D/E/N/S/T/G at position 5, G/P at position 7, D/E/N/Q/S/T/A/G/C at position 9, and D/E at position 12 (PROSITE documentation number PDOC00018), and the most conserved positions are 1, 3, and 12. *Asterisks* indicate atypical EF-hand motif that is not consistent with the consensus sequences at positions 1, 3, or 12. Predicted *T. rubripes* (Tr)-Nox5 sequence was not a complete sequence; therefore the 1st EF-hand motif sequence was not shown. The *arrows* indicate motifs that have 17 to 23 additional amino acids inserted in the indicated position. Abbreviations of species are as in Figure 2.

	EF-I	EF-II	EF-III	EF-IV
	X Y Z-Y-X-Z	X Y Z-Y-X -Z	X Y Z-Y-X -Z	X Y Z-Y-X -Z
**human-Hs-Nox5α**	AG-EDGEISLQE*	DSDRSGTITLQE	DIDGSGSIDPDE	DADGNGAITFEE
**dog-Cf-Nox5**	AE-KDREINLQQ*	DSDGSGTITLQE	DVDGSGSIDADE	DKDCNGAITFDE
**cow-Bt-Nox5**	AG-EDGEINLQD*	DSDGSGTITLQE	DVDGSGSIDADE	DKDCSGTITFEE
**chicken-Gg-Nox5**	AG-HDEEIGLEE*	DVDGSGTISLAE	DVDGSGSIDAAE	DQDGNGSITFQE
**frog-Xt-Nox5**	AG-DDKEIDLEE*	DSDGSGSISLDE	DVDGSGSIDPSE	DKDHSGSITFQE
**opposum-Md-Nox5**	AG-EDREIDLQE*	DSDGSGTITLQE	DVDGNGSIDPDE	DKDHSGSITFEE
**fugu-Tr-Nox5**	------------	DSDGSSSISLDE	DVDGSGSIDPDE	DKDHSGSITFEE
**medaka-Ol-Nox5**	AG-DDKEINLCE*	DSDGSGSISLDE	DVQGSGSIDPDE*	DTDNSGSITFEE
**sea urchin-Sp-Nox5A**	AG-EDRQIDEDE*	DQDGSGYISLDE	DVDGNGAIDHEE	DTDGSGAISFEE
**sea urchin-Sp-Nox5B**	AG-DDNLIDLDE*	DTDQSGSISLKE	DVDGSGFIDFDE	DVDGDGEVSFEE
**fruit fly-Dm-Nox5**	VG-NEQEIRREE*	DKDNSGSISLQE	DIDGDGLIQHKE	DPHNSGEITYEA*
**mosquito-Ag-Nox5**	VG-NEKEIRREE*	DKDNSGSISLQE	DLDGDGLIQHRE	DKYNRGAITYEA*
**honeybee-Am-Nox5**	VG-NEKEIRREE*	DKDNSGTISLQE	DIDGDGLIQLRE	DQSNRGAITFEA*
**fungus-Mg-NoxC**	------------	------------	DHDGDGCIDYSE	------------
**fungus-Fg-NoxC**	------------	------------	DHDNDGHINYEE	------------
**At-rbohF**	------------	------------	DKNEDGRITEEE	DPERLGYIELWQ*
**At-rbohI**	------------	------------	CYQLSSNLVKHI*	APDGLYYIELKD*
**At-rbohC**	------------	------------	DKDADGRLTEDE	DPDNIGYIMLES*
**At-rbohG**	------------	------------	DKDSDGRLTEDE	DPDHMGYIMMES*
**At-rbohA**	------------	------------	DKDSDGRLNEAE	DPYHYGYIMIEN*
**At-rbohD**	------------	------------	DKDEDGRVTEEE	DPDNAGFIMIEN*
**At-rbohB**	------------	------------	DKNLDGRITGDE	DRDNLGYIELHN*
**At-rbohE**	------------	------------	DSNEDGKITREE	DPENFGYIELWQ*
**At-rbohH**	------------	------------	DKNGDGKLTEEE	DPDHKGYIEMWQ*
**At-rbohJ**	------------	------------	DKDGDGKLTEEE	DPNEQGYIEMWQ*
**amoeba-Dd-NoxC**	------------	------------	DIYDKGFISRDD*	DKNMDGYIDFEE
**mouse-Mm-Duox1**	------------	DKDGNGYLSFRE	DFDGNGLISKDE	------------
**mouse-Mm-Duox2**	------------	DKDGNGYISFRE	DLDGNGFLSKDE	------------
**dog-Cf-Duox1**	------------	DKDGNGYLSFRE	DFDGNGLISKDE	------------
**dog-Cf-Duox1**	------------	DKDGNGYLSFRE	DLDANGFLSKDE	------------
**human-Hs-Duox1**	------------	DKDGNGYLSFRE	DFDGNGLISKDE	------------
**human-Hs-Duox2**	------------	DKDGNGYLSFRE	DLDENGFLSKDE	------------
**rat-Rn-Duox1**	------------	DKDGNGYLSFRE	DFDGNGLISKDE	------------
**rat-Rn-Duox2**	------------	DKDGNGYISFRE	DLDGNGFLSKEE	------------
**chicken-Gg-Duox**	------------	DKDGNGYISFRE	DIDENGFLSKEE	------------
**frog-Xt-Duox1**	------------	DKDHNGYLSFEE	DVNGNGILPKEE	------------
**frog-Xt-Duox2**	------------	DEDGNGYLSFRE	DVDGNGFLSKEE	------------
**fugu-Tr-Duox**	------------	DKDGNGSLSFQE	DIGGTGSLSKGE*	------------
**tetraodon-Tn-Duox**	------------	DKDGNGYLSFQE	------------	------------
**zebrafish-Dr-Duox**	------------	DKDGNGYLSFQE	DIKGDGFLSKEE*	------------
**medaka-Ol-Duox**	------------	DTDHSGYLSFQE	DVGGNGYLSKEE	------------
**ascidian-Ci-Duox-B**	------------	DTDHSGYLSFRE	DVDHSGEINREE	------------
**ascidian-Ci-Duox-A**	------------	DSDGSGAISFRE	DLDKSGELSKKE	------------
**ascidian-Ci-Duox-C**	------------	DSDEDGTISFRE	DLDKSGGLSKEE	------------
**ascidian-Ci-Duox-D**	------------	DEDQDGFISFHD	DLNQNGSLTKQQ*	------------
**sea urchin-Sp-Duox**	------------	DQDNSGSISFRE	DIDRSGHLSREE	------------
**fuit fly-Dm-Duox**	------------	DKDQDGRISFQE	DNDRNGVIDKGE	------------
**mosquito-Ag-Duox**	------------	DKDKDGRISFQE	DNDRNGVIDKGE	------------
**honeybee-Am-Duox**	------------	DKDRDGRISFQE	DKDCNGVIDKEE	------------
**nematode-Ce-Duox1**	------------	AKHNEDSLSFNE*	DLEGKNKVLRKD*	------------
**nematode-Ce-Duox2**	------------	AKHNEDSLSFNE*	DLEGKNKVLRKD*	------------
