# Supplementary material for: Molecular evolution of the reactive oxygen-generating NADPH oxidase (Nox/Duox) family of enzymes
Source: BMC Evol Biol. 2007 Jul 6;7:109. doi: 10.1186/1471-2148-7-109 (PMC1940245; doi:10.1186/1471-2148-7-109)
Supplement: Additional File 8 — Alignment of EF-hand regions of plant Nox, NoxC, Nox5, and Duox. Alignment of EF-hand region of plant Nox, NoxC, Nox5, and Duox are provided. EF-hand motif regions and non-EF-hand "helix-loop-helix (HLH)" structure regions are shown. [file 1471-2148-7-109-S8.pdf]

Additional file 8

Alignment of EF-hand regions of plant Nox, NoxC, Nox5, and Duox

Gray-boxes indicate EF-hand motifs. EF-I to EF-IV shown above the alignment correspond to the number of the four EF-hands of Nox5. *Underlines* represent a “helix-loop-helix (HLH)” region of Duox orthologs, and *Bold letters* represent the loop region of the “HLH” region. The letters X, Y, Z, -Y, -X, and -Z indicate the six residues that participate in calcium-binding. The 1st transmembrane domains (the 2nd for Duox) were indicated as TM1 (or TM2 for Duox) above the alignment. To describe species of the genes, we use the same abbreviations as in Additional file 4.

```
                                                      -EF-I
                                                      X
human-Nox5alpha         QTGPEGCRGTMSAEEDARWLRWVTQQFKTIAGE--------------------------- 41
dog-Nox5                ----------MSTEEDAKWLQWVTHQFETIAEK--------------------------- 36
cow-Nox5                ----------MNAEEDAKWLQWVTHQFKTIAGE--------------------------- 41
chicken-Nox5            ----------MGTAEDAAWLRWVTERFQSIAGH--------------------------- 32
frog-Nox5               ----------MSTEDDSKWLEWVTKQFENIAGD--------------------------- 24
opossum-Nox5            ----------MNADDDAKRLEWVSFQFE-IAGE---------------------------
fugu-Nox5               ------------------------------------------------------------
medaka-Nox5             SSGCSGVSCIMSVDEDARWLEWVTKQFESIAGD--------------------------- 44
Sp-Nox5A                LAFSSVCGNAQPADEDSQWLSWAEKQFCQIAGE--------------------------- 42
Sp-Nox5B                --------MAGMQEKDEKWLVKLEKHFQEVAGD--------------------------- 25
fruit fly-Nox5          ---SHLTNSRPRTGFDKSSLARLEQLFRTTVGN--------------------------- 90
mosquito-Nox5           ---SNLTNSRPRTGFDKSSLERLEQLFIKTVGN--------------------------- 89
honeybee-Nox5           ---SNISDTRPLAGFDKRSLEWLEKIFKQTVGN--------------------------- 88
Mg-NoxC                 ---TAAASPNDEHVKIEPSTKDKGEDKPKAADA--------------------------- 50
Fg-NoxC                 ------------------------------------------------------------
At-rbohF                LRKQRAQLDRTRSS-AQRALRGLRFISNKQKNV-----------------------DGWN 97
At-rbohI                LSQRPTRPNRDGSG-TERAIHGLKFISSKENGI-----------------------VDWN 186
At-rbohC                PRP--AKLDRTKSA-ASQALKGLKFISKTDGG------------------------AGWS 158
At-rbohG                PR-----LDRSKST-AGQALKGLKIISKTDGN------------------------AAWT 108
At-rbohA                PRPQLAKLRRSKSR-AELALKGLKFITKTDGV------------------------TGWP 153
At-rbohD                PSPAVRRFDRTSSA-AIHALKGLKFIATKT--------------------------AAWP 183
At-rbohB                ------RLDRSKSFGAMFALRGLRFIAKNDAVG-----------------------RGWD 104
```

```
At-rbohE           KRKEDAKLQRSTSS-AQRALKGLQFINKTTRGNSC------------VCDWDCDCDQMWK 201
At-rbohH           KKTGPQRVERTTSS-AARGLQSLRFLDRTVTGRE---------------------RDAWR 127
At-rbohJ           KRPGPQRVERTTSS-AARGLQSLRFLDRTVTGRE---------------------RDSWR 137
Dd-NoxC            NNNNNNNNNNNNNNYNNNIDNNNNNNNNNNNNNINNCNNNNINNDNNNNNNNDNDNNNIN 273
mouse-Duox1        --------FRHLFSQVLDINQADAGTLPLDSST--------------------------- 792
mouse-Duox2        --------FRQLFAQVLDINQADAGTLPLDSSQ--------------------------- 796
rat-Duox1          --------FRHLFSQVLDINQADAGTLPLDSST--------------------------- 788
dog-Duox1          --------FRHLFSQVLDIDQADAGTLPLDSSQ--------------------------- 792
human-Duox1        --------FRHLFSQVLDINQADAGTLPLDSSQ--------------------------- 792
dog-Duox2          --------FRYLFAQVLDIDQADAGTLPLNSSQ--------------------------- 555
human-Duox2        --------FRHLFAQVLDINQADAGTLPLDSSQ--------------------------- 796
rat-Duox2          --------FRQLFAQVLDINQADAGTLPLDSSQ--------------------------- 796
chicken-Duox       --------FRHLFARMLDIDKTDAGELNFESSQ--------------------------- 764
frog-Duox1         --------IRHSLSHVIDINKEHAGTT---QGQ--------------------------- 759
frog-Duox2         --------FRQSFAQVLHITQSDN-MG---SSD--------------------------- 759
tetraodon-Duox     --------IRHAFSKVLEIDKCDAGDMSDVSRK843
fugu-Duox          --------IRHAFSKVLEIDKCDAGDMSGVSRK--------------------------- 769
zebrafish-Duox     --------FKHAFSKVLDIDKSDAGDLSSRTR---------------------------- 769
medaka-Duox        --------FSKVLEIQKCDAGDMSGVSPRRARE--------------------------- 800
Ci-Duox-B          --------LRQTFAEALNIDDAVEQNSKAKYRH--------------------------- 773
Ci-Duox-A          --------FKAALIAVSSIKVEDGDMSDVINSR--------------------------- 749
Ci-Duox-C          --------FRTALSQVA-MNIESEDL-DRPKRK--------------------------- 686
Ci-Duox-D          ---------SMALTSVVLQSMRMEFG--IQISS--------------------------- 702
Sp-Duox            --------FLMAFSQAFKLDFDPENLESLNNKETK------------------------- 919
fruit fly-Duox     --------FREAYALTFGLRPGERRRRSDASSDG-------------------------- 770
mosquito-Duox      --------FREAYALTFGLRPGERRRRSDASLDG-------------------------- 771
honeybee-Duox      --------FREAYALTFGLRPGERRRRSEDSDSG-------------------------- 775
Celeg-Duox2        --------FREAYAKAFND---SELQDSETSFDS-------------------------- 802
Celeg-Duox1        --------FREAYAKAFND---SELQDSETSFDS-------------------------- 794

                                --EF-I--                      -EF-II-
                                Z-Y-X -Z                      X Y Z-Y
human-Nox5alpha    -------------DGEISLQEFKAALHVKES--FFAERFFALFDSDRSGT---------- 76
dog-Nox5           -------------DREINLQQFKTALNVKEAI-LFAERFFTLFDSDGSGT---------- 72
cow-Nox5           -------------DGEINLQDFKKALKVKEASSFFAERFFVLFDSDGSGT---------- 78
chicken-Nox5       -------------DEEIGLEEFKAALQVKES--FFAERFFALFDVDGSGT---------- 67
frog-Nox5          -------------DKEIDLEEFKTALKVKES--FFAERFFALFDSDGSGS---------- 59
opossum-Nox5       -------------DREIDLQEFKKALKVKES--FFAERFFALFDSDGSGT---------- 57
fugu-Nox5          ---------------------------------FFAERFFALFDSDGSSS---------- 17
medaka-Nox5        -------------DKEINLCEFKTALKVKES--FFAERFFALFDSDGSGS---------- 79
Sp-Nox5A           -------------DRQIDEDEFKMALNIKKS--FFAERFFHLFDQDGSGY---------- 77
Sp-Nox5B           -------------DNLIDLDEFINALNVKKS--FFAERFFELIDTDQSGS---------- 60
fruit fly-Nox5     -------------EQEIRREEFQKIVTSKNP--FFTERVFQIFDKDNSGS---------- 125
mosquito-Nox5      -------------EKEIRREEFKKIVTSKNP--FFTERVFQIFDKDNSGS---------- 124
honeybee-Nox5      -------------EKEIRREEFNKIVTSKNP--FFTDRVFQIFDKDNSGT---------- 123
Mg-NoxC            -------------RDDGSEAEARADQHPEKPAQANGPETADGDHHPFANS---------- 87
Fg-NoxC            --------------------KSVAADHPVRSSQ--------------------------- 21
At-rbohF           DVQSNFEKFEK--NGYIYRSDFAQCIGMKD----SKEFALELFDALSRRRRLKVEK---- 247
At-rbohI           DVQNNFAHLSK--DGYLFKSDFAHCIGLENEN--SKEFADELFDALCRRRRIMVDK---- 238
At-rbohC           AVEKRFNQITATTGGLLLRTKFGECIGMT-----SKDFALELFDALARRRNITGEV---- 209
At-rbohG           VVEKRYLKITANTDGLLLRSKFGECIGMN-----SKEFALELFDALARKSHLKGDV---- 159
At-rbohA           EVEKRFYVMTMTTNGLLHRSRFGECIGMK-----STEFALALFDALARRENVSGDS---- 204
At-rbohD           AVDQRFDKLSADSNGLLLSAKFWECLGMNKE---SKDFADQLFRALARRNNVSGDA---- 236
At-rbohB           EVAMRFDKLAV--EGKLPKSKFGHCIGMVE----SSEFVNELFEALVRRRGTTSSS---- 154
```

```
At-rbohE                KVEKRFESLSK--NGLLARDDFGECVGMVD----SKDFAVSVFDALARRRRQKLEK---- 251
At-rbohH                SIENRFNQFSV--DGKLPKEKFGVCIGMGD----TMEFAAEVYEALGRRRQIETENG--- 178
At-rbohJ                SIENRFNQFAV--DGRLPKDKFGVCIGMGD----TLEFAAKVYEALGRRRQIKTENG--- 188
Dd-NoxC                 TVDNHDDDIINNSNNFNKNEYPSSNISPISPKSSISSFPTNLNNSINNTGSMVSDSLSSC 333
mouse-Duox1             -------KVREALTCELSRAEFADSLGLKPQD-MFVESMFSLADKDGNGY---------- 834
mouse-Duox2             -------QVREALTCELSRAEFADSLGLKPQD-MFVESMFSLADKDGNGY---------- 838
rat-Duox1               -------KVREALTCELSRAEFADSLGLKPQD-MFVESMFSLADKDGNGY---------- 830
dog-Duox1               -------KVQEALTCELSRAEFAESLGLKPQD-MFVESMFSLADKDGNGY---------- 834
human-Duox1             -------KVREALTCELSRAEFAESLGLKPQD-MFVESMFSLADKDGNGY---------- 834
dog-Duox2               -------KVREALTCELSRAEFAESLGLKPQD-MFVESMFSLADKDGNGY---------- 597
human-Duox2             -------KVREALTCELSRAEFAESLGLKPQD-MFVESMFSLADKDGNGY---------- 838
rat-Duox2               -------QVREALTCELSRAEFADSLGLKPQD-MFVESMFSLADKDGNGY---------- 838
chicken-Duox            -------KARESLTCELSRAEFAEALGLKVNS-MFVDSMFSLADKDGNGY---------- 806
frog-Duox1              -------NFRDVLQCELSREEFADSLGLNPNA-QFVESMFSMADKDHNGY---------- 801
frog-Duox2              -------KVKEALKCELSRTEFAESLGLKPQS-MFVESMFSLADEDGNGY---------- 831
tetraodon-Duox          -------KAKEVLQCELTAGEFADALGLKPDS-LFVDSMFTLADKDGNGY---------- 885
fugu-Duox               -------KAKEVLQCELTAAEFADALGLKADS-LFVDSMFTLADKDGNGS---------- 810
zebrafish-Duox          ----------EALQCELTRAEFASVLGLKSDS-LFVESMFTLADKDGNGY---------- 808
medaka-Duox             -----------VLQCELTASEFADALCLKPDS-LFVDSMFTLADKDGNGY---------- 836
Ci-Duox-B               -------KTMEVADCELTQMELAETMGMKPDS-LFVTQMFQLADTDHSGY---------- 815
Ci-Duox-A               -------EARKFIHVELSKKEFADYLKLKPNS-LFVDQMFSSADSDGSGA---------- 791
Ci-Duox-C               -------TLKDLMKVNITKSEFAEYLKLKEDS-LFVEQMFLVADSDEDGT---------- 728
Ci-Duox-D               -------FPENLVDIKLSQEEFASLLQMKSSS-LFVQHIFTTADEDQDGF---------- 744
Sp-Duox                 -------ETKDILECELTKTEFADVLTMKPDS-LFVEQMFELVDQDNSGS---------- 958
fruit fly-Duox          -------EVMTVMRTSLSKAEFAAALGMKPND-MFVRKMFNIVDKDQDGR---------- 812
mosquito-Duox           -------EVMTVMRTSLSKSEFAAALGMKQDD-MFVRKMFNIVDKDKDGR---------- 813
honeybee-Duox           -------EVVTVMRTSLSKSEFASALGMRADA-VFVKKMFNIVDKDRDGR---------- 817
Celeg-Duox2             -------SNDDILNETISREELASAMGMKANN-EFVKRMFAMTAKHNEDS---------- 844
Celeg-Duox1             -------SNDDILNETISREELASAMGMKANN-EFVKRMFAMTAKHNEDS---------- 836

                                                     EF-II-                      EF-III
                                                     -X -Z                       X
human-Nox5alpha         -----------------------------ITLQELQEALTLLIHGSPMDKLKFLFQVYDI 107
dog-Nox5                -----------------------------ITLQELLEALTLLIHGNPMDKLKFLFQVYDV 103
cow-Nox5                -----------------------------ITLQELQKALTLLIHGSPMDKLKFLFQVYDV 109
chicken-Nox5            -----------------------------ISLAELHGALALLLRGTAADKLRFLFQVYDV 98
frog-Nox5               -----------------------------ISLDELLKALNLLIHGNETDKLRFLFQVYDV 90
opossum-Nox5            -----------------------------ITLQELLGALNLLIHGNTMDKLKFLFQVYDV 88
fugu-Nox5               -----------------------------ISLDELLKALDLLIHGSETDKLRFLFQVYDV 48
medaka-Nox5             -----------------------------ISLDELLKALDLLIHGTETDKLRFLFQVYDV 110
Sp-Nox5A                -----------------------------ISLDELMEGLYLLTKGDPVDKLRFLFSVYDV 108
Sp-Nox5B                -----------------------------ISLKELIGALRLLVNGTEQEKLHFLFQVYDV 91
fruit fly-Nox5          -----------------------------ISLQEFIDAIHQFSGGQSADDKIRFLFKVYDI 156
mosquito-Nox5           -----------------------------ISLQEFIDAIHQFAGQSPEDKIKFLFKVYDL 155
honeybee-Nox5           -----------------------------ISLQEFVDAMHQFAGKSPDDKIKFLFKVYDI 154
Mg-NoxC                 -----------------------------YQVDSARAADDPNVVYLRDDEIETFLDELDH 118
Fg-NoxC                 -----------------------------------------NVEFLTDKEISDFLDDLDH 40
At-rbohF                -----------------------------INHDELYEYWSQINDESFDSRLQIFFDIVDK 278
At-rbohI                -----------------------------INLQELYEFWYQITDESFDSRLQIFFNMYCY 269
At-rbohC                -----------------------------IDGDQLKEFWEQINDQSFDSRLKTFFDMVDK 240
At-rbohG                -----------------------------ITETELKKFWEQINDKSFDSRLITFFDLMDK 190
At-rbohA                -----------------------------ININELKEFWKQITDQDFDSRLRTFFAMVDK 235
At-rbohD                -----------------------------ITKEQLRIFWEQISDESFDAKLQVFFDMVDK 267
At-rbohB                -----------------------------ITKTELFEFWEQITGNSFDDRLQIFFDMVDK 185
```

```
At-rbohE          -----------------------------ITKDELHDFWLQISDQSFDARLQIFFDMADS 282
At-rbohH          -----------------------------IDKEQLKLFWEDMIKKDLDCRLQIFFDMCDK 209
At-rbohJ          -----------------------------IDKEQLKLFWEDMIKKDLDCRLQIFFDMCDK 219
Dd-NoxC           RNSISSSSIDSSVASIPITIQSIDFEDKNIKSDQFKIISKSNIENTIETNPIPPFNQTNN 393
mouse-Duox1       -----------------------------LSFREFLDILVVFMKGSPEEKSRLMFRMYDF 865
mouse-Duox2       -----------------------------ISFREFLDILVVFMKGSSEDKSRLMFTMYDL 869
rat-Duox1         -----------------------------LSFREFLDILVVFMKGSPEEKSRLMFRMYDF 861
dog-Duox1         -----------------------------LSFREFLDILVVFMKGSPEEKSRLMFRMYDF 865
human-Duox1       -----------------------------LSFREFLDILVVFMKGSPEEKSRLMFRMYDF 865
dog-Duox2         -----------------------------LSFREFLDILVVFMKGSPEDKSRLMFTMYDL 628
human-Duox2       -----------------------------LSFREFLDILVVFMKGSPEDKSRLMFTMYDL 869
rat-Duox2         -----------------------------ISFREFLDILVVFMKGSPQDKSRLMFTMYDL 869
chicken-Duox      -----------------------------ISFREFLDILVVFMKGSSEEKSKLMFRMYDI 837
frog-Duox1        -----------------------------LSFEEFCFILCSLIKGSAEDKLKFIFSMHDV 832
frog-Duox2        -----------------------------LSFREFLNILVIFMTGTPKEKSELMFKMYDV 862
tetraodon-Duox    -----------------------------LSFQEFLDVIVIFMKG--------------- 901
fugu-Duox         -----------------------------LSFQEFLDVIVIFMKGSSEEKSSLMFSMNDI 841
zebrafish-Duox    -----------------------------LSFQEFLDVIVIFMTGTSEEKSKLLFSTHDI 839
medaka-Duox       -----------------------------LSFQEFLDVMVIFMKGSPEEKSKLMFSMNDV 869
Ci-Duox-B         -----------------------------LSFREFADLIILLMNGSPEQKAKMLFDMYDV 846
Ci-Duox-A         -----------------------------ISFREFLDIMVLFTKGSPEEKAKLMFNMYDL 822
Ci-Duox-C         -----------------------------ISFREFLDIIVLFTKGTPKEKAQLMFNMYDL 759
Ci-Duox-D         -----------------------------ISFHDFRKIIVLFVKGSPNEKLRLLFDMFDL 775
Sp-Duox           -----------------------------ISFREFLDVIVVFAKGQPEDKLKLMFNMYDI 989
fruit fly-Duox    -----------------------------ISFQEFLETVVLFSRGKTDDKLRIIFDMCDN 843
mosquito-Duox     -----------------------------ISFQEFLETVVLFSRGKTDDKLRIIFDMCDN 844
honeybee-Duox     -----------------------------ISFQEFLDTVLLFSRGKTEDKLRIIFDMCDK 848
Celeg-Duox2       -----------------------------LSFNEFLTVLREFVNAPQKQKLQTLFKMCDL 875
Celeg-Duox1       -----------------------------LSFNEFLTVLREFVNAPQKQKLQTLFKMCDL 867

                  -
                  Y
human-Nox5alpha   D----------------------------------------------------------- 108
dog-Nox5          DGWARQGGVCRYRVGPSTRP---------------------------------------- 123
cow-Nox5          DVCARQGAFASTEGAGAG------------------------------------------ 127
chicken-Nox5      D----------------------------------------------------------- 99
frog-Nox5         DIWCLCPPTGSRTDSEWNSRHTVH------------------------------------ 114
opossum-Nox5      DGLCYRSYSPS-TGSQWSLGKCSS------------------------------------ 111
fugu-Nox5         D----------------------------------------------------------- 49
medaka-Nox5       D----------------------------------------------------------- 111
Sp-Nox5A          D-----------------------------------------------------------
Sp-Nox5B          D-----------------------------------------------------------
fruit fly-Nox5    D----------------------------------------------------------- 157
mosquito-Nox5     D----------------------------------------------------------- 156
honeybee-Nox5     D----------------------------------------------------------- 155
Mg-NoxC           N----------------------------------------------------------- 119
Fg-NoxC           D----------------------------------------------------------- 41
At-rbohF          N----------------------------------------------------------- 279
At-rbohI          Q----------------------------------------------------------- 270
At-rbohC          D----------------------------------------------------------- 241
At-rbohG          D----------------------------------------------------------- 191
At-rbohA          D----------------------------------------------------------- 236
At-rbohD          D----------------------------------------------------------- 268
At-rbohB          N----------------------------------------------------------- 186
```

```
At-rbohE                N----------------------------------------------------------- 283
At-rbohH                N----------------------------------------------------------- 210
At-rbohJ                D----------------------------------------------------------- 220
Dd-NoxC                 QCEVQLQSHSLPTILKQPHIYKSKSFSSSINSNSKIKKIKKSRSFEIESKINLFDVINHI 453
mouse-Duox1             D----------------------------------------------------------- 866
mouse-Duox2             D----------------------------------------------------------- 870
rat-Duox1               D----------------------------------------------------------- 862
dog-Duox1               D----------------------------------------------------------- 866
human-Duox1             D----------------------------------------------------------- 866
dog-Duox2               D----------------------------------------------------------- 629
human-Duox2             D----------------------------------------------------------- 870
rat-Duox2               D----------------------------------------------------------- 870
chicken-Duox            D----------------------------------------------------------- 838
frog-Duox1              N----------------------------------------------------------- 833
frog-Duox2              D----------------------------------------------------------- 863
tetraodon-Duox          ------------------------------------------------------------
fugu-Duox               G----------------------------------------------------------- 842
zebrafish-Duox          K----------------------------------------------------------- 840
medaka-Duox             G----------------------------------------------------------- 870
Ci-Duox-B               D----------------------------------------------------------- 847
Ci-Duox-A               D----------------------------------------------------------- 823
Ci-Duox-C               D----------------------------------------------------------- 760
Ci-Duox-D               N----------------------------------------------------------- 776
Sp-Duox                 D----------------------------------------------------------- 990
fruit fly-Duox          D----------------------------------------------------------- 844
mosquito-Duox           D----------------------------------------------------------- 845
honeybee-Duox           D----------------------------------------------------------- 849
Celeg-Duox2             E----------------------------------------------------------- 876
Celeg-Duox1             E----------------------------------------------------------- 868

                                                --EF-III-
                                                 Z-Y-X -Z
human-Nox5alpha         ------------------------GSGSIDPDELRTVLQSCLRESAISLPDEKLDQLTLA 144
dog-Nox5                ---------------HSAASPLGTGSGSIDADELRTVLRSCMRESAISLPDEKLDQLTLA 168
cow-Nox5                ---------------PQASSSPETGSGSIDADELRTVLQSCLYESAISLPKEKLDQLTLA 172
chicken-Nox5            ------------------------GSGSIDAAELLLVLRACLRESAISLPPQRLHDMARV 135
frog-Nox5               --------------WQCLEPFSFPGSGSIDPSELRTVLKSCLRESAISLPEEKLDDLTLV 160
opossum-Nox5            --------------------PLGHGNGSIDPDELRVVLQSCLKESAISLPEEKLDDLTLA 151
fugu-Nox5               ------------------------GSGSIDPDELRIVLKSCLRESAISLPEEKLDDLTLV 85
medaka-Nox5             ----------------ACFMMKVTGSGSIDPDELRTVLKSCLSESAISLPEEKLDDLTLV 153
Sp-Nox5A                ------------------------GNGAIDHEELKVVLRACLCESSMTISEATIDALTSA 95
Sp-Nox5B                ------------------------GSGFIDFDELKTVLRSCTAESAMTLCDETLTELTEI 78
fruit fly-Nox5          ------------------------GDGLIQHKELHDVIRHCIKENGMEFSEDQIEDLTSA 193
mosquito-Nox5           ------------------------GDGLIQHRELQHVMRACMEENGMRFSEDQIEDLTMA 192
honeybee-Nox5           ------------------------GDGLIQLRELEHVMRACLEENGIRFSEEQIEELTMA 191
Mg-NoxC                 ------------------------GDGCIDYSEVERKLDEVHDELAPTAQPHHLHHD--S 153
Fg-NoxC                 ------------------------NDGHINYEEVERKLDQEHANLVPKPSAHHVISTDHS 77
At-rbohF                ------------------------EDGRITEEEVKEIIMLSASANKLSRLKEQAEEYAAL 315
At-rbohI                ------------------------LSSNLVKHIDQHIIILSASANNLSRLRERAEEYAAL 306
At-rbohC                ------------------------ADGRLTEDEVREIISLSASANNLSTIQKRADEYAAL 277
At-rbohG                ------------------------SDGRLTEDEVREIIKLSSSANHLSCIQNKADEYAAM 227
At-rbohA                ------------------------SDGRLNEAEVREIITLSASANELDNIRRQADEYAAL 272
At-rbohD                ------------------------EDGRVTEEEVAEIISLSASANKLSNIQKQAKEYAAL 304
At-rbohB                ------------------------LDGRITGDEVKEIIALSASANKLSKIKENVDEYAAL 222
```

```
At-rbohE          ------------------------EDGKITREEIKELLMLSASANKLAKLKEQAEEYASL 319
At-rbohH          ------------------------GDGKLTEEEVKEVIVLSASANRLGNLKKNAAAYASL 246
At-rbohJ          ------------------------GDGKLTEEEVKEVIVLSASANRLVNLKKNAASYASL 256
Dd-NoxC           YLNLSKVGSEEQKITSVFKLYDIYDKGFISRDDLKEVLNYRTKQNGLKFQDFTMESLIDH 513
mouse-Duox1       ------------------------GNGLISKDEFIRMLRSFIEISNNCLSKAQLAEVVES 902
mouse-Duox2       ------------------------GNGFLSKDEFFTMMRSFIEISNNCLSKAQLAEVVES 906
rat-Duox1         ------------------------GNGLISKDEFIRMLRSFIEISNNCLSKDQLAEVVES 898
dog-Duox1         ------------------------GNGLISKDEFIRMLRSFIEISNNCLSKAQLTEVVES 902
human-Duox1       ------------------------GNGLISKDEFIRMLRSFIEISNNCLSKAQLAEVVES 902
dog-Duox2         ------------------------ANGFLSKDEFFTMMRSFIEISNNCLSKAQLTEVVES 665
human-Duox2       ------------------------ENGFLSKDEFFTMMRSFIEISNNCLSKAQLAEVVES 906
rat-Duox2         ------------------------GNGFLSKEEFFTMMRSFIEISNNCLSKDQLAEVVES 906
chicken-Duox      ------------------------ENGFLSKEEFLRMLRYFSSAQQTFSQRLQLQVSAQA 874
frog-Duox1        ------------------------GNGILPKKEEFSRMLRSFRNVS-SFLSNEKTENVIES 868
frog-Duox2        ------------------------GNGFLSKEEFFTMLKSFIEISNNCISKDQTEQVIEI 899
tetraodon-Duox    --------------------------------------RSFLEISNCTLSKAQAEDGIRA 923
fugu-Duox         ------------------------GTGSLSKGEF-------------------------- 852
zebrafish-Duox    ------------------------GDGFLSKEEFTSLLRSFIDIS-GALSKSQADDGIAA 875
medaka-Duox       ------------------------GNGYLSKEEFARMLRSFIEISNGALSKTQAEDGIKA 906
Ci-Duox-B         ------------------------HSGEINREEGRNMIKSFLEMAGANLGPDEVSAAVST 883
Ci-Duox-A         ------------------------KSGELSKKEFKVMLKSMMDMVNASVDSEQVEELVNT 859
Ci-Duox-C         ------------------------KSGGLSKEEFTTMLKSMMEMVNSSADVDNIDSVVDD 796
Ci-Duox-D         ------------------------QNGSLTKQQFKEMCMCTADTYQSTVDQNVLENVLSK 812
Sp-Duox           ------------------------RSGHLSREEFRQMLKSMMEMVSASVEETDLDKLIHD 1026
fruit fly-Duox    ------------------------RNGVIDKGELSEMMRSLVEIARTTSLGDDQVTELID 880
mosquito-Duox     ------------------------RNGVIDKGELSEMMRSLVEIARTTSVTDEQVNELID 881
honeybee-Duox     ------------------------CNGVIDKEELSEMLRSLVEIARTTSLSDDHVTELID 885
Celeg-Duox2       ------------------------GKNKVLRKDLAELVKSLNQTAGVHITESVQLRLFND 912
Celeg-Duox1       ------------------------GKNKVLRKDLAELVKSLNQTAGVHITESVQLRLFNE 904

                       -----EF-IV----
                       X Y Z  -Y-X -Z
human-Nox5alpha   LFESADADGNG--AITFEELRDELQRFPGVMENLTI------------------------ 178
dog-Nox5          LFESADKDCNG--AITFDELRDELQRFPGVMENLTIR----------------------- 203
cow-Nox5          LFESADKDCSG--TITFEELRDELQRFPGVLENLTI------------------------ 206
chicken-Nox5      LLEAADQDGNG--SITFQELQQQLEAVPGLMESLTI------------------------ 169
frog-Nox5         LFESADKDHSG--SITFQELKEELERFPEVMENLTI------------------------ 194
opossum-Nox5      LFESADKDHSG--SITFEELQEELDKFPEVMENLTI------------------------ 185
fugu-Nox5         LFESADKDKSG--AITFEELKAELESFPEVMENLTI------------------------ 119
medaka-Nox5       LFESADTDNSG--SITFEELKEELENFPEVMENLTI------------------------ 188
Sp-Nox5A          LFEAADTDGSG--AISFEELKEELEKNPDVMENLTI------------------------ 129
Sp-Nox5B          LFDDADVDGDG--EVSFEELSEQLQRYPGITSNLTI------------------------ 112
fruit fly-Nox5    MFEDADPHNSG--EITYEALKNQLHKHGGLLENLSI------------------------ 227
mosquito-Nox5     MFEDADKYNRG--AITYEALKNQLEKHGGLLENLSI------------------------ 226
honeybee-Nox5     LFDDADQSNRG--AITFEALKKQLEKHEGLLENLSI------------------------ 225
Mg-NoxC           KQDRERHVFLR--SVIG-SDQDRIPRADFARVVKSWR----------------------- 187
Fg-NoxC           DDDRTRHAFLR--RMMGDSGVDQIPRDEFAKMVKEWK----------------------- 112
At-rbohF          IMEELDPERLG--YIELWQLETLLLQKDTYLNYSQAL----------------------- 350
At-rbohI          IMEELAPDGLYSQYIELKDLEILLLEKDISHSYSLPF----------------------- 343
At-rbohC          IMEELDPDNIG--YIMLESLETLLLQAATQSVITSTG----------------------- 312
At-rbohG          IMEELDPDHMG--YIMMESLKKLLLQAETKSVSTDINSE--------------------- 264
At-rbohA          IMEELDPYHYG--YIMIENLEILLLQAPMQDVRDGEG----------------------- 307
At-rbohD          IMEELDPDNAG--FIMIENLEMLLLQAPNQSVRMGDS----------------------- 339
At-rbohB          IMEELDRDNLG--YIELHNLETLLLQVPSQSNNSPSS-A--------------------- 259
```

```
At-rbohE            IMEELDPENFG--YIELWQLETLLLQRDAYMN---------------------------- 349
At-rbohH            IMEELDPDHKG--YIEMWQLEILLTGMVTNADTEKMK----------------------- 281
At-rbohJ            IMEELDPNEQG--YIEMWQLEVLLTGIVSNADSHKVVR---------------------- 292
Dd-NoxC             IFQQFDKNMDG--YIDFEEFKSELTINNENKVKEKEE----------------------- 548
mouse-Duox1         MFRESGF-QDKEELTWEDFHFML-RDHDSDLRFTQLCVKGVEVPEVIKNLCRRASYISQE 960
mouse-Duox1         MFRESGF-QDKEELTWEDFHFML-RDHDSDLRFTQLCVKG-------------------- 944
rat-Duox1           MFWESGF-QDKEELTWEDFHFML-RDHDSDLRFTQLCVKGVEVPEVIKNLCRRASYISQE 956
dog-Duox1           MFRESGF-QDKEELTWEDFHFML-RDHDSELRFTQLCVRGVEVPEVIKDLCRRASYISQE 960
human-Duox1         MFRESGF-QDKEELTWEDFHFML-RDHNSELRFTQLCVKGVEVPEVIKDLCRRASYISQD 960
dog-Duox2           MFRESGF-QDKEELTWEDFHFML-RDHDSELRRTQLCVRGVG------------DIFKPN 711
human-Duox2         MFRESGF-QDKEELTWEDFHFML-RDHDSELRFTQLCVKGGGGGGNGI-----RDIFKQN 959
rat-Duox2           MFRESGF-QDKEELTWEDFHFML-RDHDSDLRFTQLCVKGGAGGTG--------DIFKQS 956
chicken-Duox        AHCFSSS-VPK-EATWKPMGSTL-TTR--------------------------------- 898
frog-Duox1          MFNEAGI-SNKKELAWEDFYGLF-KDHKNILNQTNLYFDGIHVIN------------QSY 914
frog-Duox1          MFRDSGF-QNKEELTLEDFHYLL-RDHDKELRFSQICIAGMDAPEVFNQMCRRVSFVDTT 957
tetraodon-Duox      MMQATGF-DRKEKITWKDFHFLL-QDHEKELQFAQLNVKGMEKRGQRRLSRDQRVSFICP 981
fugu-Duox           ---------ARMLRFDPHFLFIL-APVPITRPILPFCRSFIEISN-----------CTLS 891
zebrafish-Duox      MLQTAGL-YNKDRFSWEDFHFLL-RDHSAQLNIKGMEVLGK---------------KKLG 918
medaka-Duox         MMQAAGF-NNKEQISWEDFHFLL-ADHDKELQFAKLNVKGQTCIVKEVLPLWMEKEGRKR 964
Ci-Duox-B           IFKEAGIGENKDSLTLEDFTYVLLKDHREAFESSELSMPGRSNLYKDQGELSEQNKMSDQ 943
Ci-Duox-A           MLATHGY-EHKESLTLEDFQTVM-QKYSTELSDASLTVPAVKSFYSFCLRYFFIYFCTNE 917
Ci-Duox-C           MMRANGF-SSKDSLDLNDFLMLL-GQYS-DISNQQSTSNGHK-----------TYQAKRQ 842
Ci-Duox-D           VVPLKND-EEIQYLKFDDFKKLM-DLPEMDEAMLNLTAPVIG-------------KNKKS 857
Sp-Duox             MFQNAGL-GDKEALSLDDFIAVM-AEHKDELNNAKLDIAGNIPQIAGQKDAGPPGRGAAT 1084
fruit fly-Duox      GMFQDVGLEHKNHLTYQDFKLMM-KEYKGDFVAIGLDCKGA------------------- 920
mosquito-Duox       GMFQDVGLEHKNHLTYEDFKLMM-KEYKGDFVAIGLDCKGA------------------- 921
honeybee-Duox       GMFQDAGLERKDYLTYNDFKLMM-KEYKGDFVAIGLDCKGA------------------- 925
Celeg-Duox2         VLHKSGVSDDAEYLTCNNFDALF-SEISDVQPIGLPFN--------------------- 949
Celeg-Duox1         VLHYAGVSNDAKYLTYDDFNALF-SDIPDKQPVGLPFN--------------------- 941
```

```
human-Nox5alpha     ------------------------------------------------SAAHWLTAPAPR 190
dog-Nox5            ----------------------------------------------CGSASRVGTARVQV 217
cow-Nox5            ------------------------------------------------SAAHWLTPPAPQ 218
chicken-Nox5        ------------------------------------------------SAASWLKPPAPT 181
frog-Nox5           ------------------------------------------------SAANWLKPPAVQ 206
opossum-Nox5        ------------------------------------------------SAANWLKPPSSK 197
fugu-Nox5           ------------------------------------------------SAANWLKPPDLE 131
medaka-Nox5         ------------------------------------------------SAANWLKPPDLD 201
Sp-Nox5A            ------------------------------------------------SAASWLKPPSLK 141
Sp-Nox5B            ------------------------------------------------------------ 112
fruit fly-Nox5      ------------------------------------------------TIDRWLVPIAED 239
mosquito-Nox5       ------------------------------------------------SIDRWLVPLPQE 238
honeybee-Nox5       ------------------------------------------------SIDRWLVPPKPE 237
Mg-NoxC             ----------------------------------------------VPSMKQEGDKKDDQ 201
Fg-NoxC             ----------------------------------------------IPSLKQAKKEEEED 126
At-rbohF            -----------------------------------------------SYTSQALSQNLQG 363
At-rbohI            -----------------------------------------------SQTSRALSQNLK- 355
At-rbohC            ---------------------------------------------ERKNLSHMMSQRLKP 327
At-rbohG            ---------------------------------------------ERKELSDMLTESLKP 279
At-rbohA            -----------------------------------------------KKLSKMLSQNLMV 320
At-rbohD            -----------------------------------------------RILSQMLSQKLRP 352
```

```
At-rbohB             ---------------------------------------------NKRALNKMLSQKLIP 273
At-rbohE             -------------------------------------------------YSRPLSTTSGG 360
At-rbohH             -------------------------------------------------KSQTLTRAMIP 292
At-rbohJ             -------------------------------------------------KSQQLTRAMIP 303
Dd-NoxC              ------------------------------------------------NTNYNFKEENIG 560
mouse-Duox1          KICPSPRMSAHCARNN----MKTASSPQRLQCPMDTDPPQEIRRRFGKKVTSFQPL--LF 1014
mouse-Duox2          --------GAGGTKDIFK--FKQSSACR----------VSFINRTPGNRVMGPSPR--LY 980
rat-Duox1            KICPSPRMSAHCARNN----TKTASSPQRLQCPVDTDPPQEIRRRFGKKVTSFQPL--LF 1010
dog-Duox1            KICPSPRVSARCPHSN----TEVEWTPQRLQCPVDTDPPQEIRRRFGKKVTSFQPL--LF 1014
human-Duox1          MICPSPRVSARCSRSD----IETELTPQRLQCPMDTDPPQEIRRRFGKKVTSFQPL--LF 1014
dog-Duox2            ISCRVSFITRTPGKRS----CSQDLEFSASEAPELGGP--GLKKRFGKKVVGPTPR--LY 763
human-Duox2          ISCRVSFITRTPGERS----HPQGLGPPAPEAPELGGP--GLKKRFGKKAAVPTPR--LY 1011
rat-Duox2            NACRVSFLTRTPGNR----------------------------------VMAPSPR--LY 980
chicken-Duox         ----SPFLLAN----------------------------------------QYQLH--LY 912
frog-Duox1           VLCIICSIDSNIYTNI----SSALCFILFICFIALLLIKKHLCHPNRNTSQIQLPN--IY 968
frog-Duox2           NMCQSPNPSSLTGRIRKKVRNTTFLMHINFFRTNFPHLFDLEKQTRKLINKPKTSS--EA 1015
tetraodon-Duox       ASRWGHWFSI----------IFNCNDHFNTSCSMSAASARRKYTRFADRKSQFLPN--VF 1029
fugu-Duox            KKQAEDAIG-----------AMMTAAGFDHKEKITWEDFHFLLRDHEKELHVRRPN--VY 938
zebrafish-Duox       RQHKVSFIRK----------NSSSSSVEELTHTPEEEHGQELRQRQTKKAGQSQAK--LY 966
medaka-Duox          LSQCQRVSFICPKKSVICFWLCLTVCSNKD-------KAEGLELRRRKNTSIKTPN--VY 1015
Ci-Duox-B            RRVDKPYIIKDNNQ------LLSTYSTYNLSDDVICIPNNNIRQNITVQNFFFRSNGKVF 997
Ci-Duox-A            SFLHVRLLRAITQNFL---------VFIKLSLLIKNYEPKIYRKNPEKKNRTPETS-RIQ 967
Ci-Duox-C            SKYHRYSKRFKEG-------------YVRLHCIKSMFTKLYITHRPQTPQRRLTR----K 885
Ci-Duox-D            LAKSSKNERPNRGNFI---------QTAEGQGIVRNNHPQIIAPPCEDKSPNVST--VEN 906
Sp-Duox              VIRRGNLNSRARQTII-------RAYKDRDQASTKANGAAKGAGGSGGGVRKRAQSKSVR 1137
fruit fly-Duox       ------------------------------------------KQNFLDTSTNVARMTSFN 938
mosquito-Duox        ------------------------------------------KQNFLDTSTNVARMTSFH 939
honeybee-Duox        ------------------------------------------KQNFLDTSTNVARMTSFH 943
Celeg-Duox2          ------------------------------------------RKNYNSHIKEPSCHTSFP 967
Celeg-Duox1          ------------------------------------------RKNYQPSIGETSSLNSFA 959


                                                   ---TM1-(TM2 for Duox)--
human-Nox5alpha      PR----------PRRPRQLTRAYWHNHRSQLFCLATYAGLHVLLFGLAASAHRDL----- 235
dog-Nox5             PL----------QLRRLALTWAYWHNHRSHLLCLAAFAGLHLLLFALAASEHRAR----- 262
cow-Nox5             RH----------RRQPRLLTSAYWHNHRSHVLCLAVFVGLHMLLFALAASAYRAF----- 263
chicken-Nox5         RH----------SRRPRCPTSRSWHNHRGQLAFLGGYVSLNLLLFTLAALRHFGG----- 226
frog-Nox5            NK----------SHTPRYLTRTYWHNNRSKLLFMCCYWCLNVLLFGLAAVNHASL----- 251
opossum-Nox5         KR----------PQIPRHLTSAYWHNNC183-AVLAVYIGLNILLFTLAALKYQS----- 241
fugu-Nox5            QKKQ--------NHTPRYLTRAYWHNNCRKLLFLCMYAFFSLMLFVNAMLQHSYG----- 178
medaka-Nox5          QNKH---------QTPRYLTRAYWQNNSRKLLFLFGYGVLNLLLFVVAMLRHSDG----- 247
Sp-Nox5A             PSR---------RVLPRYLTWRYVHNNYRKILFLVVFILINVALFTEAAYRYAKK----- 237
Sp-Nox5B             -------------------SYNTLRNHFSVIIFWIVFVMINAGLAAWGAYEGYQS----- 198
fruit fly-Nox5       RQAGGAAKSGFWNSLPHQFSLAYMKNNQVFVTYLFFYITVNLCLFISRAIQYRAS----- 294
mosquito-Nox5        DTKKKRKKK----PLPHQLTAPYIKNNYVYLSFLTVFTLINVGLFVSRAIQYRNS----- 289
honeybee-Nox5        SKRKSRLQLLAS-LRPYQLTKPYMKNNYVYIFFISIFILINVSLFVSRLYEYRKS----- 291
Mg-NoxC              DKYLR-------DMSTFRRVRAYWAVHGPEIAFLGIVVGLQLGLGIWQCHKYASGEQ--- 251
Fg-NoxC              KSYIK-------RLPGWRRIRSYWAVHGPEIVFLGVVISMQLAFGIWQLVKYQTTPG--- 176
At-rbohF             LRGKSR------IHRMSSDFVYIMQENWKRIWVLSLWIMIMIGLFLWKFFQYKQKD---- 413
At-rbohI             ----DR------RWRMSRNLLYSLQLDNWKRIWVLTLWFVIMAWLFMWKCYQYKHKD---- 401
At-rbohC             TFNRNP------LKRWYRGLRFFLLDNWQRCWVIVLWFIVMAILFTYKYIQYRRSP---- 377
At-rbohG             TRDPNH------LRRWYCQLRFFVLDSWQRVWVIALWLTIMAILFAYKYIQYKNRA---- 329
At-rbohA             PQSRNL------GARFCRGMKYFLFDNWKRVWVMALWIGAMAGLFTWKFMEYRKRS---- 370
```

```
At-rbohD          AKESNP------LVRWSEKIKYFILDNWQRLWIMMLWLGICGGLFTYKFIQYKNKA---- 402
At-rbohB          TKDRNP------VKRFAMNISYFFLENWKRIWVLTLWISICITLFTWKFLQYKRKT---- 323
At-rbohE          ------------------------VNNWQRSWVLLVWVMLMAILFVWKFLEYREKA---- 392
At-rbohH          ERYRTP------MSKYVSVTAELMHENWKKLWVLALWAIINVYLFMWKYEEFMRNP---- 342
At-rbohJ          KRYRTP------TSKYVVVTAELMYEHWKKIWVVTLWLAVNVVLFMWKYEEFTTSP---- 353
Dd-NoxC           IYTEKE---------SFHSLKRYLKIEGSKLFFISLFFIINSILVITSFLNVHANNKR-- 1542
mouse-Duox1       TEAHREKFQRSRRHQTVQQFKRFIENYRRHIGCVAVFYTITGALFLERAYYYAFAAHHSG 1074
mouse-Duox2       TEALQEKKQSGFLAQKFKQYKRFVENYRRHIVCVTIFSAICIGLFADRAYYYGFASPPTD 1040
rat-Duox1         TEAHREKFQRSRRHQTVQQFKRFIENYRRHIGCVAVFYTITGALFLERAYYYAFAAHHSG 1070
dog-Duox1         TEAQREKFQRSRRHQTLQQFKRFIENYRRHIGCVAVFYAITGGLFLERAYYYAFGAHHMG 1074
human-Duox1       TEAHREKFQRSCLHQTVQQFKRFIENYRRHIGCVAVFYAIAGGLFLERAYYYAFAAHHTG 1074
dog-Duox2         TEALKEKMQRGLLAQKLRQYKRFVENYRRHIVCVVVFSAICAGLFAERAYYYAFASPPSG 823
human-Duox2       TEALQEKMQRGFLAQKLQQYKRFVENYRRHIVCVAIFSAICVGVFADRAYYYGFASPPSD 1071
rat-Duox2         TEALQEKMQRGFLAQKLKQFKRFVENYRRHIVCVTIFSAICAGLFADRAYYYGFASPPTD 1040
chicken-Duox      TEAQRKKYQQNKVKQKIQEFKRFIENYRRHIVCVVLFSAITAGLFVERAYYYAFASPSTG 972
frog-Duox1        TVARREKYETSTFRQKIQQFKRLVENYRRHIVCLIIFYGISAGLFAERAYYYGFASPSSG 1028
frog-Duox2        RLYKKEKYPKGIIHQKLQEFKRFAENYRRHIVCVVIFYGISVGLFLERAYHYAFESQHRG 1075
tetraodon-Duox    VDPKREQYIRSPIRQKIQQFKRFVENYRRHIVCFIVVYGITAGLCLERCYYYGFQAESTG 1089
fugu-Duox         VDPRRERYIRSPVRQKIQHFKRFVENYRRHIVCSALVYGMAAGLCLERCYYYGFQAESTG 998
zebrafish-Duox    VRPQRERFNRNPVQQCVQQFKRFIENYRRHIICTVVIYAISAGLALERCIYYGLQAHSSG 1026
medaka-Duox       VKAKRQEYIRNPVQQKIQQFKRFIENYRRHIVCFIITYSITVGVTLERCYYYGLQAEATG 1075
Ci-Duox-B         SDATVSHEEKTWFARTWKYLKRNVENYRRHIFCMVIFYGITIALVVERATFYAFGAEHIG 1057
Ci-Duox-A         IKVYEKVFKPGRLNNWLRAMENYVENHRLQIFWLTLYLLVLAGIFIERAYFYSVEREFAG 1027
Ci-Duox-C         VKTVREDYKTKSYEKFFVALVKLTEHYANHIFCLSLYSLITAGVFLN-AFFVVYSKNATG 944
Ci-Duox-D         VMPDVYFQNSSKFRLAYRAIRRLFECYALHIFWTSLYIWITIGVFLW-AFSMTIYPSFIF 965
Sp-Duox           VETVQQEEAKTQSSKAYNTVVRFFENNRLQIFYVVLYLLVLAGVFIERAYYYSVEREFAG 1168
fruit fly-Duox    IEPMQ-DKPRHWLLAKWDAYITFLEENRQNIFYLFLFYVVTIVLFVERFIHYSFMAEHTD 997
mosquito-Duox     IEPIS-DSRRHWMQEKWDCYTTFLEENRQNIFYLFLFYVITIVLFVERFIHYSFMAEHTD 998
honeybee-Duox     IDQLPPEDSKTWAQKQWDAISTFLEENRQNIFYLFVFYVTTIALFVERFIYYSFMAEHTD 1003
Celeg-Duox2       IVDHST--PAPLSLIQR--ICAFLETYRQHVFIIFCFVAINIVLFFELFWHSRYLNEDRD 1023
Celeg-Duox1       VVDRSINSSAPLTLIHK--VSAFLETYRQHVFIVFCFVAINLVLFFERFWHYRYMAENRD 1017
```
