# Supplementary material for: Molecular evolution of the reactive oxygen-generating NADPH oxidase (Nox/Duox) family of enzymes
Source: BMC Evol Biol. 2007 Jul 6;7:109. doi: 10.1186/1471-2148-7-109 (PMC1940245; doi:10.1186/1471-2148-7-109)
Supplement: Additional File 9 — Amino acid sequences of ferric reductase (FRE) proteins of fungi and alignment of FRE protein and human Nox2 protein sequences. Amino acid sequences of FRE of A. nidulans, Saccharomyces cerevisiae, Schizosaccharomyces pombe and alignment of FRE and human Nox2 proteins are provided. [file 1471-2148-7-109-S9.pdf]

Additional file 9

Amino acid sequences of ferric reductase (FRE) proteins of fungi and alignment of FRE protein and human Nox2 protein sequences

Sequences were obtained from the GenBank^{TM} databases (http://www.ncbi.nlm.nih.gov/). To describe species of the genes, we used the following abbreviations: An, *A. nidulans*; Scerevisiae, *Saccharomyces cerevisiae*; Spombe, *Schizosaccharomyces pombe*. *Gray boxes* in alignment indicate the Nox/Duox-conserved amino acid residues shown in Figure 6.

>Scerevisiae-FRE1: GenBank^{TM} No. NP_013315
MVRTRVLFCLFISFFATVQSSATLISTSCISQAALYQFGCSSKSKSCYCKNINWLGSVTACAYENSKSNKTLDS
ALMKLASQCSSIKVYTLEDMKNIYLNASNYLRAPEKSDKKTVVSQPLMANETAYHYYYEENYGIHLNLMR
SQWCAWGLVFFWVAVLTAATILNILKRVFGKNIMANSVKKSLIYPSVYKDYNERTFYLWKRLPFNFTTRGK
GLVVLIFVILTILSLSFGHNIKLPHPYDRPRWRRSMAFVSRRADLMAIALFPVVYLFGIRNNPFIPITGLSFSTFN
FYHKWSAYVCFMLAVVHSIVMTASGVKRGVFQSLVRKFYFRWGIVATILMSIIIFQSEKVFRNRGYEIFLLIH
KAMNIMFIIAMYYHCHTLGWMGWIWSMAGILCFDRFCRIVRIIMNGGLKTATLSTTDDSNVIKISVKKPKFF
KYQVGAFAYMYFLSPKSAWFYSFQSHPFTVLSERHRDPNNPDQLTMYVKANKGITRVLLSKVLSAPNHTVD
CKIFLEGPYGVTVPHIAKLKRNLVGVAAGLGVAAIYPHFVECLRLPSTDQLQHKFYWIVNDLSHLKWFENEL
QWLKEKSCEVSVIYTGSSVEDTNSDESTKGFDDKEESEITVECLNKRPDLKELVRSEIKLSELENNNITFYSCG
PATFNDDFRNAVVQGIDSSLKIDVELEEESFTW
>Spombe-FRE1: GenBank^{TM} No. NP_595271
MILARDDKWTLGSIALIFVLLIGFALLFLLERFRVKEKSRTFKDCVNVYQCPSKGERVYLALRHWFIFLATHK
AQMTLILSPLVMLVTIPFTGKETKNSIASYDWNLTGVAARLGYLSCGLFFVSYFFSLKNNPFCLMLFSSHEK
MNYLHRWLSVYAVLISVLHGILFMIFSAQSYKPLLYDKISIYGYFITVVLFLMTVASLPSVRRKFFEWFFVLH
HTCSVLIIFLIWLHHPRTIVYMKACIIIYAFDRGCRLFRSIWNRSNFRIYLLNEDMIYMVGRKPKRSFFALPWA
AGSHVYINIPSLSYWQVHPFTLASAPFDDFIELFVAVHSGFTERLANRLYSMPHEYPNFSLAPGTPESLSNTYR
ELNSFKSYAVEIENTAQGHTYEPEDLYLETTVFMDGPYGTTSNVFKEYSYVLLIAGGVGFSYTLPILRDLILK
ECNVTSITFIWSCRSLSLLKVASKSLNSLLHQSNVRLKIINHFTGSISCKESSEFSNQTTENSEMEFFDDRPDLD
MYIQKFFDYVGYQTAALAACGSQSFLKRIKNSVNKSISSTTDIYQHYEEL
>Spombe-FRE2: GenBank^{TM} No. NP_595065
MAINSSDKWTVIAICLILGILLAFILMFWLERFRVIIKSNAHKHDPSDKRQIWLEKYYLFVRQIYTYLVTHKVI
LTLIAVPVVFAISIPFIGMQTPASSHGKQTTQVSTGNWSKNAVAARLGFLACGLYVTSYFFSIKNNPFALLLIS
SHEKMNYVHRRLSQYAIMIGAIHGFAYIGLAAQGKRALLTARVTIIGYVILGLMVIMIVSSLPFFRRRFYEWF
FVLHHMCSIGFLITIWLHHRRCVVYMKVCVAVYVFDRGCRMLRSFLNRSKFDVVLVEDDLIYMKGPRPKKS
FFGLPWGAGNHMYINIPSLSYWQIHPFTIASVPSDDFIELFVAVRAGFTKRLAKKVSSKSLSDVSDINISDEKIE
KNGDVGIEVMERHSLSQEDLVFESSAAKVSVLMDGPYGPVSNPYKDYSYLFLFAGGVGVSYILPIILDTIKKQ

SRTVHITFVWSARSSALLNIVHKSLCEAVRYTEMNINIFCHLTNSYPVEEVSSLNSQSARNYSLQYLNGRPDV
NDYFKDFLHATGTQTAALASCGSDKLLRHLKSCVNTHSPSTVDLYQHYEEI

>An-FRE: GenBank™ No. AAN61438

MGVDLLPPGQERQRHVHPPMNIALATPLFALAGGFLILFVGRLAIRMRHHLRLRAVLRNDDQTPFTNRNGF
MAWTNRHVFYAPLFGARHSRELRIGRAHMGTVPLRIETLILALYIAINFAFFVCLVDWWEDYQEKLYQVKY
AGGHLAVMNTPGLVLAAARNNPLIPLLGISFDTFNLFHRWVGRVIVVGAIIHMSAVIAGLIAEHGFETTTHII
WEVPFFIWGMIALFGFILIAIQSVSLLRHAFYEVFLHLHVALAVMSFVGLWYHLRGLEQQNVVLGTIILWGL
ERVTRVASLVWRNVGKQRTVADFELLPGNVIRATVTLARTGEFRAGQHMYLYVPSVGLWTSHPFSVAWTS
TEEVSVDSDSNESFKMLLDRKPQTTISFLIKREDGFTRELQRKAANSDTCQFTTTVFAEGPYGGLEDLNSYGT
VLLIASGVGITNTMSYLYQFLEGFSARKTAVRRVNLVWVTRSVEDLHWIDPWMKSVFTHPAIATKESFQNN
RLAVSVQVYVTRKEASEASVGDSENLWAFSAPSGVSVSVGFGRPGFAVVIEREMETQVGAMAVSVCGNGC
VTDDVRQAVREAQKGAKTISLHDEAFCW

```
Spombe-FRE1        --------------------MILARDDKWTLGSIALIFVLLIGFALLFLLERFRVKEKSR 40
Spombe-FRE2        --------------------MAINSSDKWTVIAICLILGILLAFILMFWLERFRVIIKSN 40
Scerevisiae-FRE1   ------------------------------------------------------------
Aspergillus-FRE    MGVDLLPPGQERQRHVHPPMNIALATPLFALAGGFLILFVGRLAIRMRHHLRLRAVLRND 60
human-Nox2         -------------------------MGNWAVNEGLSIFVILVWLGLNVFLFVWYYRVYDI 35

Spombe-FRE1        TFKDCVNVYQCPSKGERVYLALRHWFIFLATHK--AQMTLILSPLVMLVTIPFTGKETKN 98
Spombe-FRE2        AHKHDP-SDKRQIWLEKYYLFVRQIYTYLVTHK--VILTLIAVPVVFAISIPFIGMQTPA 97
Scerevisiae-FRE1   ------------------YLWKRLPFNFTTRGK--GLVVLIFVILTILSLSFGHNIKLPH 40
Aspergillus-FRE    DQTPFTNRNGFMAWTNRHVFYAPLFGARHSRELRIGRAHMGTVPLRIETLILALYIAINF 120
human-Nox2         P--------------PKFFYTRKLLGSALALARAPAACLNFNCMLILLPVCRNLLSFLRG 81

Spombe-FRE1        S--------IASYDWNLTGVAARLGYLSCGLFFVSYFFSLKNNPFCLMLFSSHEKMNYLH 150
Spombe-FRE2        SSHGKQTTQVSTGNWSKNAVAARLGFLACGLYVTSYFFSIKNNPFALLLISSHEKMNYVH 157
Scerevisiae-FRE1   PYD------RPRWRRSMAFVSRRADLMAIALFPVVYLFGIRNNPFIPITGLSFSTFNFYH 94
Aspergillus-FRE    AFFVCLVDWWEDYQEKLYQVKYAGGHLAVMNTPGLVLAAARNNPLIPLLGISFDTFNLFH 180
human-Nox2         SSACCSTRVRRQLDRNLTFHKMVAWMIALHSAIHTIAHLFNVEWCVNARVNNSDPYSVAL 141

Spombe-FRE1        RWLSVYAVLISVLHGILFMIFSAQS------YKPLLYDKISIYGYFITVVLFLMTVASLP 204
Spombe-FRE2        RRLSQYAIMIGAIHGFAYIGLAAQG------KRALLTARVTIIGYVILGLMVIMIVSSLP 211
Scerevisiae-FRE1   KWSAYVCFMLAVVHSIVMTASGVKRG----VFQSLVRKFYFRWGIVATILMSIIIFQSEK 150
Aspergillus-FRE    RWVGRVIVVGAIIHMSAVIAGLIAEHGFETTTHIIWEVPFFIWGMIALFGFILIAIQSVS 240
human-Nox2         SELGDRQNESYLNFARKRIKNPEGG------LYLAVTLLAGITGVVITLCLILIITSSTK 195

Spombe-FRE1        SVRRKFFEWFFVLHHTCSVLIIFLIWLHHPRT--IVYMKACIIIYAFDRGCRLFRSIWNR 262
Spombe-FRE2        FFRRRFYEWFFVLHHMCSIGFLITIWLHHRRC--VVYMKVCVAVYVFDRGCRMLRSFLNR 269
Scerevisiae-FRE1   VFRNRGYEIFLLIHKAMNIMFIIAMYYHCHTLGWMGWIWSMAGILCFDRFCRIVRIIMNG 210
Aspergillus-FRE    LLRHAFYEVFLHLHVALAVMSFVGLWYHLRGLEQQNVVLGTIILWGLERVTRVASLVWRN 300
human-Nox2         TIRRSYFEVFWYTHHLFVIFFIG---LAIHGAERIVRGQTAESLAVHNITVCEQKISEWG 252

Spombe-FRE1        -SNFRIYLLNEDMIYMVGRKPKRSFFALPWAAGSHVYINIPS---LSYWQVHPFTLASAP 318
Spombe-FRE2        -SKFDVVLVEDDLIYMKGPRPKKSFFGLPWGAGNHMYINIPS---LSYWQIHPFTIASVP 325
Scerevisiae-FRE1   GLKTATLSTTDDSNVIKISVKKPKFFKYQVGAFAYMYFLSPKSAWFYSFQSHPFTVLSER 270
Aspergillus-FRE    VGKQRTVADFELLPGNVIRATVTLARTGEFRAGQHMYLYVPS---VGLWTSHPFSVAWTS 357
human-Nox2         KIKECPIPQFAGNPPMTWKWIVGPMFLYLCERLVRFWRSQQK-VVITKVVTHPFKTIELQ 311

Spombe-FRE1        FDDFIELFVAVHSGFTERLANRLYSMPHEYPNFSLAPGTPESLSNTYRELNSFKSYAVEI 378
Spombe-FRE2        SDDFIELFVAVRAGFTKRLAKKVSSK-------SLSDVSDINISDEKIEKNGDVGIEVME 378
Scerevisiae-FRE1   HR---------------------------------DPNNPDQLTMYVKANKG-------I 290
```

```
Aspergillus-FRE      TEEVSVDSDSNES-----------------FKMLLDRKPQTTISFLIKREDG-------F 393
human-Nox2           MKKKGFKMEVGQYIFVKCPKVSKLEWHP----FTLTSAPEEDFFSIHIRIVG--DWTEGL 365

Spombe-FRE1          ENTAQGHTYEPEDLYLETTVFMDGPYGTTS-NVFKEYSYVLLIAGGVGFSYTLPILRDLI 437
Spombe-FRE2          RHSLSQEDLVFESSAAKVSVLMDGPYGPVS-NPYKDYSYLFLFAGGVGVSYILPIILDTI 437
Scerevisiae-FRE1     TRVLLSKVLSAPNHTVDCKIFLEGPYGVTVPHIAKLKRNLVGVAAGLGVAAIYPHFVECL 350
Aspergillus-FRE      TRELQRKAANSDTCQFTTTVFAEGPYGGLE--DLNSYGTVLLIASGVGITNTMSYLYQFL 451
human-Nox2           FNACGCDKQEFQDAWKLPKIAVDGPFGTAS-EDVFSYEVVMLVGAGIGVTPFASILKSVW 424

Spombe-FRE1          LK------ECNVTSITFIWSCRSLSLLKVASKSLNSLLHQ---------SNVRLKIIN-- 480
Spombe-FRE2          KK------QSRTVHITFVWSARSSALLNIVHKSLCEAVRY---------TEMNINIFC-- 480
Scerevisiae-FRE1     RLP-----STDQLQHKFYWIVNDLSHLKWFENELQWLKEK---------SCEVSVIYTGS 396
Aspergillus-FRE      EGFS--ARKTAVRRVNLVWVTRSVEDLHWIDPWMKSVFTHPAIATKESFQNNRLAVSVQV 509
human-Nox2           YKYCNNATNLKLKKIYFYWLCRDTHAFEWFADLLQLLESQMQE------RNNAGFLSYNI 478

Spombe-FRE1          HFTGSISCKESSEFSN-----QTTENSEMEFFDDRPDLDMYIQKFFDYVGYQT--AALAA 533
Spombe-FRE2          HLTNSYPVEEVSSLNS-----QSARNYSLQYLNGRPDVNDYFKDFLHATGTQT--AALAS 533
Scerevisiae-FRE1     SVEDTNSDESTKGFDD-----KEESEITVECLNKRPDLKELVRSEIKLSELENNNITFYS 451
Aspergillus-FRE      YVTRKEASEASVGDSENLWAFSAPSGVSVSVGFGRPGFAVVIEREMET---QVGAMAVSV 566
human-Nox2           YLTGWDESQANHFAVHHDEEKDVITGLKQKTLYGRPNWDNEFKTIASQHPNTR--IGVFL 536

Spombe-FRE1          CGSQSFLKRIKN-SVNKSISSTTDIYQHYEEL---- 564
Spombe-FRE2          CGSDKLLRHLKS-CVNTHSPSTVDLYQHYEEI---- 564
Scerevisiae-FRE1     CGPATFNDDFRN-AVVQGIDSSLKIDVELEEESFTW 486
Aspergillus-FRE      CGNGCVTDDVRQ-AVREAQKGAKTISLHDEAFCW-- 599
human-Nox2           CGPEALAETLSKQSISNSESGPRGVHFIFNKENF-- 570
```
