# Supplementary material for: Molecular evolution of the reactive oxygen-generating NADPH oxidase (Nox/Duox) family of enzymes
Source: BMC Evol Biol. 2007 Jul 6;7:109. doi: 10.1186/1471-2148-7-109 (PMC1940245; doi:10.1186/1471-2148-7-109)
Supplement: Additional File 12 — Additional methods information. Methods information about gene identification, estimates of substitution rates, generation of point mutations, and Western blot analysis are provided. [file 1471-2148-7-109-S12.pdf]

Additional file 12

Additional methods information

Gene identification

We performed the UCSC genomic BLAT search [1] to look for possible orthologs of Nox5 of *R. norvegicus*, *M. musculus*, *B. taurus*, *M. domestica*, *D. rerio,* and Nox3 of *X. tropicalis*, *D. rerio*, *T. rubripes*, *T. nigroviridis*. Likewise, we utilized the NCBI Eukaryotic Genome Database [2] to search for Nox5 orthologs of *D. novemcinctus* and *O. cuniculus*. We also performed a BLASTP search in the yeast protein database to identify orthologs in *S. pombe* and *S. cerevisiae* [3] and in the NCBI Microbial Genome database [4] for homologues in prokaryotes, *Escherichia coli* K12 and *Staphylococcus aureus* RF122.

Estimates of substitution rates

Based on alignment of amino acid sequences of Nox domains (see Additional file 5) and common regions of each regulatory subunit (see Additional file 7), we counted the number of identical amino acid residues of the following species: *H. sapiens, C. familliaris*, *R. norvegicus*, *M. musculus*, *B. taurus* (Nox5 ortholog only), *G. gallus*, *X. tropicalis*, *D. rerio*, *T. rubripes*, and *T. nigroviridis*. Compared species are shown in

Additional file 3 as species A (e.g., dog Nox1) and species B (e.g., human Nox1). Substitution rate per amino acid site was calculated by the following two equations: rate1 (R1) =(N_{total} - N_{homo})/(N_{total})/2 and rate2 (R2) = R1/divergence time (10^6 years)/1000, where N_{total} is the total amino acid number of one species shown as species B and N_{homo} is the total number of homologous amino acids between the two sequences shown as species A and B. Calculated R1 and R2 and estimated divergence time are shown in Additional file 3. Several NOXO1 proteins were partial sequences, where the minimum length was 281 amino acids (*X. tropicalis*). To accurately compare substitution rates, we used trimmed NOXO1 sequences corresponding to the same length of *X. tropicalis* NOXO1 (residues 30-273 in human NOXO1) and trimmed p47*phox* sequences (residues 32-267). Alignments of p22*phox*, p47*phox*, NOXO1, p67*phox*, and NOXA1 used to estimate R1 and R2 are shown in Additional file 7.

Generation of point mutations of human Nox2

Nox2 carrying a substitution of Arg-73 to Glu (R73E) was constructed by PCR amplification of human Nox2 cDNA (GenBank™ accession No. NP_000397) using primer set A (N2-primer 1, TTTT*GGATCC*ACCATGGGGAACTGGGCTGTGAATGAGGGGCTC; R73E-primer 2, CTGAGGAAGGACAGCAGATTTTCACAGACTGGCAAGAGAATCAG) and

primer set B (R73E-primer 3, CTGATTCTCTTGCCAGTCTGTGAAAATCTGCTGTCCTTCCTCAG; N2-primer 4, TTTTGCGGCCGCTTAGAAGTTTTCCTTGTTGAA). Primers 2 and 3 contain nucleotides that correspond to the mutated amino acid. Primers 1 and 4 contain BamH1 (italics) and NotI (underlined) sites, respectively. After performing a second PCR with primers 1 and 4, the amplified nucleotide fragment was digested by each enzyme and then was subcloned into pcDNA3 (Invitrogen, Carlsbad, CA). Using a similar strategy, nucleotide fragments coding for mutations R80E, L94A, D95R, K314E, G322E, G389E, P390A, and G392E were amplified and subcloned into pcDNA3. Point mutation E568K was generated by primer set N2-primer 1 and E568K-primer 4 (TTTTGCGGCCGCTTAGAAGTTTTTCTTGTTGAAAATGAAATGCACTCCCCGA). E568K-primer 4 contains a NotI site (underlined), thus the fragment was digested with enzymes BamH1 and Not1 and subcloned into pcDNA3. Using a similar strategy, F570A was generated by specific F570A-primer 4 (TTTTGCGGCCGCTTATGCGTTTTCCTTGTTGAAAATGAAATGCACTCCCCGA), which contains a NotI site (underlined).

Measurement of ROS from Nox2-transfected cells

Forty-eight hours after transfection, cells were washed twice with Hanks' balanced salt solution (HBSS), harvested by centrifugation at 500 x *g* for 5 minutes, and resuspended in HBSS. Cells (1×10^5) were mixed with 200 μM luminol and 0.32 units of horseradish peroxidase in a 200 μL total volume in each well of a 96-well plate. To activate ROS production, cells were treated with control vehicle (DMSO) or 200 nM phorbol 12-myristate 13-acetate (PMA) before measurement. Luminescence was quantified using a FluoStar™ luminometer (BMG Labtech, Durham, NC), and peak values are reported.

Western blot analysis

Immunoprecipitated protein was resolved by 10% SDS-PAGE and transferred to polyvinylidene difluoride membrane. Following blocking with BSA (3%), proteins were probed using the indicated antibodies. Visualization was carried out with horseradish peroxidase-conjugated secondary antibodies against mouse IgG (Bio-Rad, Hercules, CA) and an enhanced chemiluminescent substrate kit (Pierce, Rockford, IL).

References

1. UCSC-genome-server: **http://genome.ucsc.edu/cgi-bin/hgBlat**.
2. NCBI-Eukaryote-Genome-Database: **http://www.ncbi.nlm.nih.gov/sutils/genom_table.cgi?organism=euk**.
3. NCBI-Fungi-Genomes-BLAST-server:

http://www.ncbi.nlm.nih.gov/sutils/genom_table.cgi?organism=fungi

4. NCBI-Microbial-Genome-Database: **http://www.ncbi.nlm.nih.gov/sutils/genom_table.cgi?organism=microb**.
