# Supplementary material for: Molecular evolution of the reactive oxygen-generating NADPH oxidase (Nox/Duox) family of enzymes
Source: BMC Evol Biol. 2007 Jul 6;7:109. doi: 10.1186/1471-2148-7-109 (PMC1940245; doi:10.1186/1471-2148-7-109)
Supplement: Additional File 5 — Alignment of Nox-domains of Nox and Duox proteins. Amino acid sequences of Nox and Duox orthologs were trimmed to the length corresponding to human Nox2 and aligned. Sixty-eight amino acid residues conserved among all Nox and Duox proteins (shown in Figure 6) are indicated by asterisks below the alignments. [file 1471-2148-7-109-S5.pdf]

Additional file 5

Alignment of Nox-domains of Nox and Duox proteins

Amino acid sequences were trimmed to the length corresponding to human Nox2 and aligned. The amino acid numbers were re-calculated to indicate those corresponding to the full-length sequences after alignment. Above the alignment, predicted domains of human Nox2, which contains six transmembrane domains TM(I) through TM(VI), two-FAD binding subregions FAD1 and FAD2, and four NADPH-binding subregions NADPH1 through NADPH4, are shown. To describe sequences of the different species, we used the following naming: Hs (as *H. sapiens*), Bt (*B. taurus*), Cf (*C. familliaris*), Rn (*R. norvegicus*), Mm (*M. musculus*), Gg (*G. gallus*), Md (*M. domestica*), Xt (*X. tropicalis*), Dr (*D. rerio*), Tr (*T. rubripes*), Tn (*T. nigroviridis*), medaka (*O. latipes*), Ci (*C. intestinalis*), Sp (*S. purpuratus*), Dm (*D. melanogaster*), Ag (*A. gambiae*), Am (*A. mellifera*), Ce (*C. elegans*), At (*A. thaliana*), Dd (*D. discoideum*), Pa (*P. anserina*), An (*A. nidulans*), Mg (*M. grisea*), Fg (*F. graminearum*), Cc (*C. crispus*) and Py (*P. yezoensis*). Amino acids conserved among all Nox and Duox proteins (shown in Figure 6) are indicated by *asterisks* below the alignments.

```
                                                     _________TM(I)_________
Hs-Nox1                ---------------------------MGNWVVNHWFSVLFLVVWLGLNVFLFVDAFLKY 33
Cf-Nox1                ---------------------------MGNWVVNHWFSVLFLATWLGLNVFLFVHAFLSY 33
Mm-Nox1                ---------------------------MGNWLVNHWLSVLFLVSWLGLNIFLFVYAFLNY 33
Rn-Nox1                ---------------------------MGNWLVNHWLSVLFLVSWLGLNIFLFVYVFLNY 33
Gg-Nox1                ---------------------------MGNWLVNHWFSAAVLAAWLGINIFLFTYYFLFF 33
Xt-Nox1                ---------------------------MGNWIANNWFSVVVLATWLGLNIFIFINFFMIF 33
Tn-Nox1                ---------------------------MGNWIVNHGLTSFILVVWMGINIFLFVWFYLFY 33
Tr-Nox1                ---------------------------MGNWIINHGLTAFILVVWMGINIFLFVWFYLFY 33
Ol-Nox1                ---------------------------MANWIINNGFPAVMVLVWMTINTFLFVWYYLQY 33
Dr-Nox1                ---------------------------MGNWIINHGLSAFIVVVWMAINIALFVHFYLFY 33
Mm-Nox2                ---------------------------MGNWAVNEGLSIFVILVWLGLNVFLFINYYKVY 33
Rn-Nox2                ---------------------------MGNWAVNEGLSIFVILVWLGLNVFLFVKYYKVY 33
Hs-Nox2                ---------------------------MGNWAVNEGLSIFVILVWLGLNVFLFVWYYRVY 33
Cf-Nox2                ---------------------------MGNWIENEGLSIFVILVWLGLNVFLFIWFYGVY 33
```

```
Gg-Nox2         ---------------------------MGNWVENEGLSIFVVLVWLGLNVFLFWWFYLAY 33
Xt-Nox2         ---------------------------MGNWIVNEGLSIAVIIVWLGLNGYLFWNFYLVY 33
Tn-Nox2         ---------------------------MGNFAANEGLSVFVILVWLGINAFLFVHFYMAF 32
Tr-Nox2         ---------------------------MGNFAANEGLSTFVILVWLGINAFLFVHFYMAF 32
Ol-Nox2         ---------------------------MGNFVANEGLSIFVILVWLGINAYLFVQFYMNF 32
Dr-Nox2         ---------------------------MGNFAANEGLSVFVILVWLGINVFLFVYFYLAF 32
Hs-Nox3         --------------------------MMGCWILNEGLSTILVLSWLGINFYLFIDTFYWY 32
Cf-Nox3         --------------------------MMGCWILNESLSVILVLSWLGVNLYLFIDTFCWY 32
Mm-Nox3         --------------------------MPVCWILNESGSFVVALLWLAVNAYLFIDTFFWY 32
Rn-Nox3         --------------------------MPTCWILNESVSFVVALLWLAINIYLFIDTFCWY 32
Gg-Nox3         ---------------------------MACWILNEKLSVLLLLVWLGLNLYLFIDTFHWY 33
Ci-Nox2         -----------------------MNARLNGFLVNEFPKYIVFLLWLGLNGFLFGYYYNFY 37
Sp-Nox2A        ---------------------------MGDKFLNEGLKYFFLLLWLAANVAYWVVTFLVY 33
Sp-Nox2B        ------------------------------------------AAWAIVNLIIWLVTFFKY 18
Mm-Nox4         -----------------------MAVSWRSWLANEGVKHLCLLIWLSLNVLLFWKTFLLY 37
Rn-Nox4         -----------------------MALSWRSWLANEGVKHLCLLVWLSLNVLLFWKTFLLY 37
Hs-Nox4         -----------------------MAVSWRSWLANEGVKHLCLFIWLSMNVLLFWKTFLLY 37
Cf-Nox4         -------------------MENAVMILCTEYYEHDEEYIDYRFIWLSLNVLLFWKAFLLY 41
Gg-Nox4         ------------------------------------------------------------
Xt-Nox4         -----------------------MALPCASWLSNEALKHLFLLSWLALNIGLFYKTFTVY 37
Ol-Nox4         -----------------------MAVSVRSWLANEAGKHFVLMLWLAANTWLFLDTYLLY 37
Tr-Nox4         -------------------------MSVRSWIANEGGKFLVLMLWLGVNTWMFLNTFLLF 35
Ci-Nox4         -------------------------MQLKNYLVNDGLRLFIWVAWISINASLFYFTFMYY 35
Ag-Nox          ------------------------------------------LLWTGFNFVVFCKAFSNY 18
Pa-NoxA         --------------------MGGLVPLLKKQLTGS--KILFHILFWTFHWGIFAYGWWKQ 37
Mg-NoxA         ---------------------MSVGEFLAKQLTAQ--KLFFNISFWGFHIGIFAYGWYKQ 36
Fg-NoxA         ------------------MGSQLGFVELIKKQFVPG-KLLYHFLFWTFHWGIFAYGWWKQ 40
An-NoxA         ----------------------MGRYPLKSYFAPS--KLFFYTWFWGAHIAIFAYGWYHQ 35
Pa-NoxB         RWTPLTRMLLSGEMTQERQKELTPREKFDKWMVNEGYRRIFVFVFMFLHAILFAFSFVNF 83
Fg-NoxB         RWTPLTRMLLSGEMTQEKQQELSSREKFDRWMINEGYRRFFVFVFMILHALIFSFACVHY 72
Mg-NoxB         RWTPLTRMLLSGEMTQERQKELTPREKFDRWMVNEGYRRFFVFVFMILHAMVFAFGFVNY 87
Dd-NoxA         --------------------MRLPTKEEIQRYWVNEGNKLILVILYTLGNIAAFVYTFVH 37
Dd-NoxB         --------KNEKIGLRSKIFKSKIFIKIRGWWWHRGISTYIMLFYIALNIGVGVHMFYNM 204
At-rbohC        ----------------------------RFFLLDNWQRCWVIVLWFIVMAILFTYKYIQY 373
At-rbohG        ----------------------------RFFVLDSWQRVWVIALWLTIMAILFAYKYIQY 325
At-rbohA        ----------------------------KYFLFDNWKRVWVMALWIGAMAGLFTWKFMEY 366
At-rbohB        ----------------------------SYFFLENWKRIWVLTLWISICITLFTWKFLQY 319
At-rbohD        ----------------------------KYFILDNWQRLWIMMLWLGICGGLFTYKFIQY 398
At-rbohF        ----------------------------VYIMQENWKRIWVLSLWIMIMIGLFLWKFFQY 409
At-rbohI        ----------------------------LYSLQDNWKRIWVLTLWFVIMAWLFMWKCYQY 397
At-rbohE        --------------------------------VNNWQRSWVLLVWVMLMAILFVWKFLEY 388
At-rbohH        ----------------------------AELMHENWKKLWVLALWAIINVYLFMWKYEEF 338
At-rbohJ        ----------------------------AELMYEHWKKIWVVTLWLAVNVVLFMWKYEEF 349
Hs-Nox5         --------------------------------WHNHRSQLFCLATYAGLHVLLFGLAASA 226
Cf-Nox5         --------------------------------WHNHRSHLLCLAAFAGLHLLLFALAASE 225
Bt-Nox5         --------------------------------WHNHRSHVLCLAVFVGLHMLLFALAASA 243
Md-Nox5         --------------------------------WHNNCGKLAVLAVYIGLNILLFTLAALK 215
Xt-Nox5         --------------------------------WHNNRSKLLFMCCYWCLNVLLFGLAAVN 224
Tr-Nox5         --------------------------------WHNNCRKLLFLCMYAFFSLMLFVNAMLQ 151
Tn-Nox5         ------------------------MLQHRSGAACYMLAKGCGQQCLNFNCTFVMVAARAP 35
Gg-Nox5         --------------------------------WHNHRGQLAFLGGYVSLNLLLFTLAALR 199
Ol-Nox5         --------------------------------WQNNSRKLLFLFGYGVLNLLLFVVAMLR 238
Dr-Nox5         --------------------------------WHNNSRKLFFLCLYGLLNTFLFIMAMLK 129
Sp-Nox5A        --------------------------------VHNNYRKILFLVVFILINVALFTEAAYR 230
Sp-Nox5B        --------------------------------RNHFSVIIFWIVFVMINAGLAAWGAYEG 179
```

```
Ag-Nox5      ----------------------------APYIKNNYVYLSFLTVFTLINVGLFVSRAIQ- 280
Am-Nox5      ----------------------------KPYMKNNYVYIFFISIFILINVSLFVSRLYE- 282
Dm-Nox5      ----------------------------LAYMKNNQVFVTYLFFYITVNLCLFISRAIQ- 285
Dd-NoxC      --------------------TEKESFHSLKRYLKIEGSKLFFISLFFIINSILVITSFLN 601
Mg-NoxC      -----------------DKYLRDMSTFRRVRAYWAVHGPEIAFLGIVVGLQLGLGIWQCH 243
Fg-NoxC      -----------------KSYIKRLPGWRRIRSYWAVHGPEIVFLGVVISMQLAFGIWQLV 163
Cc-NoxD      ----------MIPRSKPDVARPSARIEAYLSTHAFKVLFFAFYGAAVTLMFAWGFKAEFT 50
Py-NoxD      ----------MGDDKPPPPKSRVSRVESYLSTNGFVLTFLGLYILANVILFFFAATPERR 45
Xt-Duox1     ----------------------------------YRRHIVCLIIFYGISAGLFAERAYYY 20
Xt-Duox2     ----------------------------------YRRHIVCVVIFYGISVGLFLERAYHY 70
Gg-Duox      ----------------------------------YRRHIVCVVLFSAITAGLFVERAYYY 964
Tr-Duox      ----------------------------------YRRHIVCSALVYGMAAGLCLERCYYY 990
Tn-Duox      ----------------------------------YRRHIVCFIVVYGITAGLCLERCYYY 1081
Ol-Duox      ----------------------------------YRRHIVCFIITYSITVGVTLERCYYY 1067
Dr-Duox      ----------------------------------YRRHIICTVVIYAISAGLALERCIYY 1018
Mm-Duox2     ----------------------------------YRRHIVCVTIFSAICIGLFADRAYYY 1032
Rn-Duox2     ----------------------------------YRRHIVCVTIFSAICAGLFADRAYYY 1032
Hs-Duox2     ----------------------------------YRRHIVCVAIFSAICVGVFADRAYYY 1063
Cf-Duox2     ----------------------------------YRRHIVCVVVFSAICAGLFAERAYYY 815
Hs-Duox1     ----------------------------------YRRHIGCVAVFYAIAGGLFLERAYYY 1066
Cf-Duox1     ----------------------------------YRRHIGCVAVFYAITGGLFLERAYYY 1066
Rn-Duox1     ----------------------------------YRRHIGCVAVFYTITGALFLERAYYY 1062
Mm-Duox1     ----------------------------------YRRHIGCVAVFYTITGALFLERAYYY 1066
Ci-DuoxA     ----------------------------------HRLQIFWLTLYLLVLAGIFIERAYFY 1092
Ci-DuoxC     ----------------------------------YANHIFCLSLYSLITAGVFLN-AFFV 957
Ci-DuoxD     ----------------------------------YALHIFWTSLYIWITIGVFLW-AFSM 1035
Sp-Duox      ----------------------------------NRLQIFYVVLYLLVLAGVFIERAYYY 1189
Dm-Duox      ----------------------------------NRQNIFYLFLFYVVTIVLFVERFIHY 989
Ag-Duox      ----------------------------------NRQNIFYLFLFYVITIVLFVERFIHY 990
Am-Duox      ----------------------------------NRQNIFYLFVFYVTTIALFVERFIYY 995
Ci-DuoxB     ----------------------------------YRRHIFCMVIFYGITIALVVERATFY 1010
Ce-Duox1     ----------------------------------YRQHVFIVFCFVAINLVLFFERFWHY 1009
Ce-Duox2     ----------------------------------YRQHVFIIFCFVAINIVLFFELFWHS 1015


             _               _________TM(II)_________
Hs-Nox1      EKADKYYYTRKILGSTLACARASALCLNFNSTLILLP-VCRNLLSFLRG---TCSFCSRT 89
Cf-Nox1      EKADKYYYTREILGSTLAWARASARCLNFNSMLILLP-VCRNLLSFLRG---TCSFCRRT 89
Mm-Nox1      EKSDKYYYTREILGTALALARASALCLNFNSMMILIP-VCRNLLSFLRG---TCSFCNRT 89
Rn-Nox1      EKSDKYYYTREILGTALALARASALCLNFNSMVILIP-VCRNLLSFLRG---TCSFCNHT 89
Gg-Nox1      DRDERYFYTRAILGSALAWARASAKCLNFNSMLILLP-VCRNLLSFLRG---SFSCCRRT 89
Xt-Nox1      EKGDSYSYTRELLGSALAWARGSAACLNFNCLLILLP-VCRNLLSFLRG---TCTCVQRS 89
Tn-Nox1      DLGDQFFYTRHLLGSALAWARAPAAVLNFNCMLILLP-VCRNLLSLLRG---SFVCCGRS 89
Tr-Nox1      DLGERFFYTRHLLGSALAWARAPAAVLNFNCMLILLP-VCRNLLSLFRG---SFVCCGRS 89
Ol-Nox1      DRDDDFYYTRHLLGSALAWARAPAAVLNFNCMLILLP-VCRNLLSLIRG---SFVCCSRT 89
Dr-Nox1      DQGERFEYTRELLGSALAWARAPAAVLNFNCMLILLP-VCRNLLSLLRG---SFVCCGRT 89
Mm-Nox2      DDGPKYNYTRKLLGSALALARAPAACLNFNCMLILLP-VCRNLLSFLRG---SSACCSTR 89
Rn-Nox2      DDEPKYNYTRKLLGSALALARAPAACLNFNCMLILLP-VCRNLLSFLRG---SSACCSTR 89
Hs-Nox2      DIPPKFFYTRKLLGSALALARAPAACLNFNCMLILLP-VCRNLLSFLRG---SSACCSTR 89
Cf-Nox2      NNGEEFFYTRKLLGFALPLARAPAACLNFNCMLILLP-VCRNLLSFLRG---SSACCSTR 89
Gg-Nox2      DLPQNFFYTRVLLGRALALARAPAACLNFNCMLILLP-VCRNLLSFLRG---SSACCSTR 89
Xt-Nox2      DEGEKYFYSRKLYGSALAWARAPAACLNFNCLLILLP-VCRNLLSFLRG---SSACCGRS 89
Tn-Nox2      -LVERWFYTRVLLGHALSWARAPAACLNFNCMLILLP-VCRNLLSFLRG---SIQCCSRT 88
Tr-Nox2      -LVDRWFYTRVLLGHALSWARAPAACLNFNCMLILLP-VCRNLLSFLRG---SIQCCSRT 88
```

Ol-Nox2	-LVERWFYTRVLLGHALSWARAPAACLNFNCMLILLP-VCRNLLSFLRG---SIQCCSRT	88
Dr-Nox2	-LIDKYYYTRVILGHALSWARAPAACLNFNCMLILLP-VCRNLLSFLRG---SIQCCSRT	88
Hs-Nox3	EEEESFHYTRVILGSTLAWARASALCLNFNCMLILIP-VSRNLISFIRG---TSICCRGP	90
Cf-Nox3	EEEESFLYTRVILGSTLAWARASAVCLNFNCMLILLP-ISRNLISFMRG---TSTCCRGL	90
Mm-Nox3	TEEEAFFYTRVILGSALAWARASAVCLNFNCMLILLP-VSRNFISLVRG---TSVCCRGP	90
Rn-Nox3	AEEESFFYTRVILGSALAWARASAVCLNFNCMLILLP-VSRNFVSLVRG---TSVCCRGP	90
Gg-Nox3	EDEDAYVYTRIMLGSTLAWARASATCLNFNCMLILLP-VSRNLISFLRG---ASACCGGA	89
Ci-Nox2	NTKKTFFYTRVLLGPALALARAPAACLNLNCLLVLLP-VCRNLLSLFRK---ACMCCPRR	93
Sp-Nox2A	EQGPQYFYIRRITGVGLSIAKAAGAALNLNSMIILLP-ICRNLISFFRGSCATNTLCRRS	92
Sp-Nox2B	MDNANYIYTKYLMKNGLPVARASAACLNFNSMLILFP-VCRNMISYLRGSCESTKFSRRN	77
Mm-Nox4	NQGPEYYYIHQMLGLGLC--RASASVLNLNCSLILLP-MCRTVLAYLRGS--QKVPS-RR	93
Rn-Nox4	NQGPEYYYIHQMLGLGLCLSRASASVLNLNCSLILLP-MCRTVLAYLRGS--QKVPS-RR	93
Hs-Nox4	NQGPEYHYLHQMLGLGLCLSRASASVLNLNCSLILLP-MCRTLLAYLRGS--QKVPS-RR	93
Cf-Nox4	NQGPEYHYLHQMLGLGLCLSRASASVLNLNCSLILLP-MCRTLLAYLRGS--QKVPS-RR	97
Gg-Nox4	---PSYLTLPVFVQLGLCVSRASASVLNLNCCLVLLP-MCRILLAFLRGS--QKVAS-RK	53
Xt-Nox4	YSGPQYFYLHQMLGLGLCVSRASASVLNLNCSLVLLP-MCRTVIGLLRGP--KMVNIHWK	94
Ol-Nox4	STGQQYHYLYQMLGLGLCISRASASVLNLNCSLVLLP-MCRSLLTFIRGS--HTMST-RR	93
Tr-Nox4	SSGEQYYYLYKMLGLGLCISRASASVLNLNCSLVLLP-MCRSLLKFIRGT--HTVSS-RK	91
Ci-Nox4	YNGIQFYYLHQMLGYGLCISRASAACINLNSSFILFP-MCRGLVTFMRGL--PRGVG-RQ	91
Ag-Nox	HHDVEYYYLSRILGNGLCVSRGTAPVLNVTMALITLP-TCKTFNLLLHKL--FGRCSTRL	75
Pa-NoxA	AADARLAG-LNTLQYSVWLSRGAGLVLSVDGMLILLP-VCRTIMRFIR---------PKI	87
Mg-NoxA	FSDPRLAG-LNTLTFSVWISRGAGLVLSVDGMLILLP-VCRTIMRWIR---------PKI	86
Fg-NoxA	AVDPRLAG-LNTLKFSVWISRGAGLVLSVDCMLILLP-VCRTVMRWVR---------PKI	90
An-NoxA	AKSEPLSP-LNVLSYSVWISRGAGLVLTVDGTLILLP-MCRNLVRFLR---------PKL	85
Pa-NoxB	AVKENLQIARDTFGPTFMIARSAALVLHVDVALILFP-VCRTLISMARQ-------TPLN	135
Fg-NoxB	AQKESLETSRQTFGFTFIIARSAALVLHVDVAIILFP-VCRTLISLLRQ-------TPLN	124
Mg-NoxB	AVKDNLQRARDTFGPTFMIARAAALVLHFDVALILFP-VCRTFISLARQ-------TPLN	139
Dd-NoxA	YYNSP---AFEVVGYGVCFARGCAQLLKLNCALILVP-VLRNLLSFLRG-------TFLN	89
Dd-NoxB	YHSD----IFKFLGLSFCFSRTAARLINLNSAVILLP-VLRNFLSWLRG-------TIVN	256
At-rbohC	RRSPVYYPVMGDCVC----MAKGAAETVKLNMALILLP-VCRNTITWLRN-----KTRLG-	422
At-rbohG	KNRAVYEVLGPCVC----LAKGAAETLKLNMALILLP-VCRNTITWLRN-----KTRLG-	374
At-rbohA	RKRSAYEVMGVCVC----IAKGAAETLKLNMAMILLP-VCRNTITWLRT-----KTKLS-	415
At-rbohB	KRKTVFEVMGYCVT----VAKGSAETLKFNMALILLP-VCRNTITWLRT-----KSKLIG	369
At-rbohD	KNKAAYGVMGYCVC----VAKGGAETLKFNMALILLP-VCRNTITWLRN-----KTKLG-	447
At-rbohF	KQKDAFHVMGYCLL----TAKGAAETLKFNMALILFP-VCRNTITWLR------STRLS-	457
At-rbohI	KHKDAFHVMGYCLV----MAKGAAETLKFNMALILLP-VCRNTITYLR------STALS-	445
At-rbohE	REKAAFKVMGYCLT----TAKGAAETLKLNMALVLLP-VCRNTLTWLR------STRAR-	436
At-rbohH	MRNPLYNITGRCVC----AAKGAAETLKLNMALILVP-VCRKTLTILR------STFLN-	386
At-rbohJ	TTSPLYNITGRCLC----AAKGTAEILKLNMALILVP-VLRRTLTFLR------STFLN-	397
Hs-Nox5	----HRD-LGASVM----VAKGCGQCLNFDCSFIAVL-MLRRCLTWLRA------TWLA-	275
Cf-Nox5	----HRA-RGASVM----VAKGCGQCLNLDGSFIAVL-MLRRCLTWLRA------TWLA-	301
Bt-Nox5	----YRA-FGSSVM----VAKGCGQCLNFDCSFIAVL-MLRRCLTWLRA------TWLA-	309
Md-Nox5	----YQS-SGTRIM----IARGGGQCLNFNCSFIVVL-MLRRCLTWLRA------TWLA-	281
Xt-Nox5	----HAS-LGGWIM----VAKGCGQCLNFNCTFIVVM-MLRRCLTWLRT------TCVV-	290
Tr-Nox5	----HSY-GGGWYM----VAKGCGQCLNFNCTFIMVL-MLRRCLTWLRA------TWVV-	217
Tn-Nox5	AGRGLEPRVKRASP-----------------TCVQVL-MLRRCLTWLRA------TWVV-	70
Gg-Nox5	----HFG-GSGWVA----AARGCGQCLNFNCPFIAVMPMLRACLTRLRA------TPAG-	266
Ol-Nox5	----HSD-GGVWLM----VARGCGQCLNFNCTFIVVL-MLRRCLTWLRA-----TWVVR-	287
Dr-Nox5	----HAD-GGLWIM----LARGCGQCLNLNCTFVMVL-MLRRCLTWLRA-----TWVVR-	173
Sp-Nox5A	---YAKKKSNWCLI----TARGCGQCLNFNSAFVLVL-MLRKTITTLRT-----TKAAE-	279
Sp-Nox5B	YQSVSDERHPAAIS----IARSAGRCLSFECCFVLVL-MLRKLLTILRN-----TFLMS-	244
Ag-Nox5	---YRN--SNGFVI----MARACGQCLNFNCAFILVL-MLRQCITFLRT-----RGFTA-	329
Am-Nox5	---YRK--SNGYVM----LARACGQCLNFNCSFILVL-MLRQCITFLRT-----HGFNS-	331
Dm-Nox5	---YRA--SNGFVI----IARACGQCLNFNCAWVLVL-MLRHSLTYLRG-----RGLSS-	334
Dd-NoxC	VHANN-KRAIELFGPGVYITRIAAQLIEFNAAIILMT-MCKQLFTMIRN-------TKFK	653

```
Mg-NoxC     KYAS-GEQYQAAFGWGVALAKLCAGALYPTFFFLILS-MSRYFSTFLRR------SYHLS 296
Fg-NoxC     KYQT-TPGYRAAFGWGVVMAKTCAGALYPTFFFLILS-MSRYFSTWLRR------SYHIS 221
Cc-NoxD     FEDNFDMPHFNTVRWFIGIARGMGYTLNLNTAFVILL-ASRLLFTKLRD------SPLQL 103
Py-NoxD     LWP---VGHYR--RNLTPVARGAGNLINFNSAVILLV-SARKFMSWLRNT------PLNM 98
Xt-Duox1    GFASPSSGIADATFIGLIISRGSAASISFMFSYMLLT-MCRNLITLLRET-------FLN 1072
Xt-Duox2    AFESQHRGISEVTMPGIIISRGTAASISFMFSYILLT-MCRNLITFLRET-------FLN 1122
Gg-Duox     AFASPSTGIAQTTFVGIIISRGSAACISFMYSYILLT-MCRNLITVLRET-------FLN 1016
Tr-Duox     GFQAESTGLPETSVVGVALARGSAAAVSFLFPYMLLT-VCRNLITLCRET-------FLN 1042
Tn-Duox     GFQAESTGLPETSVVGVAFARGSAAAISFLFPYMLLT-VCRNIITLCRET-------FLN 1133
Ol-Duox     GLQAEATGVPGTTVVGIIVARGSAAGVSFLFPYMLLT-VCRNLITLCRET-------FLN 1119
Dr-Duox     GLQAHSSGIPETSMVDVLVSRGSAAAISFLFPYMLLT-VCRNLITMCRET-------FLN 1070
Mm-Duox2    GFASPPTDIEETTYVGIILSRGTAASISFMFSYILLT-MCRNLITFLRET-------FLN 1084
Rn-Duox2    GFASPPTDIEETTYVGIILSRGTAASISFMFSYILLT-MCRNLITFLRET-------FLN 1084
Hs-Duox2    GFASPPSDIAQTTLVGIILSRGTAASVSFMFSYILLT-MCRNLITFLRET-------FLN 1115
Cf-Duox2    AFASPPSGIAETTFVGIILSRGTAASISFMFSYILLT-MCRNLITFLRET-------FLN 867
Hs-Duox1    AFAAHHTGITDTTRVGIILSRGTAASISFMFSYILLT-MCRNLITFLRET-------FLN 1118
Cf-Duox1    AFGAHHMGITDTTRVGIILSRGTAASISFMFSYILLT-MCRNLITFLRET-------FLN 1118
Rn-Duox1    AFAAHHSGITDTTRVGIILSRGTAASISFMFSYILLT-MCRNLITFLRET-------FLN 1114
Mm-Duox1    AFAAHHSGITDTTRVGIILSRGTAASISFMFSYILLT-MCRNLITFLRET-------FLN 1118
Ci-DuoxA    SVEREFAGLRRIAGYGVSITRGAASAMMFTYTSLLVT-MCRNTITALRET-------FLH 1144
Ci-DuoxC    VYSKYATGLYGIGGPLMALARASAAALMFNFSTLLLT-MCRNIITFLRET-------FLH 1009
Ci-DuoxD    TIFNKAAGLGVIAGNALPLARASAAALMFNISTLLLT-MCKNILTSLRET-------QLH 1087
Sp-Duox     SVEREFAGLRRIAGFGVSVTRGAASAMMFTYSSLLVT-MCRNTITKLRET-------FLH 1241
Dm-Duox     SFMAEHTDLRHIMGVGIAITRGSAASLSFCYSLLLLT-MSRNLITKLKEF-------PIQ 1041
Ag-Duox     SFMAEHTDLRHIMGVGIAITRGSAASLSFCYSLLLLT-MSRNLLTKLKEF-------PIQ 1042
Am-Duox     SFMAEHTDLRHIMGVGIAITRGSAAALSFCYSLLLLT-MSRNLLTKLKEF-------SIQ 1047
Ci-DuoxB    AFGAEHIGIRRVTEWGIIISRASAAAISFHFSFILLT-MSRNLITTCRET-------FLK 1062
Ce-Duox1    RYMAENRDLRRVMGAGIAITRGAAGALSFCMALILLT-VCRNIITLLRET-------VIA 1061
Ce-Duox2    RYLNEDRDLRRVMGAGIAITLSSAGALSFCMALILLT-VCRNIITLLRET-------VIA 1067
                               *  *               *      *

                   ________________TM(III)____________________
Hs-Nox1     LRKQLD-HNLTFHKLVA-YMICLHT-----------------------AIHIIAHLFNF- 123
Cf-Nox1     LRKQLD-HNLTFHKLVA-YMICLHT-----------------------AIHIIAHLFNF- 123
Mm-Nox1     LRKPLD-HNLTFHKLVA-YMICIFT-----------------------VIHIIAHLFNF- 123
Rn-Nox1     LRKPLD-HNLTFHKLVA-YMICIFT-----------------------AIHIIAHLFNF- 123
Gg-Nox1     LRKQLD-HNLTFHKLVA-YALALLT-----------------------AVHTIAHLFNL- 123
Xt-Nox1     MRKQLD-NNLAFHKLVG-YTIALMT-----------------------AIHTIAHLFNV- 123
Tn-Nox1     MRKQLD-KNLSFHKLVA-YMIALMT-----------------------AVHTVAHLLNV- 123
Tr-Nox1     MRKQLD-KNLSFHKLVA-YMIALMT-----------------------AVHMIAHLLNV- 123
Ol-Nox1     MRKQLD-KNLSFHKLVA-YMIALMT-----------------------AVHTVAHLLNL- 123
Dr-Nox1     VRKQLD-KNLTFHKLVA-YMIALMT-----------------------AVHTIAHLFNA- 123
Mm-Nox2     IRRQLD-RNLTFHKMVA-WMIALHT-----------------------AIHTIAHLFNV- 123
Rn-Nox2     IRRQLD-RNLTFHKMVA-WMIALHT-----------------------AIHTIAHLFNV- 123
Hs-Nox2     VRRQLD-RNLTFHKMVA-WMIALHS-----------------------AIHTIAHLFNV- 123
Cf-Nox2     IRRQLD-RNLTFHKLVA-WMIALHT-----------------------AIHTIAHLFNV- 123
Gg-Nox2     VRRQLD-RNLTFHKMVA-WMIALHT-----------------------AIHTIAHLFNV- 123
Xt-Nox2     LRRQLD-RNLTFHKMVA-WMIALHT-----------------------AIHTGAHLFNV- 123
Tn-Nox2     AARQLD-RNLTFHKLVA-YMIAFHT-----------------------AVHIVAHLFNF- 122
Tr-Nox2     AARQLD-RNITFHKLVA-YMIAFHT-----------------------AVHIVAHLFNF- 122
Ol-Nox2     AARQLD-RNLTFHKLVA-YMIAFHT-----------------------AVHIIAHLFNF- 122
Dr-Nox2     AARQLD-RNITFHKLVA-YMIAFHT-----------------------AVHIIAHLFNF- 122
Hs-Nox3     WRRQLD-KNLRFHKLVA-YGIAVNA-----------------------TIHIVAHFFNL- 124
Cf-Nox3     WRRQLD-KNLKFHKLVA-YGIAVNA-----------------------TIHIVAHLCNL- 124
```

```
Mm-Nox3     WRRQLD-KNLNFHKLVA-YGIAVNS-----------------------VIHIVAHLFNL- 124
Rn-Nox3     WRRQLD-KNLKFHKLVA-YGIAVNS-----------------------VIHIVAHLFNL- 124
Gg-Nox3     PRRQLD-KNIAFHKVVA-YGIAVNA-----------------------TIHIVAHLINI- 123
Ci-Nox2     IRRVLD-KNIKFHRMCA-YMIVLMT-----------------------LIHYFAHCFNV- 127
Sp-Nox2A    VRRQLD-KNLTFHKTVA-YMIVVWT-----------------------IVHVVAHAFNF- 126
Sp-Nox2B    LRRQLD-KNITFHKLIA-YAIGFFV-----------------------ILHVGAHCFNL- 111
Mm-Nox4     TRRLLD-KSKTLHITCG-VTICIFS-----------------------GVHVAAHLVNA- 127
Rn-Nox4     TRRLLD-KSKTLHITCG-ITICIFS-----------------------GVHVAAHLVNA- 127
Hs-Nox4     TRRLLD-KSRTFHITCG-VTICIFS-----------------------GVHVAAHLVNA- 127
Cf-Nox4     TRRLLD-KSRTFHITCG-VTICIFS-----------------------GVHVAAHLVNA- 131
Gg-Nox4     TRRLID-KSKTFHVTCG-VTVCIFS-----------------------VLHVAAHLVNA- 87
Xt-Nox4     TRRMLD-KHKTFHAACG-LAICLFS------------------------AHVLNA----- 123
Ol-Nox4     MRRLLD-KSKSFHVACG-IAICIFS-----------------------AVHVSAHLINV- 127
Tr-Nox4     TRRMLD-KYKTFHVACG-LAICIFS-----------------------AIHVSAHLANA- 125
Ci-Nox4     VRRLLD-RGRSFHILCG-YILCLLA-----------------------GVHCAAHAYNA- 125
Ag-Nox      LVHYLE-KTKVLHLILG-CSLLIVA-----------------------IVHSVAHFVNI- 109
Pa-NoxA     KFIPLD-ENIWMHRQLA-YSMLLFT-----------------------IIHTAAHYVNF- 121
Mg-NoxA     RFIPLD-ENIWFHRQIA-YAMLIFS-----------------------ITHTAAHYVNF- 120
Fg-NoxA     RFLPLD-ENLWMHRQLA-YSMLLFT-----------------------CLHTGAHYVNF- 124
An-NoxA     RWLPLD-ENIWFHRQVA-YATLVFT-----------------------ILHVAAHYVNF- 119
Pa-NoxB     GIIQFD-KNITFHITTA-WSIVFWS-----------------------WVHTIAHWNNF- 169
Fg-NoxB     GILQFD-KNITFHIVTA-WSIVFWS-----------------------WVHTIAHWNNF- 158
Mg-NoxB     GIIQFD-KNITFHITTA-WSIVFFS-----------------------WVHTVAHWNNF- 173
Dd-NoxA     NYVPFD-KNIVFHKLIA-WVICFAT-----------------------FGHVMAHFNNF- 123
Dd-NoxB     NYIPID-KHLNFHKLCA-FMLFCCT-----------------------IIHCVGHYISF- 290
At-rbohC    RVVPFD-DNLNFHKVIA-VGIIVGV-----------------------TMHAGAHLACD- 456
At-rbohG    VFVPFD-DNLNFHKVIA-VGIAIGV-----------------------AIHSVSHLACD- 408
At-rbohA    AIVPFD-DSLNFHKVIA-IGISVGV-----------------------GIHATSHLACD- 449
At-rbohB    SVVPFD-DNINFHKVVA-FGIAVGI-----------------------GLHAISHLACD- 403
At-rbohD    TVVPFD-DSLNFHKVIA-SGIVVGV-----------------------LLHAGAHLTCD- 481
At-rbohF    YFVPFD-DNINFHKTIA-GAIVVAV-----------------------ILHIGDHLACD- 491
At-rbohI    HSVPFD-DCINFHKTIS-VAIISAM-----------------------LLHATSHLACD- 479
At-rbohE    ACVPFD-DNINFHKIIA-CAIAIGI-----------------------LVHAGTHLACD- 470
At-rbohH    RVVPFD-DNINFHKVIA-YMIAFQA-----------------------LLHTALHIFCN- 420
At-rbohJ    HLIPFD-DNINFHKLIA-VAIAVIS-----------------------LLHTALHMLCN- 431
Hs-Nox5     QVLPLD-QNIQFHQLMG-YVVVGLS-----------------------LVHTVAHTVN-- 307
Cf-Nox5     QVLPLD-QNIQFHQFVG-YVVIVLS-----------------------LVHTVAHIVNF- 335
Bt-Nox5     QVLPLD-HNIQFHQLMG-YVVVGLS-----------------------LVHTVAHVVNF- 343
Md-Nox5     RVLPLD-QNVEFHQLIG-YVVVGFS-----------------------FLHTTAHVVNF- 315
Xt-Nox5     RFLPLD-QNVVLHELIG-YVIFVLT-----------------------VIHTAAHVTNF- 324
Tr-Nox5     RVLPLD-QNILLHQIVG-YAILFYT-----------------------LLHTSAHIFNF- 251
Tn-Nox5     RVLPLD-QNILLHQIVG-YAILFYT-----------------------LLHTCAHVFNFG 105
Gg-Nox5     RALPLE-HCVALHQPVG-SAVLALA-----------------------VLHAGAHVANYG 301
Ol-Nox5     -VLPLD-QNILLHQIVG-YAIFCFS-----------------------LGHAIAHVVN-- 319
Dr-Nox5     -VLPLD-QNILLHQIVG-YAILIFS-----------------------VGHTGAHIMN-- 205
Sp-Nox5A    -ILPTD-QNIVFHKLVG-IFIALLS-----------------------GIHTLGHIGNAW 313
Sp-Nox5B    -VLPLD-QHVVIHKIVA-VFIIILS-----------------------VIHTAGHIANIG 278
Ag-Nox5     -FLPLD-QHIYLHKLTG-VLVAIFS-----------------------LVHTIMHLFNFT 363
Am-Nox5     -VLPLD-QHIYLHKVTG-GLICVFS-----------------------IAHTLMHLLNFG 365
Dm-Nox5     -YLPLD-HHVYLHKLTG-ITISVLS-----------------------LIHTIMHLFNFS 368
Dd-NoxC     FLFPVD-KYMTFHKLIG-YTLIIAS-----------------------FLHTIGWIVGM- 687
Mg-NoxC     RFINWD-LSQEFHIKIS-IVALVLA-----------------------SLHALGHLSG-- 329
Fg-NoxC     RFFNWD-LSQEFHIRIS-CVAILLA-----------------------TLHAIGHLTG-- 254
Cc-NoxD     -VLPFDAAFPALHIVVGY------------------------TIFFAVLVHGSFHFVWLI 138
Py-NoxD     -VVPFDKAMPAFHMLVGR------------------------VFLAASVVHVGFHLPVYV 133
```

```
Xt-Duox1          QYIPFD-SAVDFHRLIA-VTALVLS-----------------------ILHSLGHLVNVY 1107
Xt-Duox2          QYIPFD-AAVDFHRLVA-KTAIILT----------------------EFKLLFSHQTDIK 1158
Gg-Duox           RYIPFD-AAVDFHRWIA-MAALIFS-----------------------VLHTAGHLVNVY 1051
Tr-Duox           RYIPFD-AAIDFHRFMA-MAAIVLS-----------------------VVHTLAHVVNIY 1077
Tn-Duox           RYVPFD-AAIDFHRLMA-MTAIVLAGWRSRYEGTHLRARKLTAASPPPVVHTLAHVVNIY 1191
Ol-Duox           RFIPFD-AAIDFHRSLA-MTAVVLS-----------------------VAHSLAHVVNIY 1154
Dr-Duox           RYIPFD-AAIDLHRQMA-ATALILS-----------------------VVHSLGHLVNVY 1105
Mm-Duox2          RYIPFD-AAVDFHRWIA-MAAVVLA-----------------------VLHSAGHAVNVY 1119
Rn-Duox2          RYIPFD-AAVDFHRWIA-MAAVVLA-----------------------VVHSLGHAVNVY 1119
Hs-Duox2          RYVPFD-AAVDFHRWIA-MAAVVLA-----------------------ILHSAGHAVNVY 1150
Cf-Duox2          RYVPFD-AAVDFHRWIA-MAAVVLA-----------------------ILHSAGHAVNVF 902
Hs-Duox1          RYVPFD-AAVDFHRLIASTAIPVA------------------------VLHSVGHVVNVY 1153
Cf-Duox1          RYVPFD-AAVDFHRLIASTAIVLTG-----------------------RALDAGHVVNVY 1154
Rn-Duox1          RYIPFD-AAVDFHRFIASTAIILT------------------------VLHSAGHVVNVY 1149
Mm-Duox1          RYIPFD-AAVDFHRLIASTAIILT------------------------VLHSAGHVVNVY 1153
Ci-DuoxA          LYIPFD-SAITMHRIIAWLALFFTG-----------------------MHIIGHSLNIY 1179
Ci-DuoxC          RFIPFD-SAVTMHRIVAWMALAFTA-----------------------LHILAHGINFY 1044
Ci-DuoxD          LYIPFD-AAVAFHKLVAWMALFFTA-----------------------LHIIAHGINFY 1122
Sp-Duox           RYVPFD-SALNMHKLIAMLALFFSI-----------------------MHTIGHSINFY 1276
Dm-Duox           QYIPLD-SHIQFHKIAACTALFFSV-----------------------LHTVGHIVNFY 1076
Ag-Duox           QYIPLD-SHIQFHKIAACTALFFSL-----------------------LHTVGHIVNFY 1077
Am-Duox           QYIPLD-SHIQFHKIAACTALFFSV-----------------------LHTVGHMVNFY 1082
Ci-DuoxB          NFIPFD-SAVAFHKQIAYVALVETI-----------------------FHVLGHASNFY 1097
Ce-Duox1          QYIPFD-SAIAFHKIVALFAAFWAT-----------------------LHTVGHCVNFY 1096
Ce-Duox2          QYIPFD-SAIAFHKIVALFTLFWST-----------------------LHTIGHCVNFY 1102
                      **     *                                   *   *

Hs-Nox1           ---------------------DCYSRSRQA-----------TDGSLASILSSLSHDEKKG 151
Cf-Nox1           ---------------------ERYSRSRQA-----------TDGSLASILSTLSHQGKEE 151
Mm-Nox1           ---------------------ERYRRSQQA-----------MDGSLASVLSSLSHPEKE- 150
Rn-Nox1           ---------------------ERYSRSQQA-----------MDGSLASVLSSLFHPEKE- 150
Gg-Nox1           ---------------------ERYNHSQQA-----------NDGSLHAVLSKMHLQDSN- 150
Xt-Nox1           ---------------------ERYCDAAQK-----------KTDTLPGELSSIG----ED 147
Tn-Nox1           ---------------------EWYNNSRQG-----------VYDELSTALSKLDDAN--G 149
Tr-Nox1           ---------------------EWYNNSRQG-----------VYDKLSTALSNLEDTK--N 149
Ol-Nox1           ---------------------EWLNNSKLG-----------VYGKLSTALSNLEDEG--N 149
Dr-Nox1           ---------------------ERYSNSLEG-----------EDGDLAFELSLLQDSSELN 151
Mm-Nox2           ---------------------EWCVNARVG-----------ISDRYSIALSDIGDNE--N 149
Rn-Nox2           ---------------------EWCVNARVG-----------TSDPYSVALSNIGDKE--N 149
Hs-Nox2           ---------------------EWCVNARVN-----------NSDPYSVALSELGDRQ--N 149
Cf-Nox2           ---------------------EWCVNARVN-----------NSDVYSIALSNLGDNP--G 149
Gg-Nox2           ---------------------EWSVHARVE-----------EEGTLAAVLSRLGDSP--N 149
Xt-Nox2           ---------------------ERLVDARVE-----------ANGTIQAALTDLGDRE--G 149
Tn-Nox2           ---------------------EFFMDAQLNR----------NSSYLPFILSEIGTGD--N 149
Tr-Nox2           ---------------------EFFMDAQLNR----------NSSYLPFILSEIGTGD--N 149
Ol-Nox2           ---------------------EYFMDAQLNR----------NHSHLPFILSEIGNDE--N 149
Dr-Nox2           ---------------------ERFMDSQLMI----------NSSHLPYVLSQIGNND--N 149
Hs-Nox3           ---------------------ERYHWSQSE-----------EAQGLLAALSKLGNTP--N 150
Cf-Nox3           ---------------------QRYHWSQSA-----------EVQGLPATLSKLGNAP--N 150
Mm-Nox3           ---------------------ERYHLGQAK-----------DAEGLLAALSKLGDAP--N 150
Rn-Nox3           ---------------------ERYHLGQAK-----------DAEGLLAALSKLGNAP--N 150
Gg-Nox3           ---------------------ERYHNSQSK-----------EAGGLQNKLSGLGKRP--N 149
Ci-Nox2           ---------------------DFFTSAYQSKILATDTPAIIQQKKLIAKLIQIGNNG--N 164
```

```
Sp-Nox2A     ---------------------RNLYNHYRCVTT--------DNDELCEGISAIGRKFKAK 157
Sp-Nox2B     ---------------------QNLYNG-RKATS--------EDDWLANRLSQP--SFDLN 139
Mm-Nox4      ---------------------LNFSVNYSED-----------FLELNAARYQNEDPR--- 152
Rn-Nox4      ---------------------LNFSVNYSEH-----------FLALNAARYQNEDPR--- 152
Hs-Nox4      ---------------------LNFSVNYSED-----------FVELNAARYRDEDPR--- 152
Cf-Nox4      ---------------------LNFSVNYNED-----------FTELNAARYRDEDPR--- 156
Gg-Nox4      ---------------------LNFSENYNED-----------FLAINAANYRGEDPR--- 112
Xt-Nox4      ---------------------VNFSVNYNHE-----------FPSINVARYKN------- 144
Ol-Nox4      ---------------------VNFSAGFSED-----------FPALNLARYKGEDPK--- 152
Tr-Nox4      ---------------------ANFSTSYSEE-----------FPSLNVARYRGEDPK--- 150
Ci-Nox4      ---------------------VYFSKYYNSR-----------YKDLNVAKYSNQNPL--- 150
Ag-Nox       ---------------------VNFIDNYDER-----------YREINWANGPDDNVL--- 134
Pa-NoxA      ---------------------YNVEKTQ--------------IRPVTAVQIHYVQP---- 142
Mg-NoxA      ---------------------FNVERLQ--------------IRAQTAVQIHYAQP---- 141
Fg-NoxA      ---------------------YNVEITQ--------------IRPVTALQIHYAQP---- 145
An-NoxA      ---------------------YNIERKQ--------------LRPETALQIHYAQP---- 140
Pa-NoxB      ---------------------AQVAAKNN-------------LGIYGWLLANFVSG---- 191
Fg-NoxB      ---------------------AQVAIKYN-------------LGIYGWLLANFVSG---- 180
Mg-NoxB      ---------------------AQIAAQQK-------------LGIYGWLLANFVSG---- 195
Dd-NoxA      ---------------------R-LYQDITPQEYK-------RILGIDYPNLTPIKYA--- 151
Dd-NoxB      ---------------------KKINDDVLKIDDG-------KSVAGDYLNININNFPDEK 322
At-rbohC     --------------------FPRLLHATPEAYR---------PLRQFFGDEQPKSYWH-- 485
At-rbohG     --------------------FPLLIAATPAEYM---------PLGKFFGEEQPKRYLH-- 437
At-rbohA     --------------------FPRLIAADEDQYE---------PMEKYFG-PQTKRYLD-- 477
At-rbohB     --------------------FPRLLHAKNVEFE---------PMKKFFGDERPENYGW-- 432
At-rbohD     --------------------FPRLIAADEDTYE---------PMEKYFGDQ-PTSYWW-- 509
At-rbohF     --------------------FPRIVRATEYDYN---------RYLFHYFQTKQPTYFD-- 520
At-rbohI     --------------------FPRILASTDTDYK---------RYLVKYFGVTRPTYFG-- 508
At-rbohE     --------------------FPRIINSSPEQFV---------LIASAFN-GTKPTFKD-- 498
At-rbohH     --------------------YPRLSSCSYDVFL---------TYAGAALGNTQPSYLG-- 449
At-rbohJ     --------------------YPRLSSCPYNFYS---------DYAGNLLGAKQPTYLG-- 460
Hs-Nox5      ---------------------FVLQAQAEASP----------FQFWELLLTTRPG-IG-- 333
Cf-Nox5      ----------------------ALQAQAEAS----------PFQFWELLLTTRPG-IG-- 360
Bt-Nox5      ----------------------ALQAQSETS----------PFRFWELLLTTRPG-IG-- 368
Md-Nox5      ----------------------AQLAQSENS----------TFQFWEYLLTTRPG-IG-- 340
Xt-Nox5      ----------------------TLINLTEKTG---------AYTFWEYLLTIRPG-IG-- 350
Tr-Nox5      ------------------------VQLSESS----------GFTLWEYLLTTRPG-IG-- 274
Tn-Nox5      TPHTHARTVFPDAEGTCACFPCALQRTSESS----------GFTLWEYLVTTRPG-IG-- 152
Gg-Nox5      ----------------------RLAQDGHG-------------ALSEFLLVARPG-GG-- 323
Ol-Nox5      --------------------FDLTSTNLSQHSE---------FLLWEYLLTTRPM-IG-- 347
Dr-Nox5      --------------------FARLSQNDGAYQ------------LWEYLFTIRPG-IG-- 230
Sp-Nox5A     --------------------FVEKTTDGNVTMS---------ALLFTNPHLTSLG-LA-- 341
Sp-Nox5B     --------------------LIYQVENVNGTTA---------AWILDVLVRPFPG-LG-- 306
Ag-Nox5      --------------------TIVVYDPVLNANN---------YTTAEWLFTARPGLFG-- 392
Am-Nox5      --------------------TIVIYDEILNHNN---------YTLSEWLLTSRPALFG-- 394
Dm-Nox5      --------------------IIVINDPNINAGH---------YTIGEWLLTDRPGLFG-- 397
Dd-NoxC      ---------------------AVATGKPD-------------NIFYDCLAPHFKFRPTVW 713
Mg-NoxC      ---------------------TFNWGSRPER-----------QDAVGVLLGEDQVPRP-- 355
Fg-NoxC      ---------------------SFVHGSDPAN-----------EDAVAEALGPDKVPRP-- 280
Cc-NoxD      -----------------------TWDAWTWG----------------------------- 146
Py-NoxD      -----------------------VSKPWGPG----------------------------- 141
Xt-Duox1     ----------------------IFTIIPLS----------VLSCLFPTVFVDDGSDHPNK 1135
Xt-Duox2     ----------------------LMIVFTLRL---------CMSCYFLQATYSNDNVLSLN 1187
Gg-Duox      ----------------------IFSVTPLS----------VLSCLFSSVFMNDGP----- 1074
Tr-Duox      ----------------------IFSLSDLS----------ILSCLFPKVFRNNGSERPMK 1105
```

```
Tn-Duox             ----------------------IFSMSDLS----------ILACLFPKVFLNNGSELPMK 1219
Ol-Duox             ----------------------IFSISDLS----------ILSCLFPKVLNNNGSELPPK 1182
Dr-Duox             ----------------------IFCISDLS----------ILACLFPKVFSNNGSELPMK 1133
Mm-Duox2            ----------------------IFSVSPLSLMA------CVFPNVFVNDGSKFPPKYY-- 1149
Rn-Duox2            ----------------------IFSVSPLSLMT------CVFPSVFVNDGSKLPPKYY-- 1149
Hs-Duox2            ----------------------IFSVSPLSLLA------CIFPNVFVNDGSKLPQKFY-- 1180
Cf-Duox2            ----------------------IFSVSPLSLLA------CIFPNIFMNDGSQLPQKFY-- 932
Hs-Duox1            ----------------------LFSISPLSVLS------CLFPGLFHDDGSELPQKYY-- 1183
Cf-Duox1            ----------------------LFSISPLSVLS------CLFPGLFHNDGSEFPQKYY-- 1184
Rn-Duox1            ----------------------LFSISPLSVLS------CLFPDLFHDDGSEFPQKYY-- 1179
Mm-Duox1            ----------------------LFSISPLSVLS------CLFPGLFHDDGSEFPQKYY-- 1183
Ci-DuoxA            ----------------------AISTQTPGD----------LTCLFRDFWRT--SDVLPK 1205
Ci-DuoxC            ----------------------SIVTQSPDD----------MACLFRDMWYP--SDYIPT 1070
Ci-DuoxD            ----------------------SIQTQTPSD----------LLCLFRDLWFP--SDYRPT 1148
Sp-Duox             ----------------------HISTQTADD----------LTCYFRDFFHR--SHELPK 1302
Dm-Duox             ----------------------HVSTQSHEN----------LRCLTREVHFA--SDYKPD 1102
Ag-Duox             ----------------------HVSTQSIEN----------LKCLTKEVHFT--SDYRPD 1103
Am-Duox             ----------------------HVSTQPLAH----------LRCLTSELSFP--SDARLT 1108
Ci-DuoxB            ----------------------HFCVHPLPV----------LACLFPKIFVDDGSDLPKS 1125
Ce-Duox1            ----------------------HVGTQSQEG----------LACLFQEAFFG--SNFLPS 1122
Ce-Duox2            ----------------------HVGTQSDRG----------LACLFQETFFG--SDVVPT 1128


                                        __________TM(IV)________________________
Hs-Nox1             G--SWLNPIQ-----SRNTTVEYVTFTSIA------------------------------ 174
Cf-Nox1             D--SWLNPMQ-----SPNMTVEYVTFTSIA------------------------------ 174
Mm-Nox1             D--SWLNPIQ-----SPNMTVMYAAFTSIA------------------------------ 173
Rn-Nox1             D--SWLNPIQ-----SPNVTVMYAAFTSIA------------------------------ 173
Gg-Nox1             ---KWLNPIH-----SNQTTVEYVAFTTIP------------------------------ 172
Xt-Nox1             G--TWLNPVR-----SPTVTPPYFAFTTIA------------------------------ 170
Tn-Nox1             T--TYLNPIR-----ITDLIPTYFAFTTIA------------------------------ 172
Tr-Nox1             T--TYLNPIR-----SPTLS-TYFVFTTIA------------------------------ 171
Ol-Nox1             E--TFLNPLQHIEA-DPQQKPIKFAFTSIA------------------------------ 176
Dr-Nox1             T--TYLNPFP-----SNSTTPMIFVFTSIA------------------------------ 174
Mm-Nox2             E--EYLNFARE-KI-KNPEGGLYVAVTRLA------------------------------ 175
Rn-Nox2             E--EYLNFARE-KI-KNPEGGLYVAVTRLA------------------------------ 175
Hs-Nox2             E--SYLNFARK-RI-KNPEGGLYLAVTLLA------------------------------ 175
Cf-Nox2             E--SYLNFARR-RI-KNPEGGLYVAVTLLA------------------------------ 175
Gg-Nox2             E--SYINFYRQ-TI-PNPVGGLYVAFTYLA------------------------------ 175
Xt-Nox2             E--SYLNFVRS-RV-PNPIGGINVAFTFLA------------------------------ 175
Tn-Nox2             A--SFLNPIRT-NE-TN---PTIVMFTTIA------------------------------ 172
Tr-Nox2             A--SFLNPIRT-NE-TN---PTIVMFTTIA------------------------------ 172
Ol-Nox2             V--SFLNPIRS-NE-TKS--PTIVMFTTIA------------------------------ 173
Dr-Nox2             R--SYLNPIRS-ND-TN---PTIVMFTTVA------------------------------ 172
Hs-Nox3             E--SYLNPVR-----TFPTNTTTELLRTIA------------------------------ 173
Cf-Nox3             E--SYLNPIR-----TFHTNTITELLTTIA------------------------------ 173
Mm-Nox3             E--SYLNPVR-----TFDMGTTTELLMTVS------------------------------ 173
Rn-Nox3             E--SYLNPVR-----TLYTGTTTQLLMTVS------------------------------ 173
Gg-Nox3             E--SYLNPIR-----TYETNTTGEVLTTIA------------------------------ 172
Ci-Nox2             E--TYLNPIR------KSVFSVGAVFLLTG------------------------------ 186
Sp-Nox2A            PEDNWLNPIQGAKTLPAGLGLIEQALIPIA------------------------------ 187
Sp-Nox2B            P----FKTIRSSD--VSGLGVIGPGLSLLA------------------------------ 163
Mm-Nox4             ----------------------KLLFTTIP------------------------------ 160
Rn-Nox4             ----------------------KLLFTTVP------------------------------ 160
```

```
Hs-Nox4      ----------------------KLLFTTVP------------------------------ 160
Cf-Nox4      ----------------------KLLFTTVP------------------------------ 164
Gg-Nox4      ----------------------KLLFATVP------------------------------ 120
Xt-Nox4      --------------------------EVVP------------------------------ 148
Ol-Nox4      ----------------------LIIFTTIP------------------------------ 160
Tr-Nox4      ----------------------WIILTTIP------------------------------ 158
Ci-Nox4      ----------------------LMLVTSLS------------------------------ 158
Ag-Nox       ----------------------RLLFATPT------------------------------ 142
Pa-NoxA      -----------------------------G------------------------------ 143
Mg-NoxA      -----------------------------G------------------------------ 142
Fg-NoxA      -----------------------------G------------------------------ 146
An-NoxA      -----------------------------A------------------------------ 141
Pa-NoxB      -----------------------------P------------------------------ 192
Fg-NoxB      -----------------------------P------------------------------ 181
Mg-NoxB      -----------------------------P------------------------------ 196
Dd-NoxA      -------------------------FATLA------------------------------ 156
Dd-NoxB      Y----------------------LFFKSVP------------------------------ 330
At-rbohC     ------------------------FVNSVE------------------------------ 491
At-rbohG     ------------------------FVKSTE------------------------------ 443
At-rbohA     ------------------------FVQSVE------------------------------ 483
At-rbohB     ------------------------FMKGTD------------------------------ 438
At-rbohD     ------------------------FVKGVE------------------------------ 515
At-rbohF     ------------------------LVKGPE------------------------------ 526
At-rbohI     ------------------------LVNTPV------------------------------ 514
At-rbohE     ------------------------LMTGAE------------------------------ 504
At-rbohH     ------------------------LMLTSV------------------------------ 455
At-rbohJ     ------------------------LMLTPV------------------------------ 466
Hs-Nox5      ------------------------WVHGSA------------------------------ 339
Cf-Nox5      ------------------------WIHGLA------------------------------ 366
Bt-Nox5      ------------------------WVHGSA------------------------------ 374
Md-Nox5      ------------------------WVYGTA------------------------------ 346
Xt-Nox5      ------------------------WISGTA------------------------------ 356
Tr-Nox5      ------------------------WVKGTA------------------------------ 280
Tn-Nox5      ------------------------WVKGTA------------------------------ 158
Gg-Nox5      ------------------------GFGGTA------------------------------ 329
Ol-Nox5      ------------------------WVKGTA------------------------------ 353
Dr-Nox5      ------------------------WVNGTA------------------------------ 236
Sp-Nox5A     ------------------------PVSGSA------------------------------ 347
Sp-Nox5B     ------------------------LVEGSC------------------------------ 312
Ag-Nox5      ------------------------LIGGCA------------------------------ 398
Am-Nox5      ------------------------LVRGYA------------------------------ 400
Dm-Nox5      ------------------------LIPGCA------------------------------ 403
Dd-NoxC      E----------------------MIFNSLP------------------------------ 721
Mg-NoxC      ---------------------YSAYVSSLP------------------------------ 364
Fg-NoxC      ---------------------YIDYVRSLP------------------------------ 289
Cc-NoxD      ---------------------LWSFNMSV------------------------------- 154
Py-NoxD      ---------------------YNGFTQLF------------------------------- 149
Xt-Duox1     Y--------------------YWWFFETVP------------------------------ 1145
Xt-Duox2     L--------------------HPECLPLLT------------------------------ 1197
Gg-Duox      ---------------------DPFPILPFT------------------------------ 1083
Tr-Duox      W--------------------SWWFFQTVP------------------------------ 1115
Tn-Duox      W--------------------SWWFFETVPGTAEVKMVNLSSHRKMMPVHPGCRKATRTP 1259
Ol-Duox      W--------------------YWWFLQTVP------------------------------ 1192
Dr-Duox      W--------------------TFWFFKTVP------------------------------ 1143
Mm-Duox2     ----------------------WWFFETVP------------------------------ 1157
```

```
Rn-Duox2    ----------------------WWFFETVP------------------------------ 1157
Hs-Duox2    ----------------------WWFFQTVP------------------------------ 1188
Cf-Duox2    ----------------------WWFFQTVP------------------------------ 940
Hs-Duox1    ----------------------WWFFQTVP------------------------------ 1191
Cf-Duox1    ----------------------WWFFQTVP------------------------------ 1192
Rn-Duox1    ----------------------WWFFQTVP------------------------------ 1187
Mm-Duox1    ----------------------WWFFQTVP------------------------------ 1191
Ci-DuoxA    FH--------------------YWCWQTIT------------------------------ 1215
Ci-DuoxC    FV--------------------FWLFQTIT------------------------------ 1080
Ci-DuoxD    FV--------------------FWCLQTLT------------------------------ 1158
Sp-Duox     FH--------------------YWAWGTIT------------------------------ 1312
Dm-Duox     IT--------------------FWLFQTVT------------------------------ 1112
Ag-Duox     IT--------------------YWLFQTIT------------------------------ 1113
Am-Duox     IS--------------------FWLFRTVT------------------------------ 1118
Ci-DuoxB    LT--------------------WWFFETIT------------------------------ 1135
Ce-Duox1    IS--------------------YWFFSTIT------------------------------ 1132
Ce-Duox2    LS--------------------YWFYGTIT------------------------------ 1138


            __________TM(IV)______                                ____TM(V)__
Hs-Nox1     -------GLTGVIMTIALILMVTSATEFIRR--------------SYFEVFWYTHHLFIF 213
Cf-Nox1     -------GLTGVIITIALVLMVTSAMEFIRR--------------SYFEVFWYTHHIFII 213
Mm-Nox1     -------GLTGVIATVALVLMVTSAMEFIRR--------------NYFELFWYTHHLFIV 212
Rn-Nox1     -------GLTGVVATVALVLMVTSAMEFIRR--------------NYFELFWYTHHLFII 212
Gg-Nox1     -------GLTGVIITLALILMVTSSTEFIRR--------------NYFEVFWYTHHLFII 211
Xt-Nox1     -------GLTGVVITLALILMITSSTEFIRR--------------CYFEVFWYTHHLFVI 209
Tn-Nox1     -------GLTGVIITLSLILIITSSMEVIRR--------------SYFEVFWYTHHLFII 211
Tr-Nox1     -------GLTGVIITLALILIITSSMEVIRR--------------SYFEVFWYTHHLFII 210
Ol-Nox1     -------GLTGVVITLSLILIITSSMEVIRR--------------SYFEVFWYIHHLFIV 215
Dr-Nox1     -------GLTGVVITLALILMITSSMEVIRR--------------SYFEVFWYTHHLFIV 213
Mm-Nox2     -------GITGIVITLCLILIITSSTKTIRR--------------SYFEVFWYTHHLFVI 214
Rn-Nox2     -------GITGIVITLCLILIITSSTKTIRR--------------SYFEVFWYTHHLFVI 214
Hs-Nox2     -------GITGVVITLCLILIITSSTKTIRR--------------SYFEVFWYTHHLFVI 214
Cf-Nox2     -------GITGIVITLCLILIITSSTKTIRR--------------SYFEVFWYTHHLFVI 214
Gg-Nox2     -------GLTGVIITLALILIITSSTKIIRR--------------SYFEVFWYTHHLFVI 214
Xt-Nox2     -------GLTGVVITLALILIITSSTKTIRR--------------SYFEVFWYTHHLFVI 214
Tn-Nox2     -------GLTGVAITLALILIITSSMEVIRR--------------SYFEVFWYTHHLFVI 211
Tr-Nox2     -------GLTGVAITLALILIITSSMEVIRR--------------SYFEVFWYTHHLFII 211
Ol-Nox2     -------GLTGVVITLALILIITSSMEVIRR--------------SYFEVFWYTHHLFVI 212
Dr-Nox2     -------GLTGVVITLALILIITSSMEVIRR--------------SYFEVFWFTHHLFIV 211
Hs-Nox3     -------GVTGLVISLALVLIMTSSTEFIRQ--------------ASYELFWYTHHVFIV 212
Cf-Nox3     -------GVTGLIISLALVLIMTSSTESIRQ--------------VSYELFWYTHHVFII 212
Mm-Nox3     -------GITGLGISLALVFIMTSSTEFIRR--------------SSYELFWYTHHIFVF 212
Rn-Nox3     -------GITGLVISLALILIMTSSTEFIRQ--------------SSYELFWYTHHIFIF 212
Gg-Nox3     -------GVTGVMITVAFVLIVTSSTELIRR--------------SCYEVFWYTHHLFVV 211
Ci-Nox2     -------GWTGVIITLSLFFMVTSSLEFIRR--------------SYFEVFWFTHHLFIV 225
Sp-Nox2A    -------GWSGAVLTLALILMFSSATEFIRR--------------SYFETFWITHHLFIV 226
Sp-Nox2B    -------GWTGAVLALTYILMFTSATEFIR---------------YYFETFWLTHHLFVI 201
Mm-Nox4     -------GLTGVCMVVVLFLMVTASTYAIRV--------------SNYDIFWYTHNLFFV 199
Rn-Nox4     -------GLTGVCMVVVLFLMVTASTYAIRV--------------SNYDIFWYTHNLFFV 199
Hs-Nox4     -------GLTGVCMVVVLFLMITASTYAIRV--------------SNYDIFWYTHNLFFV 199
Cf-Nox4     -------GLTGVGMVLVLFLMITASTYAIRV--------------SNYDIFWYTHNLFFV 203
Gg-Nox4     -------GLTGVIMVLVLFLMCTASTYAIRV--------------SNYDIFWYTHNLFFV 159
Xt-Nox4     -------GVTGVLMVLILFLMCTASTSSIRT--------------ANYGIFLHTHNLFFI 187
```

```
Ol-Nox4        -------GVSGVLLVLILLLMFISSSHCIRV--------------CNYEIFWYTHNLFIV 199
Tr-Nox4        -------GVTGIVLVLILFLMLMSSSNCVRS--------------FNYEIFWYTHNLFIV 197
Ci-Nox4        -------GITGILLVISLVVISAFASRPIRR--------------NNHNKFWKTHHIFIV 197
Ag-Nox         -------GFSGCIMLLTLAAMAYLARRSMRD--------------RFYNSFFTSHHLFLV 181
Pa-NoxA        -------GATGHVMLLCMLLMYTTAHHRIRQ--------------QSFETFWYTHHLFIP 182
Mg-NoxA        -------GATGHMMLLCMLLMYTTAHHRIRQ--------------QSFETFWYTHHLFIP 181
Fg-NoxA        -------GITGHIMLLCMLLMFTSAHARIRQ--------------QSFETFWYTHHLFIP 185
An-NoxA        -------GVTGHVMLFCMMLMYTTAHHRIRQ--------------QSFETFWYTHHLFIP 180
Pa-NoxB        -------GWTGYVMLIALMGMVITSVEKTRR--------------ANYERFWYTHHMFIV 231
Fg-NoxB        -------GWTGYVMLIALMGMVLTSMEKPRR--------------ANFERFWYTHHMFIV 220
Mg-NoxB        -------GWTGYVMLIALMGMVFTSVEKPRR--------------ANYERFWYTHHFFIV 235
Dd-NoxA        -------GWTGHVVCIVMVLMYTSAVESIRR--------------PMFEGFWYTHHLFVV 195
Dd-NoxB        -------GITGHIMLLILILIVSSSMWRIRR--------------PMFEIFWYVHHLFIP 369
At-rbohC       -------GITGLVMVLLMAIAFTLATPWFRRGKLN-YLPGPLKKLASFNAFWYTHHLFVI 543
At-rbohG       -------GITGLVMVFLMVIAFTLAMPWFRRGKLEKKLPGPLKKLASFNAFWYTHHLFVI 496
At-rbohA       -------GVTGIGMVVLMTIAFTLATTWFRRNKLN--LPGPLKKITGFNAFWYSHHLFVI 534
At-rbohB       -------GWTGVTMVVLMLVAYVLAQSWFRRNRAN--LPKSLKRLTGFNAFWYSHHLFVI 489
At-rbohD       -------GWTGIVMVVLMAIAFTLATPWFRRNKLN--LPNFLKKLTGFNAFWYTHHLFII 566
At-rbohF       -------GITGILMVILMIISFTLATRWFRRNLVK--LPKPFDRLTGFNAFWYSHHLFVI 577
At-rbohI       -------GITGIIMVAFMLIAFTLASRRCRRNLTK--LPKPFDKLTGYNAFWYSHHLLLT 565
At-rbohE       -------GITGISMVILTTIAFTLASTHFRRNRVR--LPAPLDRLTGFNAFWYTHHLLVV 555
At-rbohH       -------SITGVLMIFFMGFSFTLAMHYFRRNIVK--LPKPFNVLAGFNAFWYAHHLLVL 506
At-rbohJ       -------SVTGVLMIIFMGISFTLAMHYFRRNIVK--LPIPFNRLAGFNSFWYAHHLLVI 517
Hs-Nox5        -------SPTGVALLLLLLLMFICSSSCIRR-------------SGHFEVFYWTHLSYLL 379
Cf-Nox5        -------SPTGVALLLLLLLMFACSSSCIRRS-------------GHFEVFYWTHLSYLP 406
Bt-Nox5        -------SPTGVALLLLLLLMFACSSSCVRRS-------------GHFEVFYWTHLSYLP 414
Md-Nox5        -------SLTGIVLQLLILIMLVCSCSFVRRS-------------GHFEVFYWTHLLYIS 386
Xt-Nox5        -------SITGILLQLLICLMLLFSNTFVRKG-------------GYFEVFYWTHLSYIW 396
Tr-Nox5        -------SVTGVVLQFMICLMVLCSSTFVRRS-------------GHFEIFYWSHLSYVW 320
Tn-Nox5        -------SLTGVVLQLLIGLMVLCSSTFVRAH-------------GHFEVFYWSHLSYVW 198
Gg-Nox5        -------PQTGLALQLLLFAMLAFSSPCVRRG-------------GHFELFYWSHLSYVP 369
Ol-Nox5        -------SLTGVVLLLLICLMVLCSSTFVRR-------------SGHFEVFYWSHLSYIW 393
Dr-Nox5        -------SITGVVIQILIGLMVVCSSTFVRR-------------SGHFEVFYWSHLSYIW 276
Sp-Nox5A       -------FLTGWVLDIILAIMVICSMPFVRR-------------SGHFQVFYFTHMLYVV 387
Sp-Nox5B       -------IITGILIIIVLIIMTICSLPFIRR-------------NGYFKVFYWTHQLCIV 352
Ag-Nox5        -------NPTGVVLVLILLIMFICSQPFVRR-------------GGSFEVFYWTHLLYVP 438
Am-Nox5        -------NPTGFILVILFLIIMICSMPFVRR-------------GGCFEIFYWSHLLYIP 440
Dm-Nox5        -------NPTGVALLAILVVMFVCSQPFVRR-------------KGSFEVFYWTHLLYVP 443
Dd-NoxC        -------GVTGFIMISFLIIMAILSLKIIRK--------------SNFELFYYSHHLFIG 760
Mg-NoxC        -------GITGLTALGLFYTLALLSMPQVRR--------------WNYEVFQLAHLLMFP 403
Fg-NoxC        -------GFTGITALGLFWILCLLSIPQVRR--------------WNYEVFQLGHLLMFP 328
Cc-NoxD        ---------ITGFLLAIVFGTMLVLARPSVRK--------------NNFRLFYAVHIIGAT 192
Py-NoxD        ---------ITGSMLVALFAILFVTSVRVNRS--------------KRYELFWYSHAICAS 187
Xt-Duox1       -------GMTGVLLLAVMALMYVFSCYHFRR--------------VSFRCFWLTHHLYVV 1184
Xt-Duox2       -------GLTGVLLLAVLALMYVFSSHHFRR--------------ISFRGFWVTHHFYIL 1236
Gg-Duox        -------GMTGVLLLIILAVMYVFATHHFRR--------------VSFQAFWITHHLYVL 1122
Tr-Duox        -------GLTGILLLFTLAFMYVFASRYFRR--------------ISFRGFWFTHCLYVV 1154
Tn-Duox        ILVLLLLGLTGILLLFTLAFMYVFASRYFRR--------------ISFRGFWLTHCLYVL 1305
Ol-Duox        -------GITGVLLLLVLAFMYVFASRYFRH--------------ISFRAFWITHYLYIV 1231
Dr-Duox        -------GITGVILLLIFAFMYVFASHYFRR--------------ISFRGFWITHHLYVL 1182
Mm-Duox2       -------GMTGVLLLLVLAIMYVFASHHFRR--------------HSFRGFWLTHHLYVV 1196
Rn-Duox2       -------GMTGVLLLLVLAIMYVFASHHFRR--------------HSFRGFWLTHHLYVV 1196
Hs-Duox2       -------GMTGVLLLLVLAIMYVFASHHFRR--------------RSFRGFWLTHHLYIL 1227
Cf-Duox2       -------GMTGVLLLLVLAIMYVFASHHFRR--------------RSFRGFWLTHHLYIV 979
Hs-Duox1       -------GLTGVVLLLILAIMYVFASHHFRR--------------RSFRGFWLTHHLYIL 1230
```

```
Cf-Duox1        -------GLTGVMLLLVLAIMYVFASHHFRR--------------HSFRGFWLTHHLYIL 1231
Rn-Duox1        -------GLTGVLLLLALAIMYVFASHHFRR--------------RSFRGFWLTHHLYIF 1226
Mm-Duox1        -------GLTGVLLLLALAIMYVFASHHFRR--------------RSFRGFWLTHHLYIF 1230
Ci-DuoxA        -------GITGVLLTLIVIVMYTFASDYSRR--------------RVFQWFWWTHNFGYI 1254
Ci-DuoxC        -------GITGVILTLALIVMYVFASNYARR--------------MIFNWFRWTHKLGYL 1119
Ci-DuoxD        -------GNTGVLLTIIFIVMYVFSLDYPRQ--------------VMFNWFQWIHFFGYI 1197
Sp-Duox         -------GFTGILLVMVCTVIYTFAFQYARR--------------RVFNLFWFTHNM-WI 1350
Dm-Duox         -------GTTGVMLFIIMCIIFVFAHPTIRK--------------KAYNFFWNMHTL-YI 1150
Ag-Duox         -------GVTGVMLFVTMCIIFAFAHPTIRK--------------KAYKFFWNAHSL-YV 1151
Am-Duox         -------GLTGILLFIVMTIIFVFAHPTIRQ--------------KAYKFFWSTHSL-YV 1156
Ce-Duox1        -------GLTGIALVAVMCIIYVFALPCFIK--------------RAYHAFRLTHLL-NI 1170
Ce-Duox2        -------GLTGIGLVIVMSIIYVFALPKFTR--------------RAYHAFRLTHLL-NI 1176
Ci-DuoxB        -------GLTGVLLLLTISIIYVFAMQYSRR--------------FCFRAFWITHHL-YT 1173
                         **                   *                    *   *


                ____TM(V)______
Hs-Nox1         Y-ILGLGIHGIGGIVRGQTEESMNESHPRKCAESF-EMWDD------------------- 252
Cf-Nox1         Y-FIGLGIHGIGGIVRGQTEESLNESHPHRCAESF-KQWDD------------------- 252
Mm-Nox1         Y-IICLGIHGLGGIVRGQTEESLGESHPHNCSHSF-HEWDD------------------- 251
Rn-Nox1         Y-IICLGIHGLGGIVRGQTEESMSESHPRNCSYSF-HEWDK------------------- 251
Gg-Nox1         Y-FIGLVIHGVAGLVRGQTEESMKEVHPQSCAEFL-VNKSKE------------------ 251
Xt-Nox1         F-FIGLVFHGAGRIVRGQTSDSMETNNYEKCHNSF-TQWQN------------------- 248
Tn-Nox1         F-FAGLVFHGAGRIVRSQ-QTTNPPHNTSYCKDHP-DDWGH------------------- 249
Tr-Nox1         F-FAGLVFHGAGRIVRSQ-NSE-PAHDATFCKDRT-EDWGK------------------- 247
Ol-Nox1         F-FVGLVFHGYGRIVRSQ-FNT-DDHNATFCKDRP-DDWGK------------------- 252
Dr-Nox1         F-FAGLVFHGAGRVVRGQ-VTTDPPHNNSFCEDQP-DNWGK------------------- 251
Mm-Nox2         F-FIGLAIHGAERIVRGQTAESLEEHNLDICADKI-EEWG-------------------- 252
Rn-Nox2         F-FIGLAIHGAERIVRGQTSDSLKEHNLDVCADKI-KEWG-------------------- 252
Hs-Nox2         F-FIGLAIHGAERIVRGQTAESLAVHNITVCEQKI-SEWG-------------------- 252
Cf-Nox2         F-FIGLAIHGAERIVRGQTAASRLEHNYKVCADNI-SQWG-------------------- 252
Gg-Nox2         F-FIGLVIHGAGRIVRGQTAVSLAEHIPEVCSKNF-TDWG-------------------- 252
Xt-Nox2         F-FIGLVIHGAGKIVRGQTDKSLEKHNSTECEDKF-TEWG-------------------- 252
Tn-Nox2         F-FIGLVLHGFGRIVRGQTAASLKTNKPEVCADRF-EEWGR------------------- 250
Tr-Nox2         F-FIGLVLHGFGRIVRGQTPASLKSNDPTVCADQF-EDWGR------------------- 250
Ol-Nox2         F-FIGLVFHGFGRIVRGQTSRSLDSNDPDVCADRF-EDWGK------------------- 251
Dr-Nox2         F-FIGLVLHGIGRIVRGQTDADLQVHDPTICHSKF-EKWGQ------------------- 250
Hs-Nox3         F-FLSLAIHGTGRIVRGQTQDSLSLHNITFCRDRY-AEWQT------------------- 251
Cf-Nox3         F-FIGLAIHGAGRIVRGQTPESQLLHNVTFCRDHH-AQWQK------------------- 251
Mm-Nox3         F-FISLAIHGGGRIIRGQTPESLRLHNVTYCRDHY-AEWQA------------------- 251
Rn-Nox3         L-FISLAIHGGGRIIRGQTPESLRLHNVTFCRDHF-DEWQE------------------- 251
Gg-Nox3         F-FIGLIIHGTGQLVRGQTPHSLLLHNITYCKEHY-LEWEK------------------- 250
Ci-Nox2         F-YGFLVVHGISMQVRGQTPQSLTVHDPIRCSTIDPATWSQ------------------- 265
Sp-Nox2A        Y-FAMLLAHGVGGIIRSQ--TNLDRHDVVFCSENL-DVWGP------------------- 263
Sp-Nox2B        Y-YAMLMTHGMGGVVKYQ--TNVDEHDPVECMVDE-ETFDQ------------------- 238
Mm-Nox4         F-YMLLLLHVSGGLLKYQTNVDTHPPGCISLNQTSSQNMSIPDYVSEHF----------- 247
Rn-Nox4         F-YMLLLLHVSGGLLKYQTNLDTHPPGCISLNRTPSQNMSIADYVSEHF----------- 247
Hs-Nox4         F-YMLLTLHVSGGLLKYQTNLDTHPPGCISLNRTSSQNISLPEYFSEHF----------- 247
Cf-Nox4         F-YMLLMLHVSGGLLKYQANLDTHPPGCININGTRYQNIHLPNYRSEHF----------- 251
Gg-Nox4         F-YILLMLHVSGGVLKYQTNLEEHPPGCFNPNKTLLGNMTVPKSFEELFPDY-------- 210
Xt-Nox4         F-YLLLLLHACAGVLKYQSNLEEHPPGCLYLNRSAQGEVPGAAADGGEFPGR-------- 238
Ol-Nox4         F-YIILMVHMAGGALKFQTNIEAHPPGCLRASQS-------------------------- 232
Tr-Nox4         F-YIVLMVHMVGGVLKYQTNIEAHPPGCLTANQSNM------------------------ 232
Ci-Nox4         F-YALIFIHAMDGVIKYQTNVDQHKPGCFILIDQTNNTSNISMVQPEPEPFPNHPSMVKP 256
Ag-Nox          F-YGMMFYHPLRLVHGGSTLNDAIDRFNIIKHQTNVDK---------------------- 218
```

```
Pa-NoxA         F-FLGLYTHTVGCFVRDTADAISPFAGDEYWEHCI------------------------- 216
Mg-NoxA         F-FLGLYTHTVGCFVRDTVEPHSPFAGDEYWNHCI------------------------- 215
Fg-NoxA         F-FLGLYTHTVGCFVRDTPEAFSPFAGDEFWEHCI------------------------- 219
An-NoxA         F-LLGLYTHATGCFVRDSAEPYSPFAGERFWKHCI------------------------- 214
Pa-NoxB         F-FFFWSIHGAFCMIQPDFAPFCISIG----TQAI------------------------- 261
Fg-NoxB         F-FFFWSIHGAFCMIQPDVAPFCTSIG----SSAI------------------------- 250
Mg-NoxB         F-FFFWSIHGAFCMIQPDFAPFCMSFG----TSAI------------------------- 265
Dd-NoxA         F-FGLLVVHGLHSILEP-TSF--------------------------------------- 214
Dd-NoxB         F-YILLCFHGYSKILKKDPQS--------------------------------------- 389
At-rbohC        V-YILLVAHGYYLYLTRD------------------------------------------ 560
At-rbohG        V-YILLVLHGYYIYLNKE------------------------------------------ 513
At-rbohA        V-YSLLVVHGFYVYLIIEP----------------------------------------- 552
At-rbohB        V-YVLLIVHGYFVYLSKE------------------------------------------ 506
At-rbohD        V-YALLIVHGIKLYLTKI------------------------------------------ 583
At-rbohF        V-YILLILHGIFLYFAKP------------------------------------------ 594
At-rbohI        V-YVLLVIHGVSLYLEHK------------------------------------------ 582
At-rbohE        V-YIMLIVHGTFLFFADK------------------------------------------ 572
At-rbohH        A-YILLIIHGYYLIIEKP------------------------------------------ 523
At-rbohJ        A-YALLIIHGYILIIEKP------------------------------------------ 534
Hs-Nox5         V-WLLLIFHG-------------------------------------------------- 388
Cf-Nox5         M-WILLILHGPN------------------------------------------------ 417
Bt-Nox5         M-WLLLILHGPN------------------------------------------------ 425
Md-Nox5         I-WALLIVHGPN------------------------------------------------ 397
Xt-Nox5         I-WILLFLHTPK------------------------------------------------ 407
Tr-Nox5         V-WILLMVHCAN------------------------------------------------ 331
Tn-Nox5         V-WALLMVHCAN------------------------------------------------ 209
Gg-Nox5         V-WALLLFHAPN------------------------------------------------ 380
Ol-Nox5         V-LILLIVHC-------------------------------------------------- 402
Dr-Nox5         V-SALLVVHC-------------------------------------------------- 285
Sp-Nox5A        F-WGLLLIHG-------------------------------------------------- 396
Sp-Nox5B        F-WCLIIIHS-------------------------------------------------- 361
Ag-Nox5         F-WILVLFHG-------------------------------------------------- 447
Am-Nox5         Y-WILVILHA-------------------------------------------------- 449
Dm-Nox5         F-WILCLFHG-------------------------------------------------- 452
Dd-NoxC         F-YVLLILHGTMGWIRPPTF---------------------------------------- 779
Mg-NoxC         I-IGLLAAHGTAQLLQYAMFGY-------------------------------------- 424
Fg-NoxC         I-IGLMMAHGTAALLQWPMFGY-------------------------------------- 349
Cc-NoxD         LFFGLLIIHGMFRQVPYTYK---------------------------------------- 212
Py-NoxD         LGFVLLMIHGLHYGVYWTYR---------------------------------------- 207
Xt-Duox1        F-YILTIIHGSFALIQQP------------------------------------------ 1201
Xt-Duox2        L-YILTILHGSFGLLQQP------------------------------------------ 1253
Gg-Duox         L-YVLVIIHGSYALIQQP------------------------------------------ 1139
Tr-Duox         V-YALTVIHGSYALIQEP------------------------------------------ 1171
Tn-Duox         V-YALTVVHGSYAFIQEP------------------------------------------ 1322
Ol-Duox         V-YILTVIHGSFALLQEP------------------------------------------ 1248
Dr-Duox         I-YVLTVVHGSYGLLQQP------------------------------------------ 1199
Mm-Duox2        L-YVLIIIHGSYALIQLP------------------------------------------ 1213
Rn-Duox2        L-YALIIIHGSYALIQLP------------------------------------------ 1213
Hs-Duox2        L-YALLIIHGSYALIQLP------------------------------------------ 1244
Cf-Duox2        L-YVLLIIHGSFGLIQLP------------------------------------------ 996
Hs-Duox1        L-YVLLIIHGSFALIQLP------------------------------------------ 1247
Cf-Duox1        L-YVLLIIHGSFGLIQLP------------------------------------------ 1248
Rn-Duox1        L-YILLIIHGSFALIQMP------------------------------------------ 1243
Mm-Duox1        L-YILLIIHGSFALIQMP------------------------------------------ 1247
Ci-DuoxA        FLFFFMILHGSGFLVQDP------------------------------------------ 1272
```

```
Ci-DuoxC            SLYFFSFVHGSGMLISSP------------------------------------------ 1137
Ci-DuoxD            SVYFFTVLHGSGMLIQIP------------------------------------------ 1215
Sp-Duox             IYFILMFLHGSGRLVQPP------------------------------------------ 1368
Dm-Duox             GLYLLSLIHGLARLTGPP------------------------------------------ 1168
Ag-Duox             VLYALCLVHGLARLTGAP------------------------------------------ 1169
Am-Duox             VLYALCLIHGLARLTGSP------------------------------------------ 1174
Ci-DuoxB            VLYILTILHGSLGIVQAP------------------------------------------ 1191
Ce-Duox1            AFYALTLLHGLPKLLDSP------------------------------------------ 1188
Ce-Duox2            GFYALTILHGLPSLFGSP------------------------------------------ 1194
                            *


Hs-Nox1             ------------------------------------------------------------
Cf-Nox1             ------------------------------------------------------------
Mm-Nox1             ------------------------------------------------------------
Rn-Nox1             ------------------------------------------------------------
Gg-Nox1             ------------------------------------------------------------
Xt-Nox1             ------------------------------------------------------------
Tn-Nox1             ------------------------------------------------------------
Tr-Nox1             ------------------------------------------------------------
Ol-Nox1             ------------------------------------------------------------
Dr-Nox1             ------------------------------------------------------------
Mm-Nox2             ------------------------------------------------------------
Rn-Nox2             ------------------------------------------------------------
Hs-Nox2             ------------------------------------------------------------
Cf-Nox2             ------------------------------------------------------------
Gg-Nox2             ------------------------------------------------------------
Xt-Nox2             ------------------------------------------------------------
Tn-Nox2             ------------------------------------------------------------
Tr-Nox2             ------------------------------------------------------------
Ol-Nox2             ------------------------------------------------------------
Dr-Nox2             ------------------------------------------------------------
Hs-Nox3             ------------------------------------------------------------
Cf-Nox3             ------------------------------------------------------------
Mm-Nox3             ------------------------------------------------------------
Rn-Nox3             ------------------------------------------------------------
Gg-Nox3             ------------------------------------------------------------
Ci-Nox2             ------------------------------------------------------------
Sp-Nox2A            ------------------------------------------------------------
Sp-Nox2B            ------------------------------------------------------------
Mm-Nox4             ---------------HGSLPRGFSKLEDR-YQKTLVKI---------------------- 269
Rn-Nox4             ---------------HGSLPGGFSKLEDH-YQKTLVKI---------------------- 269
Hs-Nox4             ---------------HEPFPEGFSKPAEF-TQHKFVKI---------------------- 269
Cf-Nox4             ---------------HESFPGGLSKPDEL-TQNRSVNI---------------------- 273
Gg-Nox4             --------------TTEPFPEDLTFPQPL-VQSNFMRI---------------------- 233
Xt-Nox4             --------------AARALMGSFFSHEDMSVHNNSEKI---------------------- 262
Ol-Nox4             ----------------------LRQQQQAEDLD-QKQR---------------------- 247
Tr-Nox4             ----------------------DPQAKEMEKADNEERR---------------------- 248
Ci-Nox4             KMVAVPEPPHREPFPNMKLASKMPSKTDMKFMNNTAPQPHHVIMPEPEPMPHKGAHGAPN 316
Ag-Nox              ----------------HKIMCDLVDNVTLHSNEDLLVL---------------------- 240
Pa-NoxA             ------------------------------------------------------------
Mg-NoxA             ------------------------------------------------------------
Fg-NoxA             ------------------------------------------------------------
An-NoxA             ------------------------------------------------------------
```

```
Pa-NoxB          ------------------------------------------------------------
Fg-NoxB          ------------------------------------------------------------
Mg-NoxB          ------------------------------------------------------------
Dd-NoxA          ------------------------------------------------------------
Dd-NoxB          ------------------------------------------------------------
At-rbohC         ------------------------------------------------------------
At-rbohG         ------------------------------------------------------------
At-rbohA         ------------------------------------------------------------
At-rbohB         ------------------------------------------------------------
At-rbohD         ------------------------------------------------------------
At-rbohF         ------------------------------------------------------------
At-rbohI         ------------------------------------------------------------
At-rbohE         ------------------------------------------------------------
At-rbohH         ------------------------------------------------------------
At-rbohJ         ------------------------------------------------------------
Hs-Nox5          ------------------------------------------------------------
Cf-Nox5          ------------------------------------------------------------
Bt-Nox5          ------------------------------------------------------------
Md-Nox5          ------------------------------------------------------------
Xt-Nox5          ------------------------------------------------------------
Tr-Nox5          ------------------------------------------------------------
Tn-Nox5          ------------------------------------------------------------
Gg-Nox5          ------------------------------------------------------------
Ol-Nox5          ------------------------------------------------------------
Dr-Nox5          ------------------------------------------------------------
Sp-Nox5A         ------------------------------------------------------------
Sp-Nox5B         ------------------------------------------------------------
Ag-Nox5          ------------------------------------------------------------
Am-Nox5          ------------------------------------------------------------
Dm-Nox5          ------------------------------------------------------------
Dd-NoxC          ------------------------------------------------------------
Mg-NoxC          ------------------------------------------------------------
Fg-NoxC          ------------------------------------------------------------
Cc-NoxD          ------------------------------------------------------------
Py-NoxD          ------------------------------------------------------------
Xt-Duox1         ------------------------------------------------------------
Xt-Duox2         ------------------------------------------------------------
Gg-Duox          ------------------------------------------------------------
Tr-Duox          ------------------------------------------------------------
Tn-Duox          ------------------------------------------------------------
Ol-Duox          ------------------------------------------------------------
Dr-Duox          ------------------------------------------------------------
Mm-Duox2         ------------------------------------------------------------
Rn-Duox2         ------------------------------------------------------------
Hs-Duox2         ------------------------------------------------------------
Cf-Duox2         ------------------------------------------------------------
Hs-Duox1         ------------------------------------------------------------
Cf-Duox1         ------------------------------------------------------------
Rn-Duox1         ------------------------------------------------------------
Mm-Duox1         ------------------------------------------------------------
Ci-DuoxA         ------------------------------------------------------------
Ci-DuoxC         ------------------------------------------------------------
Ci-DuoxD         ------------------------------------------------------------
Sp-Duox          ------------------------------------------------------------
Dm-Duox          ------------------------------------------------------------
```

```
Ag-Duox          ------------------------------------------------------------
Am-Duox          ------------------------------------------------------------
Ci-DuoxB         ------------------------------------------------------------
Ce-Duox1         ------------------------------------------------------------
Ce-Duox2         ------------------------------------------------------------


                                              ________TM(VI)________
Hs-Nox1          -------------RDSHCRRPKFEGHPPESWKWI-LA-PVILYICERILRFYRSQQKVVI 297
Cf-Nox1          -------------HDSHCKHPRFEGLPAESWKWI-LA-PGVLYILERILRFYRSQQKVVI 297
Mm-Nox1          -------------HKGSCRHPHFAGHPPESWKWI-LA-PIAFYIFERILRFYRSQQKVVI 296
Rn-Nox1          -------------YERSCRSPHFVGQPPESWKWI-LA-PIAFYIFERILRFYRSRQKVVI 296
Gg-Nox1          ------------CRHQCCKEPEFGSIPAEVGMGVGLQ-ESRLLHTPGCCXSSHTGTELPA 298
Xt-Nox1          ------------SKSSNRDDDDHHNDKCTVPAFQGNE-PGVRYKLKMYFETRQKRKTHAI 295
Tn-Nox1          --------------IPECPIPQFAGGFPQTWMWV-IA-PMFLYVCERLIRFVRYMQTVRY 293
Tr-Nox1          --------------IPECPIPQFSGGFPQTWMYV-IG-PMVLYLCERLIRFIRYMQTVRY 291
Ol-Nox1          --------------IPECPIPQFKGGEPMTWKWV-IG-PMIIYVCERVLRFIRYMQAVQY 296
Dr-Nox1          --------------IPECPIPQFAGGSPQTWMYV-IG-PMIIYICERLLRFIRYMQPVTY 295
Mm-Nox2          -------------KIKECPVPKFAGNPPMTWKWI-VG-PMFLYLCERLVRFWRSQQKVVI 297
Rn-Nox2          -------------KIKECPVPKFAGNPPMTWKWI-VG-PMFLYLCERLVRFWRSQQKVVI 297
Hs-Nox2          -------------KIKECPIPQFAGNPPMTWKWI-VG-PMFLYLCERLVRFWRSQQKVVI 297
Cf-Nox2          -------------KIPDCPIPQFSGNPPMTWKWI-VG-PMFLYLCERLVRFWRSQQKVVI 297
Gg-Nox2          -------------KKGACPVPQFAGNPPMTWKWV-VG-PMFLYFCERLVRFWRSQQKVVI 297
Xt-Nox2          -------------NITSCPIPQFAGNEPMTWKWV-VA-PMVLYVFERLVRFWRSQQKVVI 297
Tn-Nox2          -------------NGSDCAVPEFAGNPPMTWKWV-VG-PMILYVCERIVRFYRSHQKVVI 295
Tr-Nox2          -------------NGSNCAVPEFAGNPPMTWKWV-VG-PMILYVCERLVRFYRSHQKVVI 295
Ol-Nox2          -------------NESGCAVPAFAGNPPMTWKWV-VG-PMFLYVCERLVRIYRSHQKVVI 296
Dr-Nox2          -------------NVTDCPVPIFAGNPPKTWKWV-VG-PMFLYVCERLVRFYRSQQKVVI 295
Hs-Nox3          --------------VAQCPVPQFSGKEPSAWKWI-LG-PVVLYACERIIRFWRFQQEVVI 295
Cf-Nox3          --------------MAQCPMPQFSGKEPSAWKWV-LG-PVVLYACERIIRFWRFQQEVVI 295
Mm-Nox3          --------------AALCPVPQFSGKEPSAWKWA-LG-PVVLYACERIIRFWRSHQEVVI 295
Rn-Nox3          --------------AASCPVPQFSGKEPSAWKWT-LG-PVVLYACEIIIRFWRSHQEVVI 295
Gg-Nox3          --------------ATQCPLPQFSGNKPVAWKWV-VS-PVVLYICERIVRFWRFQQEVVI 294
Ci-Nox2          ---------------NNCPTPVFAGSPPMTWKWV-IA-PMVLYVIERIIRLVRFNQQVEV 308
Sp-Nox2A         -------------TSAQCEDPVFKDGSAASYKWV-SG-PLFIYLLERTIRFWRSCQTVTL 308
Sp-Nox2B         -------------CVID-NPPLFAGTPGASWKWC-VT-PLCVYFLERILRMIRTWPDVTI 282
Mm-Nox4          ---------------CLEEP-KFQAHFPQTWIWISGP--LCLYCAERLYRCIRSNKPVTI 311
Rn-Nox4          ---------------CLEEP-KFQAHFPQTWIWISGP--LCLYCAERLYRCIRSNKPVTI 311
Hs-Nox4          ---------------CMEEP-RFQANFPQTWLWISGP--LCLYCAERLYRYIRSNKPVTI 311
Cf-Nox4          ---------------CMEEP-RFQANFPQLPMWELTPCTLCLYCDT-CFTSVKLNAPKVI 316
Gg-Nox4          ---------------CSKEP-KFQSHFPETWFWISGP--LCLYCVERLYRYIRSNKPVTI 275
Xt-Nox4          ---------------CTKGP-TFRPHFPETWLWISGP--LCLYCAERLYRYIRSSKPVTI 304
Ol-Nox4          ---------------CKEDA-HFQPHYPQTWLWVSGP--LCLYCVERFYRYIRSSHPVTI 289
Tr-Nox4          ---------------CTEEA-HFQGHYPQTWIWLSGP--LCLYCAERFFRYIRSCDPVTI 290
Ci-Nox4          TTRIFVHGSWVEVMECVQPPPKFSSCRQEAWLWLCAP--LIIYVIERIGRHFRSSHDVTT 374
Ag-Nox           ---------------CEEPP-QFSAGTKRAWIWPLVG--LAIYLADISFRYLTSHSERYR 282
Pa-NoxA          --------------------------GYLGWRWELWT-GG-FYLIERLYREIRAIRETKI 248
Mg-NoxA          --------------------------GYLGWRWELWT-GG-FYLLERLYREIRARRETKI 247
Fg-NoxA          --------------------------GYLGWRWELWT-GG-AYLLERLWREVRARRSTKI 251
An-NoxA          --------------------------GYQGWRWELVA-GF-FYLCERLWREIRALRETEI 246
Pa-NoxB          --------------------------GVF-WQYWMYG-GF-AYLAERVAREIRGRHKTYI 292
Fg-NoxB          --------------------------GVF-WQFWMYS-GF-CYLAERIAREVRGRHRTFI 281
Mg-NoxB          --------------------------GVF-WQFWMYG-GF-VYMAERIAREIRGKHKTYI 296
Dd-NoxA          ------------------------------WKWVIGP-CA-LYIVERLIRLLRSKKTTML 242
```

```
Dd-NoxB      ------------------------------WMWIIAP-FI-LYSIERLIRIARSKKRVIL 417
At-rbohC     ------------------------WHNKTTWMYLVVP--VVLYACERLIRAFRSSI---K 591
At-rbohG     ------------------------WYKKTTWMYLAVP--VALYAYERLIRAFRSSI---R 544
At-rbohA     ------------------------WYKKTTWMYLMVP--VVLYLCERLIRAFRSSV---E 583
At-rbohB     ------------------------WYHKTTWMYLAVP--VLLYAFERLIRAFRPGA---K 537
At-rbohD     ------------------------WYQKTTWMYLAVP--ILLYASERLLRAFRSSI---K 614
At-rbohF     ------------------------WYVRTTWMYLAVP--VLLYGGERTLRYFRSGS---Y 625
At-rbohI     ------------------------WYRKTVWMYLAVP--VLLYVGERIFRFFRSRL---Y 613
At-rbohE     ------------------------WYQKTTWMYISVP--LVLYVAERSLRACRSKH---Y 603
At-rbohH     ------------------------WYQKTTWMYLAVP--MLFYASERLFSRLLQEHS--H 555
At-rbohJ     ------------------------WYQKTTWMYVAIP--MVLYASERLFSR-VQEHN--H 565
Hs-Nox5      ---------------------------PNFWKWLLVP--GILFFLEKAIG---LAVSRMA 416
Cf-Nox5      -----------------------------FWKWLLVP--GTLFFLEKIIGLAVSRMAALC 446
Bt-Nox5      -----------------------------FWKWLLVP--GTLFFLEKTISLAASRMAALH 454
Md-Nox5      -----------------------------FWKWLLVP--GLLFFLEKVVGLVLSRMAALS 426
Xt-Nox5      -----------------------------FWKWFLVP--GLLFLLEKLFGAAVSRTGDVY 436
Tr-Nox5      -----------------------------FWKWFVAP--GFVFLLEKIIGIAVSRMGGLY 360
Tn-Nox5      -----------------------------FWKWFVGP--GFVFLLEKIVGIAVSRMGGLH 238
Gg-Nox5      -----------------------------FWKWFLVP--GGLFVLEKAVGTAVSRAVGLR 409
Ol-Nox5      ---------------------------ANFWKWFVVP--GLLFLLEKIVG---IAVSRMG 430
Dr-Nox5      ---------------------------ANFWKWFVVP--GVAFLIEKLVG---IAVSRMG 313
Sp-Nox5A     ---------------------------PRFWYWFVVP--GIIFIVEKLSQTKCVKQARYG 427
Sp-Nox5B     ---------------------------KYFWIWFIAP--GIIYLAERLVRLQFFRRARFG 392
Ag-Nox5      ---------------------------PNFWKWFIVP--GLIYLVERTIR-LVWMRTEHG 477
Am-Nox5      ---------------------------PNFWKWFIGP--GLIYLLERIRR-IAWSRSQLG 479
Dm-Nox5      ---------------------------PNFWKWFLLP--GLVYIVERALR-FIWMRGEHG 482
Dd-NoxC      ------------------------------WKWFIVP-GF-FYTVDRSFRLFKRTHRVEV 807
Mg-NoxC      --------------------------------WLAVP--TILVLTERLVRVGTGFHRIPA 450
Fg-NoxC      --------------------------------FLAFP--TLLVLVERTVRVGLGFHRIKA 375
Cc-NoxD      --------------------------------WVIPP--LILYAIDRFLRR----RKVSA 234
Py-NoxD      --------------------------------WAAGP--MAVYIIDRLMRR----VEQKE 229
Xt-Duox1     ----------------------------RFHIFFIVP--ALIYSADKLISLSRKKIQINV 1231
Xt-Duox2     ----------------------------KFHVFLMAP--ALIFIRDSLISLRRKKTEINV 1283
Gg-Duox      ----------------------------RFHIYFIIP--ALIYGADKLLSLSRKKVEISV 1169
Tr-Duox      ----------------------------RFHIYLIPP--ALLFLLDKLISLSRKKLEIPV 1201
Tn-Duox      ----------------------------RFHIYLIPP--ALLFLLDKLISLSRKKLEIPV 1352
Ol-Duox      ----------------------------RFYIYLIPP--SLLFLLDKLISLSRKKVEIPV 1278
Dr-Duox      ----------------------------RFHIYLIPP--GLLFLLDKLISLSRKKVEIPV 1229
Mm-Duox2     ----------------------------SFHIYFLVP--AIIYGGDKLVSLSRKKVEISV 1243
Rn-Duox2     ----------------------------SFHIYFLVP--AIIYVGDKLVSLSRKKVEISV 1243
Hs-Duox2     ----------------------------TFHIYFLVP--AIIYGGDKLVSLSRKKVEISV 1274
Cf-Duox2     ----------------------------RFYIYFLVP--ALIYVGDKLVSLSRKKVEISV 1026
Hs-Duox1     ----------------------------RFHIFFLVP--AIIYGGDKLVSLSRKKVEISV 1277
Cf-Duox1     ----------------------------RFHIFFLVP--ALIYVGDKLVSLSRKKVEISV 1278
Rn-Duox1     ----------------------------RFHIFFLVP--AIIYVGDKLVSLSRKKVEISV 1273
Mm-Duox1     ----------------------------RFHIFFLVP--AIIYVGDKLVSLSRKKVEISV 1277
Ci-DuoxA     ----------------------------FFYYFFLGP--AILYTLDKLYSVSRSKCEISV 1302
Ci-DuoxC     ----------------------------QFYYYFLVP--GILFTLDKVYTYSRKKAYISV 1167
Ci-DuoxD     ----------------------------SFYYYFLVP--AILYTFDKLYSVYRKKFQLPV 1245
Sp-Duox      ----------------------------FTHYFALGP--IVLFTLDKLVSISRKKAEIAV 1398
Dm-Duox      ----------------------------RFWMFFLGP--GIVYTLDKIVSLRTKYMALDV 1198
Ag-Duox      ----------------------------RFWLFFIGP--GIVYTLDKIVSLRTKYMALDV 1199
Am-Duox      ----------------------------RFWIFFVGP--AIIYALDKVVSLRTKYMALDI 1204
Ci-DuoxB     ----------------------------VFHFYLVVP--VVIFLIDKMITISRSKVQITV 1221
Ce-Duox1     ----------------------------KFGYYVVGP--IVLFVIDRIIGLMQYYKKLEI 1218
```

```
Ce-Duox2            ----------------------------KFGYYVVGP--IVLFVIDRIIGLMQYYKSLDI 1224
                                                     **

Hs-Nox1             T---KVVMHPS---------------------------KVLELQMNKR-----GFSMEVG 322
Cf-Nox1             T---KVVMHPS---------------------------KVLELQMIKR-----GFSMEVG 322
Mm-Nox1             T---KVVMHPS---------------------------NVLELQMRKR-----GFSMEVG 321
Rn-Nox1             T---KVVMHPC---------------------------KVLELQMRKR-----GFTMGIG 321
Gg-Nox1             S---QVVMHPA---------------------------KVLELQMQKK-----GFRMEVG 323
Xt-Nox1             KPNHTTMGHPS---------------------------KVLEIQMQKR-----GFKMEVG 323
Tn-Nox1             R---RIVMRPS---------------------------KVLELQLVKS-----GFKMEVG 318
Tr-Nox1             R---KIVMRPS---------------------------KVLELQLMKR-----GFKMEVG 316
Ol-Nox1             R---KIVIHPS---------------------------KVLELQLRKK-----GFKMEVG 321
Dr-Nox1             R---KIVIRPS---------------------------KVLELQLVKP-----GFSMDVG 320
Mm-Nox2             T---KVVTHPF---------------------------KTIELQMKKK-----GFKMEVG 322
Rn-Nox2             T---KVVTHPF---------------------------KTIELQMKKK-----GFKMEVG 322
Hs-Nox2             T---KVVTHPF---------------------------KTIELQMKKK-----GFKMEVG 322
Cf-Nox2             T---KVVTHPF---------------------------KTIELQMKKK-----GFKMEVG 322
Gg-Nox2             T---KVVIHPF---------------------------KTIELQMMKK-----GFKMEVG 322
Xt-Nox2             T---KVVTHPF---------------------------KTIELQMKMK-----GFKMEVG 322
Tn-Nox2             T---KVVMHPS---------------------------KTLELRMKRK-----GFHMEVG 320
Tr-Nox2             T---KVVMHPS---------------------------KTLELQMKRK-----GFRMEVG 320
Ol-Nox2             T---KVVMHPS---------------------------KTLELQMKKK-----GFHMEVG 321
Dr-Nox2             T---KVVTHPS---------------------------KTLELQMKKK-----GFKMEVG 320
Hs-Nox3             T---KVVSHPS---------------------------GVLELHMKKR-----GFKMAPG 320
Cf-Nox3             T---KVVSHPS---------------------------GVLELHMKKR-----NFKMAPG 320
Mm-Nox3             T---KVVSHPS---------------------------AVLELHMKKR-----DFKMAPG 320
Rn-Nox3             T---KVVSHPS---------------------------AVLELHMKKR-----DFKMAPG 320
Gg-Nox3             T---KVVTHSS---------------------------GVLELHMKKH-----GFKMEAG 319
Ci-Nox2             L---KVIKHPS---------------------------RVLEIQMRKN-----GFFAEVG 333
Sp-Nox2A            T---KVVKHQS---------------------------KVIELQMKKK-----GFKMEAG 333
Sp-Nox2B            V---QVVQHQS---------------------------KVIELRMKKQ-----GFKMLPG 307
Mm-Nox4             IS---VINHPS---------------------------DVMELRMIKE-----NFKARPG 336
Rn-Nox4             IS---VINHPS---------------------------DVMELRMIKE-----NFKARPG 336
Hs-Nox4             IS---VMSHPS---------------------------DVMEIRMVKE-----NFKARPG 336
Cf-Nox4             VSN--MINHPS---------------------------DVMEIRMIKE-----NFKARPG 342
Gg-Nox4             TS---VISHPS---------------------------NVLEVRMIKD-----DFRARPG 300
Xt-Nox4             VA---VITHPC---------------------------DVVEIRMVKE-----KFSARPG 329
Ol-Nox4             VT---VIRHPC---------------------------DVVELRMLKK-----NFRARPG 314
Tr-Nox4             VT---VIRHPC---------------------------NVIELQMLKN-----KFAARPG 315
Ci-Nox4             IVK--FIEHPC---------------------------DVIELRLYRN-----GFSAKPG 400
Ag-Nox              VTT--VQTYAM---------------------------AGHAIHLRLQFCRKAMVKILPG 313
Pa-NoxA             T---RVVKHPY---------------------------DVVEIQFNKP-----SFKYKAG 273
Mg-NoxA             T---RVVRHPYGEFFFFLFFLLFGPSMVGFSVLIRDSADVVEIQFNKP-----SFKYKAG 299
Fg-NoxA             T---RVVRHPY---------------------------DVVEIQFNKP-----SFKYKAG 276
An-NoxA             V---KVVRHPY---------------------------DAMEIQFRKP-----GFKYKPG 271
Pa-NoxB             S---KVIQHPS---------------------------NVCEIQIKKE-----HTKTRAG 317
Fg-NoxB             S---KVIQHPS---------------------------NVCEIQMKKE-----HTKTRAG 306
Mg-NoxB             S---KVIQHPS---------------------------NVCEIQIKKE-----HTKTRAG 321
Dd-NoxA             I---QARIHPS---------------------------RVIEVRMKTER-----FKYKPG 267
Dd-NoxB             E---KAIMHPS---------------------------KVLELRMKRDN---DNFNFKPG 444
At-rbohC            AVTIRKVAVYPG--------------------------NVLAIHLSRPQ----NFKYKSG 621
At-rbohG            TVKVLKMAAYPG--------------------------KVLTLQMSKPT----NFKYMSG 574
At-rbohA            AVSVLKVAVLPG--------------------------NVLSLHLSRPS----NFRYKSG 613
```

```
At-rbohB            AVKVLKVAVYPG--------------------------NVLSLYMSKPK----GFKYTSG 567
At-rbohD            PVKMIKVAVYPG--------------------------NVLSLHMTKPQ----GFKYKSG 644
At-rbohF            SVRLLKVAIYPG--------------------------NVLTLQMSKPT----QFRYKSG 655
At-rbohI            TVEICKVVIYPG--------------------------NVVVLRMSKPT----SFDYKSG 643
At-rbohE            SVKILKVSMLPG--------------------------EVLSLIMSKPP----GFKYKSG 633
At-rbohH            RVNVIKAIVYSG--------------------------NVLALYVTKPP----GFKYKSG 585
At-rbohJ            RVHIIKAIVYSG--------------------------NVLALYMTKPQ----GFKYKSG 595
Hs-Nox5             AVCIMEVNLLPS--------------------------KVTHLLIKRPP----FFHYRPG 446
Cf-Nox5             IVEVNLLPSKV-----------------------------THLLIKRPP----LFHYRPG 473
Bt-Nox5             IVEVNLLPSKV-----------------------------THLLIKRPP----LFHYRPG 481
Md-Nox5             IVEVNLLSSKV-----------------------------THLVISRPP----FFRYKPG 453
Xt-Nox5             ITEVNLLASKV-----------------------------THLVIKRPP----SFQFKPG 463
Tr-Nox5             IVEVNLLPSKV-----------------------------THLVIKRPQ----FFHFKPG 387
Tn-Nox5             IVEVNLLPSKVRWTHGWPVASHVVPSAMAGRCVCVRP-QVTHLVVRRPQ----FFHFKPE 293
Gg-Nox5             IVEVHLLPSQV-----------------------------THLVIQRPR----SFRFEPG 436
Ol-Nox5             GLYIVEVNLLPS--------------------------KVTHLVIKRPQ----FFHFKPG 460
Dr-Nox5             GLYIVEVNLLPS--------------------------KVTHLVIKRPP----FFQFKPG 343
Sp-Nox5A            KTYVQEVNLLPS--------------------------GVTHLALTRPN----RFHYKAG 457
Sp-Nox5B            KVYIQKGYVLPA--------------------------NVVQLVIQRPA----KFKFHAG 422
Ag-Nox5             KTYISSGLLLPS--------------------------KVTHLVIKRPL----HFCFRPG 507
Am-Nox5             KTYISSGLLLPS--------------------------KVTHLVIKRPP----HFVFHPG 509
Dm-Nox5             KTYISSGLLLPS--------------------------KVVHLVIKRPH----HFNFRPG 512
Dd-NoxC             L---DYCLKNE---------------------------RVINLTFSKPP----SFDYKPG 833
Mg-NoxC             SLKVLDDETVELR---------------------------ATIPSERI------WKYQAG 477
Fg-NoxC             TMKVLDKETVEVT---------------------------AIIPSERL------WKYKAG 402
Cc-NoxD             VELFLSAENAVLKDG-----------------------DILELRVPKA------FSYQAG 265
Py-NoxD             VRMEVSRDVGAIKGN-----------------------SMLCLRLPRS------FTYEPG 260
Xt-Duox1            LDIQRLPS------------------------------DVIHLEFQRPN----DFDYKSG 1257
Xt-Duox2            MKADLLPS------------------------------DVTCLRFQRPA----DFDYKSG 1309
Gg-Duox             VKAELLPS------------------------------GVTHLRFQRPQ----DFDYKSG 1195
Tr-Duox             VRAELLPS------------------------------GVTHLEFKRPQ----GFVYRSG 1227
Tn-Duox             VRAELLPS------------------------------GVTHLEIKRPQ----GFVYRSG 1378
Ol-Duox             VRAELLPS------------------------------GVTHLEFKRPS----GFVYRSG 1304
Dr-Duox             LKAELLLS------------------------------DVTMLEFKRPQ----GFVYRSG 1255
Mm-Duox2            VKAELLPSG------------------------------VTYLQFQRPK----TFEYKSG 1269
Rn-Duox2            VKAELLPSG------------------------------VTYLQFQRPK----TFEYKSG 1269
Hs-Duox2            VKAELLPSG------------------------------VTYLQFQRPQ----GFEYKSG 1300
Cf-Duox2            VKAELLPSG------------------------------VTHLQFQRPQ----GFEYKSG 1052
Hs-Duox1            VKAELLPSG------------------------------VTHLRFQRPQ----GFEYKSG 1303
Cf-Duox1            VKAELLPSG------------------------------VTHLQFQRPQ----GFEYKSG 1304
Rn-Duox1            VKAELLPSG------------------------------VTHLRFQRPQ----GFEYKSG 1299
Mm-Duox1            VKAELLPSG------------------------------VTHLRFQRPQ----GFEYKSG 1303
Ci-DuoxA            VNAELLPS------------------------------EVTHLEFKRPV----NFNYKAG 1328
Ci-DuoxC            VRAELFPS------------------------------DVTHLEFKRPK----NFDYKAG 1193
Ci-DuoxD            IKAEILPS------------------------------DVVYLEFVRPS----DFYYKAG 1271
Sp-Duox             TRAELLPS------------------------------DVTMLEFKRPQ----GFEYKSG 1424
Dm-Duox             IDTDLLPS------------------------------DVIKIKFYRPP----NLKYLSG 1224
Ag-Duox             IETDLLPS------------------------------DVIKIKFYRPP----NLKYLSG 1225
Am-Duox             IETELLPS------------------------------DVIKIKFYRPP----NLKYLSG 1230
Ci-DuoxB            IKAEALPS------------------------------GVLNLVFKRPV----AFDYQSG 1247
Ce-Duox1            VNAEILPS------------------------------DIIYIEYRRPR----EFKYKSG 1244
Ce-Duox2            AHAEILPS------------------------------DIIYIEYRRPR----EFEYKSG 1250
                                                              * * *            *
```

	FAD1 FAD2	
Hs-Nox1	QYIFVNCPSISL--LEWHPFTLTSAP---EEDF-FSIHIRAA-GDWTE------------	368
Cf-Nox1	QYIFVNCPSVSY--LEWHPFTLTSAP---EEDF-FSVHIRAV-GDWTE------------	363
Mm-Nox1	QYIFVNCPSISF--LEWHPFTLTSAP---EEEF-FSVHIRAA-GDWTR------------	362
Rn-Nox1	QYIFVNCPSISF--LEWHPFTLTSAP---EEEF-FSIHIRAA-GDWTE------------	362
Gg-Nox1	QYIFVNCPAVSL--LEWHPFTLTSAP---EEDF-FSIHIRAA-GDWTE------------	364
Xt-Nox1	QYIFINCPSVSA--LEWHPFTLTSAP---EEDC-FSVHIRSA-GDWTD------------	364
Tn-Nox1	QYVFLNCPAISQ--LEWHPFTMTSAP---EEDF-FSVHIRSA-GDWTD------------	359
Tr-Nox1	QYVFLNCPAISQ--LEWHPFTMTSAP---EEDF-FSVHIRSA-GDWTD------------	357
Ol-Nox1	QYVFLNCPSISQ--LEWHPFTMTSAP---EEDF-FSVHIRSA-GDWTD------------	362
Dr-Nox1	QYVFLNCPAISQ--LEWHPFTLTSAP---EEDF-FSVHIRSV-GDWTE------------	361
Mm-Nox2	QYIFVKCPKVSK--LEWHPFTLTSAP---EEDF-FSIHIRIV-GDWTE------------	363
Rn-Nox2	QYIFVKCPQVSK--LEWHPFTLTSAP---EEDF-FSIHIRIV-GDWTE------------	363
Hs-Nox2	QYIFVKCPKVSK--LEWHPFTLTSAP---EEDF-FSIHIRIV-GDWTE------------	363
Cf-Nox2	QYIFVKCPKVSS--LEWHPFTLTSAP---EEDF-FSIHIRIV-GDWTE------------	363
Gg-Nox2	QYIFVKCPAVSK--LEWHPFTLTSAP---EEDY-FSIHVRIV-GDWTE------------	363
Xt-Nox2	QYIFVQCPAVSK--LEWHPFTLTSAP---EEDF-FSIHIRIV-GDWTE------------	363
Tn-Nox2	QYVFIQCPSVSR--LEWHPFTLTSAP---EEDY-FSAHIRIV-GDWTQ------------	361
Tr-Nox2	QYVFIQCPSVSR--LEWHPFTLTSAP---EEDY-FSAHIRIV-GDWTQ------------	361
Ol-Nox2	QYVFIQCPSISR--LEWHPFTLTSAP---EEDY-FSVHVRIV-GDWTQ------------	362
Dr-Nox2	QYIFMMCPSISQ--LEWHPFTLTSAP---EEDH-FSVHIRIV-GDWTQ------------	361
Hs-Nox3	QYILVQCPAISS--LEWHPFTLTSAP---QEDF-FSVHIRAA-GDWTA------------	361
Cf-Nox3	QYILVQCPSISW--LEWHPFTLTSAP---QEDF-FSLHIRVA-GDWTE------------	361
Mm-Nox3	QYIFIQCPSVSP--LEWHPFTLTSAP---QEDF-FSVHIRAS-GDWTE------------	361
Rn-Nox3	QYIFIQCPSISP--LEWHPFTLTSAP---QEDF-FSVHIRAS-GDWTE------------	361
Gg-Nox3	QYIFLQCTSISP--LEWHPFTLTSAP---EEDF-FSVHIRVA-GDWTA------------	360
Ci-Nox2	QYVFIMCPQLSQ--LEWHPFTLTSAP---EEDY-FSIHVRIV-GDWTT------------	364
Sp-Nox2A	QYIFLKCPSISH--VQWHPFTLTSAP---EEDH-FSVHIRVV-GDWTR------------	364
Sp-Nox2B	QYIFLKCSPISK--VQWHPFTLTSAP---EEDY-FSLHIRRV-GDWTD------------	348
Mm-Nox4	QYIILHCPSVSA--LENHPFTLTMCP-TETKAT-FGVHFKVV-GDWTE------------	379
Rn-Nox4	QYIILHCPSVSA--LENHPFTLTMCP-TETKAT-FGVHFKVV-GDWTE------------	379
Hs-Nox4	QYITLHCPSVSA--LENHPFTLTMCP-TETKAT-FGVHLKIV-GDWTE------------	379
Cf-Nox4	QYIILHCPSVSA--LENHPFTLTMCP-TETKAT-FGVHLKIV-GDWTE------------	385
Gg-Nox4	QYVILHCPRVSG--LESHPFTLTMVRNKNTKAT-FGVHLKVV-GDWTE------------	344
Xt-Nox4	QYITLLCPSVSA--LETHPFTLTMCP-TESKAT-FAIHIKVV-GDWTE------------	372
Ol-Nox4	QYILLNCPGVSP--FENHPFTLTACP-TENKQT-FGIHLRIV-GDWTE------------	357
Tr-Nox4	QYILLNCPAVSS--FENHPFTLTTCP-TENKKT-FSIHLRIV-GDWTE------------	358
Ci-Nox4	QCIWVRCPQLSK--VESHPFSLTSVP-SKDDPT-FGIHVKLR-GDWTE------------	443
Ag-Nox	QYVLLQCPAIST--LEWHPFTITELP-IEGRND-ITLTIKVR-GDWTE------------	356
Pa-NoxA	QWLFLQVPSVSK--YQWHPFTITSCP---YDPY-VSVHIRQV-GDFTR------------	314
Mg-NoxA	QWLFLQVPSVSK--YQWHPFTITSCP---YDPY-VSVHVRQV-GDFTK------------	340
Fg-NoxA	QWLFLQVPSLSK--YQWHPFTITSCP---FDPY-VSVHVRQV-GDFTR------------	317
An-NoxA	QWLFIQVPEVSN--TQWHPFTITSCP---FDDY-VSIHVRQV-GDFTR------------	312
Pa-NoxB	QYIFFCCPAVSL--WQYHPFTLTSAP---EEDY-ISIHMRVV-GDFTR------------	358
Fg-NoxB	QYIFLCCPAVSL--WQYHPFTLTSAP---EEDY-ISIHMRCV-GDFTK------------	347
Mg-NoxB	QYIFFCCPEVSV--WQYHPFTLTSAP---EEDY-ISIHMRVV-GDFTR------------	362
Dd-NoxA	QYLFLNCPTIAQ--NEWHPFTITSAP---EEDF-VSCHINVV-GNWTG------------	308
Dd-NoxB	QYLYLNCPSIAY--HEWHPFTITSAP---DDPF-ISVHINIV-GNWTR------------	485
At-rbohC	QYMFVNCAAVSP--FEWHPFSITSAP---QDDY-LSVHIRVL-GDWTR------------	662
At-rbohG	QYMFVNCPAVSP--FEWHPFSITSTP---QDDY-LSVHIKAL-GDWTE------------	615
At-rbohA	QYMYLNCSAVST--LEWHPFSITSAP---GDDY-LSVHIRVL-GDWTK------------	654
At-rbohB	QYIYINCSDVSP--LQWHPFSITSAS---GDDY-LSVHIRTL-GDWTS------------	608
At-rbohD	QFMLVNCRAVSP--FEWHPFSITSAP---GDDY-LSVHIRTL-GDWTR------------	685
At-rbohF	QYMFVQCPAVSP--FEWHPFSITSAP---EDDY-ISIHIRQL-GDWTQ------------	696
At-rbohI	QYVFVQCPSVSK--FEWHPFSITSSP---GDDY-LSIHIRQR-GDWTE------------	684

```
At-rbohE            QYIFLQCPTISR--FEWHPFSITSAP---GDDQ-LSVHIRTL-GDWTE------------ 674
At-rbohH            MYMFVKCPDLSK--FEWHPFSITSAP---GDDY-LSVHIRAL-GDWTT------------ 626
At-rbohJ            MYMFVKCPDISK--FEWHPFSITSAP---GDEY-LSVHIRAL-GDWTS------------ 636
Hs-Nox5             DYLYLNIPTIAR--YEWHPFTISSAPE--QKDT-IWLHIRSQ-GQWTN------------ 488
Cf-Nox5             DYLYLNIPTIAR--YEWHPFTISSAP---EQKDTIWLHIRSE-GQWTN------------ 515
Bt-Nox5             DYLYLNIPSIAR--YEWHPFTISSAP---EQKDTIWLHIRSQ-GQWTN------------ 523
Md-Nox5             DYIYLNIPAIAK--YEWHPFTISSAP---EQEDTIWLHIRSQ-GQWTN------------ 495
Xt-Nox5             DYIYLNIPVIAK--YEWHPFTISSAP---EQADTIWLHIRSL-GQWTN------------ 505
Tr-Nox5             DYVYINIPEIAK--YEWHPFTISSAP---EQSDCLWLHIRSM-GQWTNRLYEYFRQLDRQ 441
Tn-Nox5             RYVYINIPEIAK--YEWHPFTISSAP---EQSGTCRRRARKPPGLTGTNAPVDPLRFLPR 348
Gg-Nox5             DYIYLNIPAIAA--YEWHPFSISSAP---EQQDTIWLHIRSL-GQWTT------------ 478
Ol-Nox5             DYIYINIPVIAK--YEWHPFTISSAPE--QSDT-LWLHVRSM-GQWTN------------ 502
Dr-Nox5             DYVYINIPTIAK--YEWHPFTISSAPE--QQET-LWLHIRSM-GQWTN------------ 385
Sp-Nox5A            DYIFINIPQIAQ--YEWHPFTISSAPE--QQGT-ISMHIRSA-GNWTN------------ 499
Sp-Nox5B            EYIHVNIPSIAS--HEWHPFTISSAPE--QQEY-LTLHIRCV-GHWTK------------ 464
Ag-Nox5             DYVFVNIPAIAQ--YEWHPFTLSSAPE--QEDY-IWLHIRGV-GEWTN------------ 549
Am-Nox5             DYVFVNIPVIAR--YEWHPFTISSAPE--QEDY-IWLHIRAV-GEWTN------------ 551
Dm-Nox5             DYVFVNIPAIAN--YEWHPFTISSAPE--QEDY-MWLHIRTV-GEWTN------------ 554
Dd-NoxC             QYLLINVPHISK--LQWHPFTMTSSP---LEDK-IYVHIRVT-GNWTK------------ 874
Mg-NoxC             QWAYLQVPTISM--WQWHPFTISVCV----GKE-MRMHIKTD-GNWTG------------ 517
Fg-NoxC             QYIFLQVPKISF--FQWHPFTVSFCR----GNK-MMLHIKTD-GNWTA------------ 442
Cc-NoxD             QYAEVQVPFIN---REWHPFTIASAPQ---DKT-MCFYIKAL-GDWTK------------ 305
Py-NoxD             QYAEVKVPAISS--VQWHPFTIASAPH---EPE-LVFYIKKS-GDWTT------------ 301
Xt-Duox1            QWVRIACLDLGT--DEYHPFTLTSAP---HEDT-LSLHIRAV-GPWTT------------ 1298
Xt-Duox2            QWVRIACLALGT--NEYHPFTLTTAP---HEDI-LSLHIRAA-GPWTT------------ 1350
Gg-Duox             QWVRIACMALGT--TEYHPFTLTSAP---HEDT-LSLHIRAV-GPWTT------------ 1236
Tr-Duox             QWVRIACLTLGA--DEYHPFTLTSAP---HEDT-LSLHIRAV-GPWTS------------ 1268
Tn-Duox             QWVRIACLALGA--DEYHPFTLTSAP---HEGT-LSLHIRAV-GPWTS------------ 1419
Ol-Duox             QWVRIACLMLGA--DEYHPFTLTSAP---HEET-LSLHIRAV-GPWTS------------ 1345
Dr-Duox             QWVRIACLTLGT--DEYHPFTLTSAP---HEET-LSLHIRAA-GPWTS------------ 1296
Mm-Duox2            QWVRIACLDLGT--NEYHPFTLTSAP---HEDT-LSLHIRAV-GPWTT------------ 1310
Rn-Duox2            QWVRIACLSLGT--NEYHPFTLTSAP---HEDT-LSLHIRAV-GPWTT------------ 1310
Hs-Duox2            QWVRIACLALGT--TEYHPFTLTSAP---HEDT-LSLHIRAV-GPWTT------------ 1341
Cf-Duox2            QWVRIACLALGT--NEYHPFTLTSAP---HEDT-LSLHIRAV-GPWTT------------ 1093
Hs-Duox1            QWVRIACLALGT--TEYHPFTLTSAP---HEDT-LSLHIRAA-GPWTT------------ 1344
Cf-Duox1            QWVQIACLALGT--TEYHPFTLTSAP---HEDT-LSLHIRAA-GPWTT------------ 1345
Rn-Duox1            QWVRIACLALGT--TEYHPFTLTSAP---HEDT-LSLHIRAA-GPWTT------------ 1340
Mm-Duox1            QWVRIACLALGT--TEYHPFTLTSAP---HEDT-LSLHIRAA-GPWTT------------ 1344
Ci-DuoxA            QWVRIACLAQSQ--NEYHPFTLTSAP---HEDT-LKLHIRAV-GPWTI------------ 1369
Ci-DuoxC            QWVRIACLAQSS--SEYHPFTLSSAP---HEDT-LKLHIRAV-GPWTR------------ 1234
Ci-DuoxD            QWVRIACVGLSK--WEYHPFTLSSSP---DEET-LQLHIRAV-GPWTR------------ 1312
Sp-Duox             QWVRIACKTLSS--SEYHPFTLTSAP---HEEN-LSLHIRAI-GPWTM------------ 1465
Dm-Duox             QWVRLSCTAFRP--HEMHSFTLTSAP---HENF-LSCHIKAQ-GPWTW------------ 1265
Ag-Duox             QWVRLSCTEIKP--EEMHSFTLTSAP---HENF-LSCHIKAQ-GPWTW------------ 1266
Am-Duox             QWVRLSCTAFRS--NEFHSFTLTSAP---HENF-LSCHIKAQ-GPWTW------------ 1271
Ci-DuoxB            QWVRIASLSLGT--NEYHPFTLTSAP---HERY-LSLHIRSV-GPWTW------------ 1288
Ce-Duox1            QWVTVSSPSISCTFNESHAFSIASSP---QDEN-MKLYIKAV-GPWTW------------ 1287
Ce-Duox2            QWITVSSPSISCTFNESHAFSIASSP---QDEN-MKLYIKAV-GPWTS------------ 1293
                        *            * ****  *        *  ***   * **

Hs-Nox1             ------------------------------------------------------------
Cf-Nox1             ------------------------------------------------------------
Mm-Nox1             ------------------------------------------------------------
```

```
Rn-Nox1           ------------------------------------------------------------
Gg-Nox1           ------------------------------------------------------------
Xt-Nox1           ------------------------------------------------------------
Tn-Nox1           ------------------------------------------------------------
Tr-Nox1           ------------------------------------------------------------
Ol-Nox1           ------------------------------------------------------------
Dr-Nox1           ------------------------------------------------------------
Mm-Nox2           ------------------------------------------------------------
Rn-Nox2           ------------------------------------------------------------
Hs-Nox2           ------------------------------------------------------------
Cf-Nox2           ------------------------------------------------------------
Gg-Nox2           ------------------------------------------------------------
Xt-Nox2           ------------------------------------------------------------
Tn-Nox2           ------------------------------------------------------------
Tr-Nox2           ------------------------------------------------------------
Ol-Nox2           ------------------------------------------------------------
Dr-Nox2           ------------------------------------------------------------
Hs-Nox3           ------------------------------------------------------------
Cf-Nox3           ------------------------------------------------------------
Mm-Nox3           ------------------------------------------------------------
Rn-Nox3           ------------------------------------------------------------
Gg-Nox3           ------------------------------------------------------------
Ci-Nox2           ------------------------------------------------------------
Sp-Nox2A          ------------------------------------------------------------
Sp-Nox2B          ------------------------------------------------------------
Mm-Nox4           ------------------------------------------------------------
Rn-Nox4           ------------------------------------------------------------
Hs-Nox4           ------------------------------------------------------------
Cf-Nox4           ------------------------------------------------------------
Gg-Nox4           ------------------------------------------------------------
Xt-Nox4           ------------------------------------------------------------
Ol-Nox4           ------------------------------------------------------------
Tr-Nox4           ------------------------------------------------------------
Ci-Nox4           ------------------------------------------------------------
Ag-Nox            ------------------------------------------------------------
Pa-NoxA           ------------------------------------------------------------
Mg-NoxA           ------------------------------------------------------------
Fg-NoxA           ------------------------------------------------------------
An-NoxA           ------------------------------------------------------------
Pa-NoxB           ------------------------------------------------------------
Fg-NoxB           ------------------------------------------------------------
Mg-NoxB           ------------------------------------------------------------
Dd-NoxA           ------------------------------------------------------------
Dd-NoxB           ------------------------------------------------------------
At-rbohC          ------------------------------------------------------------
At-rbohG          ------------------------------------------------------------
At-rbohA          ------------------------------------------------------------
At-rbohB          ------------------------------------------------------------
At-rbohD          ------------------------------------------------------------
At-rbohF          ------------------------------------------------------------
At-rbohI          ------------------------------------------------------------
At-rbohE          ------------------------------------------------------------
At-rbohH          ------------------------------------------------------------
At-rbohJ          ------------------------------------------------------------
Hs-Nox5           ------------------------------------------------------------
```

```
Cf-Nox5         ------------------------------------------------------------
Bt-Nox5         ------------------------------------------------------------
Md-Nox5         ------------------------------------------------------------
Xt-Nox5         ------------------------------------------------------------
Tr-Nox5         VSTKRLSVTLRKHRQLPKAKVHTDTQRHLRPVAQLFTLTCVSSPQDEVFSSAKSNKAVAN 503
Tn-Nox5         ------------------------------------TPQLGGVLGGE------------- 359
Gg-Nox5         -----------------RLYEFFRQPEPLQPHGNLERKGGGSGCRWVSAGLCFLQ----- 516
Ol-Nox5         ------------------------------------------------------------
Dr-Nox5         ------------------------------------------------------------
Sp-Nox5A        ------------------------------------------------------------
Sp-Nox5B        ------------------------------------------------------------
Ag-Nox5         ------------------------------------------------------------
Am-Nox5         ------------------------------------------------------------
Dm-Nox5         ------------------------------------------------------------
Dd-NoxC         ------------------------------------------------------------
Mg-NoxC         ------------------------------------------------------------
Fg-NoxC         ------------------------------------------------------------
Cc-NoxD         ------------------------------------------------------------
Py-NoxD         ------------------------------------------------------------
Xt-Duox1        ------------------------------------------------------------
Xt-Duox2        ------------------------------------------------------------
Gg-Duox         ------------------------------------------------------------
Tr-Duox         ------------------------------------------------------------
Tn-Duox         ------------------------------------------------------------
Ol-Duox         ------------------------------------------------------------
Dr-Duox         ------------------------------------------------------------
Mm-Duox2        ------------------------------------------------------------
Rn-Duox2        ------------------------------------------------------------
Hs-Duox2        ------------------------------------------------------------
Cf-Duox2        ------------------------------------------------------------
Hs-Duox1        ------------------------------------------------------------
Cf-Duox1        ------------------------------------------------------------
Rn-Duox1        ------------------------------------------------------------
Mm-Duox1        ------------------------------------------------------------
Ci-DuoxA        ------------------------------------------------------------
Ci-DuoxC        ------------------------------------------------------------
Ci-DuoxD        ------------------------------------------------------------
Sp-Duox         ------------------------------------------------------------
Dm-Duox         ------------------------------------------------------------
Ag-Duox         ------------------------------------------------------------
Am-Duox         ------------------------------------------------------------
Ci-DuoxB        ------------------------------------------------------------
Ce-Duox1        ------------------------------------------------------------
Ce-Duox2        ------------------------------------------------------------


Hs-Nox1         ------------------------------------------------------------
Cf-Nox1         ------------------------------------------------------------
Mm-Nox1         ------------------------------------------------------------
Rn-Nox1         ------------------------------------------------------------
Gg-Nox1         ------------------------------------------------------------
Xt-Nox1         ------------------------------------------------------------
Tn-Nox1         ------------------------------------------------------------
```

```
Tr-Nox1             ------------------------------------------------------------
Ol-Nox1             ------------------------------------------------------------
Dr-Nox1             ------------------------------------------------------------
Mm-Nox2             ------------------------------------------------------------
Rn-Nox2             ------------------------------------------------------------
Hs-Nox2             ------------------------------------------------------------
Cf-Nox2             ------------------------------------------------------------
Gg-Nox2             ------------------------------------------------------------
Xt-Nox2             ------------------------------------------------------------
Tn-Nox2             ------------------------------------------------------------
Tr-Nox2             ------------------------------------------------------------
Ol-Nox2             ------------------------------------------------------------
Dr-Nox2             ------------------------------------------------------------
Hs-Nox3             ------------------------------------------------------------
Cf-Nox3             ------------------------------------------------------------
Mm-Nox3             ------------------------------------------------------------
Rn-Nox3             ------------------------------------------------------------
Gg-Nox3             ------------------------------------------------------------
Ci-Nox2             ------------------------------------------------------------
Sp-Nox2A            ------------------------------------------------------------
Sp-Nox2B            ------------------------------------------------------------
Mm-Nox4             ------------------------------------------------------------
Rn-Nox4             ------------------------------------------------------------
Hs-Nox4             ------------------------------------------------------------
Cf-Nox4             ------------------------------------------------------------
Gg-Nox4             ------------------------------------------------------------
Xt-Nox4             ------------------------------------------------------------
Ol-Nox4             ------------------------------------------------------------
Tr-Nox4             ------------------------------------------------------------
Ci-Nox4             ------------------------------------------------------------
Ag-Nox              ------------------------------------------------------------
Pa-NoxA             ------------------------------------------------------------
Mg-NoxA             ------------------------------------------------------------
Fg-NoxA             ------------------------------------------------------------
An-NoxA             ------------------------------------------------------------
Pa-NoxB             ------------------------------------------------------------
Fg-NoxB             ------------------------------------------------------------
Mg-NoxB             ------------------------------------------------------------
Dd-NoxA             ------------------------------------------------------------
Dd-NoxB             ------------------------------------------------------------
At-rbohC            ------------------------------------------------------------
At-rbohG            ------------------------------------------------------------
At-rbohA            ------------------------------------------------------------
At-rbohB            ------------------------------------------------------------
At-rbohD            ------------------------------------------------------------
At-rbohF            ------------------------------------------------------------
At-rbohI            ------------------------------------------------------------
At-rbohE            ------------------------------------------------------------
At-rbohH            ------------------------------------------------------------
At-rbohJ            ------------------------------------------------------------
Hs-Nox5             ------------------------------------------------------------
Cf-Nox5             ------------------------------------------------------------
Bt-Nox5             ------------------------------------------------------------
Md-Nox5             ------------------------------------------------------------
Xt-Nox5             ------------------------------------------------------------
```

```
Tr-Nox5       EDDAVTLMMYQQRSSGADVVSPAAPESQVPPEPLLDELSPAERG---------------- 547
Tn-Nox5       ------------------------------------------------------------
Gg-Nox5       AQRLNRAVCRLQGGLRAVPVGRSAPAARSTQHRLGLSKKGNKNHTGDAAIELTSYRRSGA 576
Ol-Nox5       ------------------------------------------------------------
Dr-Nox5       ------------------------------------------------------------
Sp-Nox5A      ------------------------------------------------------------
Sp-Nox5B      ------------------------------------------------------------
Ag-Nox5       ------------------------------------------------------------
Am-Nox5       ------------------------------------------------------------
Dm-Nox5       ------------------------------------------------------------
Dd-NoxC       ------------------------------------------------------------
Mg-NoxC       ------------------------------------------------------------
Fg-NoxC       ------------------------------------------------------------
Cc-NoxD       ------------------------------------------------------------
Py-NoxD       ------------------------------------------------------------
Xt-Duox1      ------------------------------------------------------------
Xt-Duox2      ------------------------------------------------------------
Gg-Duox       ------------------------------------------------------------
Tr-Duox       ------------------------------------------------------------
Tn-Duox       ------------------------------------------------------------
Ol-Duox       ------------------------------------------------------------
Dr-Duox       ------------------------------------------------------------
Mm-Duox2      ------------------------------------------------------------
Rn-Duox2      ------------------------------------------------------------
Hs-Duox2      ------------------------------------------------------------
Cf-Duox2      ------------------------------------------------------------
Hs-Duox1      ------------------------------------------------------------
Cf-Duox1      ------------------------------------------------------------
Rn-Duox1      ------------------------------------------------------------
Mm-Duox1      ------------------------------------------------------------
Ci-DuoxA      ------------------------------------------------------------
Ci-DuoxC      ------------------------------------------------------------
Ci-DuoxD      ------------------------------------------------------------
Sp-Duox       ------------------------------------------------------------
Dm-Duox       ------------------------------------------------------------
Ag-Duox       ------------------------------------------------------------
Am-Duox       ------------------------------------------------------------
Ci-DuoxB      ------------------------------------------------------------
Ce-Duox1      ------------------------------------------------------------
Ce-Duox2      ------------------------------------------------------------


Hs-Nox1       ------------------------------------------------------------
Cf-Nox1       ------------------------------------------------------------
Mm-Nox1       ------------------------------------------------------------
Rn-Nox1       ------------------------------------------------------------
Gg-Nox1       ------------------------------------------------------------
Xt-Nox1       ------------------------------------------------------------
Tn-Nox1       ------------------------------------------------------------
Tr-Nox1       ------------------------------------------------------------
Ol-Nox1       ------------------------------------------------------------
Dr-Nox1       ------------------------------------------------------------
Mm-Nox2       ------------------------------------------------------------
```

```
Rn-Nox2         ------------------------------------------------------------
Hs-Nox2         ------------------------------------------------------------
Cf-Nox2         ------------------------------------------------------------
Gg-Nox2         ------------------------------------------------------------
Xt-Nox2         ------------------------------------------------------------
Tn-Nox2         ------------------------------------------------------------
Tr-Nox2         ------------------------------------------------------------
Ol-Nox2         ------------------------------------------------------------
Dr-Nox2         ------------------------------------------------------------
Hs-Nox3         ------------------------------------------------------------
Cf-Nox3         ------------------------------------------------------------
Mm-Nox3         ------------------------------------------------------------
Rn-Nox3         ------------------------------------------------------------
Gg-Nox3         ------------------------------------------------------------
Ci-Nox2         ------------------------------------------------------------
Sp-Nox2A        ------------------------------------------------------------
Sp-Nox2B        ------------------------------------------------------------
Mm-Nox4         ------------------------------------------------------------
Rn-Nox4         ------------------------------------------------------------
Hs-Nox4         ------------------------------------------------------------
Cf-Nox4         ------------------------------------------------------------
Gg-Nox4         ------------------------------------------------------------
Xt-Nox4         ------------------------------------------------------------
Ol-Nox4         ------------------------------------------------------------
Tr-Nox4         ------------------------------------------------------------
Ci-Nox4         ------------------------------------------------------------
Ag-Nox          ------------------------------------------------------------
Pa-NoxA         ------------------------------------------------------------
Mg-NoxA         ------------------------------------------------------------
Fg-NoxA         ------------------------------------------------------------
An-NoxA         ------------------------------------------------------------
Pa-NoxB         ------------------------------------------------------------
Fg-NoxB         ------------------------------------------------------------
Mg-NoxB         ------------------------------------------------------------
Dd-NoxA         ------------------------------------------------------------
Dd-NoxB         ------------------------------------------------------------
At-rbohC        ------------------------------------------------------------
At-rbohG        ------------------------------------------------------------
At-rbohA        ------------------------------------------------------------
At-rbohB        ------------------------------------------------------------
At-rbohD        ------------------------------------------------------------
At-rbohF        ------------------------------------------------------------
At-rbohI        ------------------------------------------------------------
At-rbohE        ------------------------------------------------------------
At-rbohH        ------------------------------------------------------------
At-rbohJ        ------------------------------------------------------------
Hs-Nox5         ------------------------------------------------------------
Cf-Nox5         ------------------------------------------------------------
Bt-Nox5         ------------------------------------------------------------
Md-Nox5         ------------------------------------------------------------
Xt-Nox5         ------------------------------------------------------------
Tr-Nox5         --------------------------EAPPLREVRDHIKGLLGLLFYSNKGFNLSSLYCR 581
Tn-Nox5         ------------------------------------------------------------
Gg-Nox5         PTTTRGTEDGDRDWDTSLQAVGASTAPKPPRRPQGRGGPSLSCDARCPNDFGIEVGVHRS 636
Ol-Nox5         ------------------------------------------------------------
```

```
Dr-Nox5         ------------------------------------------------------------
Sp-Nox5A        ------------------------------------------------------------
Sp-Nox5B        ------------------------------------------------------------
Ag-Nox5         ------------------------------------------------------------
Am-Nox5         ------------------------------------------------------------
Dm-Nox5         ------------------------------------------------------------
Dd-NoxC         ------------------------------------------------------------
Mg-NoxC         ------------------------------------------------------------
Fg-NoxC         ------------------------------------------------------------
Cc-NoxD         ------------------------------------------------------------
Py-NoxD         ------------------------------------------------------------
Xt-Duox1        ------------------------------------------------------------
Xt-Duox2        ------------------------------------------------------------
Gg-Duox         ------------------------------------------------------------
Tr-Duox         ------------------------------------------------------------
Tn-Duox         ------------------------------------------------------------
Ol-Duox         ------------------------------------------------------------
Dr-Duox         ------------------------------------------------------------
Mm-Duox2        ------------------------------------------------------------
Rn-Duox2        ------------------------------------------------------------
Hs-Duox2        ------------------------------------------------------------
Cf-Duox2        ------------------------------------------------------------
Hs-Duox1        ------------------------------------------------------------
Cf-Duox1        ------------------------------------------------------------
Rn-Duox1        ------------------------------------------------------------
Mm-Duox1        ------------------------------------------------------------
Ci-DuoxA        ------------------------------------------------------------
Ci-DuoxC        ------------------------------------------------------------
Ci-DuoxD        ------------------------------------------------------------
Sp-Duox         ------------------------------------------------------------
Ce-Duox1        ------------------------------------------------------------
Ce-Duox2        ------------------------------------------------------------
Dm-Duox         ------------------------------------------------------------
Ag-Duox         ------------------------------------------------------------
Am-Duox         ------------------------------------------------------------
Ci-DuoxB        ------------------------------------------------------------


Hs-Nox1         --------------------NLIRAFEQQYS----------------------------- 374
Cf-Nox1         --------------------NLIRAFEQQCS----------------------------- 374
Mm-Nox1         --------------------NLIRTFEQQHS----------------------------- 373
Rn-Nox1         --------------------NLIRTFEQQHS----------------------------- 373
Gg-Nox1         --------------------HIIDTFQQQKL----------------------------- 375
Xt-Nox1         --------------------NLIKVFQEQAE----------------------------- 375
Tn-Nox1         --------------------KLIDIMQKLPEG---------------------------- 371
Tr-Nox1         --------------------KLIEIMQQLPEG---------------------------- 369
Ol-Nox1         --------------------QLIDTMQKLPEG---------------------------- 374
Dr-Nox1         --------------------KLLKMVENLPEG---------------------------- 373
Mm-Nox2         --------------------GLFNACGCDKQE---------------------------- 375
Rn-Nox2         --------------------GLFNACGCDKQE---------------------------- 375
Hs-Nox2         --------------------GLFNACGCDKQE---------------------------- 375
Cf-Nox2         --------------------GLFNACGCDKQE---------------------------- 375
Gg-Nox2         --------------------GLFNACGCDKQE---------------------------- 375
```

```
Xt-Nox2                 --------------------GLFKACGCDKTE---------------------------- 375
Tn-Nox2                 --------------------ALYEACGGDRSE---------------------------- 373
Tr-Nox2                 --------------------ALYEACGGDKSE---------------------------- 373
Ol-Nox2                 --------------------ALYEACGGNKSE---------------------------- 374
Dr-Nox2                 --------------------ALYSACGGDKTA---------------------------- 373
Hs-Nox3                 --------------------ALLEAFGAEGQA---------------------------- 373
Cf-Nox3                 --------------------ALWKAFGAEGQA---------------------------- 373
Mm-Nox3                 --------------------ALLKAFRVEGQA---------------------------- 373
Rn-Nox3                 --------------------ALLKAFGAEGQA---------------------------- 373
Gg-Nox3                 --------------------ALFKAFGAEEKT---------------------------- 372
Ci-Nox2                 --------------------GLSKVLGADEAGN--------------------------- 387
Sp-Nox2A                --------------------DLFKAMGADKPE---------------------------- 386
Sp-Nox2B                --------------------ELAVKMGADQAE---------------------------- 360
Mm-Nox4                 --------------------RFRDLLLPPSSQD--------------------------- 392
Rn-Nox4                 --------------------RFRDLLLPPSSQD--------------------------- 392
Hs-Nox4                 --------------------RFRDLLLPPSSQD--------------------------- 392
Cf-Nox4                 --------------------RFRDLLLPPSNQD--------------------------- 398
Gg-Nox4                 --------------------RFRDLLLLHSNQD--------------------------- 357
Xt-Nox4                 --------------------RFYELLESHLTAG--------------------------- 385
Ol-Nox4                 --------------------HFAHLLLPQPRAA--------------------------- 370
Tr-Nox4                 --------------------RFTQLLLTGSRTD--------------------------- 371
Ci-Nox4                 --------------------ELRDLMVRELNPV--------------------------- 456
Ag-Nox                  --------------------ELYDRIVQREQCK--------------------------- 369
Pa-NoxA                 --------------------ELGNAVGAGGIHA--------------------------- 327
Mg-NoxA                 --------------------ALGDATGAGAAQA--------------------------- 353
Fg-NoxA                 --------------------ELGDALGAGAAQA--------------------------- 330
An-NoxA                 --------------------ALGDALGCGPAQA--------------------------- 325
Pa-NoxB                 --------------------AVAETLGCEFDKK--------------------------- 371
Fg-NoxB                 --------------------ELAKSLGCDWSKK--------------------------- 360
Mg-NoxB                 --------------------GVSKALGCDWDRK--------------------------- 375
Dd-NoxA                 --------------------KLSTLLNPDKKMG--------------------------- 321
Dd-NoxB                 --------------------KLFKLLNPDNKLG--------------------------- 498
At-rbohC                --------------------ALKGVFSEVC------------------------------ 672
At-rbohG                --------------------AIQGVFSEVS------------------------------ 625
At-rbohA                --------------------QLRSLFSEVC------------------------------ 664
At-rbohB                --------------------QLKSLYSKVC------------------------------ 618
At-rbohD                --------------------KLRTVFSEVC------------------------------ 695
At-rbohF                --------------------ELKRVFSEVC------------------------------ 706
At-rbohI                --------------------GIKKAFSVVC------------------------------ 694
At-rbohE                --------------------ELRRVLTVG------------------------------- 683
At-rbohH                --------------------ELRSRFAKTC------------------------------ 636
At-rbohJ                --------------------ELRNRFAETC------------------------------ 646
Hs-Nox5                 --------------------RLYESFKAS------------------------------- 497
Cf-Nox5                 --------------------RLYESFKTSCPMD--------------------------- 528
Bt-Nox5                 --------------------RLFESFKKPEPVF--------------------------- 536
Md-Nox5                 --------------------RLYEYFKAADPIC--------------------------- 508
Xt-Nox5                 --------------------SLYEYFHYPQTVN--------------------------- 518
Tr-Nox5                 ------FLQSLEIIIGSATSRSKAQLRNAANKM--------------------------- 608
Tn-Nox5                 ---------------GDCTLSSPRRLPVAPHPL--------------------------- 377
Gg-Nox5                 ISHHSHWAQGSWESGCGECWKLWEQGGGGFPLL--------------------------- 669
Ol-Nox5                 --------------------RLYEYFRQT------------------------------- 511
Dr-Nox5                 --------------------RLYEYFRQP------------------------------- 394
Sp-Nox5A                --------------------RLYAFFEDR------------------------------- 508
Sp-Nox5B                --------------------RLYDVVRERELTLLEHENAGFGEIDKDHDEPLEVIVDVSS 504
Ag-Nox5                 --------------------RLHNFFEREQERLHN------GEIPALVAGRVGGAGGPVG 583
```

```
Am-Nox5      --------------------SLYSYFEKEQMKLQR------DNIFPIEN----------- 574
Dm-Nox5      --------------------RLYRYFEREQQKLQQ------SGSSQEIPQHMHAIPTPSF 588
Dd-NoxC      --------------------KLFRWLSIKKQLQ--------------------------- 887
Mg-NoxC      --------------------RLRDLAKDAPQ----------------------------- 528
Fg-NoxC      --------------------KLRELGGDS------------------------------- 451
Cc-NoxD      --------------------ELRGAFQARVDG---------------------------- 317
Py-NoxD      --------------------NLHAMFAS-------------------------------- 309
Xt-Duox1     --------------------KLRELYSSKKVENIP------------------------- 1313
Xt-Duox2     --------------------RLRELYSPQSVAELGG------------------------ 1366
Gg-Duox      --------------------RLRELYSPESLALIGK------------------------ 1252
Tr-Duox      --------------------QLRELYAEESVLRGLSE----------------------- 1285
Tn-Duox      --------------------RLRELYTQDSLQQ--------------------------- 1432
Ol-Duox      --------------------QLRELYTDESLLE--------------------------- 1358
Dr-Duox      --------------------KLREAYSPEKHQS--------------------------- 1309
Mm-Duox2     --------------------RLREIYSPPVGGT--------------------------- 1323
Rn-Duox2     --------------------RLREIYSPPVGGT--------------------------- 1323
Hs-Duox2     --------------------RLREIYSSPKGNG--------------------------- 1354
Cf-Duox2     --------------------RLRETYSLPKGDG--------------------------- 1106
Hs-Duox1     --------------------RLREIYSAPTGDR--------------------------- 1357
Cf-Duox1     --------------------RLREIYSPPTGDG--------------------------- 1358
Rn-Duox1     --------------------RLREIYSPPTGDT--------------------------- 1353
Mm-Duox1     --------------------RLREIYSPPTGDT--------------------------- 1357
Ci-DuoxA     --------------------NLRSIYNPD-VLRDSP------------------------ 1384
Ci-DuoxC     --------------------NLRNIYDPN-VLRDSP------------------------ 1249
Ci-DuoxD     --------------------NIRNIYK-----EGEP------------------------ 1323
Sp-Duox      --------------------NLRATYDPN-VVREHP------------------------ 1480
Ce-Duox1     --------------------KLRSELIRS-LNTGSP------------------------ 1302
Ce-Duox2     --------------------KLRSELIRS-LNTGSP------------------------ 1308
Dm-Duox      --------------------KLRNYFDPCNYNPED------------------------- 1280
Ag-Duox      --------------------KLRNYFDPCNYNPDD------------------------- 1281
Am-Duox      --------------------KLRNYFDPCNYNPEDE------------------------ 1287
Ci-DuoxB     --------------------NLRNLYQTA-VEHQGK------------------------ 1303


Hs-Nox1      ---------------------------PIP------------------------------ 377
Cf-Nox1      ---------------------------PIP------------------------------ 377
Mm-Nox1      ---------------------------PMP------------------------------ 376
Rn-Nox1      ---------------------------PMP------------------------------ 376
Gg-Nox1      ---------------------------EMP------------------------------ 378
Xt-Nox1      ---------------------------NPP------------------------------ 378
Tn-Nox1      --------------------------AQGP------------------------------ 375
Tr-Nox1      --------------------------AQGP------------------------------ 373
Ol-Nox1      --------------------------AQGP------------------------------ 378
Dr-Nox1      --------------------------GQGPKYVLL------------------------- 382
Mm-Nox2      --------------------------FQDAWKLP-------------------------- 383
Rn-Nox2      --------------------------FQDAWKLP-------------------------- 383
Hs-Nox2      --------------------------FQDAWKLP-------------------------- 383
Cf-Nox2      --------------------------FQDAWKLP-------------------------- 383
Gg-Nox2      --------------------------FQEAWKLP-------------------------- 383
Xt-Nox2      --------------------------FQDAWKMP-------------------------- 383
Tn-Nox2      --------------------------PQEAWKLP-------------------------- 381
Tr-Nox2      --------------------------PQEAWKLP-------------------------- 381
Ol-Nox2      --------------------------LQEAWKLP-------------------------- 382
```

```
Dr-Nox2     --------------------------VLDAWTLP-------------------------- 381
Hs-Nox3     --------------------------LQEPWSLP-------------------------- 381
Cf-Nox3     --------------------------LKEPWSLP-------------------------- 381
Mm-Nox3     --------------------------PSELCSMP-------------------------- 381
Rn-Nox3     --------------------------PSELCSMP-------------------------- 381
Gg-Nox3     --------------------------FKELWMLP-------------------------- 380
Ci-Nox2     -------------------------EVQPSWKMP-------------------------- 396
Sp-Nox2A    --------------------------QQSQDELA-------------------------- 394
Sp-Nox2B    --------------------------PLSITQLP-------------------------- 368
Mm-Nox4     -------------------------SEILP------------------------------ 397
Rn-Nox4     -------------------------SEILP------------------------------ 397
Hs-Nox4     -------------------------SEILP------------------------------ 397
Cf-Nox4     -------------------------SEILP------------------------------ 403
Gg-Nox4     -------------------------AEILP------------------------------ 362
Xt-Nox4     -------------------------TEILP------------------------------ 390
Ol-Nox4     -------------------------LEILP------------------------------ 375
Tr-Nox4     -------------------------MKTLP------------------------------ 376
Ci-Nox4     -------------------------VEILKDKIIAGGNFLEKDDELSNRGETEYKCNPDN 491
Ag-Nox      -------------------------RNLGG------------------------------ 374
Pa-NoxA     -------------------------KLY-------------------------------- 330
Mg-NoxA     -------------------------KLY-------------------------------- 356
Fg-NoxA     -------------------------KLY-------------------------------- 333
An-NoxA     -------------------------RDL-------------------------------- 328
Pa-NoxB     -------------------------K---------------------------------- 372
Fg-NoxB     -------------------------KDA-------------------------------- 363
Mg-NoxB     -------------------------G---------------------------------- 376
Dd-NoxA     -------------------------IVQ-------------------------------- 324
Dd-NoxB     -------------------------LIQ-------------------------------- 501
At-rbohC    -------------------------KPPPAGVSGLLR----------------------- 684
At-rbohG    -------------------------KPPPVG----------------------------- 631
At-rbohA    -------------------------KPRPPDEHRLNR----------------------- 676
At-rbohB    -------------------------QLPSTSQSGLFI----------------------- 630
At-rbohD    -------------------------KPPTAGKSGLLR----------------------- 707
At-rbohF    -------------------------EPPVGGKSGLLR----------------------- 718
At-rbohI    -------------------------HAPEAGKSGLLR----------------------- 706
At-rbohE    -------------------------KDLSTCVIGRSK----------------------- 695
At-rbohH    -------------------------EPTQAAAKPKPNSLMRM------------------ 653
At-rbohJ    -------------------------EPHQKS-KPSPNDLIRM------------------ 662
Hs-Nox5     ----------------DPLGR--GSKRLSRSVTMRKSQRS-------------------- 519
Cf-Nox5     -------------------------CDPKP------------------------------ 533
Bt-Nox5     -------------------------CGSKR------------------------------ 541
Md-Nox5     -------------------------LGAKR------------------------------ 513
Xt-Nox5     -------------------------RHETKR----------------------------- 524
Tr-Nox5     -------------------------HLNNFLFKMSFLADEGKQENHSLV----------- 632
Tn-Nox5     -------------------------HGPVDQPPVRVLPGTGE------------------ 394
Gg-Nox5     -------------------------LMDAPHLQVSVGPGESQQLCSIKVSSGERNGVG-- 702
Ol-Nox5     ----------------DSMEL--CSGRLATSLKKRRQQAK-------------------- 533
Dr-Nox5     --------------------DTQTNKRLTASLRSRRHQSR-------------------- 412
Sp-Nox5A    ----------------QKRNRDETELLLGSASDIRVAMET-------------------- 532
Sp-Nox5B    TKSTTAVEPNGQQSIISDTHSNRNGRRSSQTYSKRRRTASGAINSGFEPELPNGDKDGTK 564
Ag-Nox5     PRAGDATTPAGIMKQRHPPGTSKLAMEGYSAPSPVATSTAQP------AKFERQMSDNRA 637
Am-Nox5     ---------------RNNPNVSESVSMNGKFLSPIPRGNRRS------FDNSVFVSDD-- 611
Dm-Nox5     MLLNEARNPAIAGERSATPQTDFLAKNLGVQAVPPVRPPRQNRKPAPGAPIDPPATGVNR 648
Dd-NoxC     -------------------------QQQQLYNNIKQQNVLP------------------- 903
Mg-NoxC     ------------------------------------------------------------
```

```
Fg-NoxC                ------------------------------------------------------------
Cc-NoxD                ------------------------------------------------------------
Py-NoxD                ------------------------------------------------------------
Xt-Duox1               ------------------------------------------------------------
Xt-Duox2               ------------------------------------------------------------
Gg-Duox                ------------------------------------------------------------
Tr-Duox                ------------------------------------------------------------
Tn-Duox                ------------------------------------------------------------
Ol-Duox                ------------------------------------------------------------
Dr-Duox                ------------------------------------------------------------
Mm-Duox2               ------------------------------------------------------------
Rn-Duox2               ------------------------------------------------------------
Hs-Duox2               ------------------------------------------------------------
Cf-Duox2               ------------------------------------------------------------
Hs-Duox1               ------------------------------------------------------------
Cf-Duox1               ------------------------------------------------------------
Rn-Duox1               ------------------------------------------------------------
Mm-Duox1               ------------------------------------------------------------
Ci-DuoxA               ------------------------------------------------------------
Ci-DuoxC               ------------------------------------------------------------
Ci-DuoxD               ------------------------------------------------------------
Sp-Duox                ------------------------------------------------------------
Ce-Duox1               ------------------------------------------------------------
Ce-Duox2               ------------------------------------------------------------
Dm-Duox                ------------------------------------------------------------
Ag-Duox                ------------------------------------------------------------
Am-Duox                ------------------------------------------------------------
Ci-DuoxB               ------------------------------------------------------------


Hs-Nox1                ------------------------------------------------------------
Cf-Nox1                ------------------------------------------------------------
Mm-Nox1                ------------------------------------------------------------
Rn-Nox1                ------------------------------------------------------------
Gg-Nox1                ------------------------------------------------------------
Xt-Nox1                ------------------------------------------------------------
Tn-Nox1                ------------------------------------------------------------
Tr-Nox1                ------------------------------------------------------------
Ol-Nox1                ------------------------------------------------------------
Dr-Nox1                ------------------------------------------------------------
Mm-Nox2                ------------------------------------------------------------
Rn-Nox2                ------------------------------------------------------------
Hs-Nox2                ------------------------------------------------------------
Cf-Nox2                ------------------------------------------------------------
Gg-Nox2                ------------------------------------------------------------
Xt-Nox2                ------------------------------------------------------------
Tn-Nox2                ------------------------------------------------------------
Tr-Nox2                ------------------------------------------------------------
Ol-Nox2                ------------------------------------------------------------
Dr-Nox2                ------------------------------------------------------------
Hs-Nox3                ------------------------------------------------------------
Cf-Nox3                ------------------------------------------------------------
Mm-Nox3                ------------------------------------------------------------
```

```
Rn-Nox3         ------------------------------------------------------------
Gg-Nox3         ------------------------------------------------------------
Ci-Nox2         ------------------------------------------------------------
Sp-Nox2A        ------------------------------------------------------------
Sp-Nox2B        ------------------------------------------------------------
Mm-Nox4         ------------------------------------------------------------
Rn-Nox4         ------------------------------------------------------------
Hs-Nox4         ------------------------------------------------------------
Cf-Nox4         ------------------------------------------------------------
Gg-Nox4         ------------------------------------------------------------
Xt-Nox4         ------------------------------------------------------------
Ol-Nox4         ------------------------------------------------------------
Tr-Nox4         ------------------------------------------------------------
Ci-Nox4         SQYLATMASPNQSINKPCMVPNFRTQETDFQMQCTL------------------------ 527
Ag-Nox          ------------------------------------------------------------
Pa-NoxA         ------------------------------------------------------------
Mg-NoxA         ------------------------------------------------------------
Fg-NoxA         ------------------------------------------------------------
An-NoxA         ------------------------------------------------------------
Pa-NoxB         ------------------------------------------------------------
Fg-NoxB         ------------------------------------------------------------
Mg-NoxB         ------------------------------------------------------------
Dd-NoxA         ------------------------------------------------------------
Dd-NoxB         ------------------------------------------------------------
At-rbohC        ------------------------------------------------------------
At-rbohG        ------------------------------------------------------------
At-rbohA        ------------------------------------------------------------
At-rbohB        ------------------------------------------------------------
At-rbohD        ------------------------------------------------------------
At-rbohF        ------------------------------------------------------------
At-rbohI        ------------------------------------------------------------
At-rbohE        ------------------------------------------------------------
At-rbohH        ------------------------------------------------------------
At-rbohJ        ------------------------------------------------------------
Hs-Nox5         ------------------------------------------------SKGSE------- 524
Cf-Nox5         ------------------------------------------------------------
Bt-Nox5         ------------------------------------------------------------
Md-Nox5         ------------------------------------------------------------
Xt-Nox5         ------------------------------------------------------------
Tr-Nox5         ------------------------------------------------------------
Tn-Nox5         ------------------------------------------------------------
Gg-Nox5         ------------------------------------------------------------
Ol-Nox5         ------------------------------------------------AEVSA------- 538
Dr-nox5         ------------------------------------------------AQLTAKLSEN-- 424
Sp-Nox5A        ------------------------------------------------EEVEETNHAGEF 544
Sp-Nox5B        TVTEGKPYNTGQNDNESVTQKVNAAQLSLPRKADHPSASQGDCQPMSYKELGEVSMTLEV 624
Ag-Nox5         FKKIQATLQRTFSRRDQLIPRSGGAGGVGGIANEG--FSGDGQKVPLEKSLSMPDMQNKF 695
Am-Nox5         -EKITQSPQSVIGNLFKFIQNYLSITDVKKMSNNRKLHSLLVSKMPLEKSVSVPDMLPGK 670
Dm-Nox5         IRSIKKTLQRTFSRKEAVDPKKGIPNGAFIADGER--EDSNLKQRPLEKSISLPDISVKS 706
Dd-NoxC         ------------------------------------------------------------
Mg-NoxC         ------------------------------------------------------------
Fg-NoxC         ------------------------------------------------------------
Cc-NoxD         ------------------------------------------------------------
Py-NoxD         ------------------------------------------------------------
Xt-Duox1        ------------------------------------------------------------
```

```
Xt-Duox2             ------------------------------------------------------------
Gg-Duox              ------------------------------------------------------------
Tr-Duox              ------------------------------------------------------------
Tn-Duox              ------------------------------------------------------------
Ol-Duox              ------------------------------------------------------------
Dr-Duox              ------------------------------------------------------------
Mm-Duox2             ------------------------------------------------------------
Rn-Duox2             ------------------------------------------------------------
Hs-Duox2             ------------------------------------------------------------
Cf-Duox2             ------------------------------------------------------------
Hs-Duox1             ------------------------------------------------------------
Cf-Duox1             ------------------------------------------------------------
Rn-Duox1             ------------------------------------------------------------
Mm-Duox1             ------------------------------------------------------------
Ci-DuoxA             ------------------------------------------------------------
Ci-DuoxC             ------------------------------------------------------------
Ci-DuoxD             ------------------------------------------------------------
Sp-Duox              ------------------------------------------------------------
Dm-Duox              ------------------------------------------------------------
Ag-Duox              ------------------------------------------------------------
Am-Duox              ------------------------------------------------------------
Ci-DuoxB             ------------------------------------------------------------
Ce-Duox1             ------------------------------------------------------------
Ce-Duox2             ------------------------------------------------------------
```

```
Hs-Nox1              ------------------------------------------------------------
Cf-Nox1              ------------------------------------------------------------
Mm-Nox1              ------------------------------------------------------------
Rn-Nox1              ------------------------------------------------------------
Gg-Nox1              ------------------------------------------------------------
Xt-Nox1              ------------------------------------------------------------
Tn-Nox1              ------------------------------------------------------------
Tr-Nox1              ------------------------------------------------------------
Ol-Nox1              ------------------------------------------------------------
Dr-Nox1              ------------------------------------------------------------
Mm-Nox2              ------------------------------------------------------------
Rn-Nox2              ------------------------------------------------------------
Hs-Nox2              ------------------------------------------------------------
Cf-Nox2              ------------------------------------------------------------
Gg-Nox2              ------------------------------------------------------------
Xt-Nox2              ------------------------------------------------------------
Tn-Nox2              ------------------------------------------------------------
Tr-Nox2              ------------------------------------------------------------
Ol-Nox2              ------------------------------------------------------------
Dr-Nox2              ------------------------------------------------------------
Hs-Nox3              ------------------------------------------------------------
Cf-Nox3              ------------------------------------------------------------
Mm-Nox3              ------------------------------------------------------------
Rn-Nox3              ------------------------------------------------------------
Gg-Nox3              ------------------------------------------------------------
Ci-Nox2              ------------------------------------------------------------
Sp-Nox2A             ------------------------------------------------------------
```

```
Sp-Nox2B    ------------------------------------------------------------
Mm-Nox4     ------------------------------------------------------------
Rn-Nox4     ------------------------------------------------------------
Hs-Nox4     ------------------------------------------------------------
Cf-Nox4     ------------------------------------------------------------
Gg-Nox4     ------------------------------------------------------------
Xt-Nox4     ------------------------------------------------------------
Ol-Nox4     ------------------------------------------------------------
Tr-Nox4     ------------------------------------------------------------
Ci-Nox4     ------------------------------------------------------------
Ag-Nox      ------------------------------------------------------------
Pa-NoxA     ------------------------------------------------------------
Mg-NoxA     ------------------------------------------------------------
Fg-NoxA     ------------------------------------------------------------
An-NoxA     ------------------------------------------------------------
Pa-NoxB     ------------------------------------------------------------
Fg-NoxB     ------------------------------------------------------------
Mg-NoxB     ------------------------------------------------------------
Dd-NoxA     ------------------------------------------------------------
Dd-NoxB     ------------------------------------------------------------
At-rbohC    ------------------------------------------------------------
At-rbohG    ------------------------------------------------------------
At-rbohA    ------------------------------------------------------------
At-rbohB    ------------------------------------------------------------
At-rbohD    ------------------------------------------------------------
At-rbohF    ------------------------------------------------------------
At-rbohI    ------------------------------------------------------------
At-rbohE    ------------------------------------------------------------
At-rbohH    ------------------------------------------------------------
At-rbohJ    ------------------------------------------------------------
Hs-Nox5     ------------------------------------------------------------
Cf-Nox5     ------------------------------------------------------------
Bt-Nox5     ------------------------------------------------------------
Md-Nox5     ------------------------------------------------------------
Xt-Nox5     ------------------------------------------------------------
Tr-Nox5     ------------------------------------------------------------
Tn-Nox5     ------------------------------------------------------------
Gg-Nox5     ------------------------------------------------------------
Ol-Nox5     ------------------------------------------------------------
Dr-Nox5     ------------------------------------------------------------
Sp-Nox5A    IRLREMECDAAEADV--------------------------------------------- 559
Sp-Nox5B    NGDSFKMKSLNSRQS--------------------------------------------- 639
Ag-Nox5     KKRERMMVLREYMRSESERSFDEVQIRKARLQSLGLAYLSPQNKSLAQSFRYMRNKPTII 755
Am-Nox5     KKNDQLIATQGYRRKQSNN-------------------LST------------------- 692
Dm-Nox5     KKRSRLKALRALGRSESESAFDEKRVRRARNNSVGLAYLSPQNKSLAQSFRYMRTKPTII 766
Dd-NoxC     ------------------------------------------------------------
Mg-NoxC     ------------------------------------------------------------
Fg-NoxC     ------------------------------------------------------------
Cc-NoxD     ------------------------------------------------------------
Py-NoxD     ------------------------------------------------------------
Ci-DuoxA    ------------------------------------------------------------
Ci-DuoxC    ------------------------------------------------------------
Ci-DuoxD    ------------------------------------------------------------
Sp-Duox     ------------------------------------------------------------
Xt-Duox1    ------------------------------------------------------------
```

```
Xt-Duox2            ------------------------------------------------------------
Gg-Duox             ------------------------------------------------------------
Tr-Duox             ------------------------------------------------------------
Tn-Duox             ------------------------------------------------------------
Ol-Duox             ------------------------------------------------------------
Dr-Duox             ------------------------------------------------------------
Mm-Duox2            ------------------------------------------------------------
Rn-Duox2            ------------------------------------------------------------
Hs-Duox2            ------------------------------------------------------------
Cf-Duox2            ------------------------------------------------------------
Hs-Duox1            ------------------------------------------------------------
Cf-Duox1            ------------------------------------------------------------
Rn-Duox1            ------------------------------------------------------------
Mm-Duox1            ------------------------------------------------------------
Dm-Duox             ------------------------------------------------------------
Ag-Duox             ------------------------------------------------------------
Am-Duox             ------------------------------------------------------------
Ci-DuoxB            ------------------------------------------------------------
Ce-Duox1            ------------------------------------------------------------
Ce-Duox2            ------------------------------------------------------------


Hs-Nox1             ------------------------------------------------------------
Cf-Nox1             ------------------------------------------------------------
Mm-Nox1             ------------------------------------------------------------
Rn-Nox1             ------------------------------------------------------------
Gg-Nox1             ------------------------------------------------------------
Xt-Nox1             ------------------------------------------------------------
Tn-Nox1             ------------------------------------------------------------
Tr-Nox1             ------------------------------------------------------------
Ol-Nox1             ------------------------------------------------------------
Dr-Nox1             ------------------------------------------------------------
Mm-Nox2             ------------------------------------------------------------
Rn-Nox2             ------------------------------------------------------------
Hs-Nox2             ------------------------------------------------------------
Cf-Nox2             ------------------------------------------------------------
Gg-Nox2             ------------------------------------------------------------
Xt-Nox2             ------------------------------------------------------------
Tn-Nox2             ------------------------------------------------------------
Tr-Nox2             ------------------------------------------------------------
Ol-Nox2             ------------------------------------------------------------
Dr-Nox2             ------------------------------------------------------------
Hs-Nox3             ------------------------------------------------------------
Cf-Nox3             ------------------------------------------------------------
Mm-Nox3             ------------------------------------------------------------
Rn-Nox3             ------------------------------------------------------------
Gg-Nox3             ------------------------------------------------------------
Ci-Nox2             ------------------------------------------------------------
Sp-Nox2A            ------------------------------------------------------------
Sp-Nox2B            ------------------------------------------------------------
Mm-Nox4             ------------------------------------------------------------
Rn-Nox4             ------------------------------------------------------------
Hs-Nox4             ------------------------------------------------------------
```

```
Cf-Nox4         ------------------------------------------------------------
Gg-Nox4         ------------------------------------------------------------
Xt-Nox4         ------------------------------------------------------------
Ol-Nox4         ------------------------------------------------------------
Tr-Nox4         ------------------------------------------------------------
Ci-Nox4         ----------------SNSTISVYDNHEMPTHQENIDDQTCKKTSSTCLSQYSLESESQR 571
Ag-Nox          ------------------------------------------------------------
Pa-NoxA         ------------------------------------------------------------
Mg-NoxA         ------------------------------------------------------------
Fg-NoxA         ------------------------------------------------------------
An-NoxA         ------------------------------------------------------------
Pa-NoxB         ------------------------------------------------------------
Fg-NoxB         ------------------------------------------------------------
Mg-NoxB         ------------------------------------------------------------
Dd-NoxA         ------------------------------------------------------------
Dd-NoxB         ------------------------------------------------------------
At-rbohC        ------------------------------ADMLHGANN--------------------- 693
At-rbohG        -------------------------------DMLNGANS--------------------- 639
At-rbohA        ------------------------------ADSKHWDYI--------------------- 685
At-rbohB        ------------------------------ADIGQANNI--------------------- 639
At-rbohD        ------------------------------ADGGDGN----------------------- 714
At-rbohF        ------------------------------ADETTKK----------------------- 725
At-rbohI        ------------------------------ADVPNQR----------------------- 713
At-rbohE        ------------------------------FSAYCNIDM--------------------- 704
At-rbohH        ------------------------ETRAAGVNPHIEESQ--------------------- 668
At-rbohJ        ------------------------ETRARGANPHVEESQ--------------------- 677
Hs-Nox5         ----------------------------ILLEKHKFCN---------------------- 534
Ol-Nox5         ----------------------------KFAENHRVCN---------------------- 548
Dr-Nox5         ---------------------------------HRYCN---------------------- 429
Sp-Nox5A        ---------------------------VKPTLNHRGANGNLPHGLPRGYSVEREESEDNQ 592
Sp-Nox5B        ---------------------------IDVQEAPRGSTTKRQSLRVRQSLVGREKRSVCG 672
Ag-Nox5         AFKTPSLENCEPRDSTNSIVVSPGVFTQKDAEEGRTAGALPTSGAASSVVAVGSGAASAA 815
Am-Nox5         --------------------------IQSAAEEGRLQMKFEENSKKN---------HNDI 717
Dm-Nox5         AFKTPSMEEREHQVAAG---EANGASPASRAEQGQLSSRMDSADKLQLARLSLSAEGASK 823
Cf-Nox5         ------------------------------------------------------------
Bt-Nox5         ------------------------------------------------------------
Md-Nox5         ------------------------------------------------------------
Xt-Nox5         ------------------------------------------------------------
Tr-Nox5         ------------------------------------------------------------
Tn-Nox5         ------------------------------------------------------------
Gg-Nox5         ------------------------------------------------------------
Dd-NoxC         ------------------------------------------------------------
Mg-NoxC         ------------------------------------------------------------
Fg-NoxC         ------------------------------------------------------------
Cc-NoxD         ------------------------------------------------------------
Py-NoxD         ------------------------------------------------------------
Xt-Duox1        ------------------------------------------------------------
Xt-Duox2        ------------------------------------------------------------
Gg-Duox         ------------------------------------------------------------
Tr-Duox         ------------------------------------------------------------
Tn-Duox         ------------------------------------------------------------
Ol-Duox         ------------------------------------------------------------
Dr-Duox         ------------------------------------------------------------
Mm-Duox2        ------------------------------------------------------------
Rn-Duox2        ------------------------------------------------------------
```

```
Hs-Duox2          ------------------------------------------------------------
Cf-Duox2          ------------------------------------------------------------
Hs-Duox1          ------------------------------------------------------------
Cf-Duox1          ------------------------------------------------------------
Rn-Duox1          ------------------------------------------------------------
Mm-Duox1          ------------------------------------------------------------
Ci-DuoxA          ------------------------------------------------------------
Ci-DuoxC          ------------------------------------------------------------
Ci-DuoxD          ------------------------------------------------------------
Sp-Duox           ------------------------------------------------------------
Dm-Duox           ------------------------------------------------------------
Ag-Duox           ------------------------------------------------------------
Am-Duox           ------------------------------------------------------------
Ci-DuoxB          ------------------------------------------------------------
Ce-Duox1          ------------------------------------------------------------
Ce-Duox2          ------------------------------------------------------------


Hs-Nox1           ------------------------------------------------------------
Cf-Nox1           ------------------------------------------------------------
Mm-Nox1           ------------------------------------------------------------
Rn-Nox1           ------------------------------------------------------------
Gg-Nox1           ------------------------------------------------------------
Xt-Nox1           ------------------------------------------------------------
Tn-Nox1           ------------------------------------------------------------
Tr-Nox1           ------------------------------------------------------------
Ol-Nox1           ------------------------------------------------------------
Dr-Nox1           -----------------------------------------------------WIFTITS 389
Mm-Nox2           ------------------------------------------------------------
Rn-Nox2           ------------------------------------------------------------
Hs-Nox2           ------------------------------------------------------------
Cf-Nox2           ------------------------------------------------------------
Gg-Nox2           ------------------------------------------------------------
Xt-Nox2           ------------------------------------------------------------
Tn-Nox2           ------------------------------------------------------------
Tr-Nox2           ------------------------------------------------------------
Ol-Nox2           ------------------------------------------------------------
Dr-Nox2           ------------------------------------------------------------
Hs-Nox3           ------------------------------------------------------------
Cf-Nox3           ------------------------------------------------------------
Mm-Nox3           ------------------------------------------------------------
Rn-Nox3           ------------------------------------------------------------
Gg-Nox3           ------------------------------------------------------------
Ci-Nox2           ------------------------------------------------------------
Sp-Nox2A          ------------------------------------------------------------
Sp-Nox2B          ------------------------------------------------------------
Mm-Nox4           ----------------------------------------------------FIHSRNYP 405
Rn-Nox4           ----------------------------------------------------FIQSRNYP 405
Hs-Nox4           ----------------------------------------------------FIQSRNYP 405
Cf-Nox4           ----------------------------------------------------VIQSRKYP 411
Gg-Nox4           ----------------------------------------------------IFQQRHYP 370
Xt-Nox4           ----------------------------------------------------KCQQRKNP 398
Ol-Nox4           ----------------------------------------------------VVHQRRYP 383
```

```
Tr-Nox4     ----------------------------------------------------MVQHRKYP 384
Ci-Nox4     NLPKSTTLHSKDQQNLQNETTLYSEDQRNVPKSTALVSEACSTKNDIHLPCNQTVSKQLP 631
Ag-Nox      -----------------------------------------------------VDPYRRI 381
Pa-NoxA     -----------------------------------EGVDPLG-----MYDVALANGQKMP 350
Mg-NoxA     -----------------------------------EGVDPMG-----MYEVALQNGQQMP 376
Fg-NoxA     -----------------------------------DDVDPMG-----MYEVALQNGDQMP 353
An-NoxA     -----------------------------------EGLDPMG-----MYEVALQNGQQMP 348
Pa-NoxB     -----------------------------------GDASKVVGVDQSNDEVDPALRRVLP 397
Fg-NoxB     -----------------------------------GDASKVVGLTGREAEIDPAIRRVLP 388
Mg-NoxB     ------------------------------------DASKVVGVNGENPDVDPALKRVLP 400
Dd-NoxA     -----------------------------------------------ENVLKSPDG--KP 335
Dd-NoxB     -----------------------------------------------EDLKSTQNRGKRR 514
At-rbohC    --------------------------------------------------------PDFP 697
At-rbohG    --------------------------------------------------------PRFP 643
At-rbohA    --------------------------------------------------------PDFP 689
At-rbohB    --------------------------------------------------------TRFP 643
At-rbohD    --------------------------------------------------------LPFP 718
At-rbohF    ---------------------------------------------------------SLP 728
At-rbohI    ---------------------------------------------------------SFP 716
At-rbohE    --------------------------------------------------------INRP 708
At-rbohH    --------------------------------------------------------VLFP 672
At-rbohJ    --------------------------------------------------------ALFP 681
Hs-Nox5     -----------------------------------------------------------I 535
Cf-Nox5     -----------------------LSRSLKMRRSQRRPEVSEKS--------SENHLFCNI 562
Bt-Nox5     -----------------------LSRRLEMKRSQRKPQVSEMS--------SENHQFCNI 570
Md-Nox5     -----------------------LTQSLKLRRSQRKSQKEGPSP------VNENHRFCKI 544
Xt-Nox5     ---------------------QILTQKNRQHHSQVSANDMKCSY-----DMFNRHQFIDR 558
Tr-Nox5     ---------------------SDLSVTYDIEQILLKTSAHTDTEESVKTSDAHSMTLYRF 671
Tn-Nox5     ---------------------PDLQQHQEADGEPEKAAAAAEGPGFGQVWRQSSVLQHQV 433
Gg-Nox5     ---------------------AVLSRVLALPRAEHPASVLGLSP------CFWVLSLYLS 735
Ol-Nox5     -----------------------------------------------------------I 549
Dr-Nox5     -----------------------------------------------------------I 430
Sp-Nox5A    HQH----------------------RTIACQTTFEMKGAKSWRSSLRE---------EKI 621
Sp-Nox5B    KPN----------------------GDVNRKMSLVTLRRNGARHSLDLSKDLGGRPHTGL 710
Ag-Nox5     SSN---------------------PASRPVNYPVG----------------------KPL 832
Am-Nox5     EFD---------------------SISHSLNYTVG----------------------KPL 734
Dm-Nox5     PLEDQTQTGSPSRKSILRRPTFLRSLSASINNRTGGGGGGSTGSSTTNSGGKVTLDAGVM 883
Dd-NoxC     -----------------------------------DGSNFIINNNNNIDQIDLEIGLKPF 928
Mg-NoxC     ------------------------------------------------------GQEVDI 534
Fg-NoxC     -------------------------------------------------------GESEI 456
Cc-NoxD     ------------------------------------------------------AVTDSL 323
Py-NoxD     ------------------------------------------------------TDPTQV 315
Xt-Duox1    ----------------------------------------------------------YP 1315
Xt-Duox2    ----------------------------------------------------------YP 1368
Gg-Duox     ----------------------------------------------------------LP 1254
Tr-Duox     ----------------------------------GRTEASPLMSMSPSAAVSITHALFPP 1311
Tn-Duox     ------------------------------------------------------LGAFP- 1437
Ol-Duox     ------------------------------------------------------LGSYP- 1363
Dr-Duox     -------------------------------------------------------SEDSQ 1314
Mm-Duox2    -------------------------------------------------------CARYP 1328
Rn-Duox2    -------------------------------------------------------SARYP 1328
Hs-Duox2    -------------------------------------------------------CAGYP 1359
Cf-Duox2    -------------------------------------------------------CARYP 1111
Hs-Duox1    -------------------------------------------------------CARYP 1362
Cf-Duox1    -------------------------------------------------------CAKYP 1363
```

```
Rn-Duox1            -------------------------------------------------------CARYP 1358
Mm-Duox1            -------------------------------------------------------CARYP 1362
Ci-DuoxA            ----------------------------------------------------------YP 1386
Ci-DuoxC            ----------------------------------------------------------YP 1251
Ci-DuoxD            ----------------------------------------------------------YP 1325
Sp-Duox             ----------------------------------------------------------LP 1482
Dm-Duox             ----------------------------------------------------------QP 1282
Ag-Duox             ----------------------------------------------------------QP 1283
Am-Duox             ----------------------------------------------------------HP 1289
Ci-DuoxB            ----------------------------------------------------------LP 1305
Ce-Duox1            ----------------------------------------------------------FP 1304
Ce-Duox2            ----------------------------------------------------------FP 1310


                                                ____NADPH1_____
Hs-Nox1             RIEVDGPFGTASEDVFQYEVAVLVGAGIGVTPFASILKSIWYKFQCADHNLK-------- 429
Cf-Nox1             RIEVDGPFGTVSEDVFQYEVVVLVGAGIGVTPFASILKSIWYKFRHEDHNLK-------- 429
Mm-Nox1             RIEVDGPFGTVSEDVFQYEVAVLVGAGIGVTPFASILKSIWYKFQRADNKLK-------- 428
Rn-Nox1             RIEVDGPFGTVSEDVFQYEVAVLVGAGIGVTPFASFLKSIWYKFQRAHNKLK-------- 428
Gg-Nox1             RIKVDGPFGTASEDVFLYEVAMLVGAGIGVTPFASILKSIWYRFQQNDQTLK-------- 430
Xt-Nox1             RLEVDGPFGTASEDVFQYEVSMLVGAGIGVTPFASILKSIWYKFQRDDQRLK-------- 430
Tn-Nox1             KMGVDGPFGTASEDVFDYEVSMLVGAGIGVTPFASILKSIWYKFKESNPKLR-------- 427
Tr-Nox1             KMGVDGPFGTASEDVFDYEVSMLVGAGIGVTPFASILKSIWYKFKESNPKLR-------- 425
Ol-Nox1             KMGVDGPFGTASEDVFDYEVSMLVGAGIGVTPFASIMKSIWYKFKECDPKLR-------- 430
Dr-Nox1             RMGVDGPFGTASEDVFHYEVSMLVGAGIGVTPFASILKSIWYKFKDSDPKLR-------- 441
Mm-Nox2             KIAVDGPFGTASEDVFSYEVVMLVGAGIGVTPFASILKSVWYKYCDNATSLK-------- 435
Rn-Nox2             KIAVDGPFGTASEDVFSYEVVMLVGAGIGVTPFASILKSVWYKYCDNATSLR-------- 435
Hs-Nox2             KIAVDGPFGTASEDVFSYEVVMLVGAGIGVTPFASILKSVWYKYCNNATNLK-------- 435
Cf-Nox2             KIAVDGPFGTASEDVFSYEVVMLVGAGIGVTPFASILKSVWYKYCNNATNLR-------- 435
Gg-Nox2             KIAVDGPFGTASEDVFSYETVMLVGAGIGVTPFASVLKSVWYKYCHDATNLK-------- 435
Xt-Nox2             KIAVDGPFGTASEDVFSYEVAMLVGAGIGVTPFASVLKSVWYRYVNDASTLR-------- 435
Tn-Nox2             KVAIDGPFGTASEDVFRYEVVMLVGAGIGVTPFASILKSVWYKHIQKNQEVF-------- 433
Tr-Nox2             KVAIDGPFGTASEDVFRYEVVMLVGAGIGVTPFASILKSVWYKHIQNNQEVF-------- 433
Ol-Nox2             KVAIDGPFGTASEDVFRYEVVMLVGAGIGVTPFASILKSVWYKHIQNNEGVF-------- 434
Dr-Nox2             KMAVDGPFGTASEDVFRYEAVMLVGAGIGVTPFASVLKSVWYKHVQENQNVF-------- 433
Hs-Nox3             RLAVDGPFGTALTDVFHYPVCVCVAAGIGVTPFAALLKSIWYKCSEAQTPLK-------- 433
Cf-Nox3             RLAVDGPFGTTLTDVFHYPVSVCIAAGIGVTPFASLLKSIWYKC-ESQTQLK-------- 432
Mm-Nox3             RLAVDGPFGGSLADVFHYPVSVCIATGIGVTPFASLLKSVWYKCCESQSLPE-------- 433
Rn-Nox3             RLAVDGPFGGSLADVFHYPVSVCIATGIGVTPFASLLKSVWYKCCESQSLPG-------- 433
Gg-Nox3             RLVVDGPYGSATTDVFHYGVSVCIAAGIGVTPFASILKSIWYKSCNPNTVLV-------- 432
Ci-Nox2             RLAIDGPFGTASEDVFNYPVAICVGSGIGVTPFASLLKSVWYKNLNPEHEMV-------- 448
Sp-Nox2A            RVAVDGPFGTASIDIFKYQVAICVGAGIGVTPFASILKSIWLKSVNNSASLK-------- 446
Sp-Nox2B            RVQVDGPFGTSCTDIFDYDVVMCVSAGIGVTPYASTLKSIWISSRQNFCTLH-------- 420
Mm-Nox4             KLYIDGPFGSPFEESLNYEVSLCVAGGIGVTPFASILNTLLDD---WKPYKL-------- 454
Rn-Nox4             KLYIDGPFGSPFEESLNYEVSLCVAGGIGVTPFASILNTLLDD---WKPYKL-------- 454
Hs-Nox4             KLYIDGPFGSPFEESLNYEVSLCVAGGIGVTPFASILNTLLDD---WKPYKL-------- 454
Cf-Nox4             KLYIDGPFGSPFEESLNYEVSLCVAGGIGVTPFASILNTLLDD---WKPYKL-------- 460
Gg-Nox4             KLYVDGPFGSPFEESLNYEVSLCVAGGIGVTPFASVLNALLDG---WKCYKL-------- 419
Xt-Nox4             KIYVDGPFGSPSEEVFNYQISLCIAGGIGVTPFASVLNRLLDS---WDGYKL-------- 447
Ol-Nox4             TLYVDGPFGSPSEEVFNYDVSLCIAGGIGVTPFACVLNALIPSE-RWQSFRL-------- 434
Tr-Nox4             KIYVDGPFGSPSEDVFNYDVSLCVAGGIGVTPFACMLHTLLDRG--WTHFRL-------- 434
Ci-Nox4             ILCVEGPTGGAMEDIFKYKISMCVAGGIGVTPYASVLNALLKDEDLFSRMKL-------- 683
Ag-Nox              EFLLDGPYPSVMSNMLDCKRILFVGAGVGITPFVTIMRLLLSS----NVDQP-------- 429
Pa-NoxA             ALRIDGPYGAPAEDVFENEIAVLIGTGIGVTPWASILKNIWHLRNGPNPPTR-------- 402
```

Mg-NoxA	MLRIDGPYGAPAEDVFENEIAVLIGTGIGVTPWASILKNIWHLRNGPNPPTR--------	428
Fg-NoxA	ALRIDGPYGAPAEDVFENEIAVLIGTGIGVTPWAAILKNIWHLRNSPNPPRR--------	405
An-NoxA	KLRVDGPYGAPAEDVFENEIAVLIGTGIGVTPWASILKNIWHLRASPDPPRR--------	400
Pa-NoxB	RVYIDGPFGSASEDVFKYEISVLCGAGIGVTPFASILKSIWYRMNYPQKRTR--------	449
Fg-NoxB	RVYVDGPFGSASEDVFKYEVSVLVGAGIGVTPFASILKSIWYRMNYPQKKTR--------	440
Mg-NoxB	RVYVDGPFGSASEDVFKYEIAVLCGAGIGVTPFASILKSIWYRMNYPQKKTR--------	452
Dd-NoxA	ILRIDGPFGAASEEVFKYKQVILVGAGIGVTPFASILKHIKYQMARTYNTTP--------	387
Dd-NoxB	ILKIDGPFGAPAENFFKYRNLVLIGAGIGVTPFSSILRHLKNQNDKQTNADENHL-----	569
At-rbohC	KVLIDGPYGAPAQDYKKYEVVLLVGLGIGATPMISIVKDIVNNIKAKEQAQLNRME----	753
At-rbohG	KIMIDGPYGAPAQDYKKYEVVLLIGLGIGATPMISIIKDIINNTETKEQ--LSQME----	697
At-rbohA	RILIDGPYGAPAQDYKKFEVVLLVGLGIGATPMISIVSDIINNLKGVEEGSNRRQSP---	746
At-rbohB	RLLIDGPYGAPAQDYRNYDVLLLVGLGIGATPLISIIRDVLNNIKNQN---SIERG----	696
At-rbohD	KVLIDGPYGAPAQDYKKYDVVLLVGLGIGATPMISILKDIINNMKGPDRDSDIENN----	774
At-rbohF	KLLIDGPYGAPAQDYRKYDVLLLVGLGIGATPFISILKDLLNNIVKMEEHADSIS-----	783
At-rbohI	ELLIDGPYGAPAQDHWKYDVVLLVGLGIGATPFVSILRDLLNNIIKQQEQAECISGSCSN	776
At-rbohE	KLLVDGPYGAPAQDYRSYDVLLLIGLGIGATPFISILKDLLNNSRDEQTDNEFSRSDFSW	768
At-rbohH	KIFIKGPYGAPAQNYQKFDILLLVGLGIGATPFISILKDMLNHLKPGIPRSGQKYEG---	729
At-rbohJ	RIFIKGPYGAPAQSYQKFDILLLIGLGIGATPFISILKDMLNNLKPGIPKTGQKYEG---	738
Hs-Nox5	KCYIDGPYGTPTRRIFASEHAVLIGAGIGITPFASILQSIMYRHQKRKHTCPSCQHS---	592
Cf-Nox5	KCYIDGPYGTPTRRIFASEHAVLIGAGIGITPFASILQSIMYR---HQKRKNICPS----	615
Bt-Nox5	KCYIDGPYGTPTRRIFASEHAVLIGAGIGITPFASILQSILYR---HQKRKHICPN----	623
Md-Nox5	KCYLDGPYGTPTRRIFASEHAVLIGAGIGITPFASILQSIMYR---HQKRKQHCPN----	597
Xt-Nox5	YCYIDGPYGTPTRRIFTSDHAVLIGAGIGITPFASILQSIMYR---YRMRKQNCPS----	611
Tr-Nox5	QCYVDGPYGTPTRQIFASEHAILIGAGIGITPFASILQSIMYK---YRRRKQNCPN----	724
Tn-Nox5	LRRWD--YGTPTRQIFASEHAILIGAGIGITPFASILQSIMYK---YRRRKQNCPN----	484
Gg-Nox5	QCYIDGPYGTPTRRIFTSEHAVLIGAGIGITPFASILQSIMXQVGPYRQRKQSCPS----	791
Ol-Nox5	KCYVDGPYGTPTRQIFTSEHAVLIGAGIGITPFASILQSIMYRYRLRKQNCPSCNFS---	606
Dr-Nox5	KCYVDGPFGTPTRQIFASEHAILIGAGIGITPFASILQSIMCRYRMRKQNCPNCSYS---	487
Sp-Nox5A	QVFIDGPYGTATRGIFQAEHAILVGAGIGVTPFASILQSIMHRYRVGRQTCPICQHT---	678
Sp-Nox5B	EVILDGPYGAPAQHIMEAEHAVLIGAGIGITPFASILQSINERYKAARKHCPNCNHT---	767
Ag-Nox5	EIYIDGPYGAPSSHIFQAQHAILIATGIGVTPFASILQSIMHRYWKARHCCPRCSYE---	889
Am-Nox5	EIFLDGPYGAPSSHIFQAQHAVLIATGIGVTPFASILQSIMHRYWKARHTCPKCKFS---	791
Dm-Nox5	EIFIDGPYGAPSSHIFGAQHAVLIGTGIGVTPFASILQSIMHRYWKARHSCPRCQFE---	940
Dd-NoxC	RINIDGPFGSSSQYALKQKQVILVGAGIGVSPMASLLKDISLKKQRLQLLNQGDQIALEQ	988
Mg-NoxC	EIGINGPFGAPAQRFYDFNHTILVGAGIGLTPFSGILADLQAKEDRLHGGPTQKLQEQAE	594
Fg-NoxC	EVGINGPFGAPAQRFYDFNHSIIIGAGIGVTPFSGILADLQYNDDLDHGGPNHEVDHHR-	515
Cc-NoxD	QVNIRGPYGAPAQHVGLYERVVLISGGIGSTPFTSICKDLHHRKVKEN------------	371
Py-NoxD	EIKVRGPYGSPAQHVGQFENVVLIGGGVGSTPFASVVKSAHN-WMAAS------------	362
Xt-Duox1	KLYLDGPFGEGHQEWNKFEVSVLVGGGIGVTPFASILKDLVFKSSVNSRIHCK-------	1368
Xt-Duox2	KIYLDGPFGEGHQEWNKYEVSVLVGGGIGVTPFASILKDLVFKSSVNSKIACK-------	1421
Gg-Duox	KLYLDGPFGEGHQEWHKFEVSVLVGGGIGVTPFASILKDLVFKSSINSKLMCK-------	1307
Tr-Duox	KLYLDGPFGEGHQEWDDYEVSVLVGGGIGVTPFASILKDLVFKSSAKSKIRCK-------	1364
Tn-Duox	--KADGPFGEGHQEWDDYEVSILVGGGIGVTPFTSILKDLVFKSSMKSRIRCPTVFF---	1492
Ol-Duox	KLYLDGPFGEGHQEWVDFEVSVLVGGGIGVTPFASILKDLVFKSSIKSKFMCK-------	1416
Dr-Duox	KLYLDGPFGEGHQEWTDFEVSVLVGAGIGVTPFASILKDLVFKSSVKFKFHCK-------	1367
Mm-Duox2	KLYLDGPFGEGHQEWHKFEVSVLVGGGIGVTPFASILKDLVFKSSMGSQMLC--------	1380
Rn-Duox2	KLYLDGPFGEGHQEWHKFEVSVLVGGGIGVTPFASILKDLVFKSSMGAQMLC--------	1380
Hs-Duox2	KLYLDGPFGEGHQEWHKFEVSVLVGGGIGVTPFASILKDLVFKSSLGSQMLC--------	1411
Cf-Duox2	KLYLDGPFGEGHQEWHKFEVSVLVGGGIGVTPFASILKDLVFKSSLGSQMLC--------	1163
Hs-Duox1	KLYLDGPFGEGHQEWHKFEVSVLVGGGIGVTPFASILKDLVFKSSVSCQVFC--------	1414
Cf-Duox1	KLYLDGPFGEGHQEWHKFEVSVLVGGGIGVTPFASILKDLVFKSSVSCQVFC--------	1415
Rn-Duox1	KLYLDGPFGEGHQEWHKFEVSVLVGAGIGVTPFASILKDLVFKSSVSCQVFC--------	1410
Mm-Duox1	KLYLDGPFGEGHQEWHKFEVSVLVGGGIGVTPFASILKDLVFKSSVSCQVFC--------	1414
Ci-DuoxA	KLYLDGPFGEGHQDWYKYDVSVLVGGGIGVTPFASILKDLVSVAQSG--VKIQC------	1438
Ci-DuoxC	KLFLDGPFGEGHQDWYKYEVSVLVGGGIGVTPFASILKDLVNRSQSG--VAITC------	1303

```
Ci-DuoxD            KLYVDGPFGEGHQDWYKYEVAVLVGGGIGVTPFASILKDLVNKSTVG--VGIPC------ 1377
Sp-Duox             KLFLDGPYGEGHQDWYQYEVAVLVGGGIGVTPFASILKDIVNKSTIG--ARVTC------ 1534
Dm-Duox             KIRIEGPFGGGNQDWYKFEVAVMVGGGIGVTPYASILNDLVFGTSTNRYSGVAC------ 1336
Ag-Duox             KIRIEGPFGGGNQDWYKFEVAVMVGGGIGVTPYASILNDLVFGTSTNRYSGVAC------ 1337
Am-Duox             KIRIEGPFGGGNQDWYKFEVAVMVGGGIGVTPYASMLNDLVFGTSTNRYSGVAC------ 1343
Ci-DuoxB            NLYLDGPFGEGHQDWYKYEVSVLVGAGIGVTPFASILKDIVNRTSTKKGSHIPC------ 1359
Ce-Duox1            LIHMKGPYGDGNQEWMDYEVAIMVGAGIGVTPYASTLVDLVQRTSSDSFHRVRC------ 1358
Ce-Duox2            LIHMKGPYGDGNQEWMNYEVAIMVGAGIGVTPYASTLVDLVQKTSSDSFHRVRC------ 1364
                       * ****            *  * ******    *  *


Hs-Nox1             ------------------------------------------------------------
Cf-Nox1             ------------------------------------------------------------
Mm-Nox1             ------------------------------------------------------------
Rn-Nox1             ------------------------------------------------------------
Gg-Nox1             ------------------------------------------------------------
Xt-Nox1             ------------------------------------------------------------
Tn-Nox1             ------------------------------------------------------------
Tr-Nox1             ------------------------------------------------------------
Ol-Nox1             ------------------------------------------------------------
Dr-Nox1             ------------------------------------------------------------
Mm-Nox2             ------------------------------------------------------------
Rn-Nox2             ------------------------------------------------------------
Hs-Nox2             ------------------------------------------------------------
Cf-Nox2             ------------------------------------------------------------
Gg-Nox2             ------------------------------------------------------------
Xt-Nox2             ------------------------------------------------------------
Tn-Nox2             ------------------------------------------------------------
Tr-Nox2             ------------------------------------------------------------
Ol-Nox2             ------------------------------------------------------------
Dr-Nox2             ------------------------------------------------------------
Hs-Nox3             ------------------------------------------------------------
Cf-Nox3             ------------------------------------------------------------
Mm-Nox3             ------------------------------------------------------------
Rn-Nox3             ------------------------------------------------------------
Gg-Nox3             ------------------------------------------------------------
Ci-Nox2             ------------------------------------------------------------
Sp-Nox2A            ------------------------------------------------------------
Sp-Nox2B            ------------------------------------------------------------
Mm-Nox4             ------------------------------------------------------------
Rn-Nox4             ------------------------------------------------------------
Hs-Nox4             ------------------------------------------------------------
Cf-Nox4             ------------------------------------------------------------
Gg-Nox4             ------------------------------------------------------------
Xt-Nox4             ------------------------------------------------------------
Ol-Nox4             ------------------------------------------------------------
Tr-Nox4             ------------------------------------------------------------
Ci-Nox4             ------------------------------------------------------------
Ag-Nox              ------------------------------------------------------------
Pa-NoxA             ------------------------------------------------------------
Mg-NoxA             ------------------------------------------------------------
Fg-NoxA             ------------------------------------------------------------
An-NoxA             ------------------------------------------------------------
Pa-NoxB             ------------------------------------------------------------
```

```
Fg-NoxB      ------------------------------------------------------------
Mg-NoxB      ------------------------------------------------------------
Dd-NoxA      ------------------------------------------------------------
Dd-NoxB      ------------------------------------------------------------
At-rbohC     -------------------------------------------NGTSEPQRSKK------ 764
At-rbohG     -------------------------------------------KGSPQEQQGNK------ 708
At-rbohA     ---------------------------------------IHNMVTPPVSPSRKS------ 761
At-rbohB     ------------------------------------------------TNQHIK------ 702
At-rbohD     ------------------------------------------------NSNNNS------ 780
At-rbohF     ----------------------------------DFSRSSEYSTGSNGDTPRRK------ 803
At-rbohI     SNIS-----------------------------SDHSFSCLNSEAASRIPQTQR------ 801
At-rbohE     N-------------------------------------SCTSSYTTATPTSTHG------ 785
At-rbohH     ---------------------------------------SVGGESIGGDSVSGG------ 744
At-rbohJ     ---------------------------------------SVGGESLGGSSVYGGSSVNGG 759
Hs-Nox5      ---------------------------------------------WIEGVQDN------- 600
Cf-Nox5      ------------------------------------------------------------
Bt-Nox5      ------------------------------------------------------------
Md-Nox5      ------------------------------------------------------------
Xt-Nox5      ------------------------------------------------------------
Tr-Nox5      ------------------------------------------------------------
Tn-Nox5      ------------------------------------------------------------
Gg-Nox5      ------------------------------------------------------------
Ol-Nox5      ---------------------------------------------WCENLKDSD------ 615
Dr-Nox5      ---------------------------------------------WCETIKDNE------ 496
Sp-Nox5A     ---------------------------------------------WLGNIPTDM------ 687
Sp-Nox5B     ---------------------------------------------WVTD-SSSI------ 775
Ag-Nox5      ---------------------------------------------WSSEIPPTI------ 898
Am-Nox5      ---------------------------------------------WASEIPPTV------ 800
Dm-Nox5      ---------------------------------------------WASEIPKSV------ 949
Dd-NoxC      SKNE-------------------------------------------------------- 992
Mg-NoxC      KGGDVRGSTSADAPDGRRIGTSREAEAMPQRTDVDMQAPTSGDQSNTTLQETADADSDSA 654
Fg-NoxC      -----HDSEATAIPQAARRSDSSSSDEATTSDNVPETPTRQGSVGPDLINKEKQPQADKA 570
Cc-NoxD      ------------------(TM7-TM10)--------------------------------
Py-NoxD      ------------------(TM7-TM10)--------------------------------
Xt-Duox1     ------------------------------------------------------------
Xt-Duox2     ------------------------------------------------------------
Gg-Duox      ------------------------------------------------------------
Tr-Duox      ------------------------------------------------------------
Tn-Duox      ------------------------------------------------------------
Ol-Duox      ------------------------------------------------------------
Dr-Duox      ------------------------------------------------------------
Mm-Duox2     ------------------------------------------------------------
Rn-Duox2     ------------------------------------------------------------
Hs-Duox2     ------------------------------------------------------------
Cf-Duox2     ------------------------------------------------------------
Hs-Duox1     ------------------------------------------------------------
Cf-Duox1     ------------------------------------------------------------
Rn-Duox1     ------------------------------------------------------------
Mm-Duox1     ------------------------------------------------------------
Ci-DuoxA     ------------------------------------------------------------
Ci-DuoxC     ------------------------------------------------------------
Ci-DuoxD     ------------------------------------------------------------
Sp-Duox      ------------------------------------------------------------
Dm-Duox      ------------------------------------------------------------
Ag-Duox      ------------------------------------------------------------
```

```
Am-Duox                 ------------------------------------------------------------
Ci-DuoxB                ------------------------------------------------------------
Ce-Duox1                ------------------------------------------------------------
Ce-Duox2                ------------------------------------------------------------


                                            _NADPH2_
Hs-Nox1                 ----------------TKKIYFYWICRETGAFSWFNNLLTSLEQEMEELGK-------VG 466
Cf-Nox1                 ----------------TQTIYFYWICRETGAFAWFNDLLASLECEMEELGK-------VD 466
Mm-Nox1                 ----------------TQKIYFYWICRETGAFAWFNNLLNSLEQEMEELGK-------MD 465
Rn-Nox1                 ----------------TQKIYFYWICRETGAFAWFNNLLNSLEQEMDELGK-------PD 465
Gg-Nox1                 ----------------TKKIYFYWLCRDTGAFTWFNDLLASLEQKMAESGK-------AD 467
Xt-Nox1                 ----------------TKKIYFYWICRETGSFAWFADLLRSLEQEMICSGK-------DG 467
Tn-Nox1                 ----------------TRKIYFYWLCRETHAFEWFADLLQVLEKEMDERGM-------GD 464
Tr-Nox1                 ----------------TRKIYFYWLCRETHAFEWFADLLQVLEKEMDERGM-------VD 462
Ol-Nox1                 ----------------TRKIYFYWLCRETNAFEWFADLLQVLEKEMEERNL-------GD 467
Dr-Nox1                 ----------------TKRIYFYWLCRETHAFEWFADLLQVLEREMEERGM-------RD 478
Mm-Nox2                 ----------------LKKIYFYWLCRDTHAFEWFADLLQLLETQMQERNN-------AN 472
Rn-Nox2                 ----------------LKKIYFYWLCRDTHAFE-FADLLQLLETQMQERNN-------AN 471
Hs-Nox2                 ----------------LKKIYFYWLCRDTHAFEWFADLLQLLESQMQERNN-------AG 472
Cf-Nox2                 ----------------LKKIYFYWLCRDTHAFEWFADLLQLLETQMQERNN-------AG 472
Gg-Nox2                 ----------------LKKIYFYWLCRDTHAFEWFADLLQSLETQMQERNN-------AE 472
Xt-Nox2                 ----------------LKKIYFYWLCRDTQAFEWFADLLQSLETQMQERDN-------AN 472
Tn-Nox2                 ----------------TKKIYFYWLCPETEAFEWFADLLQSLEGQMADKGM-------TD 470
Tr-Nox2                 ----------------TKKIYFYWLCPETEAFEWFADLLQSLEGQMTEKGM-------TD 470
Ol-Nox2                 ----------------TKKVRYIRLLRMSSSYLLFTLMVQKKIIRAWDRHR-------GN 471
Dr-Nox2                 ----------------TKKIYFYWLCPETQAFEWFADLLQSLEKQMSDKNM-------SD 470
Hs-Nox3                 ----------------LSKVYFYWICRDARAFEWFADLLLSLETRMSEQGK-------TH 470
Cf-Nox3                 ----------------LSKVYFYWICRDPKAFEWFADLLLSLETLMSERGK-------AH 469
Mm-Nox3                 ----------------LSKVYFYWICRDAGAFEWFADLLLSLETRMSEQGK-------AH 470
Rn-Nox3                 ----------------LSKVYFYWICRDAAAFEWFADLLLSLETQMSEQGK-------AH 470
Gg-Nox3                 ----------------LQKVYFYWICRDPSTFEWFADLLFLLETKMVEKGK-------ND 469
Ci-Nox2                 ----------------LKKVYFFWICPETHAFEWFGDLLKYLERQLTEIGR-------QD 485
Sp-Nox2A                ----------------LKKVYFFWICPDTNAFEWFSTLLDSIDTHFTEQGK-------PD 483
Sp-Nox2B                ----------------LKRMYFYWICRDTHAFEWFVELLSSLELILRQIDK-------EH 457
Mm-Nox4                 -----------------RRLYFIWVCRDIQSFQWFADLLCVLHNKFWQENR-------PD 490
Rn-Nox4                 -----------------RRLYFIWVCRDIQSFQWFADLLYVLHNKFWQENR-------PD 490
Hs-Nox4                 -----------------RRLYFIWVCRDIQSFRWFADLLCMLHNKFWQENR-------PD 490
Cf-Nox4                 -----------------RRLYFIWVCRDIQSFRWFADLLCVLHNKFWQENR-------PD 496
Gg-Nox4                 -----------------RRLYFIWVCRDVESFRWFADLLCMLHNKLWQENR-------PD 455
Xt-Nox4                 -----------------QRLYFVWVCRDIHSFLWFADLLCLLHRKLWQENR-------PD 483
Ol-Nox4                 -----------------QRLYFVWVCRELQSFYWFAELLCALHEKLWQDNR-------PD 470
Tr-Nox4                 -----------------QRLYFVWVCSELQSFYWFAELLCSVHHKLWQENR-------PD 470
Ci-Nox4                 -----------------KRLYLIWSCKDPRSFSWFASLIRDVQIVLWKRNC-------PD 719
Ag-Nox                  -----------------ARVHLVWIARNLETFLWFSDEIARLQEKFWSQNK-------PD 465
Pa-NoxA                 ----------------LRRVEFIWVCKDTSSFEWFQTLLLSLEEQSAEAARVPGS-SGVE 445
Mg-NoxA                 ----------------LRRVEFLWVCKDTSSFEWFQTLLSSLEQQSTDAAGLPGG-NGVE 471
Fg-NoxA                 ----------------LRRVEFIWVCKDTGSFEWFQTLLSSLEEQSNEAARMPGS-TGVE 448
An-NoxA                 ----------------LRRVEFIWVCKDTTSFEWFQALLSSLEAQSASDAAYQGV-S--E 441
Pa-NoxB                 ----------------LSKVYFFWICRDFGSFEWFRSLLLAIEAQDVDNR---------- 483
Fg-NoxB                 ----------------LSKVYFFWICRDFDSFEWFRSLLLAVEAQDLDHR---------- 474
Mg-NoxB                 ----------------LAKVYFFWICRDFGSFEWFRSLLLAIEAQDVDNR---------- 486
Dd-NoxA                 ---------------LIDKVHFYWICRDRNSFEWFSGLIGELEMEN-HNN---------- 421
Dd-NoxB                 ---------------KINKIYFIWISRQKNSFQWFTDILAELENDERIDS---------- 604
```

```
At-rbohC      ------------ESFRTRRAYFYWVTREQGSFDWFKNIMNEVAERD-----------ANR 801
At-rbohG      ------------ETFKTRRAYFYWVTKEQGTFDWFKNIMNEIAERD-----------KSK 745
At-rbohA      ------------ETFRTKRAYFYWVTREQGSFDWFKNVMDEVTETD-----------RKN 798
At-rbohB      ------------NYVATKRAYFYWVTREQGSLEWFSEVMNEVAEYD-----------SEG 739
At-rbohD      ------------KGFKTRKAYFYWVTREQGSFEWFKGIMDEISELD-----------EEG 817
At-rbohF      ------------RILKTTNAYFYWVTREQGSFDWFKGVMNEVAELD-----------QRG 840
At-rbohI      ------------KTLNTKNAYFYWVTREQGSFDWFKEIMNEIADSD-----------RKG 838
At-rbohE      ------------GKKKAVKAHFYWVTREPGSVEWFRGVMEEISDMD-----------CRG 822
At-rbohH      -----------GGKKFPQRAYFFWVTREQASFDWFKGVMDDIAEYD-----------KTH 782
At-rbohJ      GSVNGGGSVSGGGRKFPQRAYFYWVTREQASFEWFKGVMDDIAVYD-----------KTN 808
Hs-Nox5       --------------MKLHKVDFIWINRDQRSFEWFVSLLTKLEMDQAEEAQ------YGR 640
Cf-Nox5       -CQHSWMDSAQDEDMKLHKVDFMWINRDQRSFEWFVSLLTKLEMDQAEIS------QEGP 668
Bt-Nox5       -CQHSWMESGQDEDMKLHKVDFIWINRDQQSFEWFVSLLTKLEMDQAEET------QVGR 676
Md-Nox5       -CHYSWCEDIRD-DFLIFKVDFIWINRDQKSFEWFVSLLTKLEMDQAEEE------HGGH 649
Xt-Nox5       -CQYSWCETLKENEMDLRKVDFIWINRDQKFFEWFVSLLTKLELDQADEEPD----ETGR 666
Tr-Nox5       -CNYSWCENLKDSDMKLRKVDFIWINRDQKSFEWFVSLLTKLEMDQADEE------PEGR 777
Tn-Nox5       -CNYSWCENLKDSDMKLRKVDFIWINRDQKSFEWFVSLLTKLEMDQADEE------PEGR 537
Gg-Nox5       -CETVWDE-----DMALTKVDFIWINRDQQHFEWFLDLLAALELQQEEQD------PGGR 839
Ol-Nox5       --------------MTLRKVDFIWINRDQKSFEWFVSLLTKLEMDQADEEP------EGR 655
Dr-Nox5       --------------MKLRKVDFIWINRDQKSFEWFVSLLTKLEMDQADEEP------EGR 536
Sp-Nox5A      --------------MRLKKVDFIWINRNQNAFEWFVSLLTQLEMEQAQEP-------FDR 726
Sp-Nox5B      --------------LKTKKVDFVWINRDQHSFEWFISLISAIELEQAEIPA------ADR 815
Ag-Nox5       --------------MNLRKVDFFWINRDQRSFEWFVNLLSQLEIEQAELGSA-----MER 939
Am-Nox5       --------------MHLRKVDFFWINRDQQSFEWFVNLLSQLEMEQAELGDA-----MER 841
Dm-Nox5       --------------MNLRKVDFFWINRDQRSFEWFVNLLSQLEIEQAELGGA-----MER 990
Dd-NoxC       -------ITTKFGLGNLEKVHFFWLNRDQHSFQWFEDLLIDISTNGNSNLP--------- 1036
Mg-NoxC       SSISRPSSSFSSFASDYRRVDFHWMVRDRNHLLWISELLNTVSRSQAWHHRHDAPGEYHL 714
Fg-NoxC       G----------SFAEDYRRVDFHWMVRERNYLLWLSDLLNDVSMSQDWHREHED--KPHL 618
Cc-NoxD       -------------------VDFVWTVPHENDDEWLRSELEPLADG--------------T 703
Py-NoxD       -------------------LDFVWTSPSPEHDAWLVEELLPISRS--------------G 805
Xt-Duox1      ------------------KVYFIWVTRTQHQFEWLTDIIREVEKNDKQ-----------E 1399
Xt-Duox2      ------------------KIYFIWVTRTQRHFEWFADIIREVEENDKC-----------D 1452
Gg-Duox       ------------------KIYFIWVTRTQRQFEWLADIIREVEETDRN-----------E 1338
Tr-Duox       ------------------KVYFIWVTRTQHQFEWVSDIVREVEEMDTQ-----------Q 1395
Tn-Duox       --------------PNILKVYFIWVTRTQRQFEWVSDVIREVEEMDTQ-----------E 1527
Ol-Duox       ------------------KVYFIWVTRTQRQFEWVSDIIREVEEMDTQ-----------E 1447
Dr-Duox       ------------------KVYFLWVTRTQRQFEWLSDIIREVEDMDMQ-----------D 1398
Mm-Duox2      -----------------KKIYFIWVTRTQRQFEWLADIIREVEENDRQ-----------D 1412
Rn-Duox2      -----------------KKIYFIWVTRTQRQFEWLADIIREVEENDSR-----------D 1412
Hs-Duox2      -----------------KKIYFIWVTRTQRQFEWLADIIQEVEENDHQ-----------D 1443
Cf-Duox2      -----------------KKIYFIWVTRTQRQFEWLADIIREVEENDHQ-----------D 1195
Hs-Duox1      -----------------KKIYFIWVTRTQRQFEWLADIIREVEENDHQ-----------D 1446
Cf-Duox1      -----------------KKIYFIWVTRTQRQFEWLADIIREVEENDCQ-----------D 1447
Rn-Duox1      -----------------KKIYFIWVTRTQRQFEWLADIIREVEENDSR-----------D 1442
Mm-Duox1      -----------------KKIYFIWVTRTQRQFEWLADIIREVEENDRQ-----------D 1446
Ci-DuoxA      -----------------KKVYFMWITRDQKQYEWLTDIIQEVESKDAS-----------D 1470
Ci-DuoxC      -----------------KAVYFIWVTRDQNQYEWLTDIIQEVEGKDKK-----------Q 1335
Ci-DuoxD      -----------------KSVYFLWVARDQRQFEWLLDIIEETEKNDAL-----------G 1409
Sp-Duox       -----------------KKVYFIWVTRTQKHYEWLTDIIRDVEDNDTN-----------D 1566
Dm-Duox       -----------------KKVYFLWICPSHKHFEWFIDVLRDVEKKDVT-----------N 1368
Ag-Duox       -----------------KKVYFLWICPSHKHFEWFIDVLRDVEKKDVT-----------N 1369
Am-Duox       -----------------KKVYFLWICPSHKHFEWFIDVLRDVERKDVT-----------D 1375
Ci-DuoxB      -----------------KKIYFIWVTRTQRHFEWLTDIIRELEETAGG-----------D 1391
Ce-Duox1      -----------------RKVYFLWVCSTHKNYEWFVDVLKNVEDQARS-----------G 1390
Ce-Duox2      -----------------RKVYFLWVCSSHKNFEWFVDMLKNVENQAKP-----------G 1396
```

```
                                        * **         *   **

                                                             ___NADPH3____
Hs-Nox1      FLNYRLFLT-GWDSNIVGHAALNF-------DKA-TDIVTGLKQK---TSFGRPMWDNEF 514
Cf-Nox1      FLNYRLFLT-GWDSNIASHATLNF-------DKA-TDILTGLKQK---TSFGRPMWDNEF 514
Mm-Nox1      FLNYRLFLT-GWDSNIAGHAALNF-------DRA-TDILTGLKQK---TSFGRPMWDNEF 513
Rn-Nox1      FLNYRLFLT-GWDSNIAGHAALNF-------DRA-TDVLTGLKQK---TSFGRPMWDNEF 513
Gg-Nox1      FLTYRLFLT-GWDTSIANNAALHF-------DTV-TDTVTGLRQK---TIFGRPRWDTEF 515
Xt-Nox1      FLNYRLFLT-SWDSKIAGHVVIDF-------DHA-TDTVTGLRQK---TSYGRPIWENEF 515
Tn-Nox1      FLTYKLYLT-KWDQSHADHIMVHS-------DQD-IDVVTGLRQK---TYYGRPAWDKEF 512
Tr-Nox1      FLTYKLYLT-KWDDGHVNHIKVYP-------DTD-VDMVTGLRQQ---TNYGRPNWDKEF 510
Ol-Nox1      FLTYKLFLT-GWDQGHTDQVIVHF-------DED-TDVVTGLKQK---THYGRPNWDKEF 515
Dr-Nox1      FLTYKLYLT-GWDQSHADHAMVHF-------DKD-TDIITGLKQK---THYGRPNWDKEF 526
Mm-Nox2      FLSYNIYLT-GWDESQANHFAVHH-------DEE-KDVITGLKQK---TLYGRPNWDNEF 520
Rn-Nox2      FLSYNIYLT-GWDESQANHFAVHH-------DEE-KDVITGLKQK---TLYGRPNWDNEF 519
Hs-Nox2      FLSYNIYLT-GWDESQANHFAVHH-------DEE-KDVITGLKQK---TLYGRPNWDNEF 520
Cf-Nox2      FLSYNIYLT-GWDESQANHFAVHH-------DEE-KDVITGLKQK---TLYGRPNWDNEF 520
Gg-Nox2      FLSYNIYLT-GWDETQATHFVMHH-------EEE-KDVITGLKQK---TLYGRPNWENEF 520
Xt-Nox2      FLVYNIYLT-GWDESQATAFSLHH-------DQE-KDVITGLKQK---TLYGRPNWENEF 520
Tn-Nox2      FLSYNIYLT-RWKEKEAAHFRVHH-------EAE-NDPITGLKQK---TLYGKPNWDHEF 518
Tr-Nox2      FLSYNIYLT-RWKEKEAAHFRVHH-------EAE-NDPITGLKQK---TLYGKPNWDNEF 518
Ol-Nox2      WLATPVARF-GQLKNQAAHLRVHH-------EDE-NDPITGLKQK---TLYGKPNWDNEF 519
Dr-Nox2      FLSYNIYLT-RWKDAEAAHLRVQY-------EAE-DDPITGLKQK---TRYGKPNWDNEF 518
Hs-Nox3      FLSYHIFLT-GWDENQALHIALHW-------DEN-TDVITGLKQK---TFYGRPNWNNEF 518
Cf-Nox3      FLSYHIFLT-SWDENQAVHIALHW-------DEN-TDVVTGLKQK---TFYGRPNWSNEF 517
Mm-Nox3      LLSYHIYLT-GWDENQAIHIALHW-------DES-LDVITGLKQK---AFYGRPNWNDEF 518
Rn-Nox3      LLSYHIYLT-GWDEYQAIHIALHW-------DES-LDVITGLKQK---TFYGRPNWNEEF 518
Gg-Nox3      FLSYHIFLT-GWDENQATHIALHY-------DEK-MDVITGLRQK---TFYGRPNWDSEF 517
Ci-Nox2      LIEYHIYLTRGWDHKQAKAIYAHE-------EDT-HDVITGLEQK---TNYGRPNWDEIF 534
Sp-Nox2A     FLKYYIYLSRGWNNTQAKNIYLQE-------EQE-IDAITGLRQK---THYGRPKWDSNF 532
Sp-Nox2B     LLSYSIYLTRGWDYTQAKNIFMQE-------DRE-IDAVTGLRQK---THYGRPKWDSNF 506
Mm-Nox4      FVNIQLYLSQ--TDGIQK---------------II-GEKYHTLNSR---LFIGRPRWKLLF 530
Rn-Nox4      FVNIQLYLSQ--TDGIQK---------------II-GEKYHTLNSR---LFIGRPRWKLLF 530
Hs-Nox4      YVNIQLYLSQ--TDGIQK---------------II-GEKYHALNSR---LFIGRPRWKLLF 530
Cf-Nox4      YVNIQLYLSQ--TDGIQK---------------II-GEKYQALNSR---LFIGRPRWKLLF 536
Gg-Nox4      YINIQLYLSQ--TDGIQK---------------II-GEKYQALNSR---LLIGRPRWKLLF 495
Xt-Nox4      YLNIQLYLSQ--TNGIQN---------------II-GEKYQALNSR---LSIGRPQWKLLF 523
Ol-Nox4      YLNVKLYVTQ--KDSLQS---------------MS-ELRYRPLAAR---LQVGRPKWKLLF 510
Tr-Nox4      YFNMKLYVSQ--TDSLEN---------------MS-AK-YRPLTSR---LLVGRPRWKLLL 509
Ci-Nox4      LLSVRLHITG--SNTLQS---------------EE-SGDQLLSDIQGCHVAYGRPDVTQVF 762
Ag-Nox       RFWVKLYWTQNYDEHLLA---------------EC-FGDMPSIKSR---MHRGRPNWNDVF 507
Pa-NoxA      FLKIHTYLTQKLDMDTTQNIVLNS------VGSS-VDPLTELKAR---TNFGRPNFGRIF 495
Mg-NoxA      FLKIHSYLTQKLDMDTTQNIVLNS------VGAA-LDPLTELKSR---TNFGRPNFAKLF 521
Fg-NoxA      FLKIHTYLTQKLDIDTAQNIVLNS------VGSQ-MDPLTELQSR---TNFGRPDFPRLF 498
An-NoxA      FLRIHIYLTQRLDQDTTTNIYLNS------VGQE-LDPLTELKSR---TNFGRPDFKRLF 491
Pa-NoxB      -IEIHTYLTAKIKVDDATNIMIND------ANAD-KDTITGLRSP---TNFGRPNWDMIF 532
Fg-NoxB      -IEIHTYLTARIKADDATNIMIND------ANAD-KDTITGLRSP---TNFGRPNWDMIF 523
Mg-NoxB      -IEIHTYLTAKIKADDATNIMIND------ANAD-KDAITGLRAP---TNFGRPNWDMIF 535
Dd-NoxA      FLEIHPYLTGALSAQEIRDVMYGD---------EE-KDLITGFTTP---TQFGRPKWDEIF 469
Dd-NoxB      ILEIHIFLTGALELDDYAKIKN----------AQ-KCHITNLHSK---TLFGRPNFRSIF 650
At-rbohC     VIEMHNYCTSVYEEGDARSALIHMLQSLNHAKNG-VDIVSGTRVM---SHFAKPNWRNVY 857
At-rbohG     VIELHNHCTSVYEEGDVRSALIRMLQSLNYAKNG-LDIVAGTRVM---SHFARPNWKNVY 801
At-rbohA     VIELHNYCTSVYEEGDARSALITMLQSLNHAKHG-VDVVSGTRVM---SHFARPNWRSVF 854
At-rbohB     MIELHNYCTSVYEEGDARSALITMLQSLHHAKSG-IDIVSGTRVR---THFARPNWRSVF 795
```

```
At-rbohD        IIELHNYCTSVYEEGDARVALIAMLQSLQHAKNG-VDVVSGTRVK---SHFAKPNWRQVY 873
At-rbohF        VIEMHNYLTSVYEEGDARSALITMVQALNHAKNG-VDIVSGTRVR---THFARPNWKKVL 896
At-rbohI        VIEMHNYLTSVYEEGDTRSNLLTMIQTLNHAKNG-VDIFSGTKVR---THFGRPKWKKVL 894
At-rbohE        QIELHNYLTSVYDEGDARSTLIKMVQALNHAKHG-VDILSGTRVR---THFARPNWKEVF 878
At-rbohH        VIEMHNYLTSMYEAGDARSALIAMVQKLQHAKNG-VDIVSESRH---------------- 825
At-rbohJ        VIEMHNYLTSMYEAGDARSALIAMVQKLQHAKNG-VDIVSESRIR---THFARPNWRKVF 864
Hs-Nox5         FLELHMYMTSALGKNDMKAIGLQMALDLLANKEK-KDSITGLQTR---TQPGRPDWSKVF 696
Cf-Nox5         FLELHMYMTSALGKNDMKAIGLQMALDLLAKKEK-KDSITGLQTR---TQPGRPDWNKVF 724
Bt-Nox5         FLELHMYMTSALSKNDIKAIGLQMALDLLAKKEK-KDSITGLQTR---TQPGRPDWNKVF 732
Md-Nox5         FLELHMYMTSALSKNDMKAIGLQMALDLLAKKEN-KDSITGLKTR---TQPGRPDWSKVF 705
Xt-Nox5         FLEMHMYMTSALSKNDMKAIGLQMALDLLAQKEK-KDSITGLRTR---TQPGRPDWNKVF 722
Tr-Nox5         FLEMHMYMTSALSKNDMKAIGLQMALDLLAKKEK-RDSITGMRTR---TQPGRPDWGKLF 833
Tn-Nox5         FLEMHMYMTSALSKNDMKAIGLQMALDLLAKKEK-RDSITGLRTR---TQPGRPDWGKV- 592
Gg-Nox5         FLELHLYMTSALGRSDVKAVGLQLALDLLAAKEQ-RDSITGLRTR---TQPGRPDWSQVL 895
Ol-Nox5         FLEMHMYMTSALSKNDMKAIGLQMALDLLAKKEK-KDSITGLRTR---TQPGRPEWGKVF 711
Dr-Nox5         FLEMHMYMTSALSKNDMKAIGLQMALDLLAKKEK-RDSITGLRTR---TQPGRPDWAKVF 592
Sp-Nox5A        FLELHMYMTSAMAKNDMKGIGLQMALDIMHKKGH-RDLITGLKTR---TQPGRPDWNKIF 782
Sp-Nox5B        FLDIHLYMTSALSPSDMKAIGLHVALDLIHKKKK-RDTITGLKTR---TQAGRPDWDEVF 871
Ag-Nox5         FLEMHMYITSALQKTDMKAVGLQLALDLLHEKEK-RDLITGLKTR---TNAGRPNWDKVF 995
Am-Nox5         FLEMHMYITSALQKSDMKAVTLQLAMDLVHQMEK-RDLITGLKTR---TNAGRPNWDKVF 897
Dm-Nox5         FLDMHMYITSALQRTDMKAVGLQLALDLLHEKGK-RDLITGLKTR---TNAGRPNWDKVF 1046
Dd-NoxC         KISINTFNTRVFPKNDVRVFMLWNGLDKLFKAQG-LDPTTNLPFK---THWGRPNWDTIF 1092
Mg-NoxC         DIRMQTHVTQKRKNVSTHVYRWLL----EQHRTP-EHPASPITGLINPTQFGRPDFVSIL 769
Fg-NoxC         DIRINTHVTAKQKKISTHVYRWLL----EMHRTD-EHPASPLTGLLNPTHFGRPDFDLIL 673
Cc-NoxD         ELKLHRYVTR-AKEVD-------------MEAGS-EFITS--------SNTGRPEWDEIF 740
Py-NoxD         TVRLHRHITRSAAEVEP-----------WMLDYD-EVPLK--------TTYKRPDWAAIF 845
Xt-Duox1        LLSVHIYITQLAEKFDFRTTMLYICEQHFQKVKN-QSLMTGLRSV---THFGRPPFAGFF 1455
Xt-Duox2        LVSVHIYITQLAEKFDLRTTMLYICERHFQKVLN-RSLFTGLRSI---THFGRPQFEQFF 1508
Gg-Duox         LVSVHIYITQLAEKFDLRTTMLYICERHFQKVLN-KSLFTGLRSI---THFGRPPFIPFF 1394
Tr-Duox         LVSVHTYITQVAEKFDLRTTMLYVCERRFQKVWN-RSLFTGLRSV---THFGRPPFLSFF 1451
Tn-Duox         LVSVHTYITQVAEKFDLRTTMLYVCERHFQKVWN-RSLFTGLRSV---THFGRPPFLSFF 1583
Ol-Duox         LVSVHTYITQVAEKFDLRTTMLYVCERHFQKVWN-RSLFTGLRSV---THFGRPPFVSFF 1503
Dr-Duox         LVSVHIYITQLPEKFDLRTTMLYVCERHFQKVWN-RSLFTGLRSV---THFGRPPFLAFL 1454
Mm-Duox2        LVSVHIYITQLAEKFDLRTTMLYICERHFQKALN-RSLFTGLRSI---THFGRPPFELFF 1468
Rn-Duox2        LVSVHIYITQLAEKFDLRTTMLYICERHFQKVLN-RSLFTGLRSV---THFGRPPFELFL 1468
Hs-Duox2        LVSVHIYVTQLAEKFDLRTTMLYICERHFQKVLN-RSLFTGLRSI---THFGRPPFEPFF 1499
Cf-Duox2        LVSVHIYITQLAEKFDLRTTMLYICERHFQKVLN-RSLFTGLRSI---THFGRPPFEPFF 1251
Hs-Duox1        LVSVHIYITQLAEKFDLRTTMLYICERHFQKVLN-RSLFTGLRSI---THFGRPPFEPFF 1502
Cf-Duox1        LVSVHIYITQLAEKFDLRTTMLYICERHFQKVLN-RSLFTGLRSI---THFGRPPFEPFF 1503
Rn-Duox1        LVSVHIYITQLAEKFDLRTTMLYICERHFQKVLN-RSLFTGLRSV---THFGRPPFEPFF 1498
Mm-Duox1        LVSVHIYITQLAEKFDLRTTMLYICERHFQKVLN-RSLFTGLRSI---THFGRPPFEPFF 1502
Ci-DuoxA        LLDTHIFITQFPQKFDLRTTMLYICERHFQKVAG-KSLFTGLRAVT---HFGRPEFKSFF 1526
Ci-DuoxC        ILNTHIFITQFPQKFDLRTKMLYICEENFQKIAG-KSLFTGLRAIT---HFGRPDFPDFF 1391
Ci-DuoxD        ILSTHIFITEIPNKFDLRTTMLYVCEQHFKKVSE-KSMFTGLNAVT---HFGRPNFPDFL 1465
Sp-Duox         LVSVHIFVTQFFQKFDLRTTMLYICERHFQKISN-RSLFTGLKSIT---HFGRPQFTSFL 1622
Dm-Duox         VLEIHIFITQFFHKFDLRTTMLYICENHFQRLSK-TSIFTGLKAVN---HFGRPDMSSFL 1424
Ag-Duox         VLEIHIFITQFFHKFDLRTTMLYICENHFQRLSK-TSMFTGLKAVN---HFGRPDMSSFL 1425
Am-Duox         VLEIHIFITQFFHKFDLRTTMLYICENHFQRLSK-KSIFTGLKAIN---HFGRPDMTSFL 1431
Ci-DuoxB        LVSTHIYITQFANKYDLRTTMLYICERYFQKVAN-KSMFTGLKAIT---HFGRPQFEAFL 1447
Ce-Duox1        ILETHIFVTQTFHKFDLRTTMLYICEKHFRATNSGISMFTGLHAKN---HFGRPNFKAFF 1447
Ce-Duox2        ILETHIFVTQMFHKFDLRTTMLYICEKHFRATNSGISMFTGLHAKN---HFGRPNFKAFF 1453
                                                                  *** *
```

<u>NADPH4</u>

```
Hs-Nox1     STIATSHP--------------------------KSVVGVFLCGPRTLAKSLRKCCHRYS 548
Cf-Nox1     STIANAHP--------------------------RSVVGVFLCGPQTLAKSLSKCCCQYS 548
Mm-Nox1     SRIATAHP--------------------------KSAVGVFLCGPRTLAKSLRKRCQRYS 547
Rn-Nox1     SRIATAHP--------------------------KSVVGVFLCGPPTLAKSLRKCCRRYS 547
Gg-Nox1     SAVATAHP--------------------------RSVVGVFLCGPEALAKVLRRSCHQHS 549
Xt-Nox1     SKVAEWHP--------------------------KSTVGVFLCGPQALGKTLKQCCHQYS 549
Tn-Nox1     EQVRKENP--------------------------TSVVGTFLCGPEALAEVLEKKCVKYS 546
Tr-Nox1     EQVRKENP--------------------------TSVVGTFLCGPEALGEVLAKKCGKYS 544
Ol-Nox1     DQVRKENP--------------------------ASVVGTFLCGPAALAKVLQKKCAKYS 549
Dr-Nox1     EQVRQENP--------------------------SSVVGTFLCGPQALAKDLEKKCVKYS 560
Mm-Nox2     KTIASEHP--------------------------NTTIGVFLCGPEALAETLSKQSISNS 554
Rn-Nox2     KTIASQHP--------------------------NTRIGVFLCGPEALAKTLSKQSISNS 553
Hs-Nox2     KTIASQHP--------------------------NTRIGVFLCGPEALAETLSKQSISNS 554
Cf-Nox2     KTIASQHP--------------------------NTRIGVFLCGPEALAETLSKQCISNS 554
Gg-Nox2     KTIARQHP--------------------------GSRIGVFLCGPEGLADTLNKQSISNS 554
Xt-Nox2     KTIANAHT--------------------------SSRVGVFLCGPESLAETLNKQSIANS 554
Tn-Nox2     ASIASQHP--------------------------RSKVGVFLCGPPKLGQSLQKQCLSYS 552
Tr-Nox2     TNIASKHP--------------------------GSKVGVFLCGPPQLGKSLQKQCLSHS 552
Ol-Nox2     TNIASTHP--------------------------GSKVGVFLCGPPMLGKSLEKESISHT 553
Dr-Nox2     SLIASQHP--------------------------GTKVGVFLCGPTALGKALSKQCLSHT 552
Hs-Nox3     KQIAYNHP--------------------------SSSIGVFFCGPKALSRTLQKMCHLYS 552
Cf-Nox3     RQLAYAHP--------------------------SSSIGVFFCGPKALSKTLQRMCHLYS 551
Mm-Nox3     KQIAYNHP--------------------------SSSIGVFFCGSKAMSKTLQKMCRLYS 552
Rn-Nox3     KQIAYNHP--------------------------SSSIGVFFCGPKAMSKTLQKMCRLYS 552
Gg-Nox3     KQLAENHP--------------------------SNSIGVFFCGPKNLSKILQKMCSSYS 551
Ci-Nox2     SKTARDYP--------------------------NTHIGVFFCGVAALSAKLHKMSNKHS 568
Sp-Nox2A    KMIAEENPG-----RVS---------------------SVFFCGPKALSSVLHENANKFT 566
Sp-Nox2B    SYIAEKNP------RVSRI--------------WNATIGVFFCGPKSLSTILHQSCNKHT 546
Mm-Nox4     DEIAKCNRGK--------------------------TVGVFCCGPSSISKTLHSLSNRNN 564
Rn-Nox4     DEIAKCNRGK--------------------------TVGVFCCGPSSISKTLHNLSNRNN 564
Hs-Nox4     DEIAKYNRGK--------------------------TVGVFCCGPNSLSKTLHKLSNQNN 564
Cf-Nox4     DEIAKCNRGK--------------------------TVGVFCCGPNSISKTLHKLSNRNN 570
Gg-Nox4     DEIAKYNRRK--------------------------TIGVFCCGPSKMSKILHKLSNSSN 529
Xt-Nox4     EEVAKSSRGK--------------------------TVGVFCCGPKGISKELHKLCNSAN 557
Ol-Nox4     DEIGKSNKDK--------------------------RVGVFCCGPKGISRTLHRLCNSAK 544
Tr-Nox4     NELGKTNKHK--------------------------RIGVFCCGPKAISRTLHRFCNSFQ 543
Ci-Nox4     EEIRTAAQYQR------------------------STVGVFCCGHRLLVSSVKHHCLKTK 798
Ag-Nox      IDLVTLYPKK--------------------------SVSVFSCGPKELTKEIRLKCKEYS 541
Pa-NoxA     QSMSEGIQNRTYLNGLEGN--------------MRTTVGVYFCGPSAAARDIKKAAKAAS 541
Mg-NoxA     ASMRDGIMDRTYLSGLEGS--------------MKTTVGVYFCGPSAAARDIKAACKTAS 567
Fg-NoxA     TTMRNGILDRTYLNGLESH--------------IRTTVGVYFCGPSAAARDIKLACKAAT 544
An-NoxA     TAMRNGLQDQSYMRGLHTH--------------SRTEIGVYFCGPNVAARQIKAAASSAS 537
Pa-NoxB     RGIRK------------LH--------------TPAEAGVFFCGPKGLGSQLHVFCNKYS 566
Fg-NoxB     RGIRK------------IH--------------SPAEAGVFYGGPKGLGSSLHTYCNKYT 557
Mg-NoxB     RGIRK------------LH--------------TPAEAGVFFCGPKGLGSTLHIFCNKYS 569
Dd-NoxA     ADHAL------------RY--------------AEKDVGVFFCGPKLLSKSLYKASTHYT 503
Dd-NoxB     NQLTQ------------LH--------------QREKIGVFYCGNKALGKNIIKNCNKFN 684
At-rbohC    KRIAMDHPNT--------------------------KVGVFYCGAPALTKELRHLALDFT 891
At-rbohG    KQIAMDHPGA--------------------------NVGVFYCGAPVLTKELRQLALEFT 835
At-rbohA    KRIAVNHPKT--------------------------RVGVFYCGAAGLVKELRHLSLDFS 888
At-rbohB    KHVAVNHVNQ--------------------------RVGVFYCGNTCIIGELKRLAQDFS 829
At-rbohD    KKIAVQHPGK--------------------------RIGVFYCGMPGMIKELKNLALDFS 907
At-rbohF    TKLSSKHCNA--------------------------RIGVFYCGVPVLGKELSKLCNTFN 930
At-rbohI    SKISTKHRNA--------------------------RIGVFYCGVPSLGKELSTLCHEFN 928
At-rbohE    SSIARKHPNS--------------------------TVGVFYCGIQTVAKELKKQAQDMS 912
```

```
At-rbohH            SLSSYKYLN--------------------------------YISTP-------------- 839
At-rbohJ            SELSNKHETS--------------------------RIGVFYCGSPTLVRPLKSLCQEFS 898
Hs-Nox5             QKVAAEKKG---------------------------KVQVFFCGSPALAKVLKGHCEKFG 729
Cf-Nox5             QKVAAEKK---------------------------GKVQVFFCGSPALAKVLKGHCEQFG 757
Bt-Nox5             QKVAAEKK---------------------------GKVQVFFCGSPALAKILKGHCEQFS 765
Md-Nox5             QKVAAEKK---------------------------GKVQVFFCGSPALAKVLKSHCEQLN 738
Xt-Nox5             QKIEQENK---------------------------GKVQVFFCGSPALAKIIKAHCEKFN 755
Tr-Nox5             QKVSEEKK---------------------------GKVHVFYCGSPALAKVIKAQCEHYK 866
Tn-Nox5             ------------------------------------------------------------
Gg-Nox5             GRVAEERK---------------------------GKVHVFFCGSPALAKVVRMHCERFG 928
Ol-Nox5             QKLSKENKG---------------------------KVHVFYCGAPSLAKAIKAQCERFG 744
Dr-Nox5             QKVSEEKKG---------------------------KVHVFYCGSPALAKVIKAQCERFG 625
Sp-Nox5A            TQIAREKKG---------------------------KVQVFFCGSPTLAKIIKKSCEKFN 815
Sp-Nox5B            QNLKQQHKG---------------------------KITVFFCGSPALGKVLSTKCLQYQ 904
Ag-Nox5             KQIQDQKKG---------------------------KVTVFYCGPPQLAKTLRYKCDQFG 1028
Am-Nox5             KHLQDQKKG---------------------------KVTIFYCGPPQLARILRYKCDQFG 930
Dm-Nox5             KQLQAQQKG---------------------------KVTVFYCGPPQLAKTLRYKCDQYG 1079
Dd-NoxC             QYYSK------------KY-------------SGESISVFFCCGPSQLSKELYEKCRYYT 1126
Mg-NoxC             DRHYDDMRKYKAGLVARASRGGGGEDDAASVADDEVKVGVFFCGTPIVGEILADRCKALT 829
Fg-NoxC             DEHYEEMLKFRASKRTSTR----NKEDENYEEDEELKVGVFYCGAPVVGEILADKCRELT 729
Cc-NoxD             GKIAAEAPSNS-------------------------VVGVFFCGPHKMGDSVQSAMRRAE 775
Py-NoxD             AGITERSRSGS-------------------------VVGVFFCGPHPMSKSIQDGIARAT 880
Xt-Duox1            SSLQDVHPKVK-------------------------KIGVFSCGPPGMTKNVENACRKLN 1490
Xt-Duox2            ISLQEVHPEVR-------------------------KIGVFSCGPPGMTKNVEKACQMLN 1543
Gg-Duox             DSLQEVHPEVH-------------------------KIGVFSCGPPGMTKSVEKACQQLN 1429
Tr-Duox             NSLQDVHPEVG-------------------------KIGVFSCGPPGLTKNVEKACQRMN 1486
Tn-Duox             NSLQDVHPKVG-------------------------KMGVFSCGPPGLTKNVEKACQRMN 1618
Ol-Duox             NSLQEVHPEVG-------------------------KMGVFSCGPPGLTKNVEKACQQMN 1538
Dr-Duox             SSLQEVHPEVE-------------------------KVGVFSCGPPGLTKNVEKACQQMN 1489
Mm-Duox2            NSLQEVHPQVR-------------------------KIGVFSCGPPGMTKNVEKACQLIN 1503
Rn-Duox2            DSLQEVHPQVH-------------------------KIGVFSCGPPGMTKNVEKACQLIN 1503
Hs-Duox2            NSLQEVHPQVR-------------------------KIGVFSCGPPGMTKNVEKACQLVN 1534
Cf-Duox2            KSLQEVHPQVP-------------------------KIGVFSCGP----RNDQECREGLS 1282
Hs-Duox1            NSLQEVHPQVR-------------------------KIGVFSCGPPGMTKNVEKACQLIN 1537
Cf-Duox1            KSLQEVHPQVR-------------------------KIGVFSCGPPGMTKNVEKACQLIN 1538
Rn-Duox1            NSLQEVHPQVR-------------------------KIGVFSCGPPGMTKNVEKACQLIN 1533
Mm-Duox1            NSLQEVHPQVR-------------------------KIGVFSCGPPGMTKNVEKACQLIN 1537
Ci-DuoxA            VSLTEEHAEVE-------------------------KFGVFSCGPPPMTSCVEQTCAKLN 1561
Ci-DuoxC            VTLGEEHSSVE-------------------------TFGVFSCGPPPMTEGVEKACAKLN 1426
Ci-DuoxD            KTLSWKHSEVK-------------------------KIGVFSCGPPSMTESVESACLKAN 1500
Sp-Duox             QSLEDEHPGVG-------------------------KIGVFSCGPPGMTGGVEQACVDLN 1657
Dm-Duox             KFVQKKHSYVS-------------------------KIGVFSCGPRPLTKSVMSACDEVN 1459
Ag-Duox             KFVQKKHSYVS-------------------------KIGVFSCGPRPLTKSVMSACDEVN 1460
Am-Duox             KFVQKKHSYVS-------------------------KIGVFSCGPRPLTKSVMSSCDEVN 1466
Ci-DuoxB            DSLQTKHKEVR-------------------------TLGVFSCGPPGLTNGVEDACRNLN 1482
Ce-Duox1            QFIQSEHKEQS-------------------------KIGVFSCGPVNLNESIAEGCADAN 1482
Ce-Duox2            QFIQSEHKEQS-------------------------EIGVFSCGPVNLNESIAEGCADAN 1488
                                                           ** **        *


Hs-Nox1             SLDP---------------------------------RKVQFYFNKENF----- 564
Cf-Nox1             SLDP---------------------------------RKVQFYFNKENF----- 564
Mm-Nox1             SLDP---------------------------------RKVQFYFNKETF----- 563
Rn-Nox1             SLDP---------------------------------RKVQFYFNKETF----- 563
```

```
Gg-Nox1      SLDP---------------------------------RKVKFYFNKENF----- 565
Xt-Nox1      SLDP---------------------------------RKVQFYFNKENF----- 565
Tn-Nox1      DVDP---------------------------------RKTKFYFNKENF----- 562
Tr-Nox1      DVDP---------------------------------RKTKFYFNKENF----- 560
Ol-Nox1      DVDP---------------------------------RKTKFYFNKENF----- 565
Dr-Nox1      DVDP---------------------------------RRTKFYFNKENF----- 576
Mm-Nox2      ESGP---------------------------------RGVHFIFNKENF----- 570
Rn-Nox2      ESGP---------------------------------RGVHFIFNKENF----- 569
Hs-Nox2      ESGP---------------------------------RGVHFIFNKENF----- 570
Cf-Nox2      ESGP---------------------------------RGVHFIFNKENF----- 570
Gg-Nox2      EADP---------------------------------RGVHFIFNKENF----- 570
Xt-Nox2      TVDP---------------------------------RGVHFIFNKENF----- 570
Tn-Nox2      GAD------------------------------------VKFIFNKENF----- 565
Tr-Nox2      EAD------------------------------------VKFIFNKENF----- 565
Ol-Nox2      EAG------------------------------------VKFIFNKENF----- 566
Dr-Nox2      EGG------------------------------------TEFIFNKENF----- 565
Hs-Nox3      SADP---------------------------------RGVHFYYNKESF----- 568
Cf-Nox3      SADP---------------------------------RGVHFYYNKESF----- 567
Mm-Nox3      SVDP---------------------------------RGVHFYYNKENF----- 568
Rn-Nox3      SSDP---------------------------------RGVHFYYNKENF----- 568
Gg-Nox3      TVDP---------------------------------RGVQFHYNEESF----- 567
Ci-Nox2      GGG------------------------------------VYFHYNKENF----- 581
Sp-Nox2A     SLTP---------------------------------DGAKFFYNKENF----- 582
Sp-Nox2B     SDES---------------------------------DGTRFVYYKENF----- 562
Mm-Nox4      ---S---------------------------------YGTKFEYNKESFS---- 578
Rn-Nox4      ---S---------------------------------YGTKFEYNKESFS---- 578
Hs-Nox4      ---S---------------------------------YGTRFEYNKESFS---- 578
Cf-Nox4      ---S---------------------------------YGTRFEYNKESFS---- 584
Gg-Nox4      ---P---------------------------------YGTRFEYNKESFS---- 543
Xt-Nox4      ---Q---------------------------------YGTTFEYNKESFT---- 571
Ol-Nox4      ---S---------------------------------SGATFEFNKESFS---- 558
Tr-Nox4      ---S---------------------------------SETVFEFNKES------ 555
Ci-Nox4      ---S---------------------------------SKVKFLFNKEAF----- 811
Ag-Nox       ---K---------------------------------HGCKLSYFHEGFG---- 555
Pa-NoxA      ---S---------------------------------SEVRFRFWKEHF----- 554
Mg-NoxA      ---V---------------------------------NEVEFRFWKEHF----- 580
Fg-NoxA      ---V---------------------------------PDVDFRFWKEHF----- 557
An-NoxA      ---T---------------------------------NEVKFKFWKEHF----- 550
Pa-NoxB      ---E---------------------------------PGFNFVWGKENF----- 579
Fg-NoxB      ---E---------------------------------PGFSFVWGKENF----- 570
Mg-NoxB      ---E---------------------------------PDFAFVWGKENF----- 582
Dd-NoxA      --KT---------------------------------TTCRFHYNKENF----- 517
Dd-NoxB      --GK---------------------------------NNCHLIFHKENF----- 698
At-rbohC     HKTS-----------------------------------TRFSFHKENF----- 905
At-rbohG     HKTS-----------------------------------TRFSFHKENF----- 849
At-rbohA     HKTS-----------------------------------TKFIFHKENF----- 902
At-rbohB     RKTT-----------------------------------TKFEFHKENF----- 843
At-rbohD     RKTT-----------------------------------TKFDFHKENF----- 921
At-rbohF     QKGS-----------------------------------TKFEFHKEHF----- 944
At-rbohI     QTGI-----------------------------------TRFDFHKEQF----- 942
At-rbohE     QKTT-----------------------------------TRFEFHKEHF----- 926
At-rbohH     ------------------------------------------------------
At-rbohJ     LESS-----------------------------------TRFTFHKENF----- 912
Hs-Nox5      -----------------------------------------FRFFQENF----- 737
Cf-Nox5      -----------------------------------------FKFFQENF----- 765
```

```
Bt-Nox5      -----------------------------------------FKFFQENF----- 773
Md-Nox5      -----------------------------------------FKFFKENF----- 746
Xt-Nox5      -----------------------------------------FKFFKENF----- 763
Tr-Nox5      -----------------------------------------FNFYKENF----- 874
Tn-Nox5      ------------------------------------------------------
Gg-Nox5      -----------------------------------------FRFFKENF----- 936
Ol-Nox5      -----------------------------------------FNFYKENF----- 752
Dr-Nox5      -----------------------------------------FHFYKENF----- 633
Sp-Nox5A     -----------------------------------------FSFHKENF----- 823
Sp-Nox5B     -----------------------------------------MEFRKENF----- 912
Ag-Nox5      -----------------------------------------FQFRKEVF----- 1036
Am-Nox5      -----------------------------------------FNFRKESF----- 938
Dm-Nox5      -----------------------------------------FAFRKECF----- 1087
Dd-NoxC      -----------------------CLKT----------GGTKFYFHKENF----- 1142
Mg-NoxC      ---------------------ARGRHDGS---------KIEYHFMIEVFN---- 849
Fg-NoxC      ---------------------LRGWQDGIHEL-----GRIEWLVNYDGYDDHIN 757
Cc-NoxD      INSNLRGAYLRSTKEKTLMKDLGLPQRGLIKMLMGTGCSVRFVFREENFG---- 825
Py-NoxD      ALSLARG-YRRGAIGLDGSREMRT------------------------------ 903
Xt-Duox1     -----------------------KRDE------------SYFVHHYENF----- 1504
Xt-Duox2     -----------------------KENQ------------AHFSHHFENF----- 1557
Gg-Duox      KKDQ-----------------------------------AYFAHQ--------- 1439
Tr-Duox      KRDQ-----------------------------------AHFIHHYENF----- 1500
Tn-Duox      KRDQ-----------------------------------THFIHHYENF----- 1632
Ol-Duox      KRDQ-----------------------------------AHFIHHYENF----- 1552
Dr-Duox      KRDQ-----------------------------------THFVHHYENF----- 1503
Mm-Duox2     RQDR-----------------------------------AHFVHHYENF----- 1517
Rn-Duox2     RQDR-----------------------------------AHFVHHYENF----- 1517
Hs-Duox2     RQDR-----------------------------------AHFMHHYENF----- 1548
Cf-Duox2     AHQQ-----------------------------------AGPGPLR-------- 1293
Hs-Duox1     RQDR-----------------------------------THFSHHYENF----- 1551
Cf-Duox1     RQDR-----------------------------------THFSHHYENF----- 1552
Rn-Duox1     KQDR-----------------------------------THFSHHYENF----- 1547
Mm-Duox1     RQDR-----------------------------------THFSHHYENF----- 1551
Ci-DuoxA     K-YEGAS----------------------------------F------------ 1568
Ci-DuoxC     K-YEGPT----------------------------------FSHHFENF----- 1440
Ci-DuoxD     K-HKGPM----------------------------------YAHHFENF----- 1514
Sp-Duox      K-FDGAA----------------------------------FIHHYENF----- 1671
Dm-Duox      KTRKLPY----------------------------------FIHHFENFG---- 1475
Ag-Duox      KSRKWPY----------------------------------FIHHFENFG---- 1476
Am-Duox      KGRRLPY----------------------------------FIHHFENFG---- 1482
Ci-DuoxB     K-LNKAR----------------------------------FNHFYENF----- 1496
Ce-Duox1     RQRDAPS----------------------------------FAHRFETF----- 1497
Ce-Duox2     RQRDAPS----------------------------------FAHRFETF----- 1503
                                                      *    * *
```
