# Supplementary material for: Molecular evolution of the reactive oxygen-generating NADPH oxidase (Nox/Duox) family of enzymes
Source: BMC Evol Biol. 2007 Jul 6;7:109. doi: 10.1186/1471-2148-7-109 (PMC1940245; doi:10.1186/1471-2148-7-109)
Supplement: Additional File 10 — Abbreviation of gene names in Figure 3. Names of the marker genes used in Figures 3 are provided. [file 1471-2148-7-109-S10.pdf]

Additional File 10

Abbreviation of gene names in Figure 3

Names of the marker genes used in Figures 3, *B-G* are described.

[**Figure 3B**] TSPAN6 (tetraspanin 6), SPRX2 (sushi-repeat-containing protein, X-linked 2), SYTL4 (synaptotagmin-like 4), CSTF2 (cleavage stimulation factor, 3' pre-RNA, subunit 2, 64kDa), XKRX (XK, Kell blood group complex subunit-related, X-linked), TRM12 (tRNA methyltranferase 12 homolog).

[**Figure 3C**] PRRG1 (proline rich G-carboxyglutamic acid 1), LANCL3 (LanC lantibiotic synthetase component C-like 3), XK (X-linked Kx blood group), DYNLT3 (dynein, light chain, Tctex-type 3) , SYTL5 (synaptotagmin-like 5), RPGR (retinitis pigmentosa GTPase regulator).

[**Figure 3D**] TIAM2 (T-cell lymphoma invasion and metastasis 2), TFBIM (transcription factor B1, mitochondrial), CLDN20 (claudin 20), TLK2 (Serine/threonine-protein kinase tousled-like 2), FILIP1 (filamin A interacting protein 1), TMEM30A (transmembrane protein 30A), COL12A1 (collagen, type XII, alpha 1).

[**Figure 3E**] CTSC (cathepsin C), GRM5 (glutamate receptor, metabotropic 5) , TYR (tyrosinase), NAALAD1 (N-acetylated-alpha-linked acidic dipeptidase 1), NAALAD2

(N-acetylated-alpha-linked acidic dipeptidase 2), CHORDC1 (cysteine and histidine-rich domain-containing 1).

[**Figure 3F**] CORO2B (coronin, actin binding protein, 2B), ANP32A acidic (leucine-rich) nuclear phosphoprotein 32 family, member A), STEPSP1 (sperm equatorial segment protein 1), GLCE (D-glucuronyl C5-epimerase), PAQR5 (progestin and adipoQ receptor family member V), KIF23 (kinesin family member 23), RPGRIP1 (X-linked retinitis pigmentosa GTPase regulator-interacting protein 1), CHRM2 (cholinergic receptor, muscarinic 2), PTHLH (parathyroid hormone-like hormone).

[**Figure 3G**] SORD (sorbitol dehydrogenase), NIP1 (Numb-interacting protein 1), NIP2 (Numb-interacting protein 2), SLC28A2 (solute carrier family 28 member 2), GATM (glycine amidinotransferase), SPATA5L1 (spermatogenesis associated 5-like 1), APBA2 (Amyloid beta A4 precursor protein-binding family A member 2).
