# Supplementary material for: Molecular evolution of the reactive oxygen-generating NADPH oxidase (Nox/Duox) family of enzymes
Source: BMC Evol Biol. 2007 Jul 6;7:109. doi: 10.1186/1471-2148-7-109 (PMC1940245; doi:10.1186/1471-2148-7-109)
Supplement: Additional File 3 — Supplemental Figure S3. The figure provided shows substitution rates of vertebrate Nox/Duox and Nox regulatory proteins [file 1471-2148-7-109-S3.pdf]

Additional file 3

Supplemental Figure S3. Substitution rates of vertebrate Nox/Duox and Nox regulatory proteins

The amino acid substitution rates per site of vertebrate Noxes (*A*) and of their regulatory proteins (*B*) are shown. Rate 1 (R1) indicates substitution rate per site between species A and B. Rate 2 (R2) indicates substitution rate per site, per 10^9 years. Myr means 10^6 years.

A

species A	species B	Myr	Nox1		Nox2		Nox3		Nox4		Nox5		Duox	
			substitutions per site (**R1**)	**R2**	substitutions per site (**R1**)	**R2**	substitutions per site (**R1**)	**R2**	substitutions per site (**R1**)	**R2**	substitutions per site (**R1**)	**R2**	substitutions per site (**R1**)	**R2**
C. famiriaris (dog)	*H. sapiens* (human)	83	0.065	0.78	0.040	0.48	0.065	0.78	0.070	0.84	0.065	0.78	0.030	0.36
M. musculus (mouse)	*H. sapiens* (human)	91	0.080	0.88	0.035	0.38	0.100	1.10	0.050	0.55			0.020	0.22
R. norvegicus (rat)	*H. sapiens* (human)	91	0.090	0.99	0.035	0.38	0.095	1.04	0.055	0.60			0.025	0.27
G. gallus (chicken)	*H. sapiens* (human)	310	0.165	0.53	0.080	0.26	0.135	0.44	0.115	0.37	0.195	0.63	0.105	0.34
G. gallus (chicken)	*M. musculus* (mouse)	310	0.175	0.56	0.085	0.27	0.160	0.52	0.130	0.42			0.105	0.34
G. gallus (chicken)	*R. norvegicus* (rat)	310	0.170	0.55	0.085	0.27	0.160	0.52	0.130	0.42			0.110	0.35
X. tropicalis (frog)	*H. sapiens* (human)	360	0.180	0.50	0.100	0.28			0.175	0.49	0.170	0.47	0.130	0.36
X. tropicalis (frog)	*M. musculus* (mouse)	360	0.195	0.54	0.100	0.28			0.175	0.49				
X. tropicalis (frog)	*R. norvegicus* (rat)	360	0.190	0.53	0.100	0.28			0.175	0.49				
D. rerio (zebrafish)	*H. sapiens* (human)	450	0.205	0.46	0.160	0.36							0.140	0.31
T. rubripes (fugu)	*H. sapiens* (human)	450	0.210	0.47	0.160	0.36			0.205	0.46	0.170	0.38	0.145	0.32
T. nigroviridis (tetraodon)	*H. sapiens* (human)	450	0.215	0.48	0.160	0.36								
D. rerio (zebrafish)	*X. tropicalis* (frog)	450	0.210	0.47	0.170	0.38							0.150	0.33
T. rubripes (fugu)	*X. tropicalis* (frog)	450	0.205	0.46	0.170	0.38			0.205	0.46			0.150	0.33
T. nigroviridis (tetraodon)	*X. tropicalis* (frog)	450	0.205	0.46	0.170	0.38								
D. rerio (zebrafish)	*G. gallus* (chicken)	450	0.210	0.47	0.160	0.36							0.150	0.33
T. rubripes (fugu)	*G. gallus* (chicken)	450	0.210	0.47	0.150	0.33			0.220	0.49				
T. nigroviridis (tetraodon)	*G. gallus* (chicken)	450	0.210	0.47	0.155	0.34								
B. taurus (cow)*	*H. sapiens* (human)	92									0.065	0.71		
B. taurus (cow)*	*C. famiriaris* (dog)	83									0.050	0.60		

B

species A	species B	Myr	p47*phox*		NOXO1		p67*phox*		NOXA1		p22*phox*	
			substitutions per site (**R1**)	**R2**	substitutions per site (**R1**)	**R2**	substitutions per site (**R1**)	**R2**	substitutions per site (**R1**)	**R2**	substitutions per site (**R1**)	**R2**
C. famiriaris (dog)	*H. sapiens* (human)	83	0.070	0.84	0.125	1.51	0.070	0.84	0.165	1.99	0.075	0.90
M. musculus (mouse)	*H. sapiens* (human)	91	0.080	0.88	0.135	1.48	0.080	0.88	0.190	2.09	0.060	0.66
R. norvegicus (rat)	*H. sapiens* (human)	91	0.085	0.93	0.135	1.48	0.135	1.48	0.185	2.03	0.050	0.55
G. gallus (chicken)	*H. sapiens* (human)	310	0.140	0.45	0.320	1.03	0.185	0.60	0.360	1.16		
G. gallus (chicken)	*M. musculus* (mouse)	310	0.135	0.44	0.330	1.06	0.200	0.65	0.375	1.21		
G. gallus (chicken)	*R. norvegicus* (rat)	310	0.145	0.47	0.325	1.05	0.240	0.77	0.365	1.18		
X. tropicalis (frog)	*H. sapiens* (human)	360	0.170	0.47	0.315	0.88	0.230	0.64	0.330	0.92	0.145	0.40
X. tropicalis (frog)	*M. musculus* (mouse)	360	0.165	0.46	0.310	0.86	0.235	0.65	0.340	0.94	0.130	0.36
X. tropicalis (frog)	*R. norvegicus* (rat)	360	0.160	0.44	0.310	0.86	0.270	0.75	0.340	0.94	0.130	0.36
D. rerio (zebrafish)	*H. sapiens* (human)	450	0.205	0.46	0.340	0.76	0.270	0.60	0.340	0.76	0.215	0.48
T. rubripes (fugu)	*H. sapiens* (human)	450	0.205	0.46			0.290	0.64			0.205	0.46
T. nigroviridis (tetraodon)	*H. sapiens* (human)	450	0.210	0.47	0.345	0.77	0.290	0.64			0.190	0.42
D. rerio (zebrafish)	*X. tropicalis* (frog)	450	0.210	0.47	0.275	0.61	0.280	0.62	0.315	0.70	0.185	0.41
T. rubripes (fugu)	*X. tropicalis* (frog)	450	0.185	0.41			0.295	0.66			0.195	0.43
T. nigroviridis (tetraodon)	*X. tropicalis* (frog)	450	0.185	0.41	0.300	0.67	0.295	0.66			0.175	0.39
D. rerio (zebrafish)	*G. gallus* (chicken)	450	0.195	0.43	0.290	0.64	0.255	0.57	0.345	0.77		
T. rubripes (fugu)	*G. gallus* (chicken)	450	0.195	0.43			0.280	0.62				
T. nigroviridis (tetraodon)	*G. gallus* (chicken)	450	0.200	0.44	0.300	0.67	0.280	0.62				
