# Supplementary material for: Molecular evolution of the reactive oxygen-generating NADPH oxidase (Nox/Duox) family of enzymes
Source: BMC Evol Biol. 2007 Jul 6;7:109. doi: 10.1186/1471-2148-7-109 (PMC1940245; doi:10.1186/1471-2148-7-109)
Supplement: Additional File 7 — Alignment of vertebrate p47 phox, NOXO1, p67phox, NOXA1, and p22phox proteins to estimate substitution rates. Amino acid sequences of p47phox, NOXO1, p67phox, NOXA1 and p22phox were aligned. These alignments were used to estimate evolutionary substitution rates in vertebrate orthologs of Nox regulatory subunits. [file 1471-2148-7-109-S7.pdf]

Additional file 7

Alignment of vertebrate p47*phox*, NOXO1, p67*phox*, NOXA1, and p22*phox* proteins to estimate substitution rates

To describe species of the genes, we used the following naming: human (*H. sapiens*), dog (*C. familliaris*), rat (*R. norvegicus*), mouse (*M. musculus*), chicken (*G. gallus*), frog (*X. tropicalis*), zebrafish (*D. rerio*), fugu (*T. rubripes*), and tetraodon (*T. nigroviridis*). Amino acid sequences of p67*phox*, NOXA1 and p22*phox* listed in Additional file 6 were aligned. Several sequences of NOXO1 proteins contained partial sequences: the minimum length was 281 amino acids for frog NOXO1 (see Additional file 6). To accurately compare substitution rates, we prepared the trimmed NOXO1 and p47*phox* sequences corresponding to the region of the minimum length of the NOXO1 (e.g., residues 30-273 in human NOXO1 or residues 32-267 in human p47*phox*) for alignment. Alignments of p47*phox*, NOXO1, p67*phox*, NOXA1 and p22*phox* were used to estimate R1 and R2 in Additional file 3.

p47*phox* (vertebrates)

```
human-p47phox          WQDLSEKVVYRRFTEIYEFHKTLKEMFPIEAGAINPENRIIPHLPAPKWFDGQRAAENHQ 122
dog-p47phox            WHDLSEKVVYRRFTEIYEFHKMLKEMFPIEAGDINPENRIIPHLPAPRWFDGQRAAESRQ 122
mouse-p47phox          WQDLSEKVVYRKFTEIYEFHKMLKEMFPIEAGEIHTENRVIPHLPAPRWFDGQRAAESRQ 122
rat-p47phox            WQDLSEKVVYRKFTEIYEFHKMLKEMFPIEAGEIHTENRVIPHLPAPRWYDGQRAAESRQ 122
chicken-p47phox        WNDLSEKLIYRRFTDIYEFHKALKEMFPIESGDINAENRIIPHLPAPKWFDGQRSTQSRQ 122
frog-p47phox           WQDLTEKLVYRKFTEIYEFHKSLKEMFPIEAGDISKEHRTIPHLPAPKWFDGLRSTENRQ 122
tetraodon-p47phox      WSDLTEKLIYRTYPEIYTFHKALKEMFPIEAGKIEKRDRIIPSLSAPPWLDSQKSTETRQ 122
```

```
fugu-p47phox          WSDLTEKLIYRTYPEIYTFHKSLKEMFPIEAGKIEKRDRIIPSLSAPPWLDSQKSTETRQ 122
zebrafish-p47phox     WSDQSEKLVYRRYPEVHTLHKTLKEMFPIEAGDIDEKDRIIPTLPAPKWLDNQKTTETRQ 122
                      * *  **  **        ** ******** * *    * ** * ** * *        *

human-p47phox         GTLTEYCGTLMSLPTKISRCPHLLDFFKVRPDDLKLPTD-NQTKKPETYLMPKD-GKST- 179
dog-p47phox           GTLTEYYNTLMGLPVKISRCPQLLDFFRVRPDDLKLPTD-SQVKKPETYLVPKD-GKSS- 179
mouse-p47phox         GTLTEYFNGLMGLPVKISRCPHLLDFFKVRPDDLKLPTD-SQAKKPETYLVPKD-GKNN- 179
rat-p47phox           GTLTEYFNSLMGLPMKISRCPHLLNFFKVRPDDLKLPND-SQVKKPETYLTAKD-GKNN- 179
chicken-p47phox       GTLAEYCYTLVNLPHKISRCRHVVSFFEVRPDDMNPVTD-SQIRKPEVFLLPKD-AKKN- 179
frog-p47phox          VTLSDYFSSLLSLPPKISRCPHVLNFFQVRSDDVNPVANNTNGRKPETFLLKVDTAKKN- 181
tetraodon-p47phox     TSLSDYCQALVNLPPHISRCTHLTSLFKVRPEDENPAAP-NTLKRNETFVVSRDLARGN- 180
fugu-p47phox          TTLSDYCHSLVNLPPHISRCTHLTGFFTVRPEDENPPSP-NILKRNETFVVSKDLARGN- 180
zebrafish-p47phox     ATLAEYCRSLLNLPANISRCQLIRDFFKMRPEDETPPAP-HPYKRNETFIMSTNRVRSNT 181
                        *  *   *  **  ****      *  *  *             *

human-p47phox         ATDITGPIILQTYRAIANYEKTSGSEMALSTGDVVEVVEKSESGWWFCQMKAKRGWIPAS 239
dog-p47phox           VTDITGPIILQTYRAIADFEKTSSSQMALATGDVVDVVEKSESGWWFCQTKTKRGWVPAS 239
mouse-p47phox         VADITGPIILQTYRAIADYEKSSGTEMTVATGDVVDVVEKSESGWWFCQMKTKRGWVPAS 239
rat-p47phox           VADITGPIILQTYRAIADYEKGSKTEMTVATGDVVDVVEKSESGWWFCQMKTKRGWVPAS 239
chicken-p47phox       TSDITGPIVLQTYRAIADYEKSSKSEMAVKAGDAVDVVEKSETGWWFCQLKTKRGWVPAA 239
frog-p47phox          VSDITGPIILQSYRVIADYEKNSKSELAAKNGDVVEIVEKSENGWWFCQLRNKRGWMPAA 241
tetraodon-p47phox     ASEISGPIILDMYRAIADYTKTTKYEINLLAGDQVEIVEKNQNGWWFCQMDSKRGWVPAS 240
fugu-p47phox          VSEISGPIILDMYRAIADYTKTTKYEINLHAGDQVEIVEKNQNGWWFCQCDSKRGWVPAS 240
zebrafish-p47phox     TSEITGPIILETYRVIADYSKSSKYELTLKMGDMVDIVEKSPNGWWFCQCESRRGWVPAS 241
                         * *** *  ** **   *          ** *  ***    ******    *** **

human-p47phox         FLEPLDSPDETEDPEPNYAG---EPYVAIKAYTAVEGDEVSLLEGEAVEVIHKLLDGWWV 296
dog-p47phox           YLEPLDSPDEAEDPEPNYEG---EPYVTIKAYTAEMEDEMSLQEGEAIEVIHKLLDGWWV 296
mouse-p47phox         YLEPLDSPDEAEDPDPNYAG---EPYVTIKAYAAVEEDEMSLSEGEAIEVIHKLLDGWWV 296
rat-p47phox           YLEPLDSPDEAEDPDPNYAG---EPYVTIKAYAAVEEDEVSLSEGEAIEVIHKLLDGWWV 296
chicken-p47phox       YLEPMDGPDESEEQEPNYAG---ELYVVQKSYTAVEEDELTLKEGDTIEVIHKLLDGWWV 296
frog-p47phox          YLEPLDGPDESEEQDPNYEG---DLHITTKDYSGELDDELSLQEGENVEVIHKLLDGWWV 298
tetraodon-p47phox     YLEPLDGPEESEEADPDYEGS--ELFITIKAYKAEQEDEISLDLGESIEVIHKLLDGWWV 298
fugu-p47phox          YLEPLDGPEESEEAEPDYGGSPCELYITIKAYKAEQEDEITLDLGESIEVIHKLLDGWWV 300
zebrafish-p47phox     YLEPLDGADESEEPEPNYAG---ELYKTTRGYKAVEQDEMTLEAGVIIEVIHKLLDGWWV 298
                       *** *   * *   * * *           *     **  *  *    ************

human-p47phox         IR 298
dog-p47phox           VR 298
mouse-p47phox         VR 298
rat-p47phox           VR 298
chicken-p47phox       IR 298
frog-p47phox          VR 300
tetraodon-p47phox     VR 300
fugu-p47phox          VR 302
zebrafish-p47phox     VR 300
                       *
```

NOXO1 (vertebrates)

```
zebrafish-NOXO1     WSDGNEITVYRSLEDFKKMHRQLKKKFPP-SNPFKRSARIVPEFK--------GNKWSG- 112
tetraodon-NOXO1     WSDHNEIVVYRTFQDFRKMHK------------LKRS----------------GKKKSP- 135
chicken-NOXO1       WSDQNNILIYRTLEEFKRFHKELKRKFPIESGSLRRSDRTIPRFKD----INGKQKKSGK 93
frog-NOXO1          WSDHNEILIYRTFEDFKKLNRQLKKKFPLEAGLFRKSDNLLPKLKD----VPIFRKNRTT 56
```

```
human-NOXO1        WSDGSDTFVRRSWDEFRQLKKTLKETFPVEAGLLRRSDRVLPKLL-----DAPLLGRVGR 112
dog-NOXO1          WSDGSDTFVRRSWAEFKELHKTLKEAFPVEAGLLRRSDRILPKLP-----DTSLLVRGGR 112
mouse-NOXO1        WSDNSDTFVRRSWDEFRQLQKTLKKTFPVEAGLLRRSEQVLPKLP-----DAPLLTRRGH 112
rat-NOXO1          WSDNSDTFARRSWEEFRQLQKTLKKIFPVEAGLLQRSERVLPKLPGQACRNAPLLTRRGH 118
                   ***       *    *                    *

zebrafish-NOXO1    -SKSVLRMKALEEYCGQLLKSDAQVCRSSELIQFLLPKAHDLNADFAKNCIVIMPSDVTL 171
tetraodon-NOXO1    -TRSLVRLKFLQKYCNELLSCEPRVSQSADLIQFFHPNAQDLEPEFSKNRQEEVKAEAGH 194
chicken-NOXO1      INRSLERLKLLETYTQELLKVDAKISQGEDVIQFFKAQTQDLDPCFPEDSVVIMPSEIGG 153
frog-NOXO1         -NRFIERLRLLEKYSQELLRTDGKISQCDLVLKFFTPSNNDLNPKFPENSLVMMTSDSKD 115
human-NOXO1        TSRGLARLQLLETYSRRLLATAERVARSPTITGFFAPQPLDLEPALPPGSRVILPTPEEQ 173
dog-NOXO1          TGRGLARLRLLDTYTRALLAAAEQLSRSAVLTGFFEPQPVDLEPVLPPGSLVILPTPEE- 173
mouse-NOXO1        TGRGLVRLRLLDTYVQALLATSEHILRSSALHGFFVPKPLDLEPMLPPGSLVILPTPEE- 173
rat-NOXO1          TGRGLLRLRLLETYVRSLLATSQHIVTSSTLNSFFAPKPLDLEPMLPPGSLVILPTPEE- 178
                         *   *  *   **              *      **

zebrafish-NOXO1    GSSK--AESNSGVTQPFVTETYRCIANYETKDTKNRPFKVEVDETVDVLIKDQKGWWLVE 229
tetraodon-NOXO1    G-------SVGNVTQPFVTVTYRCVSQYETKDTKNKPFKVAADEKVDVLIKDKAGWWLVE 247
chicken-NOXO1      EKKKEVQQQQLSITYPQVSQSYRCIETFETKDTKNKTFKVAKKEIVEVLLKDMTGWWLVE 213
frog-NOXO1         QKEQKKPLPEAPAIHPIVSQQYICMEDYETKDTKNRPFKVKRHELVGVLIKENTGWWLVE 175
human-NOXO1        PLSRA---AGRLSIHSLEAQSLRCLQPFCTQDTRDRPFQAQAQESLDVLLRHPSGWWLVE 230
dog-NOXO1          PHRRP---PHSPAICSLEAQSLRCLQPFSTQDTQGWPFHARAQEVIDVLLRHPSGWWLVA 229
mouse-NOXO1        PLSQP---RGSLDIHSLEAQSIPCVQPFHTLDIRDRPFHTKAQEILDILLRHPSGWWLVE 229
rat-NOXO1          PLSQP---IGSLAIHSLEAQSMRCLQPFHTLDTKDRPFHTQAKEILDILLRHPSGWWLVE 234
                                          *     * *     *     *    *     *****

zebrafish-NOXO1    NESKHLAWFPAPYLERAEMADDGPDEMDNESFQSAGVFYVATKAYKATNSDELSVELGSV 289
tetraodon-NOXO1    NEEKRMAWFPAPYLEKLEEDGD-EDDTDG-----TRTLYLTAKNYKASKGDEISVAVGAV 301
chicken-NOXO1      NADKQIAWFPASYLEQISAHKDIQNVESSDE---EGSLYFVMRAYEAQKADELSLNKGVV 270
frog-NOXO1         NEEKHLAWFPAPYLKDVDNSEDTDSGTSED----EGVLYYAAKAYEAMNSDEVSITVGVL 231
human-NOXO1        NEDRQTAWFPAPYLEEAAPGQGREGGPSLGS---SGPQFCASRAYESSRADELSVPAGAR 287
dog-NOXO1          NEEQQMAWFPAPYLEEAAPD--REG-TTLRS---SGSQFCASQAYESSHADELSVPAGAR 283
mouse-NOXO1        NKDQQVAWFPAPYLEEVATCQGQESGLALQG---SGRQFCTTQAYEGSRSDELSVPSGAR 286
rat-NOXO1          NKDQQTAWFPAPYLEEIATGQGQESGMAVQG---SGRQFCATQAYEGSRPDELSVPSGAR 291
                   *     ***** **                              *     ** *   *

zebrafish-NOXO1    LEVLQKSDNGWWIVR 304
tetraodon-NOXO1    VEVLQKSDSGWWLIR 317
chicken-NOXO1      VEVVRRSDNGWWLIR 285
frog-NOXO1         VEVIEKSNNGWWLIR 246
human-NOXO1        VRVLETSDRGWWLCR 303
dog-NOXO1          VSVLETSDRGWWLCR 298
mouse-NOXO1        VHVLETSDRGWWLCR 301
rat-NOXO1          VHVLETSDRGWWLCR 306
                     *   *  ***  *
```

p67*phox* (vertebrates)

```
human-p67phox      MSLVEAISLWNEGVLAADKKDWKGALDAFSAVQDPHSRICFNIGCMYTILKNMTEAEKAF 60
dog-p67phox        MSLAEAISLWNEGVLAADKKDWKGALEAFAAVQDPHSRICFNVGCMHTILGNMPEAEKAF 60
rat-p67phox        MSLAEAIRLWNEGVQAADKKDWKGALEAFSEVQDPHSRICFNIGCMYTILDNLQEAEQAF 60
chicken-p67phox    MSLVETIRLWQEGVCAADGKEWGAALKAFTAVQNPPAKICFNIGCTHLVLGQLAEAEEAF 60
frog-p67phox       MALVEIMRLWSEGVAAAENEDWNGALKSFTSITDPRSKICFNIGCCHLVLGDLEKAEKAF 60
```

```
tetraodon-p67phox     MSFLDTLRQWDNACTVADGQDFSAALQVFLSIQEPNSKICFNIGCLHLLNEDLSAAEKAF 60
fugu-p67phox          MSFLDTLKQWDKASTVADRQEFSEALEIFLSIEEPNSKIYFNIGCLHLLNEDLKDAEKAF 60
zebrafish-p67phox     MSFVSTLRQWDEAVACVEQRDPDAALRIFLSIEEKNSKIAFNIGCLCLNNSDLDEAEKAF 60
                      *:: . :  *.:.   .: .:   **  *  : :  ::* **:**      ::  **:**

human-p67phox         TRSINRDKHLAVAYFQRGMLYYQTEKYDLAIKDLKEALIQLRGNQLIDYKILGLQFKLFA 120
dog-p67phox           TRSINKDKHLAVAYFQRGMLYYHMEKYDAAIKDLKEALTQLRGNQLIDYKILGLQFKLFA 120
rat-p67phox           TKSINRDKHLAVAYFQRGMLYYSMEKYLLCVYCLREALVTFRKKQVLSPKPEGLQFLLQR 120
chicken-p67phox       TQSISCDKHLAVAYFQRGTVFYKRHNHEMALKDFKEALAQLRGNQLIDYKILGLRYRLFA 120
frog-p67phox          TLTIERDMHLAVGYFQRGFVFFQRGKYSLALQDWTRAYTEMRGNQLIDYKILGLIFKLYS 120
tetraodon-p67phox     DSSISKDEHLAVAFFQRAITFYKMSRQTHRPCHFQQTFKELRGNQLIDYGALGLRYKLTA 120
fugu-p67phox          DSSICKDEHLAVAFFQRAITFYKMTRQEYSLADFQQTLKELRGNQLIDYGALGLRYKLNA 120
zebrafish-p67phox     DGSIGKDEHLAVAFFQRGVTFYKKEKFEESLLDFQQAFKQLRGNQLIDYTPLGLRYKLYA 120
                        :*  * ****.:***.  ::   .         .:   :* :*::.    ** : *

human-p67phox         CEVLYNIAFMYAKKEEWKKAEEQLALATSMKSEPRHS-KIDKAMECVWKQKLYE-PVVIP 178
dog-p67phox           CEVLYNIAFMYAKNEEWKKAEEHLALAMSMKSEPRHT-KIDKAMECVWKQKLYE-PVVIP 178
rat-p67phox           SPVLYNIALMHAKKEEWKKAEEQLALATNMKSEPRHS-KIDKAMESIWVSVVVDGPCCLA 179
chicken-p67phox       CEILYNIALVYATMENWKKAEEHLTLAMSSKSEPQHN-KIDRAMEAILKQKLYE-PVAIP 178
frog-p67phox          CEILHNIALTHAKEGKWAKAEESILLALSQKVELRHNTKLEKAMEDILKEKVFA-AVKIP 179
tetraodon-p67phox     CEVLHNMALAEAQLGRWEKAQESLVKALDYSSDSKLG-AIDKALQATLKQKLFK-LTGFP 178
fugu-p67phox          CEVLHNIALTEAQMGHWEKAQESLVKALDYRTESKLG-IIDNALQATLKQKLFK-LIGFP 178
zebrafish-p67phox     CEVLHNIGLAQAQLGKWEKAQENLLTALSLRADAKFS-HIDHALDAILKHKLFP-LVEVR 178
                      . :*:*:.:  *   .* **:* :  * .   : :    ::.*::      :      .

human-p67phox         VGKLFRPNERQVAQLAKKDYLGKATVVASVVDQDSFSGFAPLQPQAA-------EPPPRP 231
dog-p67phox           MGRLFRPNERQVAQLAKKDYLGKATVVASVVDQDSFSGFAPLQPQAA-------EPPPRP 231
rat-p67phox           QYHVQRASHKQVPVLS-KDLLGEETVVASVVHQDNFSGFAPLQPQSA-------EPPPRP 231
chicken-p67phox       TGKLFRPNEKQVAQLEKKDYLGKAMVVASVVDKDSFSGFAPLQPQAS-------GPPPRP 231
frog-p67phox          KGRIFQPNERLVEQLEKKDYLGKALVVASVVDKDSFSGFAPLQPQAS-------NPPPRP 232
tetraodon-p67phox     SKVLFKPNKRYVAELEKKDYLCKAKVVASVVPQDEFSGFAPLMPQVRPHLRAASSPTAES 238
fugu-p67phox          SKVLFKPNKHYVAELEKKDYLGKAKVVASVVPQDEFSGFAPLMPQVE------SGQTFSK 232
zebrafish-p67phox     AGLLFKPNKKYVAELEKRDYLGKAKVVASVVPADEFSGFAPLQPQID-------NVPSIP 231
                         : :...: *  *  :* * :  ******  *.******* **           .

human-p67phox         KTPEIFRALEGEAHRVLFGFVPETKEELQVMPGNIVFVLKKGNDNWATVMFNGQKGLVPC 291
dog-p67phox           KTPEIFRALEGEAHRVLFGFVPETPEELQVMPGNIVFVLKKGNDNWATVMFNGQKGLVPC 291
rat-p67phox           KTPEIFRALEGEAHRVLFGFVPETPEELQVMPGNIVFVLKKGSDNWATVMFNGQKGLVPC 291
chicken-p67phox       KTPEILRALEGQPHRVLYEFIPETAEELQVLPGNIVFVLKKEKDNWATVMFNGKKGIVPC 291
frog-p67phox          KTPEILRTLQGEPHRVLFEFNPETAEEMQVLPGNIVFVLKKGDDNWATVVFNGKKGIVPC 292
tetraodon-p67phox     NSARVHRALEGEPHTVLYEFVPETSDELAVVPGNVVFVLQKGADNWASVVFNERRGLVPY 298
fugu-p67phox          PEPELLRALEGEPHTVLYKFVPETSDELAVVPGNVVFVLQKGADNWASVVFNERRGLVPY 292
zebrafish-p67phox     KVPEVLRVLEGEPHTVLYEFVPETKEELAVLPGNIVFVLHRGTDNWASVVFNEKRGLVPY 291
                        ..: *.*:*:.* **: * *** :*: *:***:****::  ****:*:** ::*:**

human-p67phox         NYLEPVELRIHPQQQPQEESSPQSD-IPAPPSSKAPGRPQLSPGQK----QKEEPKEVKL 346
dog-p67phox           NYLEPVELRIHSQQQPQEEASPESD-IPAPPSSSAPGRPQLPPGWSS--FCFLSPQDVKL 348
rat-p67phox           NYLEPVELRIHPQSQPQEDTSLESD-IPPPPNSSPPERLQLSPGHK----QKEEPKEVKR 346
chicken-p67phox       NFLEPMEL--QHKLHVQDEAPLEPD-IPEPPSSTAPRPRRPAPGQEKPDTPIHHLQESEP 348
frog-p67phox          NYLEPVELRFQSAQQTGVQSELDSP-TNRPQQSDVPAPPDATPPQL--------LKNTKE 343
tetraodon-p67phox     NYLERLEIS-LAAKLKDVRTCQPGK--PRPPSQQPPTRPER-NLLTLFSG----LEFQEA 350
fugu-p67phox          NYLERLEIT-MASKQNNVQ--------SRPPSRQPPTRPERKSGLPPCADDRRNTQMKES 343
zebrafish-p67phox     NFLEPLDIVTMTSKPVETEALNENDDIPAPPRRAAPSRPVAPEGLKTVNAKLYSPQTVNT 351
                      *:** :::                     *     *                   :  :
```

```
human-p67phox          SVPMP-YTLKVHYKYTVVMKTQPGLPYSQVRDMVSKKLELRLEHTKLSYRPRDSNELVPL 405
dog-p67phox            RVPMP-YTVKVHYKYTVVMEIQAGLPYSQLRDMVAKKLELLPEHTKLSYRPPDSHELEPL 407
rat-p67phox            SVPMP-YMLKVHYKYTVVMETQLGLPYSQLRNMVSKKLELLPEHTKLSYQRRDSPELLLL 405
chicken-p67phox        DIPKP-YVLKVHYKYTVAMQVKPDLSYKELLGLVCDKLELQPEHTMLRYKSAASGELVPL 407
frog-p67phox           AVAVASYLVKVYYKYTVAIQISSKLPFADLLTLISSKLQLLPSRMKLSFK--EDQDDVLL 401
tetraodon-p67phox      QLVDDSYVVKVRFTFTFAVIVPRGSSYATLAQKVGEKLSVPADAVILSLSSEAAEEDVIN 410
fugu-p67phox           ELADDSCVVKVRYTFTFAVLVPRGSSYATLAEKISEKLSVPANAIVLSLSSEATEQNVID 403
zebrafish-p67phox      REFSG-CVVKVHFQFTIAIAIAHGQPYGVILQMISSKLKLPASTLTLRYAKEGSAERVII 410
                               :** : :*..:      .:  :   : .**.:  .   *        :

human-p67phox          SEDSMKDAWGQVKNYCLTLWCENTVGDQGFPDEPKESEKADANNQTTEPQLKKGSQVEAL 465
dog-p67phox            SEDNMKAAWGQVRNYCLTLWCENTVGDQGFPDEPQESEKSEANNQTTEPTLKEGGHVVAL 467
rat-p67phox            SEESMKDAWAQVKNYCLTLWCEHTVGDQGFVDEPKEKENSDADNRTTEPQPKEGTQVVAI 465
chicken-p67phox        SAQNLEEAWSHSKDQCLTVWCDCTEGE-GFLPDSKPEEPQQAAAETGP------TQVVAQ 460
frog-p67phox           NEENTEKAWSLATDNCLKLKCT---------EVQVRQARSLYCSTMQGYFKAQGKYPIAL 452
tetraodon-p67phox      GSTDMEAVWGRASGRCITLWCR-------------------LAEQTSETEPRRETFLLAL 451
fugu-p67phox           GGTDMEGVWSRVSGRCITLWCR-------------------LAQTNERVQ--KESSLLAL 442
zebrafish-p67phox      EDSEMEAVWNSAKDGRLTLWCS-------------------VTEGKSASH----AKVVAL 447
                          . : .*    .  :.: *                                     *

human-p67phox          FSYEATQPEDLEFQEGDIILVLSKVNEEWLEGECKGKVGIFPKVFVEDCATTDLESTRRE 525
dog-p67phox            FTYEATQPEDLEFQQGDIIQIISMVNEDWLEGECKGKIGIFPKAFVEEHATTDLESSPRG 527
rat-p67phox            FSYDATQPEDLEFVEGDVILVLSHVNEEWLEGECKGKIGIFPKAFVEGCAAKNLEGTPRE 525
chicken-p67phox        YSYEATQPEDLEFQAGDVILVLSKVNEDWLEGQCNGKIGIFPSAFVRDGNTKDP------ 514
frog-p67phox           FEYEATQPEDLPFCKGDIIKILSHVSEDWWEGECQGRMGIFPKVFTEE------------ 500
tetraodon-p67phox      HTYDSPNPEDLTFQQGDKILLLSKVNQDWLEGQCHGNTGIFPAAFVEEVSVSE------- 504
fugu-p67phox           HSYDSSNPEDLSFHQGDRITLLSKVNQDWLEGEFNGNTGIFPAAFVEEVPANG------- 495
zebrafish-p67phox      YSYESSTPEDLEFKQGNVITVLSKVNDEWLEGQCNGKIGIFPSSFVEPLNGDPH------ 501
                       . *::. **** *  *: * ::* *.::* **: :*. ****  *..

human-p67phox          V 526
dog-p67phox            V 528
rat-p67phox            V 526
chicken-p67phox        -
frog-p67phox           -
tetraodon-p67phox      -
fugu-p67phox           -
zebrafish-p67phox      -
```

NOXA1 (vertebrates)

```
human-NOXA1          MASLGDLVRAWHLGAQAVDRGDWARALHLFSGVPAPPARLCFNAGCVHLLAGDPEAALRA 60
dog-NOXA1            MPSLGDLVHDWHRGVQAVARGDWGCALRLFSGDPDPPAKMCFNLGCVHLLAGDPEAALRA 60
mouse-NOXA1          MSSLGDQIRDWHRGVLAVAREDWDSALCFFSDVREPLARMYFNRGCVHLMAGDPEAALRA 60
rat-NOXA1            MSSLGDQIRDWHRGVLAVAREDWDSALCFFSDVREPLAKMYFNMGCVHLMAGDPEAALRA 60
frog-NOXA1           -MHYKEVVRRWHEGVVAAEGKDYDAALRSFTAIEDPPSRIWFNVGGIYLLRGDLPRALEA 59
zebrafish-NOXA1      -MLYIELIRLWDEAVKAIDIRDWQGALSKLNQITDHNCRTMFVVASTHIALGQVDLAIKA 59
                               *     *    *   **              *         *    *  *

human-NOXA1          FDQAVTKDTCMAVGFFQRGVANFQLARFQEALSDFWLALEQLRGHAAIDYTQLGLRFKLQ 120
dog-NOXA1            FDQAVTKDTCMAVGFFQRGVANFQLERFQEALSDFRLALAQLRGNAAIDYTQLGLRFKLK 120
```

```
mouse-NOXA1          FDQAVTKDTCMAVGFLQRGVANFQLQRFQEAVSDFQLALAQLRDNAVIDYTQLGLNFKLQ 120
rat-NOXA1            FDQAVTKDTCMAVGFLQRGVANFQLQRLQEAVSDFQLALAQLRGNAAIDYTQLGLDFKLQ 120
frog-NOXA1           YDKSLAQDPCLAVGYYQRGYLQFKLGRYEKALSDCHLALSNLRNNSFIDYKQLGLRHVLF 119
zebrafish-NOXA1      LDRVIAKDSCLAVGFFQRSAVHMMANRLEEALSDCIWAQKYMRENPVIDYKQLGLRYKLY 119
                      *     * * ***  **        *   * **   *    *    *** ****   *

human-NOXA1          AWEVLHNVASAQCQLGLWTEAASSLREAMSKWPEGSLNGLDSALDQVQRRGSLPPRQVPR 180
dog-NOXA1            TWEVLFNVGAAQCALGLWAEAAGSLEEALCKGPEGAGEDLHAALAQVQKQATLQLRQVPR 180
mouse-NOXA1          AWEVLYNMASAQCQAGLWTKAANTLVEAISKWPEGAQDILDIAMDKVQKQVPLQLQQVPK 180
rat-NOXA1            AWEVLYNMASVQCQAGLWTKAANTLVEAISKRPEGAQDTLEAAMDKVQKQVPLQLRQVPK 180
frog-NOXA1           SWEAQYNMAAVLCSLGRWESAEEKLKETLQ--GDGRNAKLDWALDQVQRRSLLQPMSVPE 177
zebrafish-NOXA1      SWQVLYNAAAVHSRLQQWDKARDILLAASQERGAGRSNLIDTALEAISRKDVLEPLLLPE 179
                      *    *          *  *   *         *       *         *     *

human-NOXA1          GEVFRPHRWHLKHLEPVDFLGKAKVVASAIPDDQGWGVRPQQPQGPGANHDARSLIMDSP 240
dog-NOXA1            GEVFRPHRRHVEHLEPVDFLGKAKVVSSAIPDDHLQGSRPQQRQVSGA-----------P 229
mouse-NOXA1          GEVFQPPRRYLKHLEPMDFLGKAKVVASVIPDDHN--AQPQQR----------------- 221
rat-NOXA1            GEVFQPPRRYLKHLEPMDFLGKAKVVASVIPDDHNSDIQPQQS----------------- 223
frog-NOXA1           GEFFRPRKQEVEQLNSVDFLGKPTVISSVVPNDQYSGFEPLRPQQPGFYEPCRDAMQCRE 237
zebrafish-NOXA1      GEVFRPRKLEVDQLKPRDFLGEAKVITSMIPNDDFRGFDPLRPQKPGYYEPKVEDGQ--D 237
                     ** * *       *   ****   *  *  * *      *

human-NOXA1          RAGTHQGPLDAETEVGADRCTSTAYQEQRPQVE--------------------------- 273
dog-NOXA1            SSQPPNGPGDPS--CGASLACSPHPSAPRP------------------------------ 257
mouse-NOXA1          SQAEHAGHQPSSSMCKRVLSTTGGHTSP-------------------------------- 249
rat-NOXA1            SQVEQAGLQSSSPVCKRVLSTRGGHMSP-------------------------------- 251
frog-NOXA1           AGYHRVVVHYYPENSNEVAVKANSVLFVLNKDGDWATAIHDGQKILIPTSFLEPTNPPKA 297
zebrafish-NOXA1      SRYMIMKSAYVAKGAGELTVPAGAEVFVYSDDD------RDGLAVIIY------------ 279

human-NOXA1          ---QVGKQAPLSPGLPAMGGP--------------------------------------- 291
dog-NOXA1            --------SQLGPQRAALGWT--------------------------------------- 270
mouse-NOXA1          ---------GLYDSLLASRRP--------------------------------------- 261
rat-NOXA1            ---------GLWDSLLATGGP--------------------------------------- 263
frog-NOXA1           DIKKMNNGIPLPPMKTPPTRPNVRPGMEPLTGVQAGAPVPPQPAGGAAEPYKIKALPVGM 357
zebrafish-NOXA1      DGKELANGIPHPPALKPPNRP--------------------------------------- 300

human-NOXA1          ---------------------------------GPGPCEDPAGAGGAGAGGS-------- 310
dog-NOXA1            ---------------------------------EPGGCLS-ASSQVVATGGP-------- 288
mouse-NOXA1          ---------------------------------GPGPSEVSSGSEGAATKDP-------- 280
rat-NOXA1            ---------------------------------VPGPSEDSSSAEGTATKDP-------- 282
frog-NOXA1           EPIVEVAVPVQRSVPTHKETGNVPLGNDASLVEDAGRTIIKPKGESAPEPGPFPTRQTGP 417
zebrafish-NOXA1      ------QLPSQRSLELHPSS--------------AGASHTTPPTTSSSTRIPPQQIQTSP 340
                                                        *

human-NOXA1          ------------EPLVTVTVQCAFTVALRARRGADLSSLRALLGQALPHQ--AQLGQLSY 356
dog-NOXA1            ------------ESLVTVTVQCAFTLALKVPWGAGLPHLRTLLSQALPLQ--AQHGQLSY 334
mouse-NOXA1          ------------ESLVTVTVQCHFTVPLKVPRGTGLSSFQTLLAQALLHQ--TQTGQLSY 326
rat-NOXA1            ------------ESLVTVTVQCHFTVPLKVPRGTDLSSFRTLLSQALLQQ--TQKGQFSY 328
frog-NOXA1           DNGPMATPVPTDDDKLVLSVHAEFTVNMTVSKAITYPELQGALREELRKHGEQMANHLSY 477
zebrafish-NOXA1      Q----------ESGSVVVKVHYTYTMALRVPAETPFRDLQEKIAQKLGQP--AMNIRLRH 388
                                        *    *                     *
```

```
human-NOXA1         LAPGEDGHWVPIPEEESLQRAWQDAAACPRGLQLQCRG-AGGRPVLYQVVAQHSYSAQGP 415
dog-NOXA1           RDPSHEARWVALPGEEALQGAWRDTAASPRGLQLQCRA-AGSRPVLYQAVAQHNYCAQGP 393
mouse-NOXA1         KAPGEERSWIPISTEESLQSIWRNVPVGPGGLQLQCQG-VWGRPVLYQVVAQYNYRAQRP 385
rat-NOXA1           KARGEDRAWVPISTEDSLQSVWRNVPVSPRGLQLQCRG-AWGRPVLYQVVAQYDYRAQRP 387
frog-NOXA1          RDPE-SRGLTPVTGSKDWQEVLKLSRANQVTLCCKETTLCAGRPVLYRMRAQYDYLAQGP 536
zebrafish-NOXA1     RRPG-TRVLTPLNGDDGLDCLEGVAESGRAQIWCQNEDPLANRTILYQMVALYDYNAQGP 447
                                                              *  **   *   * ** *

human-NOXA1         EDLGFRQGDTVDVLCEE--PDVPL-AVDQAWLEGHCDGRIGIFPKCFVVPAGPRMSGAPG 472
dog-NOXA1           EDLDLRQGDMVDVLCAGQGPDVPLHVVDPAWLEGHCDGRIGIFPKCFVVPAG-------- 445
mouse-NOXA1         EDLDFHQGDTVDVLCEV----------DEAWLEGHRDGCVGIFPKCFVVPAGAYVEAML- 434
rat-NOXA1           EDLDFRQGDTVDVLCEV----------DEAWLEGHRDGRVGIFPKCFVVPAATCVEALP- 436
frog-NOXA1          ADLSFQQGDLISILSEVN----------GEWLEGHCHRGIGIFPKCFAQR-AEGI----- 580
zebrafish-NOXA1     EDLEFSEGDTIDILSEVN----------EEWLEGHVAGNIGIFPQSFAHRDTDSISGAST 497
                     **    **    *                *****     ****  *

human-NOXA1         RLPRSQQGDQP 483
dog-NOXA1           -----------
mouse-NOXA1         -VLGPQPGDQN 444
rat-NOXA1           -VPEPQPGEQH 446
frog-NOXA1          -----------
zebrafish-NOXA1     D---------- 498
```

p22*phox* (vertebrates)

```
mouse-p22phox         MGQIEWAMWANEQALASGLILITGGIVATAGRFTQWYFGAYSIAAGVLICLLEYPRGKRK 60
rat-p22phox           MGQIEWAMWANEQALASGLILITGGIVATAGRFTQWYFGAYSIVAGVLICLLEYPRGKRK 60
Human-p22phox         MGQIEWAMWANEQALASGLILITGGIVATAGRFTQWYFGAYSIVAGVFVCLLEYPRGKRK 60
dog-p22phox           MGQIEWAMWANEQALASGLILIMGGIVATAGQFTKWYFGAYSIGAGVFVCLLEYPRGKRR 60
frog-p22phox          MGQIEWAMWANEQALASGLILLAGGIVAVAGQFKGWQFGAYGVAAGVFITLLEYPRSKRK 60
tetraodon-p22phox     MGKIEWAMWANEQALASGFILLAGGIVGVAGRFRGWEFAAYAVAAGVFVCLLEYPRSKRS 60
fugu-p22phox          MGKIEWAMWANEQALASGFILLTGGVVGVAGQFRGWQFAAYAVAAGVLVCLLEYPRSKRS 60
zebrafish-p22phox     MAKIEWAMWANEQALAAGLIYLTGGIVGVAGQFRGWQFAAFGIAAGVFVCLLEYPRSKRG 60
                      *  ************* * *   ** *  ** *  * * *    ***   ****** **

mouse-p22phox         KGSTMERCGQKYLTSVVKLFGPLTRNYYVRAALHFLLSVPAGFLLATILGTVCLAIASVI 120
rat-p22phox           KGSTMERCGQKYLTAVVKLFGPLTRNYYVRAVLHLLLSVPAGFLLATILGTVCLAIASVI 120
Human-p22phox         KGSTMERWGQKYMTAVVKLFGPFTRNYYVRAVLHLLLSVPAGFLLATILGTACLAIASGI 120
dog-p22phox           KGSTMERCGQKYMTKVVKVFGPLSRNYYIRAFLHLGLSVPAGFLLATILGTACLAIASSI 120
frog-p22phox          KGSTMERCGQKYLAAVVKLFGPLTRNYYVRAILHAGLAVPGGFILSTILGTVCLGIASII 120
tetraodon-p22phox     KGTSVERPGQHCFTVCVKAFGPVTKNYYVRAVLHAAICVPGGFMLATVLGCVCLGIASII 120
fugu-p22phox          KGTSVERPGQRCFTVCVKAFGPVTRNYYVRAVLHAAICVPGGFMLATVLGCVCLGIASII 120
zebrafish-p22phox     KGTSIERSGQYCFTVCVKSFGPLTRNYYVRAFLHAALCVPGGFMLATVLGCVCLGMASLI 120
                      **   ** **      ** ***   *** ** **    ** ** * * **  **  ** *

mouse-p22phox         YLLAAIRGEQWTPIEPKPKERPQVGGTIKQPPTNPPPRPPAEVRKKPSEGEEEAASAG-- 178
rat-p22phox           YLLAAIRGEQWTPIEPKPKERPQVGGTIKQPPTNPPPRPPAEVRKKPSEAEEEAASAG-- 178
Human-p22phox         YLLAAVRGEQWTPIEPKPRERPQIGGTIKQPPSNPPPRPPAEARKKPSEEEAAVAAGGPP 180
dog-p22phox           YLLAAYHGEQWIPIEPQPKERPQVGGTIKQPPSNPPPRPPAEARKKPSEEEEAAGAVGVS 180
frog-p22phox          YFLAAIRGEEWRPIEKQAEPKPRAGETIKRPPENPPPRPPAEVRRK----QADEVSVG-- 174
tetraodon-p22phox     YLVAAIRGEHWEPILPKKEIQKPVAESIKNPPQNPPPRPPAETRRKRVDDLEAAAYDN-- 178
fugu-p22phox          YLVAAIRGEHWEPILPSKEIRKPVAESIKNPPQNPPPRPPADTRRKRVEDLEAASYDN-- 178
zebrafish-p22phox     YLSAPIHGEHWEPILH-IETKKRLGESIKEPPQNPPPRPPPELRRKKADNLDAAAYDN-- 177
                      *  *   ** * **             ** ** *******   * *

mouse-p22phox         -GPQVNPMPVTDEVV 192
rat-p22phox           -GPQVNPIPVTDEVV 192
Human-p22phox         GGPQVNPIPVTDEVV 195
```

```
dog-p22phox            GGPQENPVPVIDEVV 195
frog-p22phox           -GGHVNPIPVTDNV- 187
tetraodon-p22phox      ------P-------- 179
fugu-p22phox           ------PISVTANE- 186
zebrafish-p22phox      ------PMSVTINE- 185
                             *
```
